# Bimagrumab plus semaglutide alone or in combination for the treatment of obesity: a randomized phase 2 trial

**DOI:** 10.1038/s41591-026-04204-0

**Published:** 2026-03-02

**Authors:** Steven B. Heymsfield, Louis J. Aronne, Penelope Montgomery, Lloyd B. Klickstein, Laura A. Coleman, Kiran Dole, Linda Mindeholm, Susan Spruill, Xingyuan Li, Kenneth M. Attie

**Affiliations:** 1 Pennington Biomedical Research Center, Louisiana State University, Baton Rouge, LA USA; 2 Weill Cornell Medicine, New York, NY USA; 3 Optimal Clinical Trials, Auckland, New Zealand; 4 Koslapp Therapeutics Inc., Newton, MA USA; 5 Eli Lilly and Company, Indianapolis, IN USA; 6 Independent Consultant, Loulé, Portugal; 7 Applied Statistics and Consulting, Spruce Pine, NC USA

**Keywords:** Obesity, Quality of life

## Abstract

Bimagrumab is an investigational antibody targeting type II activin receptors, intended to reduce total body and visceral fat mass and promote muscle growth. In this double-blind, placebo-controlled phase 2, trial, 507 adults with obesity (body mass index ≥30 kg m^−^^2^ or ≥27 kg m^−^^2^ with at least one obesity-associated complication (excluding diabetes) were randomized to nine groups (1:1:1:1:1:1:1:1:1 ratio) to receive treatment for 48 weeks: placebo, bimagrumab (10 mg kg^−1^ or 30 mg kg^−1^ intravenously every 12 weeks), semaglutide (1.0 mg or 2.4 mg subcutaneously once a week) and combinations thereof. An open-label treatment extension to week 72 followed. Randomization was stratified by sex across the treatment groups. The primary and secondary endpoints were absolute change from baseline in body weight at week 48 and week 72, respectively. The least squares mean absolute changes in body weight at week 48 were −9.3 kg (bimagrumab 30 mg kg^−1^), −14.2 kg (semaglutide 2.4 mg) and −17.8 kg (bimagrumab 30 mg kg^−1^ plus semaglutide 2.4 mg—that is, high-dose combination) versus −3.3 kg (placebo) (all *P* < 0.001 versus placebo). Continued improvements were observed through week 72. Common adverse events for bimagrumab included muscle spasms, diarrhea and acne, and semaglutide was associated with nausea, diarrhea, constipation and fatigue. Bimagrumab plus semaglutide resulted in substantial reductions in body weight, and safety was consistent with the known safety profiles of both drugs. ClinicalTrials.gov identifier: NCT05616013.

## Abstract (web summary)

Bimagrumab is a monoclonal antibody targeting type II activin receptors. In a randomized trial, bimagrumab combined with semaglutide led to up to 17.8 kg weight reduction, surpassing the effects of semaglutide alone (up to 14.2 kg). Notably, the combination preserved lean mass while reducing visceral fat, highlighting its potential for effective obesity management.

## Main

Obesity is a chronic disease projected to affect nearly 3.3 billion adults worldwide by 2035, with an estimated economic impact exceeding USD $4 trillion^1^. Excess adipose tissue, particularly visceral adipose tissue (VAT), increases the risk of obesity complications and related diseases, including metabolic and cardiovascular diseases^2,3^. Most weight reduction with caloric restriction, including with pharmacotherapy, is attributable to reduction in body fat mass; the remainder (approximately 25–40%) can be attributed to lean tissues, including skeletal muscle and visceral organs^4–6^. Patients with obesity who are at risk for low muscle mass, affecting both physical and metabolic function, may benefit from treatments that maximize fat mass reduction while preserving skeletal muscle^7^.

Bimagrumab is a fully human, recombinant monoclonal antibody that targets activin type II receptors (ActRIIA and ActRIIB), preventing binding of natural ligands. Due to its inhibition of myostatin and activin A signaling via the ActRII−activin receptor-like kinase 4 (ALK4) pathway, which leads to anabolic effects in skeletal muscle, bimagrumab was initially developed to treat muscle-related disorders^8^. More recently, activin signaling via the ActRII−activin receptor-like kinase 7 (ALK7) pathway in adipose tissue has been recognized as an important regulator of abdominal obesity, based on exome-wide sequencing analysis indicating the importance of the *INHBE* (inhibin subunit beta E) gene that encodes activin E and the *ACVR1C* (activin A receptor type 1C) gene that encodes ALK7 (refs. ^9,10^). By blocking ligand signaling in adipose tissue, bimagrumab increases lipid mobilization and lipolysis, leading to reduction in fat mass^11^. In a phase 2, 48-week study in adults with obesity and type 2 diabetes, treatment with bimagrumab significantly reduced total body fat mass, VAT and intrahepatic fat while increasing lean mass and lowering glycated hemoglobin (HbA1c)^12^. The study provided evidence that uncoupling of fat and lean mass loss is feasible during weight reduction.

Incretin-based therapies reduce body weight primarily by targeting the central mechanisms that regulate energy balance, thereby decreasing appetite and food intake^13^. Conversely, bimagrumab does not appear to affect food intake but primarily targets activin signaling in adipose tissue and skeletal muscle directly, leading to fat mass reduction and muscle growth^8,11,12^. In preclinical animal models with diet-induced obesity, combining bimagrumab with incretins such as semaglutide or tirzepatide resulted in enhanced fat loss and preservation of muscle mass^14,15^. Treatment with bimagrumab also prevented decreases in thigh muscle volume (assessed by magnetic resonance imaging (MRI)) in a study of low dietary protein intake in healthy volunteers^16^. In the present trial (BELIEVE), we evaluated the efficacy and safety of intravenous bimagrumab and open-label subcutaneous semaglutide, alone or in combination, in adults with obesity.

## Results

### Patient disposition

**Fig. 1:**
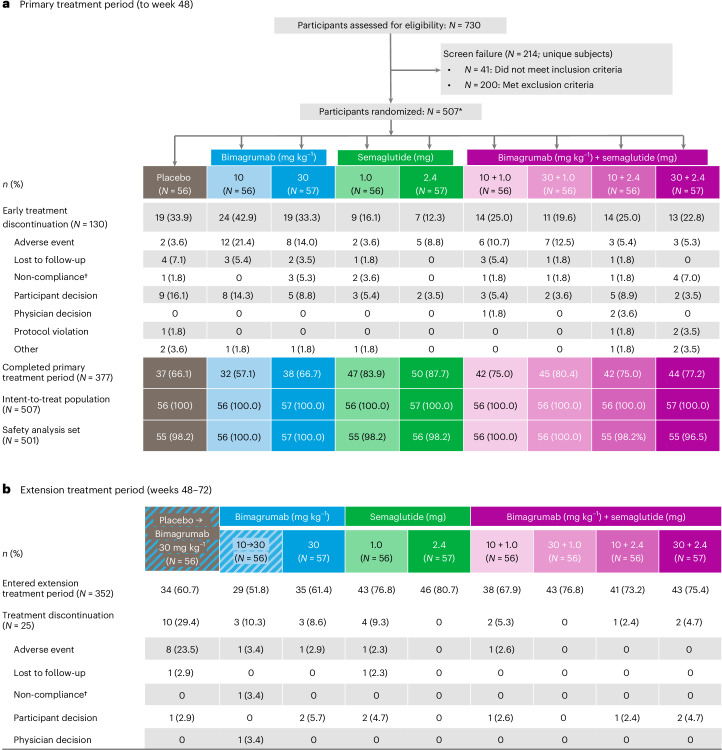
Participant disposition from randomization to week 72. *12 participants met eligibility criteria but were not randomized, and three participants did not meet all eligibility criteria but were randomized. †Non-compliance with study requirements. **a**, Participant disposition during primary treatment period (to week 48). **b**, Participant disposition during extension treatment period (weeks 48 to 72).

From 16 November 2022 to 16 May 2024, 730 participants were screened for eligibility. Of these, 507 were randomized to placebo (*n* = 56), bimagrumab (10 mg kg^−1^: *n* = 56; 30 mg kg^−1^: *n* = 57), semaglutide (1.0 mg: *n* = 56; 2.4 mg: *n* = 57) and bimagrumab plus semaglutide (combination; 10 mg kg^−1^ plus 1.0 mg: *n* = 56; 30 mg kg^−1^ plus 1.0 mg: *n* = 56; 10 mg kg^−1^ plus 2.4 mg: *n* = 56; 30 mg kg^−1^ plus 2.4 mg: *n* = 57) groups (Fig. 1). Overall, 377 (74.4%) participants completed the primary treatment period at week 48. Treatment discontinuations due to adverse events were higher in the bimagrumab groups (14.0–21.4%) than in the semaglutide (3.6–8.8%), combination (5.3–12.5%) and placebo (3.6%) groups. During the extension period, 25 (7.1%) participants discontinued treatment (Fig. 1).

### Baseline demographics and clinical characteristics

Demographic and clinical baseline characteristics were largely similar across treatment groups; most participants were female (57.4%) and White (75.1%) (Table 1). Mean values for the trial were as follows: age 47.5 years, body weight 107.5 kg, body mass index (BMI) 37.3 kg m^−^^2^, waist circumference 118.1 cm, total body fat mass (by dual-energy X-ray absorptiometry (DXA)) 45.8 kg and total body lean mass (by DXA) 58.3 kg (Table 1).

**Table 1:** Demographic and clinical characteristics of the participants at baseline

Characteristic	Placebo (*N* = 56)	Bimagrumab		Placebo + semaglutide		Bimagrumab + semaglutide				Total (*N* = 507)
		10 mg kg^−1^ (*N* = 56)	30 mg kg^−1^ (*N* = 57)	1.0 mg (*N* = 56)	2.4 mg (*N* = 57)	10 mg kg^−1^ + 1.0 mg (*N* = 56)	10 mg kg^−1^ + 2.4 mg (*N* = 56)	30 mg kg^−1^ + 1.0 mg (*N* = 56)	30 mg kg^−1^ + 2.4 mg (*N* = 57)	
Age, years	47.8 (14.6)	44.4 (10.9)	49.2 (12.2)	50.3 (11.2)	49.6 (11.8)	44.8 (12.0)	46.2 (11.7)	47.7 (10.4)	47.5 (12.7)	47.5 (12.1)
Female sex, *n* (%)	32 (57.1)	32 (57.1)	33 (57.9)	32 (57.1)	33 (57.9)	32 (57.1)	32 (57.1)	32 (57.1)	33 (57.9)	291 (57.4)
Race^a^, *n* (%)										
Asian	1 (1.8)	1 (1.8)	1 (1.8)	0 (0.0)	3 (5.3)	1 (1.8)	4 (7.1)	1 (1.8)	3 (5.3)	15 (3.0)
Black	6 (10.7)	3 (5.4)	4 (7.0)	7 (12.5)	5 (8.8)	7 (12.5)	5 (8.9)	6 (10.7)	3 (5.3)	46 (9.1)
White	38 (67.9)	44 (78.6)	45 (78.9)	40 (71.4)	46 (80.7)	41 (73.2)	39 (69.6)	43 (76.8)	45 (78.9)	381 (75.1)
Other or unknown	11 (19.6)	8 (14.3)	7 (12.3)	9 (16.1)	3 (5.3)	7 (12.5)	8 (14.3)	6 (10.7)	6 (10.5)	65 (12.8)
Hispanic or Latino, *n* (%)	6 (10.7)	11 (19.6)	3 (5.3)	4 (7.1)	2 (3.5)	5 (8.9)	7 (12.5)	7 (12.5)	8 (14.0)	53 (10.5)
Duration of obesity, years	16.6 (12.8)	14.1 (11.9)	15.1 (11.7)	18.8 (13.3)	15.1 (10.9)	16.6 (14.1)	14.3 (11.1)	13.7 (11.8)	15.2 (11.6)	15.5 (12.2)
Body weight, kg	109.6 (19.3)	105.1 (19.5)	109.1 (17.6)	105.8 (20.0)	104.5 (14.8)	108.4 (18.9)	108.8 (18.4)	108.2 (14.3)	108.1 (18.5)	107.5 (18.0)
BMI, kg m^−^^2^	38.0 (5.5)	36.5 (5.3)	37.5 (4.6)	36.6 (5.8)	36.6 (5.1)	37.9 (5.0)	38.0 (5.8)	37.1 (3.7)	37.5 (5.5)	37.3 (5.2)
BMI category, *n* (%)										
<30	1 (1.8)	3 (5.4)	1 (1.8)	8 (14.5)	3 (5.4)	2 (3.6)	5 (9.1)	1 (1.8)	3 (5.5)	27 (5.4)
≥30 to <35	16 (29.1)	22 (39.3)	17 (29.8)	15 (27.3)	21 (37.5)	16 (28.6)	15 (27.3)	15 (26.8)	20 (36.4)	157 (31.3)
≥35 to <40	22 (40.0)	17 (30.4)	22 (38.6)	15 (27.3)	18 (32.1)	17 (30.4)	16 (29.1)	27 (48.2)	14 (25.5)	168 (33.5)
≥40	16 (29.1)	14 (25.0)	17 (29.8)	17 (30.9)	14 (25.0)	21 (37.5)	19 (34.5)	13 (23.2)	18 (32.7)	149 (29.7)
Waist circumference, cm	119.1 (14.1)	116.8 (13.4)	119.3 (10.9)	117.9 (13.6)	117.3 (11.5)	117.8 (12.7)	119.3 (14.0)	117.9 (10.3)	118.0 (12.5)	118.1 (12.5)
Total body fat mass (DXA), kg	48.2 (12.1)	43.9 (12.5)	47.0 (9.6)	43.8 (10.8)	43.8 (10.9)	47.1 (11.7)	46.3 (11.8)	45.2 (9.3)	46.9 (11.1)	45.8 (11.1)
Total body fat mass (DXA), percent	43.9 (7.4)	41.9 (7.9)	43.9 (7.0)	41.8 (7.3)	41.9 (7.9)	43.5 (7.4)	42.7 (6.8)	42.4 (7.8)	43.7 (6.8)	42.9 (7.4)
Total body lean mass (DXA), kg	58.8 (13.1)	57.7 (12.8)	57.7 (13.1)	58.6 (14.5)	57.7 (10.6)	58.2 (12.6)	58.7 (11.2)	59.4 (12.8)	57.6 (12.1)	58.3 (12.5)
Total body lean mass (DXA), percent	53.6 (7.1)	55.4 (7.7)	53.5 (6.8)	55.6 (7.1)	55.4 (7.6)	54.0 (7.2)	54.7 (6.6)	55.1 (7.5)	53.8 (6.6)	54.6 (7.1)
VAT (DXA), kg	1.7 (1.0)	1.3 (0.6)	1.6 (0.8)	1.4 (0.7)	1.4 (0.6)	1.4 (0.8)	1.7 (1.1)	1.4 (0.6)	1.4 (0.7)	1.5 (0.8)
HbA1c, percent	5.5 (0.4)	5.4 (0.4)	5.5 (0.3)	5.4 (0.5)	5.4 (0.4)	5.4 (0.3)	5.5 (0.5)	5.4 (0.3)	5.4 (0.3)	5.4 (0.4)
SF-36 Physical Functioning score (transformed)^b^	72.6 (19.6)	73.8 (20.1)	70.3 (18.7)	69.1 (23.0)	74.1 (19.5)	73.3 (23.5)	77.1 (16.9)	72.7 (19.7)	70.7 (24.5)	72.6 (20.7)
IWQOL-Lite-CT Physical Function score	53.6 (21.4)	52.6 (19.1)	51.9 (19.0)	51.2 (20.8)	51.8 (17.6)	56.0 (18.9)	56.0 (20.5)	54.3 (18.1)	50.8 (19.7)	53.1 (19.4)
SBP, mmHg	131.0 (14.5)	125.1 (10.1)	128.0 (14.3)	133.9 (13.5)	131.4 (14.5)	128.9 (13.2)	128.3 (13.4)	129.4 (11.7)	128.4 (12.0)	129.4 (13.2)
DBP, mmHg	81.5 (9.9)	82.1 (7.5)	80.3 (8.6)	84.0 (7.6)	84.7 (7.6)	83.3 (6.8)	81.1 (8.3)	83.8 (7.5)	83.0 (9.9)	82.7 (8.3)
Pulse rate, bpm	70.7 (12.8)	73.0 (10.8)	70.6 (8.5)	71.8 (11.2)	71.0 (10.0)	71.0 (10.0)	71.1 (10.2)	72.4 (9.9)	72.2 (9.5)	71.5 (10.3)
hsCRP^c^, mg l^−1^	3.0 (121.1)	2.4 (171.6)	3.5 (133.1)	2.1 (97.4)	2.8 (128.1)	3.2 (139.6)	3.7 (163.0)	2.4 (123.5)	2.5 (111.2)	2.8 (132.9)
Total body BMD, g cm^−2^	1.2 (0.2)	1.2 (0.2)	1.3 (0.1)	1.2 (0.2)	1.2 (0.1)	1.2 (0.2)	1.3 (0.1)	1.2 (0.2)	1.2 (0.2)	1.2 (0.2)
Lumbar spine BMD, g cm^−2^	1.2 (0.2)	1.1 (0.2)	1.2 (0.2)	1.2 (0.2)	1.2 (0.2)	1.1 (0.2)	1.2 (0.2)	1.1 (0.2)	1.1 (0.2)	1.2 (0.2)
Total hip BMD, g cm^−2^	1.1 (0.1)	1.1 (0.1)	1.1 (0.1)	1.1 (0.2)	1.1 (0.1)	1.0 (0.2)	1.1 (0.2)	1.1 (0.1)	1.0 (0.1)	1.1 (0.1)
Femoral neck BMD, g cm^−2^	1.0 (0.2)	1.0 (0.2)	1.0 (0.2)	0.9 (0.2)	1.0 (0.1)	1.0 (0.2)	1.0 (0.2)	1.0 (0.2)	0.9 (0.2)	1.0 (0.2)
Total cholesterol^c^, mg dl^−1^	184.2 (23.0)	189.8 (21.7)	191.2 (16.7)	188.4 (22.0)	188.8 (18.4)	185.3 (22.5)	179.3 (17.6)	188.6 (21.7)	193.9 (18.3)	187.8 (20.2)
HDL cholesterol^c^, mg dl^−1^	47.2 (27.0)	46.7 (26.6)	45.3 (30.1)	48.4 (26.3)	47.0 (27.9)	47.6 (27.7)	46.9 (21.9)	46.1 (28.2)	47.1 (25.0)	46.9 (26.7)
LDL cholesterol^c^, mg dl^−1^	109.7 (39.0)	117.3 (28.4)	113.7 (41.5)	115.7 (32.7)	118.4 (29.6)	112.1 (36.4)	109.3 (24.6)	115.3 (35.8)	117.4 (26.8)	114.3 (32.9)
Triglycerides^c^, mg dl^−1^	121.8 (35.5)	112.4 (56.3)	122.1 (47.1)	112.2 (44.5)	113.6 (36.1)	108.6 (49.2)	110.4 (47.1)	120.6 (53.1)	128.2 (56.6)	116.5 (47.5)
Leptin^c^, ng ml^−1^	47.0 (86.4)	42.0 (64.4)	42.7 (76.5)	42.3 (73.9)	45.5 (86.8)	47.0 (64.8)	41.4 (79.6)	49.0 (85.0)	44.5 (84.2)	44.5 (77.6)
Adiponectin^c^, µg ml^−1^	21.0 (58.3)	21.9 (68.2)	21.0 (55.9)	22.5 (78.9)	19.9 (79.4)	25.4 (77.3)	18.9 (60.1)	19.5 (57.0)	24.3 (57.4)	21.5 (66.5)
Grip strength, kg	32.6 (12.8)	36.2 (12.8)	35.8 (13.6)	34.6 (14.2)	34.8 (15.6)	33.2 (13.8)	35.1 (14.0)	34.6 (14.1)	33.2 (13.7)	34.5 (13.8)
Fasting insulin^c^, pmol l^−1^	110.4 (80.4)	100.7 (101.0)	117.2 (82.0)	85.7 (131.3)	113.7 (69.9)	116.6 (71.8)	104.6 (77.4)	112.4 (115.4)	111.9 (68.6)	107.8 (88.6)
Free testosterone^c^, nmol l^−1^	2.2 (365.9)	1.9 (474.4)	1.9 (368.4)	2.7 (319.7)	2.2 (342.3)	2.1 (524.3)	2.9 (257.7)	2.6 (372.4)	2.3 (404.3)	2.3 (368.7)
Median (IQR) total calories, kcal d^−1^	1,783.0 (1,292.0, 2,289.5)	1,675.5 (1,210.0, 2,110.0)	1,619.5 (1,265.0, 2,175.0)	1,625.0 (1,292.0, 2,100.0)	1,837.0 (1,420.0, 2,473.0)	1,708.0 (1,153.0, 2,270.0)	1,493.5 (1,184.0, 1,847.0)	1,677.0 (1,116.8, 2,325.0)	1,710.5 (1,248.0, 2,300.0)	1,670.0 (1,235.0, 2,243.0)
Median (IQR) protein intake, % total calories	20.0 (17.1, 26.6)	20.2 (17.3, 25.7)	20.0 (15.7, 25.8)	19.6 (15.5, 25.6)	18.0 (15.0, 23.2)	20.2 (15.6, 26.0)	21.9 (16.0, 25.0)	21.6 (17.8, 26.9)	20.5 (14.0, 25.0)	20.0 (16.0, 25.0)

Data are presented as mean (standard deviation) unless otherwise indicated. *N* may vary across variables due to missing values.

^a^Race was reported by the participants. Other or unknown includes Native Hawaiian, other Pacific Islander, American Indian, Alaska Native, other, multiple or unknown.

^b^Transformed scores standardized to range from 0 to 100.

^c^Presented as geometric mean (CV [%]).

BMD, bone mineral density; bpm, beats per minute; CV, coefficient of variation; DBP, diastolic blood pressure; DXA, dual-energy X-ray absorptiometry; HbA1c, glycated hemoglobin; HDL, high-density lipoprotein; hsCRP, high-sensitivity C-reactive protein; IQR, interquartile range; IWQOL-Lite-CT, Impact of Weight on Quality of Life-Lite Clinical Trials Version; LDL, low-density lipoprotein; SBP, systolic blood pressure; SF-36, 36-Item Short Form Health Survey; VAT, visceral adipose tissue.

### Primary outcome

**Fig. 2:**
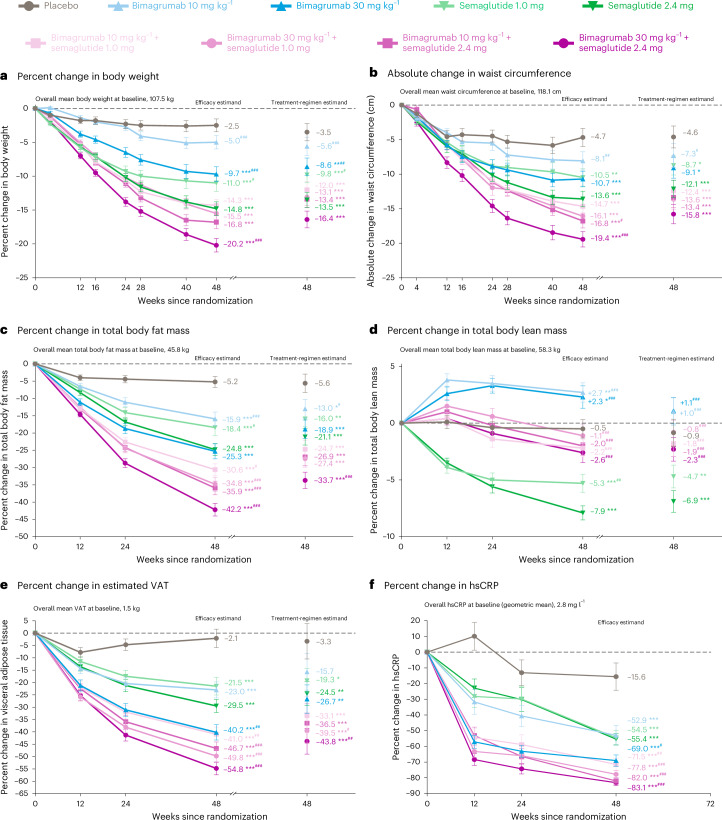
Changes in efficacy endpoints with bimagrumab, semaglutide and bimagrumab plus semaglutide versus placebo (primary treatment period; week 48). *P < 0.05, **P < 0.01, ***P< 0.001 active treatment groups versus placebo. ^#^*P* < 0.05, ^##^*P* < 0.01, ^###^*P* < 0.001 active treatment groups versus semaglutide 2.4 mg. Data are presented as LSM change from baseline ± standard error. *P* values for comparisons with placebo and semaglutide 2.4 mg were calculated using two-sided *t*-tests without multiplicity adjustment. *n* values are provided in the source data tables in the supplementary information. **a**−**e**, The LSM percent or absolute changes from baseline to week 48 in efficacy endpoints are based on an MMRM analysis for the efficacy estimand and an ANCOVA model with multiple imputation for the treatment regimen estimand. **f**, The LSM percent changes in hsCRP from baseline to week 48 are based on MMRM analysis using log transformation for the efficacy estimand. ANCOVA, analysis of covariance; hsCRP, High-sensitivity C-Reactive Protein; LSM, least-squares mean; MMRM, mixed model for repeated measures; VAT, visceral adipose tissue. Source data

For the efficacy results at week 48, nominal *P* values versus placebo and versus semaglutide 2.4 mg are reported in Table 2 and Fig. 2.

**Table 2:** Efficacy endpoints at week 48

Endpoint	Placebo (*N* = 56)	Bimagrumab		Placebo + semaglutide		Bimagrumab + semaglutide			
		10 mg kg^−1^ (*N* = 56)	30 mg kg^−1^ (*N* = 57)	1.0 mg (*N* = 56)	2.4 mg (*N* = 57)	10 mg kg^−1^+ 1.0 mg (*N* = 56)	10 mg kg^−1^+ 2.4 mg (*N* = 56)	30 mg kg^−1^+ 1.0 mg (*N* = 56)	30 mg kg^−1^+ 2.4 mg (*N* = 57)
Primary endpoint									
Body weight, kg	−3.3 ± 1.4	−6.0 ± 1.4	−9.3 ± 1.3	−9.8 ± 1.3	−14.2 ± 1.2	−12.7 ± 1.3	−14.3 ± 1.3	−13.9 ± 1.3	−17.8 ± 1.3
LSM difference from placebo (95% CI)	–	−2.7 (−6.2 to 0.8) *p* = 0.133	−5.9 (−9.5 to −2.4) *p*< 0.001	−6.5 (−10.0 to −2.9) *p* < 0.001	−10.9 (−14.4 to −7.5) *p* < 0.001	−9.4 (−12.9 to −5.9) *p* < 0.001	−11.0 (−14.4 to −7.6) *p* < 0.001	−10.6 (−14.0 to −7.1) *p* < 0.001	−14.5 (−18.0 to −11.0) *p* < 0.001
*P* value versus semaglutide 2.4 mg	*p* < 0.001	*p* < 0.001	*p* = 0.004	*p* = 0.008	–	*p* = 0.373	*p* = 0.971	*p* = 0.825	*p* = 0.039
Secondary endpoints									
Total body fat mass (DXA), kg	−1.9 ± 1.2	−5.7 ± 1.2	−8.6 ± 1.2	−6.7 ± 1.0	−9.6 ± 1.0	−11.2 ± 1.1	−11.9 ± 1.1	−12.2 ± 1.1	−15.6 ± 1.0
*P* value versus placebo	–	*p* = 0.012	*p* < 0.001	*p* = 0.002	*p* < 0.001	*p* < 0.001	*p* < 0.001	*p* < 0.001	*p* < 0.001
*P* value versus semaglutide 2.4 mg	*p* < 0.001	*p* = 0.009	*p* = 0.501	*p* = 0.036	–	*p* = 0.278	*p* = 0.122	*p* = 0.074	*p* < 0.001
Total body lean mass (DXA), kg	−0.5 ± 0.7	0.5 ± 0.7	0.4 ± 0.7	−2.6 ± 0.6	−3.9 ± 0.6	−1.1 ± 0.6	−1.2 ± 0.6	−0.6 ± 0.6	−1.3 ± 0.6
*P* value versus placebo	–	*p* = 0.192	*p* = 0.223	*p* = 0.011	*p* < 0.001	*p* = 0.475	*p* = 0.430	*p* = 0.991	*p* = 0.343
*P* value versus semaglutide 2.4 mg	*p* < 0.001	*p* < 0.001	*p* < 0.001	*p* = 0.065	–	*p* < 0.001	*p* < 0.001	*p* < 0.001	*p* < 0.001
Fat loss index^a^, %	79.2	100	100	72.0	71.1	91.1	90.8	95.3	92.3
VAT (DXA), kg	−0.1 ± 0.1	−0.3 ± 0.1	−0.4 ± 0.1	−0.3 ± 0.1	−0.4 ± 0.1	−0.5 ± 0.1	−0.6 ± 0.1	−0.6 ± 0.1	−0.7 ± 0.1
*P* value versus placebo	–	*p* = 0.010	*p* = 0.001	*p* = 0.017	*p* = 0.001	*p* < 0.001	*p* < 0.001	*p* < 0.001	*p* < 0.001
*P* value versus semaglutide 2.4 mg	*p* = 0.001	*p* = 0.519	*p* = 0.912	*p* = 0.370	–	*p* = 0.041	*p* = 0.002	*p* = 0.006	*p* < 0.001
Appendicular lean mass (DXA), %	−1.2 ± 1.4	1.3 ± 1.4	1.1 ± 1.4	−5.1 ± 1.1	−7.9 ± 1.0	−1.8 ± 1.2	−2.1 ± 1.2	−1.2 ± 1.2	−2.1 ± 1.2
*P* value versus placebo	–	*p* = 0.127	*p* = 0.162	*p* = 0.016	*p* < .001	*p* = 0.709	*p* = 0.555	*p* = 0.962	*p* = 0.552
*P* value versus semaglutide 2.4 mg	*p* < 0.001	*p* < 0.001	*p* < 0.001	*p* = 0.047	–	*p* < 0.001	*p* < 0.001	*p* < 0.001	*p* < 0.001
HbA1c, %	−0.05 (0.05)	−0.17 (0.06)	−0.15 (0.05)	−0.30 (0.05)	−0.36 (0.04)	−0.32 (0.05)	−0.46 (0.05)	−0.37 (0.05)	−0.41 (0.05)
*P* value versus placebo	–	*p* = 0.076	*p* = 0.136	*p* < 0.001	*p* < 0.001	*p* < 0.001	*p* < 0.001	*p* < 0.001	*p* < 0.001
*P* value versus semaglutide 2.4 mg	*p* < 0.001	*p* = 0.006	*p* = 0.001	*p* = 0.303	–	*p* = 0.542	*p* = 0.134	*p* = 0.921	*p* = 0.506
Prediabetes at baseline who achieved normoglycemia^b^, *n*/*N* (%)	6/15 (40)	7/11 (63.6)	12/18 (66.7)	10/13 (76.9)	13/14 (92.9)	10/11 (90.9)	14/14 (100)	11/11 (100)	9/9 (100)
SF-36 Physical Functioning score^c^	12.1 ± 2.2	11.1 ± 2.3	12.6 ± 2.0	10.7 ± 1.9	12.2 ± 1.9	15.3 ± 2.0	13.5 ± 2.0	13.5 ± 2.1	16.6 ± 1.9
*P* value versus placebo	–	*p* = 0.724	*p* = 0.863	*p* = 0.604	*p* = 0.989	*p* = 0.246	*p* = 0.620	*p* = 0.627	*p* = 0.108
*P* value versus semaglutide 2.4 mg	*p* = 0.989	*p* = 0.720	*p* = 0.868	*p* = 0.579	–	*p* = 0.236	*p* = 0.618	*p* = 0.621	*p* = 0.093
IWQOL-Lite-CT Physical Function score	13.3 ± 2.7	15.6 ± 2.7	16.1 ± 2.6	15.3 ± 2.4	19.5 ± 2.3	18.7 ± 2.6	19.3 ± 2.5	21.6 ± 2.6	20.2 ± 2.4
*P* value versus placebo	–	*p* = 0.523	*p* = 0.420	*p* = 0.567	*p* = 0.066	*p* = 0.113	*p* = 0.085	*p* = 0.017	*p* = 0.043
*P* value versus semaglutide 2.4 mg	*p* = 0.066	*p* = 0.243	*p* = 0.301	*p* = 0.188	–	*p* = 0.820	*p* = 0.965	*p* = 0.533	*p* = 0.815
SBP^d^, mmHg	−6.9 (2.1)	−4.6 (2.3)	−3.1 (1.7)	−8.6 (1.7)	−7.9 (1.7)	−4.5 (1.5)	−7.9 (1.9)	−5.3 (1.4)	−10.4 (1.7)
*P* value versus placebo	–	*p* = 0.455	*p* = 0.162	*p* = 0.542	*p* = 0.724	*p* = 0.345	*p* = 0.728	*p* = 0.529	*p* = 0.191
*P* value versus semaglutide 2.4 mg	*p* = 0.724	*p* = 0.255	*p* = 0.051	*p* = 0.774	–	*p* = 0.141	*p* = 0.991	*p* = 0.254	*p* = 0.293
DBP^d^, mmHg	−2.8 (1.3)	−2.5 (1.3)	−3.8 (1.2)	−3.1 (1.1)	−3.4 (1.0)	−2.5 (1.0)	−6.3 (1.3)	−4.2 (1.1)	−6.7 (1.0)
*P* value versus placebo	–	*p* = 0.862	*p* = 0.577	*p* = 0.843	*p* = 0.688	*p* = 0.874	*p* = 0.053	*p* = 0.409	*p* = 0.013
*P* value versus semaglutide 2.4 mg	*p* = 0.688	*p* = 0.549	*p* = 0.826	*p* = 0.836	–	*p* = 0.514	*p* = 0.079	*p* = 0.608	*p* = 0.015
Pulse rate^d^, bpm	−1.4 (1.6)	−3.2 (1.3)	−4.1 (1.3)	−0.5 (1.2)	−0.4 (1.1)	0.5 (1.3)	−2.4 (1.2)	−1.3 (1.3)	−2.2 (1.1)
*P* value versus placebo	–	*p* = 0.398	*p* =0.201	*p* =0.640	*p* =0.582	*p* =0.369	*p* =0.628	*p* =0.942	*p* =0.707
*P* value versus semaglutide 2.4 mg	*p* = 0.582	*p* = 0.090	*p* = 0.026	*p* = 0.930	–	*p* = 0.635	*p* = 0.204	*p* = 0.572	*p* = 0.232
Exploratory endpoints									
Total body BMD^e^, %	−0.2 (0.4)	0.8 (0.4)	0.6 (0.3)	0.3 (0.4)	1.0 (0.3)	1.1 (0.5)	0.2 (0.5)	0.5 (0.4)	0.6 (0.5)
Lumbar spine BMD^e^, %	0.6 (0.5)	−0.6 (0.6)	−0.0 (0.5)	−0.2 (0.5)	0.3 (0.4)	0.3 (0.5)	0.1 (0.5)	−0.8 (0.6)	−0.4 (0.4)
Total hip BMD^e^, %	−0.8 (0.3)	−1.1 (0.4)	−1.5 (0.4)	−1.4 (0.3)	−2.1 (0.4)	−2.0 (0.3)	−1.5 (0.4)	−2.2 (0.4)	−2.3 (0.5)
Femoral neck BMD^e^, %	−0.4 (0.6)	−1.4 (0.5)	−0.6 (0.5)	−0.9 (0.6)	−1.3 (0.6)	−0.8 (0.5)	−1.0 (0.5)	−0.9 (0.4)	−1.8 (0.6)
Total cholesterol^f^, mg dl^−1^	−4.6 ± 4.0	18.6 ± 5.9	20.3 ± 6.0	−18.0 ± 3.4	−19.1 ± 2.7	15.7 ± 4.5	2.2 ± 4.1	9.2 ± 5.4	1.1 ± 4.3
HDL cholesterol^f^, mg dl^−1^	0.6 ± 1.1	−2.9 ± 1.1	0.5 ± 1.2	0.4 ± 1.0	0.9 ± 0.9	−0.1 ± 1.0	0.4 ± 1.1	1.9 ± 1.1	1.6 ± 0.9
LDL cholesterol^f^, mg dl^−1^	−2.6 ± 3.3	21.2 ± 5.0	23.3 ± 7.8	−14.8 ± 3.1	−14.0 ± 2.4	17.0 ± 3.9	7.8 ± 3.9	12.9 ± 4.8	11.1 ± 4.3
Triglycerides^f^, mg dl^−1^	−3.7 ± 5.2	8.6 ± 9.1	8.3 ± 7.2	−19.5 ± 4.4	−26.5 ± 4.0	−5.9 ± 7.0	−24.8 ± 4.6	−21.7 ± 5.1	−29.0 ± 4.2
Fasting insulin^f^, pmol l^−1^	5.2 (8.0)	−11.0 (7.9)	−26.8 (5.0)	−27.1 (7.2)	−39.3 (4.9)	−36.9 (6.2)	−33.3 (5.2)	−30.6 (8.0)	−26.9 (6.5)
Free testosterone^f^, nmol l^−1^	0.3 (0.2)	0.0 (0.1)	0.3 (0.2)	0.1 (0.1)	-0.1 (0.1)	0.4 (0.2)	0.3 (0.1)	0.7 (0.1)	0.3 (0.2)
Grip strength^d^, kg	1.7 (0.9)	3.6 (1.1)	3.6 (1.1)	1.7 (0.9)	2.2 (1.0)	2.3 (1.0)	2.9 (0.9)	4.8 (1.1)	2.3 (1.5)
Median (IQR) change in total calories, kcal d^−1^	−399.0 (−906, 59)	−148.0 (−582, 463)	−122.0 (−769, 227.5)	−53.5 (−409, 401.5)	−316.7 (−786, 60)	−143.0 (−954, 362)	−87.0 (−550, 70)	−185.5 (−459, 176.9)	−245.0 (−808, 94.5)
Median (IQR) change in protein, % total calories	6.0 (−1.4, 11.1)	4.0 (−5.2, 9.1)	6.0 (1, 12)	0.1 (−4, 7.5)	3.9 (−1, 10.5)	2.0 (−5.1, 8)	1.0 (−5, 7)	2.1 (−0.8, 8)	0.4 (−4.5, 11)

Data are presented as LSM change from baseline ± standard error unless specified otherwise. All analyses were conducted in the intention-to-treat population (except blood pressure, pulse rate, total calories and protein intake for which the safety analysis set was used). The LSM changes from baseline in primary and secondary endpoints at week 48 are based on an ANCOVA model with multiple imputation for the treatment regimen estimand. Statistical inference for endpoint uses Rubin’s rule to combine analyses of imputed datasets. Missing values at week 48 were imputed 100 times based on observed data in the placebo group. The confidence intervals (CIs) and *P* values for comparisons with placebo and semaglutide 2.4 mg were calculated using two-sided *t*-tests without multiplicity adjustment.

^a^Fat loss index is defined as change in total body fat mass (measured using DXA) in kilograms, divided by sum of change in total body fat mass and change in total body lean mass in kilograms times 100 (with maximum value = 100).

^b^The proportion of participants achieving normoglycemia (HbA1c < 5.7%) among those with baseline HbA1c ≥ 5.7% is based on logistic regression with MMRM used for missing data imputation at week 48 for the efficacy estimand. *N*, number of participants with imputed data; *n*, number of participants achieving target with imputed data.

^c^Transformed scores standardized to range from 0 to 100.

^d^The LSM changes from baseline at week 48 in SBP, DBP, pulse rate and grip strength are based on MMRM for the efficacy estimand.

^e^The LSM percent changes from baseline at week 48 in total body, lumbar spine, total hip and femoral neck BMD are based on MMRM for the efficacy estimand.

^f^The LSM changes from baseline at week 48 in lipids, fasting insulin and free testosterone are based on MMRM using log transformation for the efficacy estimand.

ANCOVA, analysis of covariance; BMD, bone mineral density; bpm, beats per minute; DBP, diastolic blood pressure; DXA, dual-energy X-ray absorptiometry; HbA1c, glycated hemoglobin; IQR, interquartile range; IWQOL-Lite-CT, Impact of Weight on Quality of Life-Lite Clinical Trials Version; LDL, low-density lipoprotein; LSM, least-squares mean; MMRM, mixed model for repeated measures; SBP, systolic blood pressure; SF-36, 36-Item Short Form Health Survey; VAT, visceral adipose tissue.

#### Absolute change in body weight

##### Treatment regimen estimand

At week 48, the least squares mean (LSM) absolute change in body weight was −6.0 kg to −9.3 kg (bimagrumab), −9.8 kg to −14.2 kg (semaglutide) and −12.7 kg to −17.8 kg (combination) versus −3.3 kg (placebo) (all *P* < 0.001 versus placebo, except bimagrumab 10 mg kg^−1^; Table 2). The LSM change in absolute body weight was greater with the high-dose combination versus semaglutide 2.4 mg (−17.8 kg versus −14.2 kg; *P* < 0.05).

### Secondary outcomes

#### Percent change in body weight

##### Treatment regimen estimand

The LSM percent change in body weight was −5.6% to −8.6% (bimagrumab), −9.8% to −13.5% (semaglutide) and −12.0% to −16.4% (combination) compared to −3.5% (placebo) (Fig. 2a). By week 48, ≥15% weight reduction was achieved in 23.3% (bimagrumab 30 mg kg^−1^), 43.4% (semaglutide 2.4 mg) and 63.9% (high-dose combination) of participants (Extended Data Fig. 2).

##### Efficacy estimand

**Fig. 3:**
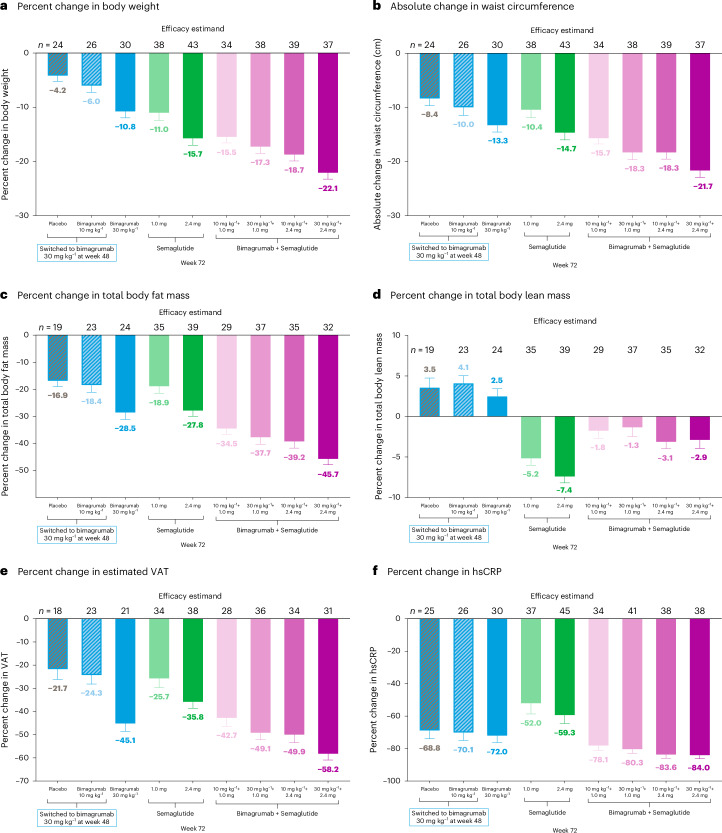
Changes in efficacy endpoints with bimagrumab, semaglutide and bimagrumab plus semaglutide versus placebo (primary plus extension treatment period; week 72). Data are presented as LSM change from baseline ± standard error. *n* represents the number of participants with baseline and post-baseline values at week 72. **a**−**e**, The LSM percent or absolute changes from baseline at week 72 in efficacy endpoints are based on MMRM analysis for the efficacy estimand. **f**, The LSM percent changes in hsCRP from baseline to week 72 are based on MMRM analysis using log transformation for the efficacy estimand. hsCRP, high-sensitivity C-reactive protein; LSM, least-squares mean; MMRM, mixed model for repeated measures; VAT, visceral adipose tissue. Source data

The LSM percent change in body weight at week 48 was −5.0% to −9.7% (bimagrumab), −11.0% to −14.8% (semaglutide) and −14.3% to −20.2% (combination) versus −2.5% (placebo) (*P* < 0.001 for the high-dose combination versus semaglutide 2.4 mg; Fig. 2a). At week 72, the LSM change in weight was −12.0 kg (−10.8%; bimagrumab 30 mg kg^−1^), −16.5 kg (−15.7%; semaglutide 2.4 mg) and −24.2 kg (−22.1%; high-dose combination) (Fig. 3a and Extended Data Table 1). By week 72, ≥15% weight reduction was achieved in 21.8% (bimagrumab 30 mg kg^−1^), 51.8% (semaglutide 2.4 mg) and 84.9% (high-dose combination) of participants (Extended Data Fig. 2).

#### Waist circumference

##### Treatment regimen estimand

Absolute LSM change in waist circumference at week 48 was −7.3 cm to −9.1 cm (bimagrumab), −8.7 cm to −12.1 cm (semaglutide) and −12.4 cm to −15.8 cm (combination) compared to −4.6 cm (placebo) (Fig. 2b). Results for improvements in waist-to-height ratio categories at week 48 are provided in Extended Data Fig. 3.

##### Efficacy estimand

Absolute LSM changes in waist circumference at week 48 were −8.1 cm to −10.7 cm (bimagrumab), −10.5 cm to −13.6 cm (semaglutide) and −14.7 cm to −19.4 cm (combination) compared to −4.7 cm (placebo) (*P* < 0.001 for the high-dose combination versus semaglutide 2.4 mg; Fig. 2b). At week 72, absolute LSM changes in waist circumference were −13.3 cm (bimagrumab 30 mg kg^−1^), −14.7 cm (semaglutide 2.4 mg) and −21.7 cm (high-dose combination) (Fig. 3b).

#### Body composition (by DXA)

##### Treatment regimen estimand

The LSM percent reduction in total body fat mass at week 48 was −13.0% to −18.9% (bimagrumab), −16.0% to −21.1% (semaglutide) and −24.7% to −33.7% (combination) compared to −5.6% (placebo) (*P* < 0.001 for the high-dose combination versus semaglutide 2.4 mg; Fig. 2c). Mean percent body fat decreased from 43.7% at baseline to 32.5% at week 48 with high-dose combination compared to 41.9% to 37.9% with semaglutide 2.4 mg. By week 48, fat mass reduction ≥25% was achieved in 30.8% (bimagrumab 30 mg kg^−1^), 36.3% (semaglutide 2.4 mg) and 73.6% (high-dose combination) of participants (Extended Data Fig. 4).

The LSM percent changes in total body lean mass at week 48 were +1.0% to +1.1% (bimagrumab), −4.7% to −6.9% (semaglutide) and −0.8% to −2.3% (combination) versus −0.9% (placebo) (*P* < 0.001 for all combination groups versus semaglutide 2.4 mg; Fig. 2d). At week 48, the proportion of weight loss due to fat mass (fat loss index) was 100% (bimagrumab 30 mg kg^−1^), 71.1% (semaglutide 2.4 mg) and 92.3% (high-dose combination). The bimagrumab 30 mg kg^−1^ plus semaglutide 1.0 mg group showed the greatest preservation of lean mass among the combination groups. Results for appendicular lean mass at week 48 are presented in Table 2.

The percent LSM changes in estimated VAT were −15.7% to −26.7% (bimagrumab), −19.3% to −24.5% (semaglutide) and −33.1% to −43.8% (combination) versus −3.3% (placebo) (*P* < 0.01 for the high-dose combination versus semaglutide 2.4 mg; Fig. 2e).

##### Efficacy estimand

The LSM percent reduction in total body fat mass at week 48 was −15.9% to −25.3% (bimagrumab), −18.4% to −24.8% (semaglutide) and −30.6% to −42.2% (combination) compared to −5.2% (placebo) (*P* < 0.05 for all combination groups versus semaglutide 2.4 mg; Fig. 2c). At week 72, LSM percent reductions in total body fat mass were −28.5% (bimagrumab 30 mg kg^−1^), −27.8% (semaglutide 2.4 mg) and −45.7% (high-dose combination) (Fig. 3c). By week 72, fat mass reduction ≥30% was achieved in 50.0% (bimagrumab 30 mg kg^−1^), 36.4% (semaglutide 2.4 mg) and 94.0% (high-dose combination) of participants (Extended Data Fig. 4).

The LSM percent changes in total body lean mass at week 48 were +2.3% to +2.7% (bimagrumab), −5.3% to −7.9% (semaglutide) and −1.1% to −2.6% (combination) versus −0.5% (placebo) (*P* < 0.001 for all combination groups versus semaglutide 2.4 mg; Fig. 2d). At week 72, LSM changes in total body lean mass were +2.5% (bimagrumab 30 mg kg^−1^), −7.4% (semaglutide 2.4 mg) and −2.9% (high-dose combination) (Fig. 3d). Results for appendicular lean mass at week 72 are presented in Extended Data Table 1.

At week 72, the proportion of weight loss due to fat mass was 100% (bimagrumab 30 mg kg^−1^), 75.6% (semaglutide 2.4 mg) and 92.2% (high-dose combination) (Extended Data Table 1).

At week 48, the percent LSM changes in estimated VAT were −23.0% to −40.2% (bimagrumab), −21.5% to −29.5% (semaglutide) and −41.0% to −54.8% (combination) versus −2.1% (placebo) (*P* < 0.01 for all combination groups versus semaglutide 2.4 mg; Fig. 2e). At week 72, reductions in estimated VAT were −45.1% (bimagrumab 30 mg kg^−1^), −35.8% (semaglutide 2.4 mg) and −58.2% (high-dose combination) (Fig. 3e).

### Metabolic parameters and physical function

#### HbA1c

HbA1c levels improved in semaglutide and combination groups at week 48 (Table 2; treatment regimen estimand). The LSM decreases in HbA1c levels at week 72 were −0.23% (bimagrumab 30 mg kg^−1^), −0.40% (semaglutide 2.4 mg) and −0.55% (high-dose combination) (Extended Data Table 1; efficacy estimand). Among participants with HbA1c ≥ 5.7% at baseline (prediabetes), normoglycemia (defined as HbA1c < 5.7%) at week 48 was achieved in 19 of 29 participants (66%, bimagrumab groups), in 23 of 27 participants (85%, semaglutide groups) and in 44 of 45 participants (98%, combination groups), compared to six of 15 participants (40%) in the placebo group (Table 2; efficacy estimand). At week 72, normoglycemia was achieved in 22 of 29 participants (76%, bimagrumab groups), in 26 of 27 participants (96%, semaglutide groups) and in 45 of 45 participants (100%, combination groups) compared to eight of 15 participants (53%) in the placebo group (Extended Data Table 1; efficacy estimand).

#### Patient-reported outcomes

##### Treatment regimen estimand

Improvements in 36-Item Short Form Health Survey (SF-36) Physical Functioning score were similar across treatment groups at week 48 (*P* value not significant versus placebo; Table 2). Combination groups containing bimagrumab 30 mg kg^−1^ showed greater improvements in Impact of Weight on Quality of Life-Lite Clinical Trials Version (IWQOL-Lite-CT) Physical Function scores at week 48 compared to the placebo group (*P* < 0.05; Table 2).

##### Efficacy estimand

At week 72, improvements in SF-36 Physical Functioning scores and IWQOL-Lite-CT Physical Function scores were greater in the high-dose combination group than in the remaining groups (Extended Data Table 1).

### Safety

Overall, safety results during the primary treatment period were consistent with known safety profiles of the two drugs. The incidence of treatment-emergent adverse events during the primary treatment period was similar among active drug treatment groups (91.1−98.2%) and greater than placebo (74.5%) (Table 3). Serious adverse events were reported in 8.8−12.5% of participants (bimagrumab), in 1.8−10.7% of participants (semaglutide) and in 3.6−9.1% of participants (combination) versus in 3.6% of participants (placebo); no deaths were reported. Common adverse events included muscle spasms (commonly, muscle cramps), diarrhea and acne with bimagrumab and nausea, diarrhea, constipation and fatigue with semaglutide, with similar events in the combination groups. All treatment discontinuations due to nausea (*N* = 6) occurred in the combination groups, and treatment discontinuations due to muscle spasms (*N* = 5) occurred in the bimagrumab monotherapy groups (Table 3). Four discontinuations were due to acne: two in the bimagrumab monotherapy groups and two in the combination groups.

**Table 3:** AEs and safety through week 48

AE	Placebo (*N* = 55)	Bimagrumab		Placebo + semaglutide		Bimagrumab + semaglutide			
		10 mg kg^−1^ (*N* = 56)	30 mg kg^−1^ (*N* = 57)	1.0 mg (*N* = 55)	2.4 mg (*N* = 56)	10 mg kg^−1^ + 1.0 mg (*N* = 56)	10 mg kg^−1^ +2.4 mg (*N* = 55)	30 mg kg^−1^ + 1.0 mg (*N* = 56)	30 mg kg^−1^ + 2.4 mg (*N* = 55)
	Number of participants (percent)								
Any AE during treatment	41 (74.5)	52 (92.9)	53 (93.0)	52 (94.5)	51 (91.1)	52 (92.9)	54 (98.2)	54 (96.4)	54 (98.2)
SAE	2 (3.6)	7 (12.5)	5 (8.8)	1 (1.8)	6 (10.7)	4 (7.1)	5 (9.1)	2 (3.6)	4 (7.3)
Death	0	0	0	0	0	0	0	0	0
AEs leading to discontinuation of study treatment	2 (3.6)	12 (21.4)	8 (14.0)	2 (3.6)	5 (8.9)	6 (10.7)	3 (5.5)	7 (12.5)	3 (5.5)
AEs reported as the primary reason for treatment discontinuation (*n* ≥ 2 in all patients)									
Nausea	0	0	0	0	0	1 (1.8)	1 (1.8)	2 (3.6)	2 (3.6)
Muscle spasms	0	3 (5.4)	2 (3.5)	0	0	0	0	0	0
Acne	0	1 (1.8)	1 (1.8)	0	0	1 (1.8)	0	1 (1.8)	0
Diarrhea	0	1 (1.8)	1 (1.8)	0	0	0	0	2 (3.6)	0
Constipation	0	0	0	0	1 (1.8)	0	0	0	1 (1.8)
Pancreatitis	0	1 (1.8)	0	1 (1.8)	0	0	0	0	0
AEs during treatment that occurred in ≥10% of participants in any treatment group									
Muscle spasms	3 (5.5)	26 (46.4)	42 (73.7)	7 (12.7)	5 (8.9)	32 (57.1)	35 (63.6)	35 (62.5)	35 (63.6)
Diarrhea	3 (5.5)	23 (41.1)	28 (49.1)	19 (34.5)	20 (35.7)	24 (42.9)	30 (54.5)	24 (42.9)	27 (49.1)
Nausea	7 (12.7)	14 (25.0)	7 (12.3)	25 (45.5)	26 (46.4)	23 (41.1)	34 (61.8)	21 (37.5)	27 (49.1)
Acne	2 (3.6)	19 (33.9)	25 (43.9)	6 (10.9)	5 (8.9)	24 (42.9)	25 (45.5)	31 (55.4)	29 (52.7)
Upper respiratory tract infection	9 (16.4)	12 (21.4)	11 (19.3)	11 (20.0)	12 (21.4)	8 (14.3)	7 (12.7)	11 (19.6)	11 (20.0)
COVID-19	8 (14.5)	14 (25.0)	11 (19.3)	12 (21.8)	16 (28.6)	10 (17.9)	3 (5.5)	3 (5.4)	9 (16.4)
Headache	6 (10.9)	11 (19.6)	6 (10.5)	10 (18.2)	10 (17.9)	9 (16.1)	12 (21.8)	12 (21.4)	9 (16.4)
Constipation	3 (5.5)	2 (3.6)	2 (3.5)	11 (20.0)	16 (28.6)	8 (14.3)	15 (27.3)	15 (26.8)	9 (16.4)
Fatigue	2 (3.6)	4 (7.1)	4 (7.0)	12 (21.8)	14 (25.0)	5 (8.9)	12 (21.8)	7 (12.5)	12 (21.8)
Blood creatine phosphokinase increased	6 (10.9)	8 (14.3)	7 (12.3)	3 (5.5)	1 (1.8)	6 (10.7)	9 (16.4)	8 (14.3)	7 (12.7)
Decreased appetite	2 (3.6)	4 (7.1)	4 (7.0)	6 (10.9)	7 (12.5)	10 (17.9)	6 (10.9)	8 (14.3)	7 (12.7)
Vomiting	1 (1.8)	4 (7.1)	3 (5.3)	5 (9.1)	9 (16.1)	6 (10.7)	10 (18.2)	6 (10.7)	7 (12.7)
Abdominal pain	3 (5.5)	1 (1.8)	3 (5.3)	3 (5.5)	6 (10.7)	8 (14.3)	8 (14.5)	5 (8.9)	8 (14.5)
Rash	3 (5.5)	7 (12.5)	1 (1.8)	4 (7.3)	2 (3.6)	8 (14.3)	5 (9.1)	5 (8.9)	5 (9.1)
Gastroesophageal reflux disease	1 (1.8)	0 (0.0)	3 (5.3)	5 (9.1)	3 (5.4)	3 (5.4)	8 (14.5)	5 (8.9)	6 (10.9)
Nasopharyngitis	3 (5.5)	6 (10.7)	2 (3.5)	4 (7.3)	5 (8.9)	4 (7.1)	2 (3.6)	4 (7.1)	2 (3.6)
Back pain	2 (3.6)	2 (3.6)	0	2 (3.6)	6 (10.7)	5 (8.9)	5 (9.1)	4 (7.1)	2 (3.6)
Arthralgia	4 (7.3)	1 (1.8)	6 (10.5)	5 (9.1)	3 (5.4)	1 (1.8)	0	4 (7.1)	3 (5.5)
Lipase increased	0	2 (3.6)	3 (5.3)	5 (9.1)	6 (10.7)	1 (1.8)	5 (9.1)	3 (5.4)	2 (3.6)
Dizziness	2 (3.6)	4 (7.1)	2 (3.5)	2 (3.6)	7 (12.5)	1 (1.8)	1 (1.8)	3 (5.4)	4 (7.3)
Dyspepsia	0	0	1 (1.8)	3 (5.5)	6 (10.7)	4 (7.1)	5 (9.1)	3 (5.4)	3 (5.5)
Abdominal pain upper	0	3 (5.4)	2 (3.5)	2 (3.6)	3 (5.4)	2 (3.6)	8 (14.5)	2 (3.6)	3 (5.5)
Abdominal distension	0	0	2 (3.5)	3 (5.5)	9 (16.1)	1 (1.8)	6 (10.9)	2 (3.6)	1 (1.8)
Myalgia	2 (3.6)	6 (10.7)	3 (5.3)	4 (7.3)	2 (3.6)	0	0 (0.0)	7 (12.5)	0
Dysgeusia	0	0	4 (7.0)	1 (1.8)	0	3 (5.4)	2 (3.6)	3 (5.4)	7 (12.7)
AESIs^a^									
Severe gastrointestinal disorders	1 (1.8)	3 (5.4)	2 (3.5)	1 (1.8)	3 (5.4)	0	2 (3.6)	1 (1.8)	0
Severe musculoskeletal and connective tissue disorders	0	0	2 (3.5)	0	0	2 (3.6)	0	0	1 (1.8)
Severe skin and subcutaneous tissue disorders	0	0	0	0	0	0	1 (1.8)	0	0
Pancreatitis^b^	1 (1.8)	1 (1.8)	0	1 (1.8)	0	0	0	0	0
Malignancy^c^	0	1 (1.8)	1 (1.8)	0	2 (3.6)	0	0	0	0

Safety endpoints were analyzed using data from participants who received at least one dose of study treatment. Investigator text for AEs was encoded using MedDRA version 25.1. Only AEs with onset during the 48-week primary treatment period or when AE study day is between 1 and 337 are included. ^a^AESIs using CTCAE grading include all pancreatitis or malignancy AEs of any grade severity and all muscle, skin (excluding rash) or gastrointestinal AEs of at least grade 3 (severe) severity. ^b^During adjudication, the committee identified an additional non-serious AE of epigastric pain and elevated lipase to be pancreatitis (high-dose combination). ^c^All four participants had squamous cell carcinoma or basal cell carcinoma of skin.

AE, adverse event; AESI, adverse event of special interest; CTCAE, Common Terminology Criteria for Adverse Events; SAE, serious adverse event.

#### Adverse events of special interest

##### Primary treatment period (through week 48)

Thirteen participants had severe (grade 3) gastrointestinal-related events, including three with pancreatitis serious adverse events (one each in placebo, bimagrumab 10 mg kg^−1^ and semaglutide 1.0 mg groups). One participant had severe acne and five had severe muscle-related events (muscle spasms and back pain) in bimagrumab-containing groups (Table 3). Four participants reported basal or squamous cell skin carcinoma (all in bimagrumab-only or semaglutide-only groups); no other malignancies were reported (Table 3).

##### Open-label extension treatment period (weeks 48–72)

There were no new safety signals during weeks 48–72 (Extended Data Table 2). Two participants had severe gastrointestinal-related events (bimagrumab 10 mg kg^−1^ plus semaglutide 2.4 mg: abdominal pain; bimagrumab 30 mg kg^−1^ plus semaglutide 1.0 mg: enteritis); one participant had severe muscle-related events (back pain; placebo switched to bimagrumab 30 mg kg^−1^ group); and two participants reported basal cell skin carcinoma (bimagrumab 30 mg kg^−1^ and semaglutide 1.0 mg groups) (Extended Data Table 2).

#### Laboratory parameters

No clinically relevant changes in hematologic or renal parameters were observed. Mean magnesium levels decreased in bimagrumab-containing groups but remained within the normal range across treatment groups. Bimagrumab-containing groups showed mean increases in alkaline phosphatase (ALP) and creatine kinase, transient increases in alanine aminotransferase (ALT) (Extended Data Fig. 5) and aspartate aminotransferase (AST) and decreases in total bilirubin and gamma-glutamyl transferase (GGT). Serum lipase increased transiently with bimagrumab but increased and remained elevated with semaglutide treatment (Extended Data Fig. 5).

#### Blood pressure (efficacy estimand)

The mean decreases in systolic blood pressure (SBP) were not significantly different between treatment groups at week 48 (bimagrumab: −3.1 mmHg to −4.6 mmHg; semaglutide: −7.9 mmHg to −8.6 mmHg; combination: −4.5 mmHg to −10.4 mmHg; placebo: −6.9 mmHg) (Table 2). The mean reduction in diastolic blood pressure (DBP) was greater in the high-dose combination group versus the placebo and semaglutide 2.4 mg groups at week 48 (−6.7 mmHg versus −2.8 mmHg and −3.4 mmHg, respectively; *P* < 0.05) (Table 2).

### Exploratory outcomes

#### Bone mineral density (efficacy estimand)

The LSM percent changes in total body and lumbar spine bone mineral density (BMD) were ≤1.1% in all groups at week 48 (Table 2). The LSM percent decreases in total hip BMD were significantly greater in the semaglutide 2.4 mg (−2.1%) group, the bimagrumab 10 mg kg^−1^ plus semaglutide 1 mg (−2.0%) group and the two combination groups containing bimagrumab 30 mg kg^−1^ (−2.2% to −2.3%) versus placebo (−0.8%) (*P* < 0.05). The LSM percent changes from baseline in femoral neck BMD were not significantly different in the treatment groups versus placebo (Table 2). Changes in these BMD outcomes at week 72 were similar, with greater decreases in total hip and/or femoral neck BMD than in total body or lumbar spine BMD across groups (Extended Data Table 1).

#### Lipids (efficacy estimand)

Total and low-density lipoprotein (LDL) cholesterol levels increased in the first 12 weeks in the bimagrumab-containing groups and then decreased toward baseline in the combination groups containing semaglutide 2.4 mg while remaining above baseline in the bimagrumab-only groups and the combination groups containing semaglutide 1.0 mg (Table 2, Extended Data Table 1 and Extended Data Fig. 6). By contrast, increases in high-density lipoprotein (HDL) cholesterol and decreases in triglyceride levels were similar in the combination and semaglutide-only groups at weeks 48 and 72 (Table 2, Extended Data Table 1 and Extended Data Fig. 6). At week 72, LSM percent changes in LDL cholesterol were 17.6% (bimagrumab 30 mg kg^−1^), −8.9% (semaglutide 2.4 mg) and 0.1% (high-dose combination) (Extended Data Fig. 6). For triglycerides, these were −1.2% (bimagrumab 30 mg kg^−1^), −20.8% (semaglutide 2.4 mg) and −25.3% (high-dose combination) (Extended Data Fig. 6).

#### High-sensitivity C-reactive protein (efficacy estimand)

The LSM percent reductions in high-sensitivity C-reactive protein (hsCRP) at week 48 were −52.9% to −69.0% (bimagrumab), −54.5% to −55.4% (semaglutide) and −71.5% to −83.1% (combination) versus −15.6% (placebo group) (Fig. 2f). At week 72, the LSM percent reductions in hsCRP were −72.0% (bimagrumab 30 mg kg^−1^), −59.3% (semaglutide 2.4 mg) and −84.0% (high-dose combination) (Fig. 3f).

#### Adipokines, fasting insulin and free testosterone (efficacy estimand)

The LSM changes in serum leptin levels at week 48 were −20.5 ng ml^−1^ to −26.6 ng ml^−1^ (bimagrumab), −20.1 ng ml^−1^ to −20.5 ng ml^−1^ (semaglutide) and −24.9 ng ml^−1^ to −31.9 ng ml^−1^ (combination) versus −7.7 ng ml^−1^ (placebo) (Extended Data Fig. 7). The LSM increases in serum adiponectin levels at week 48 were 14.1 µg ml^−1^ to 28.6 µg ml^−1^ (bimagrumab), 6.2 µg ml^−1^ to 7.5 µg ml^−1^ (semaglutide) and 22.0 µg ml^−1^ to 33.3 µg ml^−1^ (combination) versus 4.8 µg ml^−1^ (placebo) (Extended Data Fig. 7).

The LSM changes in fasting insulin levels at week 48 are presented in Table 2. At week 72, the LSM decreases in fasting insulin were greater in the high-dose combination group (−34.4 pmol l^−1^) versus bimagrumab 30 mg kg^−1^ (−26.3 pmol l^−1^) and semaglutide 2.4 mg (−27.6 pmol l^−1^) groups (Extended Data Table 1). The LSM changes in free testosterone levels are presented in Table 2 (week 48) and Extended Data Table 1 (week 72).

#### Grip strength (efficacy estimand)

The bimagrumab 30 mg kg^−1^ plus semaglutide 1.0 mg group showed the greatest increase in grip strength among treatment groups at week 48 (4.8 kg; *P* < 0.05 versus placebo (1.7 kg)) (Table 2); all remaining groups were similar to placebo. There was a trend for increased grip strength in the bimagrumab monotherapy groups that was not significantly different compared to semaglutide 2.4 mg at week 48. Results at week 72 are presented in Extended Data Table 1.

#### Dietary intake

At week 24, the median change in total calories (kcal d^−1^) in the higher dose groups was −182.0 (bimagrumab 30 mg kg^−1^), −482.5 (semaglutide 2.4 mg) and −487.0 (high-dose combination) versus −238.5 (placebo). At week 48, participants in the semaglutide 2.4 mg and placebo groups had a greater median reduction in total caloric intake than those in the other groups (Table 2). The median increase in protein intake (as % total calories) was highest in the bimagrumab and placebo groups compared to the semaglutide and combination groups (Table 2).

## Discussion

Obesity is a disease of excess adiposity, which can be confirmed by measurement of body fat by methods such as DXA^17^. In the treatment of obesity with caloric restriction (including lifestyle intervention, incretin-based therapies^18,19^ and bariatric surgery^20^), most weight loss is fat mass, with lean mass comprising approximately 25–40% of total weight loss. All three treatment approaches can induce substantial weight reduction in individuals with obesity, but the associated reduction in lean mass may attenuate the metabolic benefits of substantial weight loss and diminish physical function in those with low muscle mass. Obesity management therapies that preserve lean mass would be expected to cause less overall weight loss unless it was accompanied by increased fat mass reduction^7^.

In this phase 2 trial, treatment with the combination of an activin pathway inhibitor (bimagrumab) plus an incretin (semaglutide) in adults with obesity achieved substantial weight loss by augmenting fat mass reduction while preserving lean mass. Although bimagrumab 30 mg kg^−1^ (10.8%) achieved numerically less weight reduction than semaglutide 2.4 mg (15.7%), weight reduction with the high-dose combination (22.1%) was greater than semaglutide 2.4 mg at week 72. Notably, the total body fat mass reduction achieved with bimagrumab 30 mg kg^−1^ (28.5%) was similar to semaglutide 2.4 mg (27.8%) at week 72 and resulted in nearly additive fat mass reduction with the high-dose combination (45.7%) owing to the distinct mechanisms of action of each drug. This reduction in fat mass achieved with the high-dose combination was in the range of results reported for bariatric surgery at similar timepoints^21,22^. Notably, a higher proportion of the weight reduction in each of the combination groups was due to fat mass loss versus in the semaglutide 2.4 mg group.

Treatment with bimagrumab 30 mg kg^−1^ resulted in a small increase above baseline in lean mass and largely preserved lean mass in the combination groups compared to the greater reduction in lean mass observed with semaglutide 2.4 mg at weeks 48 and 72. Similar changes were observed for appendicular lean mass, a proxy measure of skeletal muscle mass^23^. The combination therapy resulted in preservation of lean mass despite achieving a greater reduction in fat mass, including intra-abdominal fat (VAT), supporting the premise that measures of body composition (and waist circumference) can be more informative regarding optimal obesity management than body weight or BMI. DXA measurements also provide information regarding BMD, with observed changes possibly related to reduced mechanical loading with weight loss.

In the present trial, estimated VAT reduction was notable, particularly for bimagrumab-containing groups. This was accompanied by a greater decline in the inflammation biomarker hsCRP in the bimagrumab-containing groups versus the semaglutide groups. The higher adiponectin levels in the bimagrumab-containing groups at week 48 are likely associated with effects of bimagrumab on adipose tissue and may have downstream effects on insulin sensitivity and inflammation. These outcomes could reflect a positive impact on inflammatory mechanisms underpinning many obesity complications and related diseases, including cardiovascular and metabolic diseases^24^.

The lowering of HbA1c was greater in the semaglutide groups compared to the bimagrumab groups, with similar effects on fasting insulin. However, HbA1c lowering was similar or greater in the combination groups compared to semaglutide alone, suggesting an additive effect on glycemic control. Among participants with prediabetes (HbA1c ≥ 5.7%) at baseline, 100% reversion to normoglycemia was achieved only in the combination groups (except for the low-dose combination) by week 48.

Elevated total and LDL cholesterol levels observed in bimagrumab-containing groups returned toward baseline in the combination groups containing semaglutide 2.4 mg but remained elevated in the bimagrumab-only groups by week 72. Total and HDL cholesterol (and derived non-HDL cholesterol) normalized in the high-dose combination group by week 48. HDL cholesterol and triglycerides improved relative to baseline in the combination and semaglutide-only groups by week 72; HDL cholesterol also improved in the bimagrumab 30 mg kg^−1^ group. The magnitude of lipid changes with bimagrumab may be explained in part by effects of high intravenous doses used in this study, with likely direct effects on lipid metabolism in adipose tissue and/or liver. Future analyses will assess the mechanism and durability of effects of treatment, including post-drug withdrawal, on insulin resistance, lipid metabolism and systemic inflammation as mechanistic determinants of potential cardiovascular benefits or risks.

Adverse events related to bimagrumab and/or semaglutide were infrequent reasons for treatment discontinuation in combination groups. Muscle spasms (for example, muscle cramps) were the primary reason for discontinuation for five participants in the bimagrumab monotherapy groups but none in the combination groups. Muscle spasms have also been reported with blockade of the muscle ligands myostatin and activin A together and activin A alone^25^. The incidence of pancreatitis was infrequent and balanced across treatment groups. There were no instances of telangiectasias or hematologic abnormalities, as reported with activin receptor ligand traps^26^. Additional research is needed to understand the mechanisms underlying specific adverse events such as muscle spasms and acne.

Strengths of this trial include a full factorial study design with two dose levels of each drug (including semaglutide 2.4 mg as approved for obesity); serial direct measurements of body composition using DXA in all participants; treatment extension period to week 72, allowing evaluation of continued weight and fat mass reduction and comparisons with published studies; and the post-treatment follow-up period (ongoing) evaluating weight loss maintenance and composition of weight regain.

Limitations include the use of open-label semaglutide due to unavailability of matched placebo at trial initiation, potentially leading to early discontinuation of some participants who may have been disappointed not to receive semaglutide. Administering bimagrumab intravenously every 12 weeks, with additional loading doses at randomization (week 1) and week 4, may have contributed to early laboratory abnormalities and adverse events; subcutaneous dosing may attenuate these effects, as shown in a comparison study^27^. A phase 2 trial of bimagrumab and tirzepatide, alone or in combination, will evaluate the subcutaneous dosing of both drugs in adults with obesity or overweight (NCT06643728). Unlike previous bimagrumab studies that used MRI for direct assessment of skeletal muscle volume and intramuscular fat^12,28–31^, our study used DXA, where muscle mass was measured as part of lean mass. Future studies should include MRI to better characterize changes in muscle mass and quality along with physical function measures in populations at risk. Additionally, decreased hepatic fat fraction by MRI was reported in a previous bimagrumab study^12^; forthcoming studies could also evaluate changes in visceral and ectopic fat in various regions using MRI. The lack of significant improvement in patient-reported outcomes and grip strength in this study may be due to the broad population studied; specific subpopulations may be more responsive for these measures. Given the sample size for this study, analyses by age and gender would have resulted in subgroups that were too small to support reliable or meaningful conclusions, although these populations should be explored in future studies.

In this phase 2 trial in participants with obesity, treatment with bimagrumab plus semaglutide for 72 weeks resulted in substantial reductions in body weight. The safety findings were consistent with the known safety profiles of the two drugs. These findings support further development of bimagrumab, alone or in combination with incretin therapy, to achieve optimal weight loss, with augmented reduction in adiposity and preserved lean mass, in people living with obesity.

## Methods

### Study design and oversight

BELIEVE (NCT05616013) is a phase 2, multicenter, randomized, double-blind, placebo-controlled trial conducted at 26 sites in the United States, Australia and New Zealand. The trial adhered to the Declaration of Helsinki, Council for International Organizations of Medical Sciences international ethical guidelines and Good Clinical Practice guidelines. An independent ethics committee (New Zealand Southern Health and Disability Ethics Committee, New Zealand; Bellberry Limited Human Research Ethics Committee, Australia; and Austin Health Human Research Ethics Committee, Australia) or institutional review board (WCG Institutional Review Board, United States, and Pennington Biomedical Research Center Institutional Review Board, United States) for each site approved the protocol. All participants provided written informed consent before participation.

**Extended Data Fig. 1:**
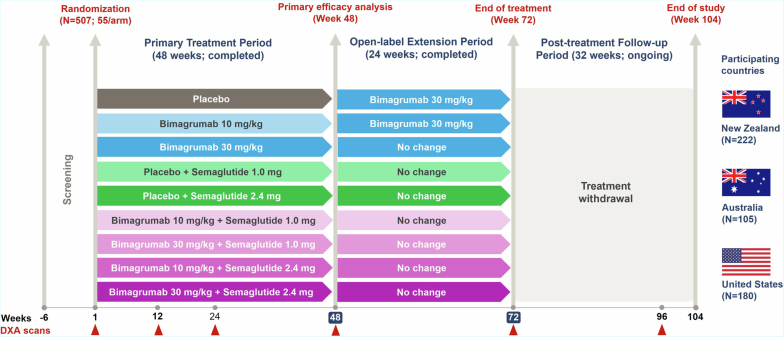
Study design. BELIEVE is a phase 2, multicenter, randomized, double-blind, placebo-controlled trial evaluating the efficacy and safety of intravenous bimagrumab and open-label subcutaneous semaglutide, alone or in combination, in adults with obesity or overweight plus at least one obesity-associated comorbidity. The trial includes a 48-week primary treatment period, blinded to bimagrumab study drug, followed by a 24-week open-label treatment extension period, and a subsequent 32-week follow-up period after treatment withdrawal. Participants were randomly assigned (1:1:1:1:1:1:1:1:1 ratio) to one of the nine treatment groups using a centralized interactive web randomization system. Stratification across the treatment groups was based on gender. After Week 48, participants in treatment groups 1 (placebo) and 2 (bimagrumab 10 mg/kg) switched to bimagrumab 30 mg/kg, while all other groups continued their original treatments without the placebo infusions during the 24-week open-label extension period. During the 32-week post-treatment follow-up period, all study treatments were withdrawn for all groups. Abbreviations: DXA, dual-energy X-ray absorptiometry; N, number of randomized participants.

**Extended Data Fig. 2:**
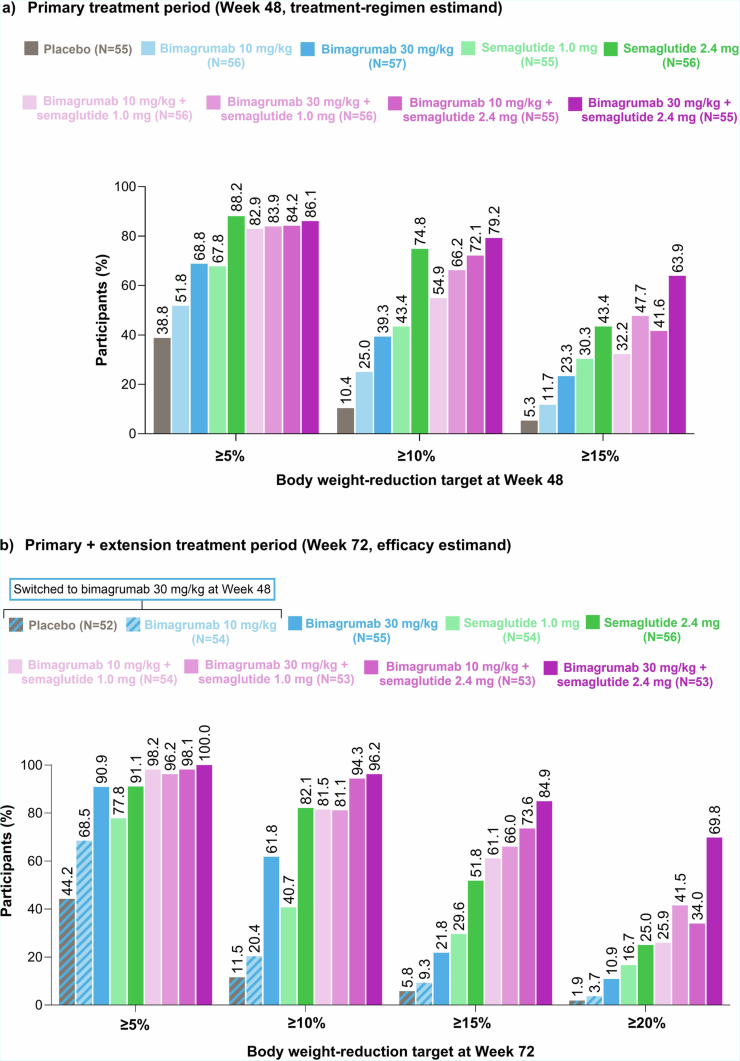
Participants who met weight-reduction thresholds. Panel **a**: The percentage of participants reaching weight-reduction thresholds at Week 48 is based on logistic regression with multiple imputation using a treatment-regimen estimand. N represents number of participants with baseline value. Panel **b**: The percentage of participants reaching weight-reduction thresholds at Week 72 is calculated using logistic regression with mixed model repeated measures for missing data imputation using an efficacy estimand. N represents number of participants based on imputed data.

**Extended Data Fig. 3:**
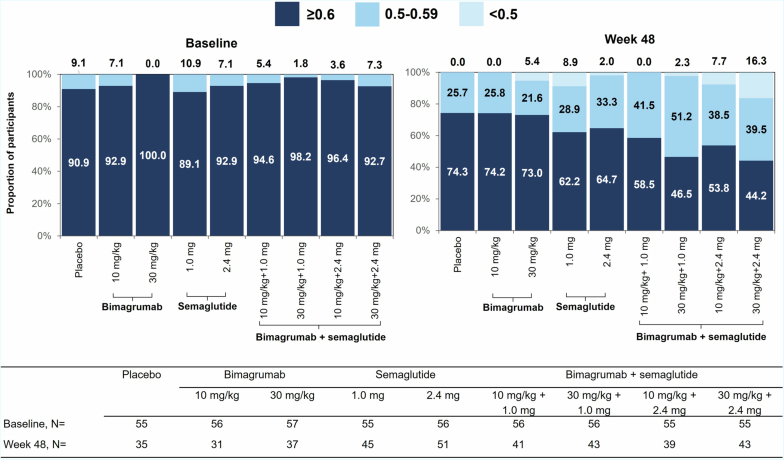
Proportion of participants in waist-to-height ratio categories at Baseline and Week 48 (Primary treatment period; treatment-regimen estimand). N=number of participants with measurement at the timepoint.

**Extended Data Fig. 4:**
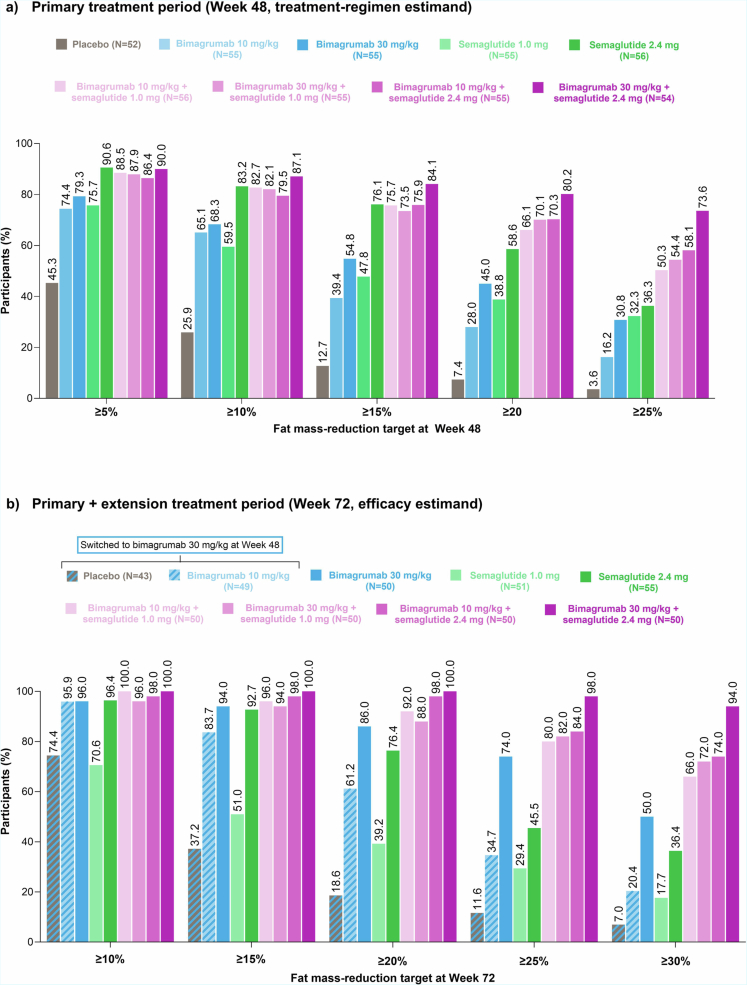
Participants who met fat mass reduction thresholds. Panel **a**: The proportion of participants achieving total body fat mass reduction thresholds at Week 48 by dual-energy X-ray absorptiometry (DXA) is based on logistic regression with multiple imputation using a treatment-regimen estimand. N represents number of participants with baseline value. Panel **b**: The proportion of participants achieving total body fat mass reduction thresholds at Week 72 is calculated based on logistic regression with mixed model repeated measures for missing data imputation using an efficacy estimand. N represents number of participants based on imputed data.

The trial consisted of four sequential periods: a 6-week screening period (ends at randomization), a 48-week blinded primary treatment period, a 24-week open-label treatment extension period (through week 72) and a 32-week treatment-withdrawal follow-up period (end of study at week 104) (Extended Data Fig. 1). We report results from the 48-week primary treatment period and the 24-week extension period (weeks 48−72) (November 2022−November 2024). The protocol and statistical analysis plan are available in the supplementary information.

Versanis Bio Inc., a wholly owned subsidiary of Eli Lilly and Company (sponsor), designed and oversaw the trial conduct. Site investigators collected data, and contract research organizations undertook site monitoring, data collation and data analysis. All authors contributed to data interpretation and authoring and/or critical review of the manuscript. The authors had access to trial data and vouch for the accuracy and completeness of the data and the fidelity of the trial to the protocol. A medical writer employed by the sponsor provided medical writing assistance.

### Participants

The trial included adults (aged ≥18 years and ≤80 years) with obesity: BMI ≥30 kg m^−^^2^ or BMI ≥27 kg m^−^^2^ with at least one obesity-associated comorbidity (for example, hypertension, insulin resistance, sleep apnea or dyslipidemia). All participants maintained a stable body weight (±5 kg) within 90 days of screening, had body weight less than 150 kg and had at least one previous unsuccessful behavioral effort to lose weight. Key exclusion criteria included diagnosis of diabetes requiring current use of an antihyperglycemic drug or HbA1c ≥6.5%. A complete list of the eligibility criteria is provided below.

### Inclusion criteria

Participants were eligible to be included in the study only if all of the following criteria applied:

1. Written informed consent must be obtained before any study-related assessments are performed.
2. Men and women between 18 years and 80 years of age, inclusive; women of childbearing potential (defined as those who are not postmenopausal or postsurgical sterilization) must meet both of the following criteria:

- Two negative pregnancy tests (at screening and at randomization, prior to dosing).
- Use of an intrauterine device, from ≥3 months before the baseline visit through ≥4 months after the last dose of bimagrumab/placebo intravenous, and an additional contraceptive (barrier) method from screening through ≥4 months after the last dose of bimagrumab/placebo intravenous.

3. BMI ≥30 kg m^−^^2^ or BMI ≥27 kg m^−^^2^ with at least one obesity-associated comorbidity (for example, hypertension, insulin resistance, sleep apnea or dyslipidemia).
4. Stable body weight (±5 kg) within 90 days of screening and body weight less than 150 kg.
5. Have a history of at least one self-reported unsuccessful behavioral effort to lose body weight.
6. Capable of using common software applications on a mobile device (smartphone).
7. Access to an internet-enabled smartphone, tablet or computer for the duration of the study, meeting minimal operations systems requirements.
8. Able to communicate well with the investigator, comply with the study requirements and adhere to the diet and activity programs for the study duration.

### Exclusion criteria

Participants were excluded from the study if any of the following criteria applied:

1. History of, or known hypersensitivity to, monoclonal antibody drugs or a contraindication to semaglutide (Ozempic or Wegovy).
2. Use of other investigational drugs at the time of enrollment or within 30 days or five half-lives of enrollment, whichever is longer, or longer if required by local regulations.
3. Lack of peripheral venous access.
4. Not able or willing to comply with protocol requirements, including lifestyle interventions.
5. Women who are pregnant or intend to become pregnant or are nursing.
6. Diseases known to cause cachexia or muscle atrophy or diseases known to cause gastrointestinal malabsorption.
7. Use of any prescription drugs known to adversely affect muscle mass or body weight. Low-dose estrogen replacement therapy in postmenopausal women and 5-α-reductase inhibitors in men are acceptable. Spironolactone and related drugs are acceptable in men and women.
8. Treatment with any medication for the indication of obesity within the past 30 days before screening.
9. Previous or planned (during the trial period) obesity treatment with surgery or a weight loss device. However, the following are allowed: (1) liposuction and/or abdominoplasty, if performed more than 1 year before screening, and (2) lap banding, intragastric balloon or dudodenal-jejunal bypass sleeve, if removed more than 1 year before screening.
10. Uncontrolled thyroid disease at screening or within 6 months prior to screening. Patients with hypothyroidism treated with thyroid hormone replacement therapy must be on a stable dose for at least 6 weeks prior to screening.
11. Diagnosis of diabetes, requiring current use of any antidiabetic drug or HbA1c ≥6.5% Note: Metabolic syndrome is not an exclusion, even if managed with an antidiabetic drug such as metformin or a sodium-glucose co-transporter 2 inhibitor. A diagnosis of prediabetes or impaired glucose tolerance managed exclusively with non-pharmacologic approaches (for example, diet and exercise) is not an exclusion.
12. History of malignancy of any organ system, treated or untreated within the past 5 years, regardless of whether there was evidence of local recurrence or metastases, except non-melanoma skin cancer treated only with local therapy— specifically, multiple endocrine neoplasia type 2 or a personal or family history of medullary thyroid cancer or known elevation of blood calcitonin higher than 50 ng l^−1^.
13. Known heart failure classified as New York Heart Association class III and IV or a history of chronic hypotension (SBP <100 mmHg or DBP <50 mmHg). Uncontrolled hypertension (SBP >180 mmHg or DBP >100 mmHg) at screening/baseline.
14. Electrocardiogram showing clinically significant abnormalities or any history of resuscitated cardiac arrest or presence of an automated internal cardioverter-defibrillator. Prolonged QT syndrome or QTcF > 450 ms (Fridericia correction) for males and QTcF >470 ms for females at screening.
15. History of unstable angina, myocardial infarction, coronary artery bypass graft surgery or percutaneous coronary intervention (such as angioplasty or stent placement) within 180 days of screening.
16. History or presence of significant coagulopathy—for example, prothrombin time/international normalized ratio (PT/INR) >1.5.
17. History of familial hypertriglyceridemia or history of fasting triglyceride higher than 500 mg dl^−1^ (5.65 mmol l^−1^).
18. Known history or presence of severe acute or chronic liver disease (compensated or decompensated), known cholelithiasis or cholecystitis or bile duct disease, acute or chronic pancreatitis (or medication associated with severe pancreatitis, such as valproate) or severe gastrointestinal dysmotility syndrome, including functional disorders such as severe irritable bowel syndrome. Serum lipase >2× upper limit of normal (ULN) or serum amylase >2× ULN at screening.
19. Liver injury as indicated by abnormal liver function tests, such as AST, ALT, GGT, ALP or serum bilirubin:
  Any single transaminase >3× ULN.
  Total bilirubin concentration increased above 1.5× ULN (except for cases of known Gilbert syndrome).
20. History or presence of substantially impaired renal function as indicated by estimated glomerular filtration rate (eGFR) <45 ml min^−1^ 1.73 m^−^^2^ or serum creatinine >1.5× ULN or proteinuria >2+ by urine dipstick or equivalent.
21. Total white blood cells <3,000 per μl, neutrophils <1,500 per μl, hemoglobin <8.5 g dl^−1^ or platelet count <100,000 per μl at screening.
22. Any chronic infections likely to interfere with study conduct or interpretation.
23. Donation or loss of 400 ml or more of blood within 8 weeks prior to initial dosing, or longer if required by local regulations, or plasma donation (>250 ml) within 14 days prior to the first dose.
24. Acute illness within the 30 days prior to screening that, in the opinion of the investigator, affects the patient’s ability to participate in the study.
25. Known or suspected abuse of alcohol or other substances including but not limited to:

- Smoking more than one pack of cigarettes daily.
- Drinking five or more alcoholic beverages on each of five or more days in the past 30 days.
- Using cannabis more than twice weekly.
- Any use of heroin, cocaine, etc.

26. Any disorder, unwillingness or inability not covered by any of the other exclusion criteria, which, in the investigator’s opinion, might jeopardize the participant’s safety or compliance with the protocol.

### Randomization during the double-blind primary treatment period

Participants were randomly assigned (1:1:1:1:1:1:1:1:1 ratio) to one of the following nine treatment groups using a centralized web-based interactive response system: (1) placebo (for bimagrumab); (2) bimagrumab 10 mg kg^−1^; (3) bimagrumab 30 mg kg^−1^; (4) placebo plus semaglutide 1.0 mg; (5) placebo plus semaglutide 2.4 mg; (6) bimagrumab 10 mg kg^−1^ plus semaglutide 1.0 mg; (7) bimagrumab 30 mg kg^−1^ plus semaglutide 1.0 mg; (8) bimagrumab 10 mg kg^−1^ plus semaglutide 2.4 mg; and (9) bimagrumab 30 mg kg^−1^ plus semaglutide 2.4 mg (high-dose combination) (Extended Data Fig. 1). Randomization was stratified by sex across the treatment groups.

### Procedures

Bimagrumab or matching placebo was administered by 30-minute intravenous infusion at the clinical trial sites. Throughout the trial, bimagrumab was dosed based on the previous visit’s body weight. Loading doses for bimagrumab or placebo were administered at randomization (week 1) and week 4, followed by dosing every 12 weeks (weeks 16, 28, 40, 52 and 64). After week 48, with the start of the open-label treatment extension period, group 1 (placebo) and group 2 (bimagrumab 10 mg kg^−1^) switched to receive bimagrumab 30 mg kg^−1^ every 12 weeks without the loading dose. Groups 4 and 5 (semaglutide 1.0 mg and 2.4 mg, respectively) discontinued placebo infusions. The participant, investigator and sponsor were blinded to bimagrumab dose or placebo−bimagrumab until database lock to avoid bias in reporting adverse events and efficacy.

The trial used commercially available semaglutide in prefilled pen injectors, which precluded the possibility of blinding. Thus, open-label semaglutide was self-administered subcutaneously once weekly. For the semaglutide 1-mg-containing groups, semaglutide was initiated at 0.25 mg once weekly for the first 4 weeks, increased to 0.5 mg from weeks 5 to 8 and increased to 1.0 mg from weeks 9 to 71. For the semaglutide 2.4-mg-containing groups, semaglutide was initiated at 0.25 mg once weekly for the first 4 weeks, with the dose increased to 0.5 mg from weeks 5 to 8, to 1.0 mg from weeks 9 to 12, to 1.7 mg from weeks 13 to 16 and to 2.4 mg from weeks 17 to 71. The higher dose of semaglutide (2.4 mg) is the approved dose for the treatment of obesity.

Participants had monthly counseling sessions to follow a diet with a daily deficit of approximately 500 kcal and ≥1.2 g kg^−1^ d^−1^ of protein and to engage in at least 150 minutes of physical activity weekly.

Grip strength was measured using the Jamar Plus Digital Hand Dynamometer, with participants seated and using their dominant hand. Each participant had one practice trial before the recorded official measurement. The investigator and study staff were trained to use the dynamometer, and the same staff member conducted all assessments for a given participant.

### Endpoints and assessments

The primary endpoint was absolute change from baseline in body weight at week 48. The secondary efficacy endpoints included here are as follows: percent change in body weight at week 48; absolute and percent change in body weight at week 72; absolute and percent changes at weeks 48 and 72 in total body fat and lean mass, appendicular lean mass and estimated VAT as assessed by DXA; absolute changes in waist circumference at weeks 48 and 72; proportion of participants who achieved body weight reduction thresholds and fat mass reduction thresholds at weeks 48 and 72; percentage of weight loss due to fat mass or lean mass at weeks 48 and 72; proportion of participants in waist-to-height ratio categories at week 48; and absolute changes in HbA1c and patient-reported outcomes (SF-36 Physical Functioning score and IWQoL-Lite-CT Physical Function score) at weeks 48 and 72.

Safety assessments included treatment-emergent adverse events, serious adverse events and changes in vital signs and laboratory assessments according to the protocol.

Exploratory endpoints included here are total and regional BMD, lipid profile, hsCRP, grip strength, fasting insulin and free testosterone levels at weeks 48 and 72 and adipokines, total caloric intake and protein intake at week 48.

### Statistical analysis

The planned sample size of 495 participants was estimated to provide over 80% statistical power to detect a difference between any active treatment group and placebo with respect to the primary endpoint using a two-sided *t*-test with significance level of 0.05. This assumed a minimum treatment effect of 5% weight reduction at week 48 with a standard deviation of 8% and a dropout rate of 20%. Efficacy endpoints were analyzed using data from all randomized participants. Safety endpoints were analyzed using data from participants who received at least one dose of study treatment.

All statistical tests were performed using a two-sided 5% significance level, with corresponding 95% confidence intervals. For the week 48 analysis, nominal *P* values for pairwise comparisons versus placebo and semaglutide 2.4 mg (the approved dose for obesity) are reported for bimagrumab, semaglutide and combination groups. A preplanned unblinded interim analysis was conducted when approximately 80% of participants completed the week 24 visit or prematurely discontinued the study treatment. An independent team evaluated the efficacy and safety profile of monotherapy and combination groups during this interim analysis for internal decision-making. An external data monitoring committee periodically reviewed safety data from the study. All week 48 analyses were prespecified in the statistical analysis plan, and all week 72 analyses were considered post hoc. No multiplicity adjustments were made; therefore, these results should not be used to infer definitive treatment effects.

Two estimands (treatment regimen estimand and efficacy estimand), based on the ICH E9(R1) guidance^32^, were used to assess treatment efficacy from different perspectives and accounted for intercurrent events differently. Both estimands were used for the primary treatment period analysis for primary and secondary endpoints, unless specified otherwise. The efficacy estimand was used for the exploratory endpoints analysis. For the open-label treatment extension period analysis, only the efficacy estimand was used due to the lack of a true placebo group.

#### Treatment regimen estimand

This estimand is used to assess the average treatment effect of bimagrumab, semaglutide or bimagrumab plus semaglutide for all randomized participants at week 48, regardless of treatment adherence and/or premature discontinuation of study treatment or placebo. For the analyses of this estimand, missing values (unobserved due to patient loss to follow-up or other reasons) were assumed to be missing at random and were handled by multiple imputation using observed data in the placebo group. Due to insufficient retrieved dropout data (that is, data from participants who discontinued treatment but remained in the study), a control-based imputation approach using the placebo group was selected as a more conservative strategy. Continuous endpoints were analyzed using the analysis of covariance (ANCOVA) model, and categorical endpoints were analyzed by logistic regression. Both models included treatment group, gender and country as fixed effects and baseline value as covariate. The analyses were conducted with multiple imputation of missing values at week 48 and statistical inference over multiple imputation of missing data guided by Rubin^33^. Categorical outcomes were derived from imputed continuous outcomes.

#### Efficacy estimand

This estimand is used to assess the average treatment effect of bimagrumab, semaglutide or bimagrumab plus semaglutide for all randomized participants at weeks 48 and 72 had they received at least one dose of study treatment, adhered to protocol-defined treatment and did not discontinue treatment prematurely. Data after intercurrent events (for example, permanent treatment discontinuation) were excluded from analysis. Continuous endpoints were analyzed using a mixed model for repeated measures (MMRM), and missing values were implicitly handled by MMRM under the assumption of missing at random. No additional imputation was performed. The MMRM includes treatment group, gender, country, visit and visit-by-treatment as fixed effects and baseline value as covariate. A logistic regression model with treatment group, gender and country as fixed effects and baseline value as covariate was used for categorical outcomes.

### Reporting summary

Further information on research design is available in the Nature Portfolio Reporting Summarylinked to this article.

## Online content

Any methods, additional references, Nature Portfolio reporting summaries, source data, extended data, supplementary information, acknowledgements, peer review information; details of author contributions and competing interests; and statements of data and code availability are available at 10.1038/s41591-026-04204-0.

## Supplementary information

Supplementary Information Investigator list, protocol and statistical analysis plan

Reporting Summary

## Source data

Source Data Figs. 2 and 3 Source data (*n*, LSM, s.e., *P* values) included in the submission.

**Extended Data Fig. 5:**
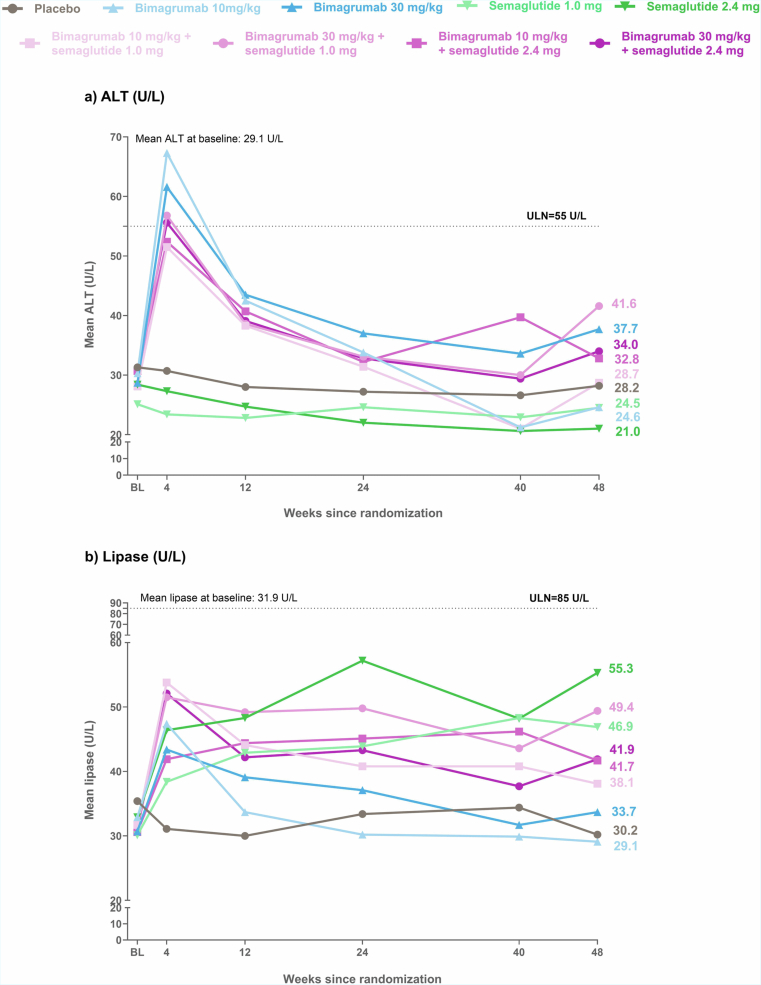
Arithmetic mean serum enzymes values at Week 48. Panel **a**: Data are presented as arithmetic means for ALT. Panel **b**: Data are presented as arithmetic means for lipase. n values are provided in the source data tables in the supplementary. Abbreviations: ALT, alanine aminotransferase; BL, baseline; ULN, upper limit of normal. Source data

**Extended Data Fig. 6:**
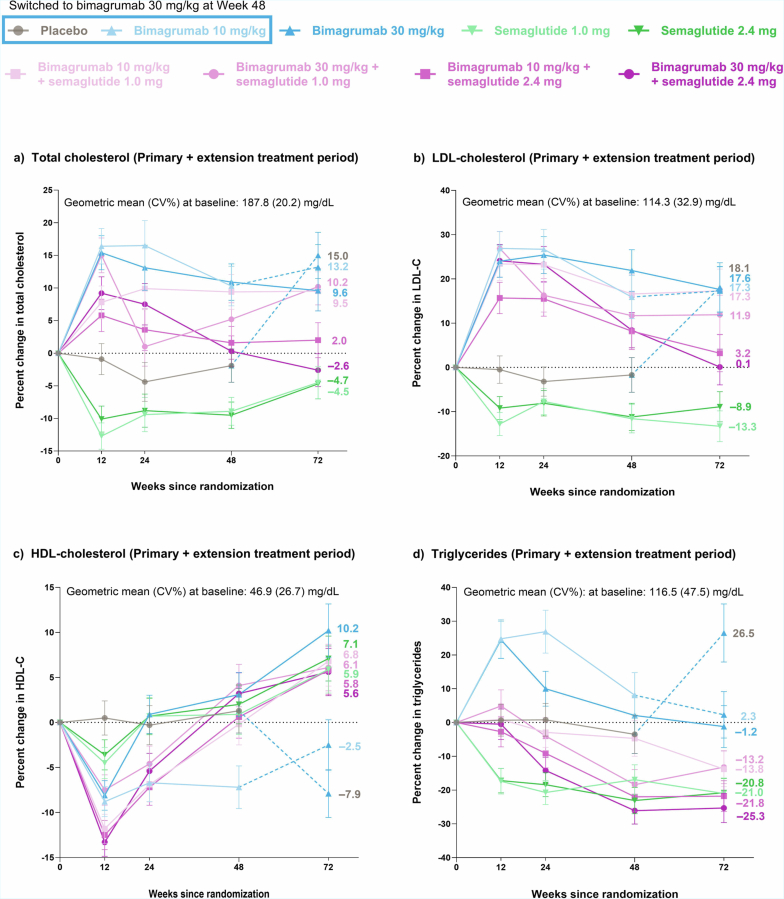
Percent change from baseline to Week 72 in serum lipids. Data are presented as LSM percent change from baseline±SE. The primary treatment period starts at the first dose of Week 1 and ends at the last visit on or prior to Week 48. The open-label extension treatment period starts at the first dose after Week 48 and ends at the last visit on or prior to Week 72. The placebo and bimagrumab 10 mg/kg groups switched to bimagrumab 30 mg/kg for the open-label extension period. n values are provided in the source data tables in the supplementary. Panels **a**-**d**: The LSM percent changes in lipids from baseline to Week 72 are based on a MMRM using log-transformation for the efficacy estimand. Abbreviations: CV, coefficient of variation; HDL, high-density lipoprotein; LDL, low-density lipoprotein; MMRM, mixed model for repeated measures; SE, standard error. Source data

**Extended Data Fig. 7:**
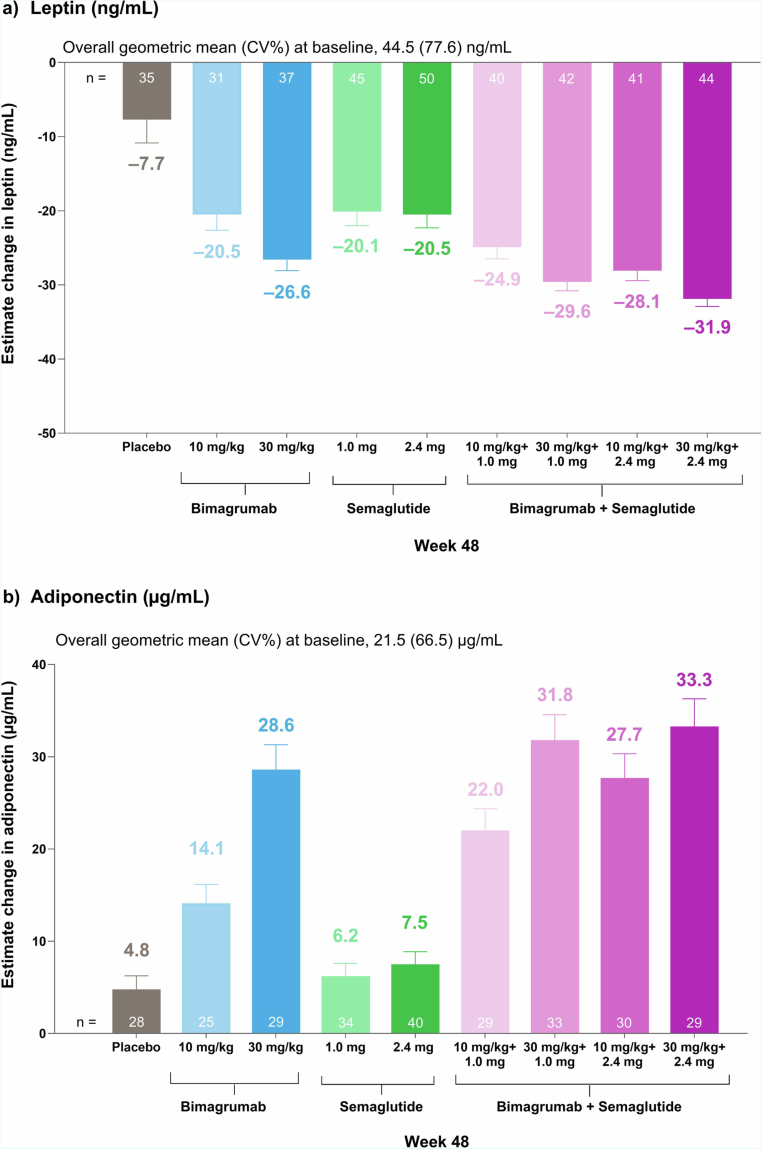
Change from baseline in adipokines at Week 48. Data are presented as LSM change from baseline±SE. n represents number of participants with baseline and post-baseline value at Week 48. Panels **a**-**b**: LSM changes from baseline at Week 48 in leptin and adiponectin are based on a MMRM using log-transformation for the efficacy estimand. Abbreviations: CV, coefficient of variation; MMRM, mixed model for repeated measures; SE, standard error. Source data

Source Data Extended Data Figs. 5, 6 and 7 Source data (*n*, arithmetic mean) for Extended Data Fig. 5; Source data (*n*, LSM, s.e.) for Extended Data Fig. 6; Source data (*n*, LSM, s.e.) for Extended Data Fig. 7.

## Figures and Tables

**Extended Data Table 1:**
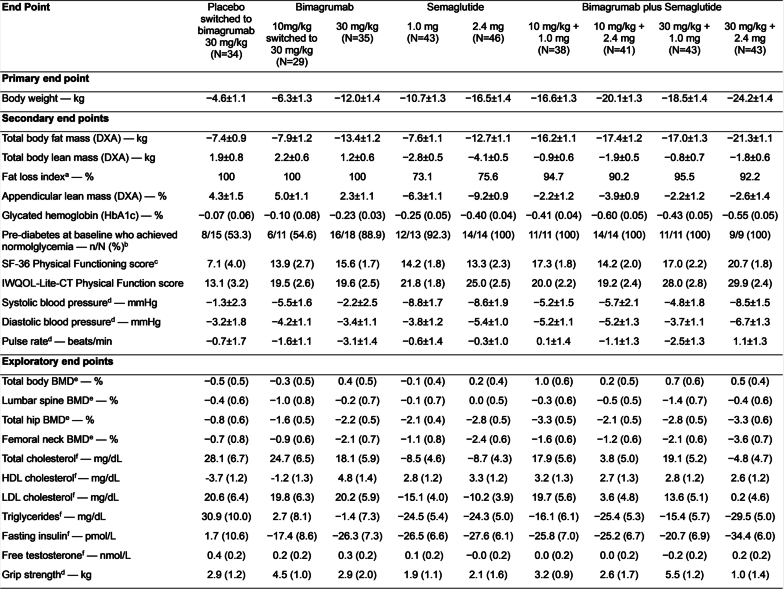
Changes from baseline in efficacy end points at Week 72

Data are presented as LSM change from baseline ± SE, unless specified otherwise. All analyses were conducted in the intention-to-treat population. Baseline was defined as the last non-missing assessment recorded on or prior to the date of the first study drug injection. The primary treatment period starts at the first dose of Week 1 and ends at the last visit on or prior to Week 48. The open-label extension treatment period starts at the first dose after Week 48 and ends at the last visit on or prior to Week 72. The placebo and bimagrumab 10 mg/kg groups switched to bimagrumab 30 mg/kg for the open-label extension period. The changes from baseline in primary and secondary endpoints at Week 72 are based on MMRM for the efficacy estimand. The variance-covariance structure for changes from baseline was unstructured.

^a^Fat loss index is defined as change in total body fat mass (measured using DXA) in kg, divided by sum of change in total body fat mass and change in total body lean mass in kg times 100 (with maximum value = 100).

^b^The proportion of participants achieving normoglycemia (glycated hemoglobin <5.7%) among those with baseline glycated hemoglobin ≥5.7% is based on logistic regression with MMRM used for missing data imputation at Week 72 for the efficacy estimand. N = number of participants in imputed data; n = number of participants achieving target in imputed data.

^c^Transformed scores standardized to range from 0 to 100.

^d^The LSM changes from baseline at Week 72 in systolic blood pressure, diastolic blood pressure, pulse rate, and grip strength are based on MMRM for the efficacy estimand.

^e^The LSM percent changes from baseline at Week 72 in total body, lumbar spine, total hip, and femoral neck BMD are based on MMRM for the efficacy estimand.

^f^The LSM changes from baseline at Week 72 in lipids, fasting insulin, and free testosterone are based on MMRM using log-transformation for the efficacy estimand.

Abbreviations: HDL, high-density lipoprotein; IWQOL-Lite-CT, Impact of Weight on Quality of Life-Lite Clinical Trials Version; LDL, low-density lipoprotein; LSM, least-squares mean; MMRM, mixed model for repeated measures; SE, standard error; SF-36, 36-Item Short Form Health Survey; VAT, visceral adipose tissue.

**Extended Data Table 2:**
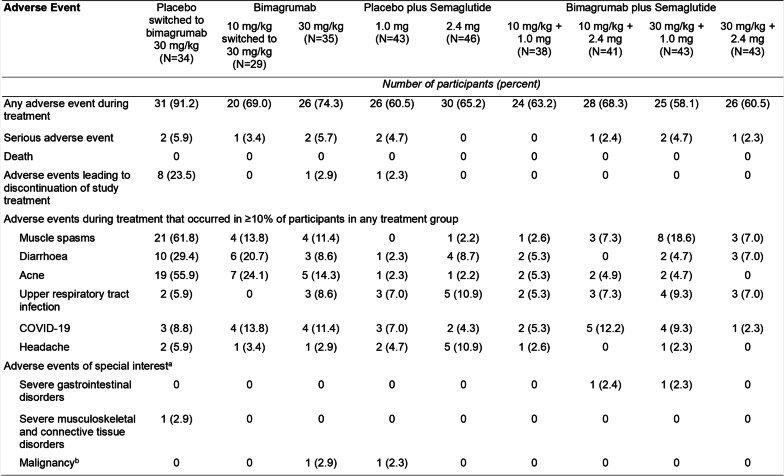
Adverse events and safety from Weeks 48 to 72

Safety endpoints were analyzed using data from participants who received ≥1 dose of study treatment. Investigator text for AEs encoded using MedDRA Version 27.1. Only AEs with onset during the open-label extension period (Weeks 48 to 72) are included.

^a^AESIs using CTCAE grading include all pancreatitis or malignancy AEs of any grade severity and all muscle, skin (excluding rash) or gastrointestinal AEs of at least grade 3 (severe) severity.

^b^Both participants had basal cell carcinoma of skin.

Abbreviations: AE, adverse event; AESI, Adverse Event of Special Interest; CTCAE, Common Terminology Criteria for Adverse Events.

## Data Availability

Eli Lilly and Company provides access to all individual participant data collected during the trial, after anonymization, with the exception of pharmacokinetic or genetic data. Data are available upon reasonable request 6 months after the indication studied has been approved in the United States and the European Union and after primary publication acceptance, whichever is later. No expiration date of data requests is currently set once data are made available. Access is provided after a proposal has been approved by an independent review committee identified for this purpose and after receipt of a signed data-sharing agreement. Data and documents, including the trial protocol, statistical analysis plan, clinical study report and blank or annotated case report forms, will be provided in a secure data-sharing environment. For details on submitting a request, see the instructions provided at https://vivli.org/. Source data are provided with this paper.
