# Supplementary material for: Bimagrumab plus semaglutide alone or in combination for the treatment of obesity: a randomized phase 2 trial
Source: Nat Med. 2026 Mar 2;32(3):869–82. doi: 10.1038/s41591-026-04204-0 (PMC13004672; doi:10.1038/s41591-026-04204-0)
Supplement: Supplementary file 1 — Investigator list, protocol and statistical analysis plan [file 41591_2026_4204_MOESM1_ESM.pdf]

nature medicine

Supplementary information https://doi.org/10.1038/s41591-026-04204-0

Bimagrumab plus semaglutide alone or in combination for the treatment of obesity: a randomized phase 2 trial

In the format provided by the authors and unedited

Table of Contents

List of the BELIEVE Phase 2 Obesity Trial Investigator....................2
Study protocol....................4
Statistical analysis plan....................117

List of the BELIEVE Phase 2 Obesity Trial Investigators (Study J4Z-MC-GIDA; NCT05616013)

	Investigator name	Site address
1.	Dr. Louis Aronne	Weill Cornell Medicine, 1305 York Avenue, New York, NY 10021, United States
2.	Dr. Steven Heymsfield	Pennington Biomedical Research Center, 6400 Perkins Rd, Baton Rouge, LA 70808, United States
3.	Dr. Damon Daniels	SPICA Clinical Research, 110 Atrium Way, Columbia, SC 29223, United States
4.	Dr. John Mark Joyce	Clinical Neuroscience Solutions (CNS) Healthcare, 5200 Belfort Rd, Jacksonville, FL 32256, United States
5.	Dr. Awawu Igbinadolor	Monroe Biomedical Research, 343 Venus St, Monroe, NC 28112, United States
6.	Dr. Almena Free	Pinnacle Research Group, 409 East 10th St, Suite 200, Anniston, AL 36207, United States
7.	Dr. Randall Quinn	Cullman Clinical Trials, 111 4th Ave NE, Cullman, AL 35055, United States
8.	Dr. Samuel Lederman	Altus Clinical Research, 4671 South Congress Ave, Suite 100-B, Lake Worth, FL 33461, United States
9.	Dr. Jose Cardona	Indago Research & Health Center, 3700 W 12th Ave Suite 300, Hialeah, FL 33012, United States
10.	Dr. Harish Thakkar	Mt. Olympus Medical Research, 15200 Southwest Fwy, Suite 150, Sugar Land TX 77478, United States
11.	Dr King Cheung	Emeritus Research Melbourne, Ground Floor, 1096 Toorak Road, Camberwell, 3124 Victoria, Australia
12.	Dr Stephanie Wallace	University of the Sunshine Coast - Sippy Downs, Level 1, 9 Ochre Way Sippy Downs, 4556 Queensland, Australia
13.	Dr Indika Leelasena	University of the Sunshine Coast – Morayfield, Morayfield Health Hub, Level 1, 19-31 Dickson Road, Morayfield 4506 Queensland, Australia
14.	Dr Nischal Sahai	University of the Sunshine Coast - South Brisbane, Southbank Building A1 SW Complex, 52 Merivale Street, SouthBrisbane 4101 Queensland, Australia
15.	Dr Russell Canavan	Gold Coast Hospital, 1 Hospital Boulevard, Southport, 4215 Queensland, Australia
16.	Dr Elif Ekinci	Austin Health, Hiedelberg Repatriation Hospital, 300 Waterdale Rd, Heidelberg Heights 3081 Victoria, Australia
17.	A/Prof Sarah Glastras	Royal North Shore Hospital (Northern Sydney Local Health District), NSLHD Executive Unit, Level 14 Kolling Building, Royal North Shore Hospital, Reserve Rd, St Leonards, 2065 New South Wales, Australia

18.	Dr Divyansh Joshi	Northern Beaches Clinical Research, 201/694-696 Pittwater Rd, Brookvale, 2100 New South Wales, Australia
19.	Dr Tina Baik	Optimal Clinical Trials, Level 2, 97 Grafton Road, Grafton 1010 Auckland, New Zealand
20.	Dr Penelope Montgomery	Optimal Clinical Trials, Level 2, 97 Grafton Road, Grafton 1010 Auckland, New Zealand
21.	Dean Quinn	P3 Research Limited, P3 Research Wellington Unit, 1st floor 121 Adelaide road, Mount Cook, Wellington, New Zealand.
22.	Claire Thurlow	PCRN Clinicals Trials Ltd, Trading as PCRN Clinical Trials Tasman, 469 Main Road Stoke, Nelson, 7011, New Zealand
23.	John Baker	Aotearoa Clinical Trials Trust operating as Aotearoa Clinical Trials (ACTT), Middlemore Hospital, 100 Hospital Road, Papatoetoe, Auckland 2025, New Zealand
24.	Laura Elliot	New Zealand Clinical Research OPCO Ltd AKL, 3 Ferncrort Street Grafton, 1010 Auckland, New Zealand
25.	Jane Kerr	NZCR OPCO LTD CHC, Level 3 / 264 Antigua Street, Christchurch, 8011, New Zealand
26.	Tiwini Hemi	Avalon Clinical Trials Ltd, Trading as Lakeland Clinical Trials Waikato, 6 Avalon Drive, Nawton, Hamilton, 3200, New Zealand
27.	Dr Joanne Finlay	PCRN Clinical Trials Ltd, Trading as Pacific Clinical Research Network Christchurch, Forte 2, Level 2, 132 Peterborough Street, Christchurch 8013, New Zealand

CLINICAL STUDY PROTOCOL

Bimagrumab

VER201-PH2-031

A randomized, double-blind, placebo-controlled multi-center study of intravenous bimagrumab, alone or in addition to open label subcutaneous semaglutide, to investigate the efficacy and safety in overweight or obese men and women

IND Number:	161031
EUDRA CT number:	Not applicable
Study phase	2
Version number:	5.0 (Amendment 5)
Sponsor:	Versanis Bio, Inc.<br>A wholly owned subsidiary of Eli Lilly and Co.<br>1111 Broadway, Suite 1300<br>Oakland, CA 94607<br>United States
Approval Date:	Protocol Electronically Signed and Approved by Lilly on date provided below.
Document ID:	VV-CLIN-150100

Confidentiality Statement

This document is the sole property of Versanis Bio, Inc. This document and all information contained herein must be considered and treated as strictly confidential. This document shall be used only for the purpose of the disclosure herein provided. No disclosure or publication shall be made without the prior written consent of Versanis Bio, Inc.

SPONSOR SIGNATURE PAGE

The protocol has been approved by Versanis Bio, Inc.

Sponsor's Authorized Officer:

______________________________	______________________________
PPD	Date

INVESTIGATOR'S AGREEMENT

I agree, as an Investigator conducting this study:

- To assume responsibility for the proper conduct of the study at this site.
- To conduct the study in compliance with this protocol, with any future amendments, and with any other written study conduct procedures provided, reviewed, and approved by the Sponsor.
- Not to implement any deviations from or changes to this protocol without agreement from the Sponsor, except where necessary to eliminate an immediate hazard to the participants or for administrative aspects of the study (where permitted by all applicable regulatory requirements).
- That I am aware of, and will comply with, Good Clinical Practice and all applicable regulatory requirements.
- To ensure that all persons assisting me with the study are adequately informed about the investigational drug and that they are qualified to perform their study-related duties and functions, as described in this protocol.
- That I have been informed that certain regulatory authorities require the Sponsor to obtain and supply details about the Qualified Investigator's ownership interest in the Sponsor or the study drug and more generally about his/her financial ties with the Sponsor. The Sponsor will obtain and disclose any relevant information in this regard solely for the purpose of complying with regulatory requirements.

Hence, I

- Agree to supply the Sponsor with all information regarding ownership interest and financial ties with the Sponsor (including those of my spouse and dependent children);
- Agree to promptly update this information if any relevant changes occur during the study and for 1 year following completion of the study; and
- Agree that the Sponsor may disclose this information about such ownership interests and financial ties to regulatory authorities.

______________________	______________	____________
Printed Name of Investigator	Signature of Investigator	Date

Site Name ____

Site Address ____

MEDICAL MONITOR NAME AND CONTACT INFORMATION

Role in Study	Name	Email and Telephone Number

PPD

DOCUMENT HISTORY

DOCUMENT HISTORY	
**Document**	**Date**
Original (Ver 00)	14 June 2022
Amendment 1 (Ver 1.0)	26 September 2022
Amendment 1 (Ver 1.1)	10 October 2022
Amendment 2 (Ver 2.0)	19 December 2022
Amendment 3 (Ver 3.0)	08 May 2023
Amendment 4 (Ver 4.0)	24 September 2023

SUMMARY OF CHANGES – FOR AMENDMENT 5, PROTOCOL V. 5.0

The amendment is considered to be substantial because it is likely to have a significant impact on the

- safety or the rights of the study participants, and
- reliability and robustness of the data generated in the clinical study.

Overall Rationale for the Amendment:

The secondary endpoint has been adjusted to accommodate assessment at Week 72, and new exploratory objectives/endpoints have been added to align with the study design. This amendment also updates the pre-dose safety lab review at Week 64 to monitor safety.

Additionally, this amendment provides dosing guidance for management of participants with BMI ≤22 kg/m^2 and those with perceived excessive body weight loss.

Section # and Name	Description of Change	Brief Rationale
Section 1.1. Synopsis	Revised the brief title to read as:<br>"Safety and efficacy of bimagrumab and semaglutide in adults ~~who~~ with ~~are~~ overweight or ~~obese~~ obesity"	Prioritizing person-first language and avoiding stigmatizing adjectives to describe individuals living with obesity
Section 1.3. Schedule of Activities (SoA)	Randomization and drug administration row:<br>• Referenced Footnote "f" for Week 64<br>• Updated Footnote "f" to incorporate pre-dose safety lab review at Week 64	To observe and react to potential enzyme elevations prior to the second dose of bimagrumab in Year 2 at Week 64
Section 1.1. Synopsis<br>Section 3. Objectives, Endpoints, and Estimands	• Secondary endpoint:<br>Added Week 72 to assess treatment effect on waist circumference, fat mass, VAT, trunk fat mass, SAT, lean mass, self-reported health status and weight-related quality of life<br>• Secondary objective:<br>Added "body composition"	Alignment with the study design to describe Year 2 endpoints
Section 3. Objectives, Endpoints, and Estimands	Exploratory objective:<br>Added new objective to assess the treatment withdrawal effect of bimagrumab, semaglutide, and bimagrumab in addition to	

Section # and Name	Description of Change	Brief Rationale
	semaglutide on body weight, waist circumference, and body composition	
Section 3.1. Estimands	Added information that trial product or hypothetical estimand will be used to assess the primary estimand	For consistency with SAP
Section 4.1. Overall Design	Core Treatment Period (Weeks 1 to 48):<br>• Replaced "participants ~~will be required~~" to "participants should" follow recommendations for the duration of the core treatment period<br>• Added statement where occasional diary misses are permissible, provided there is no persistent non-compliance<br><br>Open-Label Extension Treatment Period (Weeks 49 to 72):<br>• Replaced "participants ~~must~~" to "participants should" continue to follow dietary and exercise recommendations for the duration of the extension treatment period<br>• Added that participants must self-administer weekly semaglutide as in the core treatment period<br><br>Post-Treatment Follow-Up Period (Weeks 73 to 104): Replaced "participants ~~must~~" to "participants should" continue to follow dietary and exercise recommendations	Clarification
Section 5.31. Meals and Dietary Restrictions	Indicated that participants must fast for at least 8 hours prior to	

Section # and Name	Description of Change	Brief Rationale
	all visits that include blood sampling	
Section 5.3.4. Support During Treatment Withdrawal Period	Newly added section:<br>Added paragraph on accessibility to personnel and additional resources to aid in lifestyle management during the treatment withdrawal period	To clarify Sponsor's intentions regarding provision of these additional healthy lifestyle resources during the treatment withdrawal period to promote participant retention
Section 6.6.2. Semaglutide	Updated section with management of participants with BMI $\leq$22 kg/m^2 and those with perceived excessive body weight loss	To be consistent with guidance for site participants who are losing excessive weight
Section 7.1.1. Liver, Pancreas, or Muscle Related Events Stopping Criteria	Added a new timepoint "Week 64" where the participant's safety labs will be reviewed at Week 64	Updated to align with the changes made in Section 1.3 on reviewing safety labs at Week 64
Section 7.1.2. Temporary Discontinuation	Updated the section to reference Section 6.6.2 for management of participants if they cannot tolerate current dose of semaglutide	Updated to align with the changes made in Section 6.6.2
Section 8. Study Assessments and Procedures	Added "Visit 19 (Week 64)" as an exception to perform dosing within the corresponding window	To observe and react to potential enzyme elevations prior to the second dose of bimagrumab at Week 64
Section 9.4. Treatment Extension and Post-Treatment Follow-Up Period Analyses	• Updated section to include analyses and summaries of treatment effect at Week 72<br>• Deleted language on treatment outcome comparisons	To align with the changes made in Section 3 to describe year 2 endpoints
Section 10.3.2. Definition of SAE	Other situations bullet:<br>Added language that "All malignancies must be reported as SAEs"	For clarity
Section 10.8. Appendix 8: Creatine Kinase Safety	Edits made to Table 11	

SUMMARY OF CHANGES – FOR AMENDMENT 4, PROTOCOL V. 4.0

Section # and Name	Description of Change	Brief Rationale
Medical Monitor Name and Contact Information	Replaced Drug Safety Physician contact information.	Eli Lilly Global Patient Safety has replaced Parexel for Drug Safety Physician services.
Section 1.1 – Synopsis<br>Section 1.2 – Study Schema<br>Section 4.1 – Overall Design	The extension treatment period (Weeks 48-72) changed from blinded to open label	The study will be open label after Week 48 primary efficacy analysis, and unblinded after Week 48 database lock. Placebo injections will continue for Arms 2 and 3 until after the Week 48 database lock and study unblinding.
Section 1.1 – Synopsis<br>Section 3.1.1 – Estimands of Secondary Objectives	Added appendicular lean mass by DXA as secondary endpoints	Lean mass of the arms and legs has a higher proportion of skeletal muscle than total body lean mass.
Section 1.1 – Synopsis<br>Section 3.1.1 – Estimands of Secondary Objectives<br>Section 9.1 – Statistical Analyses	Removed language referring to adjustment for multiplicity.	Adjustment for multiplicity of primary or secondary endpoint analyses are no longer considered necessary by Sponsor, as study results will be used primarily for further development planning purposes.
Section 1.1 – Synopsis	Allowed for the addition of two non-voting members from the Sponsor (who are not part of the study team) to the DMC.	The DMC has requested additional support from the Sponsor by adding two non-voting members with safety and statistical expertise.
Section 1.3 – Schedule of Activities (SoA)	Removed Week 104 blood draws for PD biomarkers and anti-drug antibodies.	Follow up blood collection at Week 96 is sufficient for these parameters.
Section 3 – Objectives, Endpoints, and Estimands<br>Section 8.2.6.2 – Glucose Metabolism Parameters: HbA1c, Fasting Insulin and Glucose	Replaced HOMA2-IR with HOMA-IR .	HOMA2-IR is proprietary and unavailable; HOMR-IR is a suitable alternative.
Section 5.3.1 – Meals and Dietary Restrictions	Guidance provided for cases where participant reaches a BMI $\leq$ 22 kg/m^2.	Guidance provided for safety of participants losing excessive weight.

Section # and Name	Description of Change	Brief Rationale
Section 9.3 – Interim Analysis	Added interim analysis.	A 24-week interim analysis was added for internal decision-making purposes
Section 10.7 – APPENDIX 7: Lipase and Amylase Safety	Allowed for the addition of an adjudication committee for acute pancreatitis and other major adverse events.	The DMC has requested additional support for evaluating suspected cases of pancreatitis.

Additional changes include grammatical corrections and clarifying text.

SUMMARY OF CHANGES – FOR AMENDMENT 3, PROTOCOL V. 3.0

Section # and Name	Description of Change	Brief Rationale
Section 1.1 – Synopsis<br>Table 1 – Table of Objectives and Endpoints<br>Section 9.1 Statistical Analyses	Rephrased primary endpoint, secondary objectives, and secondary endpoints to measure a 'change', rather than specify an 'absolute' or 'percent' change.	Allow the Statistical Analysis Plan to be the source of detail for all planned analyses.
Section 1.1 – Synopsis<br>Table 1 – Table of Objectives and Endpoints<br>Section 8.2.2 – DXA Scan	Added assessment of treatment effects on subcutaneous adipose tissue to secondary objectives and secondary endpoints. Also included an analysis of decrease in body weight >15%.	More completely characterize treatment effects.
Section 1.1 – Synopsis<br>Section 1.2 – Study Schema<br>Section 1.3 – Schedule of Activities<br>Section 4.1 – Overall Design<br>Section 4.2 – Scientific Rationale for Study Design<br>Section 4.3.1 – Bimagrumab Doses<br>Section 6.1 – Study Intervention Administered<br>Section 6.7 – Continued Access to Study Intervention after the End of Treatment<br>Section 7.1 – Participant Discontinuation of Study Intervention<br>Section 7.2 – Participant Discontinuation/Withdrawal from the Study	Revised study design to add a 24-week treatment extension period and a 32-week post-treatment follow-up period. During the treatment extension, participants in Arms 1 and 4 will have blinded treatment modified to 30 mg/kg bimagrumab.	Compare the results of bimagrumab and/or semaglutide treatment for continued weight loss and fat mass reduction in the 2nd year of treatment, as well as in maintenance of weight loss and composition of weight regain, if any.<br>Allow participants on placebo or low dose bimagrumab in the 1st year to receive high dose bimagrumab during the 2nd year.
Section 1.1 – Synopsis<br>Section 4.1 - Overall Design<br>Section 5 – Study Population<br>Section 6.3 – Assignment to Study Intervention<br>Section 6.4.2 – Semaglutide	Total sample size increased from approximately 450 to 495 participants and from approximately 50 to 55 per treatment arm.	Based on revised sample size calculation in accordance with revised endpoints; also, to account for the increased number of expected participant withdrawals due to the extended duration of study.

Section # and Name	Description of Change	Brief Rationale
Section 9.5 – Sample Size Determination		
Section 1.1 – Synopsis<br>Section 3.1 – Estimands<br>Section 9.1 Statistical Analyses<br>Section 9.4 – Treatment Extension and Post-Treatment Follow-up Period Analyses<br>Section 9.5 – Sample Size Determination	Updated statistical analysis plans, including:<br>• Revised the sample size determination;<br>• Identifying Week 48 as the point of primary efficacy analysis;<br>• Added analyses for the treatment extension and follow-up periods	Provide an overview of the statistical plan for the revised study design.
Section 1.1 – Synopsis<br>Section 3.1 – Estimands<br>Section 9.1 Statistical Analyses	'Intention-to-treat' population redefined to include only randomized participants who received at least one dose of study medication.	Remove from primary analysis participants who randomize into a treatment arm but did not receive treatment.
Section 1.3 – Schedule of Activities<br>Section 4.1 – Overall Design<br>Section 7.1 – Participant Discontinuation of Study Intervention<br>Section 7.2 – Participant Discontinuation/Withdrawal from the Study	For participants permanently discontinuing study intervention early, change the required timeframe for conduct of an End of Study visit from 8 weeks to 12 weeks after last dose of bimagrumab/placebo.	Evaluate the participant for safety after the bimagrumab/placebo is expected to have been eliminated.
Section 4.1 – Overall Design<br>Section 5.4 – Screen Failures<br>Section 8 – Study Assessments and Procedures<br>Section 8.5 – Pharmacokinetics	Assessment windows added or clarified as follows:<br>• Screening window revised to allow 1 repeat of any assessment that falls outside of Week -6 to Day -1;<br>• Added a window for Week 4 dosing;<br>• Added a window for collection of post-dose bimagrumab PK samples.	Prevent the exclusion of otherwise eligible participants based solely on timing of assessments in relation to dosing.<br><br>Clarify allowable windows for select assessments.
Section 4.1 – Overall Design	Specify the requirement for dietary intake evaluation via 24-hour recall assessments at monthly dietician outreach.	Clarify the required assessments to be conducted and data collected during the dietary consultations.

Section # and Name	Description of Change	Brief Rationale
Section 4.2 – Scientific Rationale for Study Design<br>Section 9.1 Statistical Analyses	FDA guidance for primary efficacy endpoints in phase 2 trials was added.	Clarify the FDA's recommendations regarding the study's primary endpoint.
Section 5.2 – Exclusion Criteria	Exclusion Criteria refined:<br>• #18 specifies medication associated with severe pancreatitis, such as valproate<br>• #20 replaces BUN exclusion with serum creatine >1.5x ULN	Prevent concomitant medications from contributing to an increased risk of on-study pancreatitis.<br><br>More closely align the measure of impaired renal function with clinical practice.
Section 5.3.1 – Meals and Dietary Restrictions	Added provision for alteration of on-study dietary restrictions if healthy weight status is achieved.	Allow standard clinical practice in dietary counseling for study participants who achieve a healthy weight.
Section 5.4 – Screen Failures	Clarification of screen failures to include participants who choose to not proceed to enroll.	Account for the fact that participants otherwise eligible may be categorized as screen failures if they chose to withdraw consent prior to dosing.
Section 6.4 – Blinding	Clarified that blinding will remain intact at least until database is locked for the core and extension treatment periods.	Specify the duration of study blind as a result of the study extension. The primary analysis remains at Week 48, which is now mid-way through the study instead of at the conclusion.
Section 6.6.1 – Bimagrumab	Bimagrumab dosing modification may be allowed for safety reasons.	Removed incongruity between this section and the guidance provided in Appendices 6, 7, and 8.
Section 6.9.1 – Prohibited Medicine	Prohibited medications and recommended actions refined:<br>• Hormone replacement therapy in women addressed;<br>• Allow short term courses of glucocorticoids;<br>• Require Medical Monitor consultation for medications that may cause weight change;	Refined the prohibition of medications based on their situational potential to significantly impact safety or efficacy.<br><br>Prevent valproate from contributing to the risk of on-study pancreatitis.

Section # and Name	Description of Change	Brief Rationale
	• Prohibit the use of valproate.	
Section 7.1.1 – Liver, Pancreas, or Muscle Related Events Stopping Criteria<br>Section 7.1.2 – Temporary Discontinuation<br>Section 8.3.4 – Clinical Safety Laboratory Tests<br>Section 10.7 – APPENDIX 7: Lipase and Amylase Safety<br>Section 10.8 – APPENDIX 8: Creatine Kinase Safety	Provided greater specificity in the guidance for study treatment interruption and/or discontinuation as a result of liver, pancreas, or muscle related events, including a requirement to review lab results at Week 4 prior to administering bimagrumab/placebo dose.	Provide Investigators with more detailed guidance on how to assure the safety of participants experiencing one of these events.
Section 7.4 – Treatment Arm and Study Stopping/Pausing Rules	Specified that SAEs of a similar type must be related to *blinded* treatment to qualify as part of the study stopping/pausing rules; expected SAEs do not qualify.	Ensure that SAEs related to semaglutide and those that are expected per the bimagrumab Reference Safety Information do not contribute to study stopping or pausing.
Section 8.1– Administrative Procedures	Specified data collection needs related to obesity history and obesity-related comorbidities.	Provide additional detail regarding key obesity-related data collection.
Section 8.4.5 – Treatment Emergent Adverse Events of Special Interest<br>Section 10.6 – Liver Safety<br>Section 10.7 – Lipase and Amylase Safety<br>Section 10.8 – Creatine Kinase Safety	Revised the list of AESIs.	Definition of AESIs was revised; monitoring of laboratory test abnormalities is outlined in Appendices.
Section 8.6.1 – PD Biomarkers<br>Section 10.2 – APPENDIX 2: Clinical Laboratory Tests	Revised list of potential PD biomarkers to also include serum CTX, BSAP, and P1NP.	To allow further analysis of bone biomarkers.
Section 10.2 – APPENDIX 2: Clinical Laboratory Tests	Clarified laboratory tests:<br>• Screening only tests performed locally to include TSH and remove HBsAb and HBcAb<br>• Added anti-drug antibodies	Clarify/align tests being performed at screening with the Schedule of Assessments and Appendix 2.

Additional changes include grammatical and clarifying text as well as revisions to reflect the extended treatment period.

SUMMARY OF CHANGES – FOR AMENDMENT 2, PROTOCOL V. 2.0

Section # and Name	Description of Change	Brief Rationale
Section 1.1 – Synopsis<br>Section 2.1 – Study Rationale<br>Section 2.3.2.1 - Bimagrumab<br>Section 3.0 – Objectives,<br>Section 9 – Statistical Considerations	Revised primary endpoint from waist circumference to body weight and reordered secondary objectives and endpoints.	Revised in response to FDA feedback.
Section 1.1 – Synopsis<br>Section 10.1.6 – Data Safety Committee	Revised timing of the first DMC meeting from 30 to 45 days after the first 25% of participants have completed dosing.	The 2 week delay will allow for collection of safety data from the 0.5 mg semaglutide + bimagrumab treatment arms.
Section 1.1 - Synopsis	Added clarification on approved dosage substitution for semaglutide.	Due to uncertain availability of semaglutide dosage forms, accepted dose levels for dose escalation are indicated.
Section 1.1 - Synopsis<br>Section 5.1 - Inclusion Criteria<br>Section 5.3.3 - Other restrictions<br>Section 10.4 - Appendix 4: Contraceptive Guidance	Clarified the criterion to have an IUD in place from 3 months prior to the baseline visit and not the screening visit. Additionally, clarified the use of a barrier for these participants starts from the screening visit and last up to 4 months after the last dose of bimagrumab/placebo.	The revised timeframe is deemed to be sufficient to ensure there are no safety issues with the placement of the IUD.
Section 1.1 – Synopsis<br>Section 5.2 - Exclusion Criteria	Key Exclusion Criteria – clarified exclusion of subjects with diabetes	Diabetic subjects on antidiabetic drug or with HbA1c≥6.5% will be excluded for this study of non-diabetic obesity.
Section 3.1 –Estimands	Estimand section revised based on the primary endpoint of body weight using the treatment policy strategy which include all subjects without relevance to intercurrent events.	Section edited to match the revised primary endpoint.
Section 1.3 - Schedule of Activities (SoA)	Added a row for “Days” and corrected the table header at week 36 to read P12.	Editorial/clarification change.
Section 1.3 - Schedule of Activities (SoA)	Added optional future use of samples informed consent as an assessment	Added for clarity.

Section # and Name	Description of Change	Brief Rationale
Section 2.3.1.1 - Investigational Intervention Bimagrumab	Added potential risk of disruption of menstrual cycles in premenopausal women with an IUD implant, due to decreased FSH levels.	Risk language in response to FDA feedback.
Section 4.1 - Baseline<br>Section 8.0 - Study Assessment and Procedures	Revised baseline window for DXA from 7 days prior to the baseline visit to 14 calendar days prior to the baseline visit. Non-baseline DXA windows revised to be -2 weeks to +1 week of the scheduled time for all other visits. The flexibility of the week 4 dosing visit was removed.	Based on site feedback, DXA assessment window extended to allow sufficient time for DXA scans to be scheduled around study visits.
Section 4.1 -Baseline	Clarified that baseline assessments should be completed at least 15 minutes prior to dosing.	Editorial/clarification change.
Section 8.4.1 – Time Period for Collecting AEs	Non-serious AEs will be collected after first dose of study medication(s) instead of after screening.	To avoid incomplete entry of medical history, medical conditions identified during the screening period will be captured as medical history.
Section 8.4.4 – Pregnancy	Pregnancies in female partners of male participants will be reported.	Outcomes of pregnancies in female participants as well as female partners of male participants will be recorded.
Section 9.3 – Interim Analysis	Removed the plan for an interim analysis.	Due to the expected rate of recruitment, an interim analysis is no longer required for development planning.
Section 10.2 – Clinical Laboratory Tests	HCV RNA removed from the screening tests	HCVAb will be used to detect hepatitis C infection; HCV RNA may be used to confirm active infection.

Additional changes include grammatical and clarifying text as well as revisions to reflect the extended treatment period.

SUMMARY OF CHANGES – FOR AMENDMENT 1.1, PROTOCOL V. 1.1

Section # and Name	Description of Change	Brief Rationale
Table of contents	Administrative	Table of Contents updated

SUMMARY OF CHANGES – FOR AMENDMENT 1, PROTOCOL V. 1.0

Section # and Name	Description of Change	Brief Rationale
1.0 Synopsis<br>1.2 Study Schema<br>1.3 Schedule of Activities<br>1.4 Study Design	Study duration increased from 28 weeks to 48 weeks. Corresponding additions to assessments:<br>• Phone calls at Weeks 32, 36 and 44; on-site visits at Weeks 40 and 48.<br>• Dosing events at Weeks 28 and 40.<br>• DXA at Week 48<br>• Safety/efficacy assessments at Weeks 40 and 48	Dosing through week 48 with placebo control will showcase greater effect of bimagrumab and combinations with semaglutide across multiple measures of efficacy.
1.0 Synopsis<br>3.0 Objectives, Endpoints and Estimands<br>9.0 Statistical Considerations	Changes in objectives and endpoints related to the increased duration of the study and the change in the lower dose of the semaglutide treatment arms.	Changes were required to harmonize with the study design.
1.0 Synopsis<br>4. Study Design<br>5. Study Population<br>2.2.2 Teratogenicity and Reproductive Toxicity Data	Age limit of 40+ years for females lowered to 18+ who are post-menopausal or post-surgically sterilized or have in place an IUD and are willing to use a form of barrier contraception	Expanded age range may provide exposure, safety and efficacy data in the 18-40 age range for females, more reflective of the intended Phase 3 study population, as recommended by FDA.
4.2 Scientific Rationale for Study Design<br>4.3 Justification for Dose<br>6.1 Study interventions Administered<br>6.6 Dose Modification<br>9.1 Statistical Analysis	Semaglutide low dose increased from 0.5 mg to 1 mg	Longer dosing period (through week 48) allows for adequate titration and exploration of an approved dose of semaglutide (Wegovy®, Ozempic®).
1.0 Synopsis<br>5.1 Inclusion Criteria<br>5.2 Exclusion Criteria	Refined inclusion criterion:<br>• Stable body weight as +/-5 kg<br>• Diagnosis of diabetes includes HbA1c ≥ 6.5%	Clarified information previously in both inclusion and exclusion criteria
1.0 Synopsis<br>9.4 Sample Size Determination	Modified sample size calculations	Longer duration of study allows for power calculation based on larger expected treatment difference vs placebo, anticipated dropout rate, and multiple endpoint testing.

9.3 Interim Analysis	Interim Analysis (group unblinding only) added at Week 24	Provides limited, group unblinded data readout to facilitate clinical development program planning.

TABLE OF CONTENTS

SPONSOR SIGNATURE PAGE 2
INVESTIGATOR'S AGREEMENT 3
MEDICAL MONITOR NAME AND CONTACT INFORMATION 4
DOCUMENT HISTORY 4
SUMMARY OF CHANGES – FOR AMENDMENT 5, PROTOCOL V. 5.0 5
SUMMARY OF CHANGES – FOR AMENDMENT 4, PROTOCOL V. 4.0 8
SUMMARY OF CHANGES – FOR AMENDMENT 3, PROTOCOL V. 3.0 10
SUMMARY OF CHANGES – FOR AMENDMENT 2, PROTOCOL V. 2.0 14
SUMMARY OF CHANGES – FOR AMENDMENT 1.1, PROTOCOL V. 1.1 15
SUMMARY OF CHANGES – FOR AMENDMENT 1, PROTOCOL V. 1.0 16
TABLE OF CONTENTS 18
LIST OF TABLES 23
LIST OF ABBREVIATIONS 24
1. PROTOCOL SUMMARY 28
1.1. Synopsis 28
1.2. Study Schema 34
1.3. Schedule of Activities (SoA) 35
2. INTRODUCTION 40
2.1. Study Rationale 40
2.2. Background 41
2.2.1. Preclinical Pharmacology 42
2.2.2. Clinical Data 43
2.3. Benefit/Risk Assessment 44
2.3.1. Risk Assessment 44
2.3.1.1. Investigational Intervention Bimagrumab 44
2.3.1.2. Semaglutide (Wegovy®, Ozempic®) 46
2.3.1.3. Study Procedures 48
2.3.2. Benefit Assessment 49
2.3.2.1. Bimagrumab 49
2.3.2.2. Semaglutide 49
2.3.3. Overall Benefit Risk Conclusion 49

3. OBJECTIVES, ENDPOINTS, AND ESTIMANDS 50
3.1. Estimands..52
3.1.1. Estimands of Secondary Objectives 52
4. STUDY DESIGN 54
4.1. Overall Design 54
4.2. Scientific Rationale for Study Design 57
4.3. Justification for Dose 58
4.3.1. Bimagrumab Doses..58
4.3.2. Semaglutide Doses...59
4.4. End-of-Study Definition 59
5. STUDY POPULATION 59
5.1. Inclusion Criteria 60
5.2. Exclusion Criteria 60
5.3. Lifestyle Considerations 63
5.3.1. Meals and Dietary Restrictions..63
5.3.2. Activity 64
5.3.3. Other Restrictions 64
5.3.4. Support During Treatment Withdrawal Period...................................64
5.4. Screen Failures...64
6. STUDY INTERVENTION(S) AND CONCOMITANT THERAPY 64
6.1. Study Interventions Administered 66
6.2. Preparation, Handling, Storage, and Accountability 68
6.3. Assignment to Study Intervention 68
6.4. Blinding 69
6.4.1. Bimagrumab and Placebo 69
6.4.2. Semaglutide 69
6.4.3. SAE and Unblinding...69
6.4.4. Unblinded Personnel...69
6.5. Study Intervention Compliance 70
6.6. Dose Modification 70
6.6.1. Bimagrumab 70
6.6.2. Semaglutide 70

6.7. Continued Access to Study Intervention after the End of Treatment 71
6.8. Treatment of Overdose 71
6.8.1. Bimagrumab 71
6.8.2. Semaglutide 71
6.9. Prior and Concomitant Therapy 71
6.9.1. Prohibited Medicine 72
6.9.2. Prohibited Non-Pharmacological Concomitant Treatments 73
6.9.3. Recommended Treatment of Adverse Events 73
7. DISCONTINUATION OF STUDY INTERVENTION, PARTICIPANT DISCONTINUATION / WITHDRAWAL AND STUDY STOPPING / PAUSING 73
7.1. Participant Discontinuation of Study Intervention 73
7.1.1. Liver, Pancreas, or Muscle Related Events Stopping Criteria 74
7.1.2. Temporary Discontinuation 74
7.2. Participant Discontinuation/Withdrawal from the Study 74
7.3. Lost to Follow-up 75
7.4. Treatment Arm and Study Stopping/Pausing Rules 75
8. STUDY ASSESSMENTS AND PROCEDURES 76
8.1. Administrative Procedures 76
8.2. Efficacy / Pharmacodynamics Assessments 77
8.2.1. Anthropometric Measurements 77
8.2.1.1. Body Weight 77
8.2.1.2. Waist Circumference 77
8.2.1.3. Body Mass Index 77
8.2.1.4. Bioelectrical Impedance Analysis 77
8.2.2. DXA Scan 78
8.2.3. Grip Strength 78
8.2.4. Impact of Weight on Quality of Life-Lite Clinical Trials Version 78
8.2.5. Short Form (SF-36) Health Survey 78
8.2.6. Physical Activity Monitoring Via Actigraphy 78
8.2.6.1. Lipid Profile 78
8.2.6.2. Glucose Metabolism Parameters: HbA1c, Fasting Insulin and Glucose 79
8.3. Safety Assessments 79

8.3.1. Physical Examinations 79
8.3.2. Vital Signs 79
8.3.3. Electrocardiograms 80
8.3.4. Clinical Safety Laboratory Tests 80
8.3.5. Pregnancy Testing 81
8.3.6. Hematology/Coagulation 81
8.3.7. Blood Chemistry 81
8.3.8. Urinalysis and Urine Chemistry 81
8.4. Adverse Events, Treatment Emergent Adverse Events, Serious Adverse Events, and Other Safety Reporting 81
8.4.1. Time Period and Frequency for Collecting AE, TEAE and SAE Information 82
8.4.2. Follow-up of AEs and SAEs 82
8.4.3. Regulatory Reporting Requirements for SAEs 82
8.4.4. Pregnancy 83
8.4.5. Treatment Emergent Adverse Events of Special Interest 83
8.5. Pharmacokinetics 83
8.6. Pharmacodynamics 84
8.6.1. PD Biomarkers 84
8.7. Genetics 84
8.8. Immunogenicity Assessments 85
9. STATISTICAL CONSIDERATIONS 85
9.1. Statistical Analyses 85
9.1.1. Multiplicity Adjustment 86
9.2. Analysis Sets 86
9.3. Interim Analysis 87
9.4. Treatment Extension and Post-Treatment Follow-Up Period Analyses 87
9.5. Sample Size Determination 87
10. SUPPORTING DOCUMENTATION AND OPERATIONAL CONSIDERATIONS 88
10.1. APPENDIX 1: Regulatory, Ethical, and Study Oversight Considerations 88
10.1.1. Regulatory and Ethical Considerations 88
10.1.2. Financial Disclosure 88
10.1.3. Informed Consent Process 89

10.1.4. Recruitment Strategy 89
10.1.5. Data Protection 89
10.1.5.1. Future Use of Stored Specimens and Data 89
10.1.6. Data Safety Committee 90
10.1.7. Site Monitoring 91
10.1.8. Clinical Monitoring 91
10.1.9. Audits and Inspections 92
10.1.10. Data Quality Assurance 92
10.1.11. Data Handling and Recordkeeping 94
10.1.12. Study and Site Start and Closure 95
10.1.13. Publication Policy 95
10.2. APPENDIX 2: Clinical Laboratory Tests 96
10.3. APPENDIX 3: AEs and SAEs: Definitions and Procedures for Recording, Evaluating, Follow-up, and Reporting 100
10.3.1. Definition of AE 100
10.3.2. Definition of SAE 101
10.3.3. Recording and Follow-Up of AE and/or SAE 102
10.3.4. Reporting of SAEs 103
10.4. APPENDIX 4: Contraceptive Guidance 105
10.5. APPENDIX 5: Genetics 106
10.5.1. Optional Consent 106
10.5.2. Use/Analysis of DNA 106
10.5.3. Storage of Genetic Samples 106
10.6. APPENDIX 6: Liver Safety: Suggested Actions and Follow-up Assessments 107
10.7. APPENDIX 7: Lipase and Amylase Safety 109
10.8. APPENDIX 8: Creatine Kinase Safety 110
11. REFERENCES 111

LIST OF TABLES

Table 1: Table of Objectives and Endpoints 50
Table 2: Study Interventions Administered 66
Table 3: Study Treatment Arms 67
Table 4: Prohibited Medications 72
Table 5: Safety Laboratory Tests 96
Table 6: Other Laboratory Tests (Central Lab) 98
Table 7: Liver Event and Laboratory Trigger Definitions 107
Table 8: Actions Required for Liver Events and Laboratory Triggers 107
Table 9: Follow Up Requirements for Liver Laboratory Triggers - Isolated Hyperbilirubinemia 108
Table 10: Safety Monitoring Guidance for Amylase and Lipase Elevations 109
Table 11: Safety Monitoring Guidance for Creatine Kinase Elevations 110

LIST OF ABBREVIATIONS

Abbreviation or Specialist Term	Explanation
ActRII	Activin Receptor Type II
ActRIIA	Activin Receptor Type II A
ActRIIB	Activin Receptor Type II B
ADA	Anti-Drug-Antibodies
AE	Adverse Event
AESI	Adverse Events of Special Interest
ALT	Alanine Aminotransferase
ALP	Alkaline Phosphate
AST	Aspartate Aminotransferase
aPTT	Activated Partial Thromboplastin Time
AUC	Area Under the Curve
BIA	Biomolecular Interaction Analysis
BMD	Bone Mineral Density
BMI	Body Mass Index
bpm	Beats Per Minute
BSAP	Bone Specific Alkaline Phosphatase
CBC	Complete Blood Count
CIOMS	Council For International Organizations of Medical Sciences
CCK18	Caspase-Cleaved K18
CK	Creatine Kinase
CL	Clearance
C_{max}	Maximum or Peak Concentration
CRF	Case Report Form
CRO	Contract Research Organization
CTC(AE)	Common Terminology Criteria (For Adverse Events)
CTX	C-terminal telopeptide
DMC	Data Monitoring Committee
DNA	Deoxyribonucleic Acid
DXA	Dual-Energy X-Ray Absorptiometry
ECG	Electrocardiogram
EDC	Electronic Data Capture

Abbreviation or Specialist Term	Explanation
EOS	End of Study
FAS	Full Analysis Set
FBM	Fat Body Mass
FFM	Fat-Free Mass
FSH	Follicle Stimulating Hormone
GDF11	Growth Differentiation Factor 11
GCP	Good Clinical Practice
GGT	Gamma-Glutamyl Transferase
GLP1	Glucagon-Like Peptide-1
HbA1c	Hemoglobin A1C
HBV	Hepatitis B Virus
HCV	Hepatitis C Virus
HDL	High Density Lipoprotein
HIV	Human Immunodeficiency Virus
HOMA2	Homeostasis Model Assessment, version 2
hsCRP	High-sensitivity C-reactive protein
IA	Interim Analysis
IB	Investigator's Brochure
ICH	International Conference on Harmonization
ICE	Intercurrent Event
IEC	Independent Ethics Committees
IL-6	Interleukin 6
IL-18	Interleukin 18
IgG	Immunoglobulin G
IMP	Investigational Medicinal Product
INR	International Normalized Ratio
IRB	Institutional Review Boards
ITT	Intention to Treat
IUD	Intrauterine Device
i.v.	Intravenous
IWQOL-Lite-CT	Impact of Weight on Quality of Life-Lite Clinical Trials
IWRS	Interactive Web Response Systems

Abbreviation or Specialist Term	Explanation
LBM	Lean Body Mass
LDH	Lactate Dehydrogenase
LDL	Low Density Lipoprotein
miR-122	MircoRNA 122
MRI	Magnetic Resonance Imaging
MTC	Medullary Thyroid Carcinoma
NGAL	Neutrophil gelatinase-associated lipocalin
NOAEL	No Observed Adverse Event Level
PD	Pharmacodynamic(s)
PI	Package Insert
PK	Pharmacokinetic(s)
PoC	Proof of Concept
PT	Prothrombin Time
QOL	Quality of Life
QTc	Corrected QT Interval
QUICKI	Quantitative Insulin-Sensitivity Check Index
SAE	Serious Adverse Event
SAF	Safety Analysis Set
SAP	Statistical Analysis Plan
SAT	Subcutaneous Adipose Tissue
SF-36	Short Form 36 Health Survey Questionnaire
SGLT2	Sodium-glucose Cotransporter-2
sIBM	Sporadic Inclusion Body Myositis
s.c.	Subcutaneous
SD	Standard Deviation
SGOT	Serum Glutamic-Oxaloacetic Transaminase
SGPT	Serum Glutamate Pyruvate Transaminase
SoA	Schedule of Activities
SOM	Study Operations Manual
SUSAR	Suspected Unexpected Serious Adverse Reactions
T2DM	Type 2 Diabetes Mellitus
TBW	Total Body Water

Abbreviation or Specialist Term	Explanation
TC	Total Cholesterol
TG	Triglyceride
TEAE	Treatment Emergent Adverse Event
TGF-β	Transforming Growth Factor Beta
ULN	Upper Limit of Normal
VAT	Visceral Adipose Tissue
WC	Waist Circumference

1. PROTOCOL SUMMARY

1.1. Synopsis

Sponsor/Company	Versanis Bio, Inc.
Investigational Product	Bimagrumab
Protocol Number	VER201-PH2-031
Title	A randomized, double-blind, placebo-controlled study of intravenous bimagrumab, alone or in addition to open label subcutaneous semaglutide to investigate the efficacy and safety in overweight or obese men and women
Brief Title	Safety and efficacy of bimagrumab and semaglutide in adults with overweight or obesity
Study Phase	2
Study Type	Interventional
Rationale	Most weight loss programs, including obesity pharmacotherapy, reduce body weight; however, this often occurs at the expense of lean mass. Bimagrumab has been shown to reduce fat mass accompanied by an increase in muscle mass. This study investigates if bimagrumab, alone or in addition to standard of care semaglutide, can preserve/increase muscle mass in the presence of weight and fat mass loss.
Primary Objective	1. To assess treatment effect of bimagrumab, semaglutide, and bimagrumab in addition to semaglutide vs placebo on body weight
Secondary Objectives	1. To assess the treatment effect of bimagrumab, semaglutide, and bimagrumab in addition to semaglutide vs placebo on waist circumference (WC, cm)<br>2. To assess treatment effects on fat mass, visceral adipose tissue (VAT), trunk fat mass, subcutaneous adipose tissue (SAT), and lean mass (body composition)<br>3. To assess the safety and tolerability of bimagrumab, semaglutide and bimagrumab in addition to semaglutide<br>4. To assess the proportion of patients in each treatment group experiencing a change in the categorical classification of obesity based on BMI and waist-to-height ratio (WHtR) group<br>5. To assess treatment effects on glucose metabolism<br>6. To assess treatment effects on self-reported health status and weight-related quality of life
Primary Endpoint	1. Change from baseline in total body weight at 48 weeks
Secondary Endpoints	1. Change from baseline in WC (cm) at 48 and 72 weeks<br>2. a. Change from baseline at 48 and 72 weeks in total body fat mass (kg and % body fat) by dual-energy x-ray absorptiometry (DXA)<br>b. Change from baseline at 48 weeks in VAT, SAT, and trunk fat mass (kg) by DXA<br>c. Proportion of participants who achieve a reduction from baseline at 48 weeks in the following measures of obesity:

	i. WC ≥ 5 cm<br>ii. Body weight ≥ 5%, ≥ 10%, and ≥ 15%<br>iii. Fat mass ≥ 5%, ≥ 10%, and ≥ 15% by DXA<br>iv. Fat mass ≥ 10% with <5% decrease (or an increase) in lean mass by DXA<br>d. Percentage of weight loss due to fat mass or lean mass at 48 weeks by DXA<br>e. Change from baseline at 48 and 72 weeks in fat mass (kg and % body fat) by bioelectrical impedance analysis (BIA)<br>f. Change from baseline at 48 and 72 weeks in total body lean mass (kg and % body lean) by DXA and BIA and appendicular lean mass by DXA<br>3. Safety and tolerability measurements throughout 48 weeks:<br>a. Incidence and severity of treatment emergent adverse events (TEAEs), treatment-related AEs, serious adverse events (SAEs) and evaluation of TEAEs of special interest (including significant muscle, skin, and gastrointestinal TEAEs, malignancy, and pancreatitis)<br>b. Clinical safety laboratory evaluation, including anti-drug antibodies (ADA)<br>c. Vital signs (temperature, blood pressure, pulse rate)<br>4. Proportion of patients with change from baseline in categorical classification of obesity at any time up to 48 weeks:<br>a. BMI categories:<br>i. Healthy weight: 18.5 kg/m^2 to 24.9 kg/m^2<br>ii. Overweight: 25 kg/m^2 to 29.9 kg/m^2<br>iii. Obesity class 1: 30 kg/m^2 to 34.9 kg/m^2<br>iv. Obesity class II: 35 kg/m^2 to 39.9 kg/m^2<br>v. Obesity class III: ≥ 40 kg/m^2<br>b. WHtR ratio categories: <0.5; 0.5-0.59; ≥0.6 [1]<br>5. Change from baseline in HbA1c at 48 weeks<br>6. Change from baseline at 24, 48, and 72 weeks in:<br>• Quality of Life Short Form 36 survey (SF-36) total score and physical functioning score<br>• Impact of Weight on Quality of Life-Lite for Clinical Trials survey (IWQoL-Lite for CT) total score and physical function score

<table>
<tr><td>Study Design</td><td>This is a multicenter, randomized, double-blind, placebo-controlled (for bimagrumab) study of i.v. bimagrumab, alone or in addition to open label s.c. semaglutide to investigate efficacy and safety in overweight or obese men and women.

Participants will be randomized to one of the following nine treatment arms (bimagrumab or placebo by i.v. infusion, semaglutide by s.c. injection; exact doses of semaglutide, by subcutaneous injection, to be determined, not to exceed 2.5 mg) for the 48-week core treatment period:
1. Placebo + no semaglutide
2. Placebo + semaglutide 1.0 mg
3. Placebo + semaglutide 2.4 mg
4. Bimagrumab 10 mg/kg + no semaglutide
5. Bimagrumab 10 mg/kg + semaglutide 1.0 mg
6. Bimagrumab 10 mg/kg + semaglutide 2.4 mg
7. Bimagrumab 30 mg/kg + no semaglutide
8. Bimagrumab 30 mg/kg + semaglutide 1.0 mg
9. Bimagrumab 30 mg/kg + semaglutide 2.4 mg

The core treatment period is followed by a 24-week open-label treatment extension period (which will remain blinded until after the 48-week database lock and study unblinding), during which 2 of the treatment arms will follow a modified treatment assignment:
1. Switch from placebo to bimagrumab 30 mg/kg + no semaglutide
2. No change; Placebo + semaglutide 1.0 mg; placebo replaced by no treatment after study unblinding
3. No change; Placebo + semaglutide 2.4 mg; placebo replaced by no treatment after study unblinding
4. Switch from bimagrumab 10 mg/kg to 30 mg/kg + no semaglutide
5. No change; bimagrumab 10 mg/kg + semaglutide 1.0 mg
6. No change; bimagrumab 10 mg/kg + semaglutide 2.4 mg
7. No change; bimagrumab 30 mg/kg + no semaglutide
8. No change; bimagrumab 30 mg/kg + semaglutide 1.0 mg
9. No change; bimagrumab 30 mg/kg + semaglutide 2.4 mg

The extension period is then followed by a 32-week post-treatment follow-up period, during which all study treatments will be withdrawn for all arms.

The study will consist of a screening visit to assess eligibility, a baseline visit, followed by clinic visits/phone contacts every 4 weeks during the treatment and follow-up periods.</td></tr>
<tr><td>Number of participants (planned)</td><td>Approximately 495; approximately 55 participants per treatment group</td></tr>
<tr><td>Investigational product, dosage and mode of administration</td><td>1. Low dose bimagrumab – 10 mg/kg by i.v. infusion
2. High dose bimagrumab – 30 mg/kg by i.v. infusion
For both dose levels, loading doses at Weeks 1 (Day 1) and 4, followed by q12 week dosing (Weeks 16, 28, 40, 52, and 64); there will be no loading dose for Arm 1 during switch from placebo to bimagrumab.</td></tr>
</table>

Reference therapy, dosage and mode of administration:	Bimagrumab placebo: Formulation buffer composed of histidine, trehalose and polysorbate by i.v. infusion.<br>Doses at Weeks 1 (Day 1), 4, 16, 28, and 40; at Weeks 52 and 64, placebo infusion will be replaced by no treatment after the 48-week database lock and study unblinding.
Comparator therapy, dosage and mode of administration	1. Low dose semaglutide – 1.0 mg s.c. weekly<br>Dose escalation schedule listed below<br>• Weeks 1 (Day 1) to 4: 0.25 mg s.c. weekly<br>• Weeks 5 to 8: 0.5 mg s.c. weekly<br>• Weeks 9 to 71: 1.0 mg s.c. weekly<br>2. High dose semaglutide – 2.4 mg s.c. weekly<br>Dose escalation schedule listed below (exact doses to be determined, not to exceed 2.5 mg)<br>• Weeks 1 (Day 1) to 4: 0.25 mg s.c. weekly<br>• Weeks 5 to 8: 0.5 mg s.c. weekly<br>• Weeks 9 to 12: 1.0 mg s.c. weekly<br>• Weeks 13 to 16: 1.7 mg s.c. weekly*<br>• Weeks 17 to 71: 2.4 mg s.c. weekly*<br>*Semaglutide doses of 1.7 mg and 2.4 mg may be substituted with 1.5 mg and 2.5 mg, respectively, based on availability of semaglutide pens.
Duration of Study	Up to 110 weeks, which includes an up to 6-week screening period, a baseline visit, a 72-week treatment period (48 weeks core plus 24 weeks extension), and a 32-week post-treatment follow-up period.
Key Inclusion Criteria	• A written informed consent must be obtained before any study-related assessments are performed.<br>• Men and women between 18 and 80 years, inclusive; women of child-bearing potential (defined as those who are not post-menopausal or post-surgical sterilization) must meet both of the following criteria:<br>• Two negative pregnancy tests (at screening and at randomization, prior to dosing)<br>• Use of intrauterine device, from at least 3 months before the baseline visit through at least 4 months after the last dose of bimagrumab/placebo i.v., and an additional contraceptive (barrier) method from screening through at least 4 months after the last dose of bimagrumab/placebo i.v.<br>• Body mass index (BMI) ≥ 30 kg/m^2 or BMI ≥ 27 kg/m^2 with one or more obesity-associated comorbidities (e.g., hypertension, insulin resistance, sleep apnea, or dyslipidemia)<br>• Stable body weight (± 5 kg) within 90 days of screening, and body weight <150 kg<br>• Have a history of at least one self-reported unsuccessful behavioral effort to lose body weight

	• Able to communicate well with the Investigator, comply with the study requirements and adhere to the diet and activity programs for the study duration
Key Exclusion Criteria	• History of, or known hypersensitivity to, monoclonal antibody drugs or a contraindication to semaglutide (Ozempic® or Wegovy®)<br>• Use of other investigational drugs at the time of enrollment or within 30 days or 5 half-lives of enrollment, whichever is longer, or longer if required by local regulations<br>• Treatment with any medication for the indication of obesity within the past 30 days before screening<br>• Diagnosis of diabetes, requiring current use of any antidiabetic drug or HbA1c ≥ 6.5%<br>• *Note: Metabolic syndrome is not an exclusion, even if managed with an anti-diabetic drug such as metformin or an SGLT2 inhibitor. A diagnosis of prediabetes or impaired glucose tolerance managed exclusively with non-pharmacologic approaches (e.g., diet and exercise) is not an exclusion.*<br>• Any chronic infections likely to interfere with study conduct or interpretation such as hepatitis B (HBV), hepatitis C (HCV), or human immunodeficiency virus (HIV). History of hepatitis A or hepatitis C successfully treated is not exclusionary. Active COVID-19 infection.<br>• Donation or loss of 400 mL or more of blood within 8 weeks prior to initial dosing, or longer if required by local regulation, or plasma donation (> 250 mL) within 14 days prior to the first dose<br>• Any disorder, unwillingness, or inability not covered by any of the other exclusion criteria, which in the Investigator's opinion, might jeopardize the participant's safety or compliance with the protocol
Sample Size Justification:	Approximately 495 participants will be randomized to 1 of 9 treatment groups. The sample size calculation is based on the primary efficacy estimand for weight. Based on the FDA guidance on treatment for weight management, the minimum meaningful treatment effect for weight loss is 5% of baseline body weight over 1 year of treatment. Assuming the minimum treatment effect of 5% weight loss with a standard deviation (SD) of 8, for a minimum effect size of 0.62 [2, 3], a sample size of 45 participants per treatment arm will have approximately 83.6% statistical power to detect a treatment difference between any active group and placebo/no treatment using a 2-sided t-test at alpha=0.05. Assuming a 20% dropout rate, enrollment will target 55 participants per treatment arm.<br>Sample size was estimated using a t-test for 2 means in NQuery Advisor V9.1 (**www.statsols.com**). [4, 5]

Statistical Methods	All randomized participants who received at least one dose of study medication will be included in an "intention-to-treat" population (treatment policy strategy). Baseline and efficacy results will be summarized by randomized treatment arm with active treatment arms compared to placebo/no treatment, and combination treatment arms to each monotherapy of the same dose.<br>Safety and tolerability results will be summarized by actual treatment received.<br>A detailed statistical analysis plan (SAP) will be finalized prior to database lock and will describe all planned analyses.
Data Monitoring/Other Committee	A data monitoring committee (DMC) will be convened to review the available safety data after 25% of participants have been treated for at least 45 days, and periodically thereafter, with ad hoc meetings as necessary. The committee will consist of at least 2 independent clinicians, knowledgeable in obesity treatment, and 1 independent biostatistician. Up to 2 additional non-voting members from the Sponsor with relevant safety and statistical expertise may also be included. Prior to convening the first DMC data review meeting, a DMC charter will be drafted and approved by the committee members. The primary role of the DMC is to advise on study conduct to ensure participant safety. This may include recommendations to discontinue a treatment arm or the study as a whole.

1.2. Study Schema

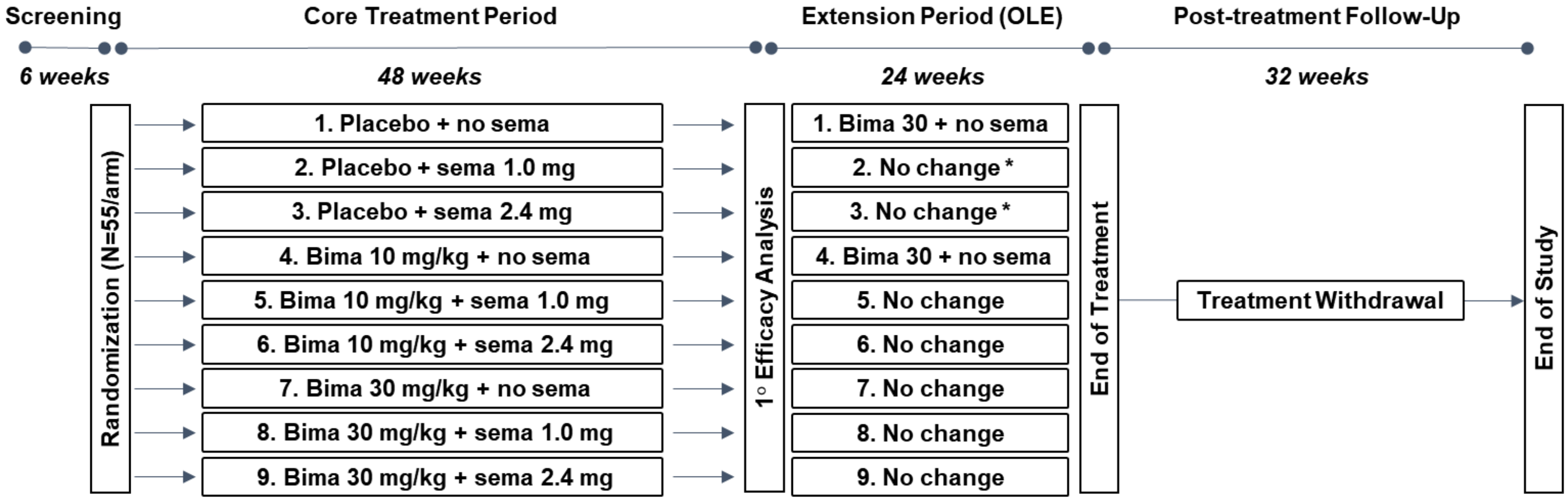

Bima = bimagrumab; sema = semaglutide

- Bima/placebo IV doses at Weeks 0, 4, 16, 28, 40, 52, and 64
- Sema SC doses weekly through Week 71
- DXA scans at Weeks 0, 12, 24, 48, 72 and 96

* Placebo replaced by no treatment in arms 2 and 3 after Week 48 database lock and study unblinding

1.3. Schedule of Activities (SoA)

Core Treatment Period (Screening through Week 48)

Study Phase	SCR	B/L	Core Treatment												
**Visit (V), Phone (P)**	**V1**	**V2**	**P3**	**V4**	**P5**	**V6**	**V7**	**P8**	**V9**	**V10**	**P11**	**P12**	**V13**	**P14**	**V15**
**Week [b]**	**-6 to -1**	**1**	**2**	**4**	**8**	**12**	**16**	**20**	**24**	**28**	**32**	**36**	**40**	**44**	**48**
**Day**	**-41 to -1**	**1**	**8**	**29**	**57**	**85**	**113**	**141**	**169**	**197**	**225**	**253**	**281**	**309**	**337**
**PARTICIPANT RELATED INFORMATION AND ASSESSMENTS**															
Clinic Visit	X	X		X		X	X		X	X			X		X
Phone Call			X		X			X			X	X		X	
Informed consent	X														
Optional genetic and future use of samples informed consent [c]	X														
Demography	X														
Inclusion/Exclusion criteria	S	S[d]													
Medical History	X	X													
Con Meds/Therapies	X	X													
Drug screen	S														
Body height	X														
Hep B and C/HIV	X														
Lipid Panel	X														
FSH [e], HbA1c, TSH [e]	S														
**RANDOMIZATION & DRUG ADMINISTRATION**															
Randomization		X													
Drug administration: bimagrumab/placebo IV		X		X[f]			X			X			X		
Drug administration: semaglutide SC (self-administered) [g]		Weekly to week 71													
**EFFICACY**															
Body weight, including BIA [h]	X	X		X		X	X		X	X			X		X
Waist circumference	X	X		X		X	X		X	X			X		X

Study Phase	SCR	B/L	Core Treatment											
**Visit (V), Phone (P)**	**V1**	**V2**	**P3**	**V4**	**P5**	**V6**	**V7**	**P8**	**V9**	**V10**	**P11**	**P12**	**V13**	**P14**	**V15**
**Week [b]**	**-6 to -1**	**1**	**2**	**4**	**8**	**12**	**16**	**20**	**24**	**28**	**32**	**36**	**40**	**44**	**48**
**Day**	**-41 to -1**	**1**	**8**	**29**	**57**	**85**	**113**	**141**	**169**	**197**	**225**	**253**	**281**	**309**	**337**
DXA Scan		X[b]				X			X						X
Grip strength		X				X			X						X
QoL Questionnaires [i]		X				X			X						X
Actigraphy		X													
**SAFETY**															
Adverse Events		X													
Physical Examination [j]	X	X		X		X			X				X		X
Vital signs [k]	X	X		X		X	X		X	X			X		X
ECG	X					X									X
Hematology [l] (CBC)	X	X		X		X			X				X		X
Clinical chemistry [l]	X	X		X		X			X				X		X
Coagulation [l]	X	X		X		X			X				X		X
Urine chemistry [l]		X				X			X				X		X
Urinalysis	X	X				X			X				X		X
Pregnancy test [n]	X	X		X		X	X		X	X			X		X
Blood draw: Safety Biomarkers [l, o]		X [o]		X [o]		X [o]			X [o]				X [o]		X [o]
Blood draw: PD biomarkers [l, m, p]		X				X			X						X
Blood draw: Anti-drug antibodies		X		X		X			X						X
Blood draw: Pharmacokinetics [b, q]		X		X		X	X		X	X					X
Training: eDiary, exercise program, semaglutide self-administration		X													
Dietary/physical activity counselling		X		X	X	X	X	X	X	X	X	X	X	X	X
Exercise program [r]		X													
eDiary completion [s]		X													

Extension and Post-Treatment Follow-Up Periods (Week 52 through End of Study)

Study Phase	Extension Treatment					EOT[a]	Post-Treatment Follow-Up							EOS[a]
Visit (V), Phone (P)	V16	P17	P18	V19	P20	V21	P22	P23	V24	P25	P26	V27	P28	V29
Week [b]	52	56	60	64	68	72	76	80	84	88	92	96	100	104[t]
Day	365	393	421	449	477	505	533	561	589	617	645	673	701	729
**PARTICIPANTS RELATED INFORMATION AND ASSESSMENTS**														
Clinic Visit	X			X		X			X			X		X
Phone Call		X	X		X		X	X		X	X		X	
Con Meds/Therapies	X													
**RANDOMIZATION & DRUG ADMINISTRATION**														
Drug administration: bimagrumab/placebo IV	X			X[f]										
Drug administration: semaglutide SC (self-administered) [g]	Weekly to Week 71													
**EFFICACY**														
Body weight, including BIA [h]	X			X		X			X			X		X
Waist circumference	X			X		X			X			X		X
DXA Scan						X						X		
Grip strength						X						X		
QoL Questionnaires [i]						X						X		
Actigraphy	X													
**SAFETY**														
Adverse Events	X													
Physical Examination [j]				X		X			X			X		
Vital signs [k]	X			X		X			X			X		X
ECG														
Hematology [l] (CBC)				X		X			X			X		
Clinical chemistry [l]				X		X			X			X		
Coagulation [l]				X		X			X			X		
Urine chemistry [l]				X		X			X			X		
Urinalysis				X		X			X			X		

Study Phase	Extension Treatment					EOT[a]	Post-Treatment Follow-Up							EOS[a]
**Visit (V), Phone (P)**	**V16**	**P17**	**P18**	**V19**	**P20**	**V21**	**P22**	**P23**	**V24**	**P25**	**P26**	**V27**	**P28**	**V29**
**Week [b]**	**52**	**56**	**60**	**64**	**68**	**72**	**76**	**80**	**84**	**88**	**92**	**96**	**100**	**104[t]**
**Day**	**365**	**393**	**421**	**449**	**477**	**505**	**533**	**561**	**589**	**617**	**645**	**673**	**701**	**729**
Pregnancy test [n]	X			X		X								
Blood draw: Safety Biomarkers [l, o]						X [o]						X [o]		
**SAFETY**														
Blood draw: PD biomarkers [l, m, p]						X						X		
Blood draw: Anti-drug antibodies						X								
Blood draw: Pharmacokinetics [b, q]						X								
**OTHER ASSESSMENTS**														
Dietary/physical activity counselling	X	X	X	X	X	X	X	X	X	X	X	X	X	
Exercise program [r]									X					
eDiary completion [s]									X					

Abbreviations: SCR, screening; B/L=baseline; D=day; EOS=end of study; V=visit; P=phone; BIA=bioimpedance analysis; DXA= dual-energy X-ray absorptiometry; HbA1C=hemoglobin A1C; PD=pharmacodynamic; ECG= electrocardiogram; Hep=hepatitis; HIV=human immunodeficiency virus; CBC=complete blood count ; aPTT=activated partial thromboplastin time; PT/INR=prothrombin time/international normalized ratio; IUD=intrauterine device; DNA=deoxyribonucleic acid; eDiary=electronic diary; S=remains as source data at site and is not collected in the eCRF.

a. **EARLY TREATMENT DISCONTINUATION:** At the time of discontinuing treatment early, the End of Treatment (EOT) visit should be conducted as soon as possible, followed by the End of Study (EOS) visit at least 12 weeks after the last dose of bimagrumab/placebo.
b. **VISIT:** Visit windows are defined in Section 8. Windows for PK collection times are defined in Section 8.5
c. **OPTIONAL GENETIC INFORMED CONSENT:** May be collected at a future visit.
d. **INCLUSION/EXCLUSION**: Eligibility to be confirmed prior to dosing.
e. **SCREENING FSH, HbA1c and TSH**: Follicle-stimulating hormone testing to be conducted for post-menopausal women at screening (see footnote o for other timepoints). HbA1c required for all participants at screening. Thyroid-stimulating hormone testing to be conducted at screening unless a normal value is documented within 6 months prior to screening.
f. **WEEKS 4 AND 64 PRE-DOSE SAFETY LABS**: Prior to administering the Week 4 and Week 64 doses of bimagrumab/placebo, Investigators must obtain and review results of safety labs against the safety guidance presented in Sections 10.6, 10.7, and 10.8, Appendices 6, 7, and 8.
g. **SEMAGLUTIDE ADMINISTRATION**: Semaglutide is to be administered s.c. weekly by the participant at home. On study visit days, participants will take their dose on site, in the presence of study personnel, after study assessments. The last dose of semaglutide is taken at the beginning of week of 71, on Day 498.
h. **BODY WEIGHT, INCLUDING BIA:** Bioelectrical impedance scale to be used to measure weight, body fat, and lean (muscle) mass.
i. **QOL QUESTIONNAIRES:** Questionnaires include IWQoL-Lite for CT and SF-36.

j. **PHYSICAL EXAMINATION:** A complete physical examination is required, including skin assessment for acne or lesions and, for males, assessment of gynecomastia.
k. **VITAL SIGNS**: Includes body temperature, blood pressure, pulse rate.
l. **CLINICAL LABORATORY TESTS**: Refer to Appendix 2 for a complete list of all analytes included in each panel.
m. **BLOOD DRAWS**: Blood draws should be collected in the fasted state (approximately 8 hours), except for the screening visit, where fasting is not required. Time of last meal should be noted.
n. **PREGNANCY TEST**: Serum pregnancy test is required for all female participants at screening. Urine pregnancy test can be used at all other visits for pre-menopausal female participants, unless serum is required based on local requirements.
o. **SAFETY BIOMARKERS**: A sample for safety biomarkers will be collected for all participants but will only be analyzed for participants with abnormal liver or pancreatic test results per safety labs.
p. **BLOOD DRAW: PD BIOMARKERS:** PD markers may include adipokines, fasting lipid profile, inflammation biomarkers, endocrine biomarkers, TGFβ superfamily, and bone biomarkers.
q. **PHARMACOKINETIC SAMPLING:** For bimagrumab – samples are to be collected pre-dose and again 15 min after the bimagrumab infusion and flush have completed on specified dosing days, and as convenient at EOS visit. For semaglutide – samples are to be collected pre-dose on specified visits and as convenient at EOS visit.
r. **EXERCISE PROGRAM**: Participants will be encouraged to exercise for at least 150 minutes per week.
s. **eDIARY**: Exercise and dosing information collected.

2. INTRODUCTION

2.1. Study Rationale

Obesity is one of the greatest public health challenges of the 21st century. Its prevalence has tripled in many countries since the 1980s, and the numbers of those affected continue to rise at an alarming rate. In 2016, globally more than 1.9 billion adults (39%) were overweight; of these, 650 million (13%) were obese. In addition to causing various physical disabilities and psychological problems, excess weight drastically increases a person's risk of developing a number of comorbidities, including cardiovascular disease, cancer, insulin resistance and type 2 diabetes mellitus (T2DM) [6]. The Global Burden of Disease study reported that 4.7 million people died prematurely in 2017 as a result of obesity [7].

A body weight reduction of 5-10% has been associated with significant and clinically meaningful improvements in insulin sensitivity, glycemic control, hypertension, and dyslipidemia. Combining lifestyle management with pharmacotherapy is increasingly recognized as an effective and safe treatment option for many patients with obesity [8]. Currently, 5 medications are on the market for long term use in the US, the latest is once weekly s.c. semaglutide (a GLP1-receptor agonist), approved in 2021 for chronic weight management in adults with obesity (BMI >30 kg/m^2) or overweight with at least one weight-related condition (BMI >27 kg/m^2).

Adipose tissue, in particular, visceral adipose tissue (VAT), is involved in energy storage and is considered an endocrine and paracrine organ that affects several metabolic processes, through the release of cytokines and bioactive mediators. Cytokines and mediators can influence body weight, as well as insulin resistance and pathogenesis of diabetes mellitus, lipid metabolism and inflammation, thus explaining premature atherosclerosis in people with obesity [9-11]. VAT rather than body mass index (BMI) determines metabolic risk and is associated with metabolic syndrome even in participants with normal BMI, defined as normal weight obese [12-14]. Therefore, loss of VAT might be of great importance to prevent cardiovascular disease.

Most weight loss programs are able to reduce body weight within the first few weeks; however, a significant amount of the weight loss includes loss of LBM. Also, obesity pharmacotherapy results in loss of LBM in connection with loss of FM [15], so it is important to focus on body composition in an effort to maintain LBM. The loss of LBM is problematic for several reasons, including impacts on health, ability to conduct activities of daily living and potential effects on emotion and psychological states. The loss of LBM impedes sustainability of successful obesity therapy by causing lowered resting energy expenditure/metabolism, fatigue, decline in neuromuscular function, and increased risk for injury [16-18]. In an effort to offset LBM loss, studies have incorporated exercise in conjunction with a weight loss program; however, this has only provided modest benefit, most likely due to the modest caloric expenditure experienced with moderate exercise [19, 20]. In general, it is recognized that for every 1 kg of FM lost, approximately 0.26 kg of lean tissue is lost in post-menopausal women [21]. Importantly, the lean mass lost is only partially regained with weight gain. There are no approved obesity interventions that preserve or build lean mass while promoting fat mass loss.

Bimagrumab is a recombinant human monoclonal antibody binding to the activin receptor type II (ActRII). Bimagrumab previously has been studied in muscle wasting indications and has been shown to increase skeletal muscle mass in healthy volunteers, in patients with sporadic inclusion body myositis (sIBM), and in patients with sarcopenia.

In a recent randomized double-blind, placebo-controlled study including 75 overweight or obese patients with type 2 diabetes mellitus (T2DM), bimagrumab 10 mg/kg i.v. every 4 weeks for 48 weeks in addition to lifestyle intervention, resulted in a marked loss in FM, an increase in LBM, and improvement in a range of metabolic biomarkers [5]. Participants lost 5.1 kg ($p<0.001$) body weight in the bimagrumab group vs. the placebo group with a decrease in placebo-adjusted WC of 9.5 cm ($p<0.001$). Patients lost 20.5% of FM, had a 3.6% increase in LBM, and had a decrease of 0.76 percentage points in their HbA1c levels, whereas placebo patients only had a decrease in total body FM of 0.5%, LBM reduction of 0.8%, and an increase of 0.04 percentage points in HbA1c levels. The loss in total body FM and gain in LBM led to net weight losses of 6.5% and 0.8% in patients receiving bimagrumab or placebo, respectively. Additionally, greater reductions in VAT and hepatic fat fraction were observed in the bimagrumab group versus placebo group. The expansion of the lean mass (i.e., skeletal muscle) compartment in the presence of negative energy balance and weight loss is a novel feature in weight loss pharmacotherapy. Overall, adverse effects (AEs) were balanced between the bimagrumab and placebo groups. Participants on bimagrumab vs. placebo reported more muscle symptoms, diarrhea and nausea. The frequency of gastrointestinal AEs was highest during the first dosing period with bimagrumab and diminished with repeat dosing. More patients in the bimagrumab group experienced transient elevations of pancreatic and liver enzymes, which tended to subside after the first dose of bimagrumab. The etiology of these elevations is unclear but could be related to mobilization of adipocyte triglycerides and amino acids as a new metabolic equilibrium is reached during early bimagrumab dosing.

The planned study is designed to evaluate the effect of bimagrumab alone or in addition to the approved obesity therapeutic semaglutide in obese or overweight participants with at least one obesity related co-morbidity, without T2DM. The main endpoint in the study is body weight. Other endpoints include waist circumference, body composition, and evaluation of the impact of treatment on metabolic parameters and safety.

2.2. Background

The activin type 2 receptors ActRIIA and ActRIIB, abbreviated as ActRII, modulate signals for ligands belonging to the transforming growth factor beta (TGF-β) superfamily. These included growth differentiation factor 8 (GDF8, myostatin), GDF11, and activins, which are all negative regulators of skeletal muscle growth, acting via the ActRII receptor signaling pathway to inhibit muscle protein synthesis and myocyte differentiation and proliferation. A pharmacological way to block these ligands' action to increase muscle mass and hence exercise capacity is through inhibition of ActRII [22]. Blockade of ActRII results in hypertrophy of skeletal muscle through an increase in size of existing myofibers [23-25], which in turn is hypothesized to improve peripheral insulin sensitivity, since skeletal muscle is one of the major glucose-utilizing tissues in the body [22].

Activin receptor signaling is also active in adipose tissue and contributes to the regulation of adipose tissue mass. Adipocytes express the highest levels of the type 1 receptor ALK7 and are

the major tissue source of activin B. In rodents, knockout of the ALK7 receptor decreases total fat mass and confers resistance to diet-induced obesity. In humans, single nucleotide polymorphisms in the ALK7 gene are associated with lower BMI and resistance to obesity [26, 27].

Bimagrumab, a recombinant human, monoclonal antibody (modified IgG, λ2) binds competitively to the ActRII with a greater affinity than its natural ligands.

Information currently available on pharmacology, toxicology, pharmacokinetics, and pharmacodynamics are reported in the Investigator Brochure (please refer to most recent edition) and has been obtained from in vitro experiments, animal studies, toxicology studies, and human trials.

2.2.1. Preclinical Pharmacology

The co-administration of CDD866, a murinized version of bimagrumab, with semaglutide was studied in obese C57Bl/6J mice. Animals were fed a high fat diet (60% of total caloric input as fat) for 20 weeks and reached body weights of approximately 50 grams (twice normal) at the time the therapeutic interventions were initiated. The high fat diet was continued throughout the experimental period and the two drugs were evaluated individually and when co-administered, along with vehicle-alone groups. The primary efficacy assessment was total body fat mass determined weekly by magnetic resonance spectroscopy. Each drug alone exhibited the expected effect on body fat mass and lean mass and when administered together, the decrease in body fat mass was at least additive. Greater than 30% body fat mass loss was observed over 3 weeks with the co-administration of the two highest dose levels of bimagrumab and semaglutide. Other than the expected decrease in food intake in the semaglutide arms of the study, there were no safety or tolerability issues observed. Specifically, a quantitative open field test of behavior showed no significant differences between the drug-treated groups and vehicle-treated groups.

Teratogenicity and Reproductive Toxicity Data

In embryo-fetal development studies, bimagrumab was seen to be developmentally toxic in the rat and rabbit and teratogenic in the rat (NOAEL of 1 mg/kg). The exact mechanism, direct or indirect, underlying the reproductive organ changes observed with bimagrumab is unclear. A pre- and postnatal development (dose range-finding) study in the rat was terminated at the end of the weaning phase of pup development because of a high incidence of litter death in the control animals (reason unknown). In rats dosed at 1 or 10 mg/kg there was an increase in the number of females unable to complete the process of parturition and an increase in the number of stillborn pups. This observation was not seen in the control animals.

Women of child-bearing potential, who are not post-menopausal or post-surgical sterilization, must use highly effective contraception during treatment and for 4 months after their last dose of bimagrumab due to the risk of teratogenicity and embryo-fetal death observed with bimagrumab in animals.

Clinical studies evaluating single and multiple doses of bimagrumab showed no difference in changes in testicular volume or sperm count or quality between male participants receiving bimagrumab and those receiving placebo. Furthermore, monoclonal antibodies are large molecules that do not readily (< 1%) pass the blood-testis barrier and are not readily excreted in

seminal fluid. As a result of this data, no contraception is required for male participants participating in bimagrumab trials.

2.2.2. Clinical Data

Human safety and tolerability

Bimagrumab has been studied in Phase 2/3 clinical studies that have evaluated the safety, tolerability, PK and efficacy of bimagrumab in different populations, including 5 phase 2a/proof of concept studies (recovery from disuse, sporadic inclusion body myositis (sIBM), sarcopenia with mobility limitations, and cancer cachexia and chronic obstructive pulmonary disease cachexia), two phase 2b studies (sarcopenia and hip fracture recovery) and one phase 2b/3 study in sIBM patients. Study results to date indicate bimagrumab has a good safety profile; is generally well tolerated; has predictable nonlinear kinetics caused by target-mediated drug disposition; and reliably increases muscle volume and decreases adipose tissue levels. Body composition changes begin within 2 weeks of administration and continue throughout exposure in both healthy volunteers and patients regardless of age.

Transient cases of episodic, involuntary muscle contractions (referred to as “cramps or spasms”), acne and diarrhea of mostly mild intensity have been observed in study participants with symptoms occurring more frequently in participants receiving the highest doses of drug (30 mg/kg). Several participants have discontinued treatment in earlier studies because of an AE (exacerbation of acne, muscle cramps or diarrhea). Data from a dedicated hormonal study showed no effect on the hormonal axis except for a decrease in FSH levels in postmenopausal women with complete resolution after bimagrumab clearance. Data from a dedicated 24-week cardiac safety study indicated no effect of bimagrumab on multiple cardiac parameters in the context of the expected skeletal muscle hypertrophy.

Human pharmacokinetic data

Bimagrumab exhibits a non-linear kinetic profile following single and repeat i.v. administrations, very likely caused by target-mediated drug disposition. The total clearance (CL) of bimagrumab is concentration-dependent and is the sum of linear CL and non-linear CL. The half-life is also concentration dependent. The half-life ranges from 19 days in the linear portion of the profile (high bimagrumab concentrations) to 5 days when the maximum CL is achieved. Based on graphical exploration, the nonlinear CL saturation seems to occur below a threshold serum concentration of approximately 10 μg/mL.

Bimagrumab, like any human IgG antibody, is expected to be eliminated via intracellular catabolism, following fluid-phase or receptor mediated endocytosis. Since the majority of immunoglobulins undergo proteolytic degradation, bimagrumab is not expected to yield active metabolites. Since bimagrumab is a human IgG with large molecular size (~150 kDa), little intact immunoglobulin can be filtered by the kidney, hence little/no antibody is expected to be excreted in the urine.

There was only limited evidence of an effect of the dietary protein intake on the PK of bimagrumab. PK profiles of bimagrumab in healthy volunteers of Japanese descent, older adults up to 83 years of age, obese adults, and patients with sIBM were similar to those of healthy younger adults.

Human pharmacodynamic data

Consistent, measurable increases in muscle size have been observed in clinical studies. In healthy volunteers, single doses of 3, 10 and 30 mg/kg i.v. and repeat doses of 3 and 10 mg/kg resulted in gains in thigh muscle volume of +2.7-5.5% from baseline over placebo at 4−12 weeks post dose. The increase in muscle volume was associated with improvements in physical function in patients with sIBM (+6.0%) in an early PoC study. The muscle increase was also seen in a recent Phase 3 study, but the improved function was not repeated. Patients with sarcopenia saw 8% increases in muscle volume 8 weeks after a single dose of 30 mg/kg, which resulted in clinically relevant improvements in muscle strength and mobility performance.

Consistent, dose-dependent, linear decreases in total body fat measured by DXA have also been observed in clinical studies with bimagrumab. Reductions in fat mass of between 2.5 and 3.5 kg over 6 months occurred in participants with sarcopenia, hip fracture, sporadic inclusion body myositis and in healthy volunteers participating in a cardiac safety study and in elderly healthy participants. Efficacy results in obese and overweight patients with T2DM are reported in Section 2.1.

For more information on bimagrumab, please refer to the most recent version of the Investigators Brochure. For information on semaglutide, refer to the current Wegovy® or Ozempic® package insert.

2.3. Benefit/Risk Assessment

More detailed information about the known and expected benefits and risks and reasonably expected adverse events (AEs) of bimagrumab can be found in the Investigators Brochure and in the Package Insert for semaglutide.

2.3.1. Risk Assessment

2.3.1.1. Investigational Intervention Bimagrumab

A total of 21 clinical studies including 3 extension studies have been conducted with bimagrumab, including 1,540 adults, with 1,042 receiving the bimagrumab. Dose levels ranged from 0.01 mg/kg to 30 mg/kg, with larger studies using 1 mg, 3 mg, and 10 mg/kg as i.v. infusion, s.c. injections of 2 mg/kg and fixed doses of 52.5 mg, 150 mg, 300 mg, 525 mg, and 1500 mg; the majority of most studies assessed i.v. infusions of 10 mg/kg and 30 mg/kg. Treatment durations ranged from single to multiple doses over 6 to 12 months. The longest continuous exposure in obese adults with T2DM was 12 months with doses of 10 mg/kg body weight (1200 mg maximum) and 12-26 months in patients with sIBM.

Muscle symptoms

In a recent bimagrumab study in obese or overweight patients with T2DM, an increased AE incidence of muscle spasms (usually described as muscle cramps) of 15 (40.5%), all mild (32.4%) or moderate (8.1%) in intensity was observed in the bimagrumab group. One (2.7%) participant reported muscle fatigue and one (2.7%) reported muscle stiffness; both were mild. The majority of the AEs were judged to be related to study drug by the Investigator. In the placebo group there were 2 muscle events, one (2.6%) of muscle spasms and one (2.6%) of muscle twitching. In previous studies, in different indications, muscle symptoms were also seen

in the form of muscle spasms, tightness and twitching. In general, muscle symptoms are mostly mild or moderate, transient, painless, and of short duration. For the details of previous individual studies please refer to the Investigators Brochure.

The etiology and clinical meaning of these observations are not known. Similar symptoms have been reported with at least one other anabolic molecule, a beta-2 adrenergic agonist [28] and are commonly reported by adults performing muscle building exercise.

Skin symptoms

In the obesity study, in the bimagrumab group there were 4 (10.8%) participants with at least one AE of skin symptoms, while there were 7 (18.4% in the placebo group). All skin symptoms were mild. There were 4 participants in the bimagrumab group who reported AEs of acne (1), blister (1), and rash (2).

In previous studies, in different indications, skin symptoms were seen mostly in the form of acne and rash. In general, skin symptoms are mostly mild or moderate, transient, painless, and of short duration. For the details of previous individual studies please refer to the current edition of the Investigators Brochure.

Acne has been confirmed in these studies by dermatology consult and biopsy. Treatment has been successful with good skin hygiene using a 4-10% benzoyl peroxide wash and over the counter topical treatments. On occasion, a prescription, topical or oral antibiotic (i.e., minocycline 100 mg bid), was recommended. However, early standard of care intervention is recommended in participants when acne presents. A possible mechanistic link between bimagrumab and skin reactions remains unclear.

Diarrhea

In the obesity study, diarrhea was reported in 15 participants (40.5%, 35.1% mild, 5.4% moderate) in the bimagrumab group versus 4 (10.5%, 5.3% mild, 5.3% moderate) participants in the placebo group. Diarrhea has also been observed more frequently in other studies in different indications.

Reproductive organs

Data from a dedicated hormonal study demonstrated no clinically or statistically significant effect of bimagrumab on circulating testosterone levels and no effect on pituitary-adrenal axes in either gender and minimal effect on the pituitary-gonadal axes in men. Suppression of FSH levels in postmenopausal women and premenopausal women of non-childbearing potential has been observed with no associated safety risk identified. However, in premenopausal women with an IUD there is an associated risk of disruption of menstrual cycles. All effects were transient and resolved after bimagrumab exposure ended. Semen analysis data from the multiple dose study suggest that three doses of 10 mg/kg have no effect on sperm count or quality in males.

Women of childbearing potential must use highly efficacious contraception (see study eligibility criteria, Section 5).

Immunogenicity

A treatment related anti-drug antibody (ADA) response has been observed infrequently in participants treated with bimagrumab. Nevertheless, there was no evidence of an infusion or hypersensitivity reaction that could be related to immunogenicity or any sign of immune complex formation in the clinical trials performed so far. No positive signal in the immunogenicity assay was accompanied by a change in the bimagrumab PK profile.

Lipase and amylase elevations, and pancreatitis

Observations of dose-dependent, transient, elevations of lipase and/or amylase have been identified in several studies in the bimagrumab clinical program. The biological explanation for this temporal rise in pancreatic enzymes seen in some individuals is not yet fully understood.

As of 1 March 2022, 4 participants in 3 studies have experienced acute pancreatitis among 1042 participants administered bimagrumab. All 4 participants recovered from the acute pancreatitis, 2 discontinued study drug and 2 were considered to be related to study treatment. One participant on placebo experienced pancreatitis. To reduce the potential risk of elevated pancreatic enzymes and pancreatitis, study participants with acute or chronic pancreatitis or with elevated amylase and/or lipase will be excluded as per Section 5.2.

2.3.1.2. Semaglutide (Wegovy®, Ozempic®)

Risk of Thyroid C-Cell Tumors

In mice and rats, semaglutide caused a dose-dependent and treatment-duration-dependent increase in the incidence of thyroid C-cell tumors (adenomas and carcinomas) after lifetime exposure at clinically relevant plasma exposures. It is unknown whether semaglutide causes thyroid C-cell tumors, including medullary thyroid carcinoma (MTC), in humans, as human relevance of semaglutide-induced rodent thyroid C-cell tumors has not been determined. Cases of MTC in patients treated with liraglutide, another GLP-1 receptor agonist, have been reported in the post marketing period; the data in these reports are insufficient to establish or exclude a causal relationship between MTC and GLP-1 receptor agonist use in humans.

Acute pancreatitis

Acute pancreatitis, including fatal and non-fatal hemorrhagic or necrotizing pancreatitis, has been observed in patients treated with GLP-1 receptor agonists, including semaglutide. Acute pancreatitis was observed in patients treated with semaglutide in clinical trials. After initiation of semaglutide, observe patients carefully for signs and symptoms of acute pancreatitis (including persistent severe abdominal pain, sometimes radiating to the back, and which may or may not be accompanied by vomiting). If acute pancreatitis is suspected, semaglutide should promptly be discontinued, and appropriate management should be initiated. If acute pancreatitis is confirmed, semaglutide should not be restarted.

Acute Gallbladder Disease

In Wegovy® randomized clinical trials, cholelithiasis was reported by 1.6% of semaglutide -treated patients and 0.7% of placebo-treated patients. Cholecystitis was reported by 0.6% of semaglutide -treated patients and 0.2% of placebo-treated patients. Substantial or rapid weight loss can increase the risk of cholelithiasis; however, the incidence of acute gallbladder disease

was greater in semaglutide -treated patients than in placebo-treated patients, even after accounting for the degree of weight loss. If cholelithiasis is suspected, gallbladder investigations and appropriate clinical follow-up are indicated.

Hypoglycemia

Semaglutide lowers blood glucose and can cause hypoglycemia. In a trial of patients with type 2 diabetes and BMI greater than or equal to 27 kg/m^2, hypoglycemia (defined as a plasma glucose less than 54 mg/dL) was reported in 6.2% of semaglutide-treated patients versus 2.5% of placebo-treated patients. One episode of severe hypoglycemia (requiring the assistance of another person) was reported in one semaglutide-treated patient versus no placebo-treated patients.

Inform patients of the risk of hypoglycemia and educate them on the signs and symptoms of hypoglycemia.

Acute Kidney Injury

There have been post marketing reports of acute kidney injury and worsening of chronic renal failure, which have in some cases required hemodialysis, in patients treated with semaglutide. Patients with renal impairment may be at greater risk of acute kidney injury, but some of these events have been reported in patients without known underlying renal disease. Most of the reported events occurred in patients who had experienced nausea, vomiting, or diarrhea, leading to volume depletion.

Monitor renal function when initiating or escalating doses of semaglutide in patients reporting severe adverse gastrointestinal reactions. Monitor renal function in patients with renal impairment reporting any adverse reactions that could lead to volume depletion.

Hypersensitivity

Serious hypersensitivity reactions (e.g., anaphylaxis, angioedema) have been reported with semaglutide. If hypersensitivity reactions occur, discontinue use of semaglutide, treat promptly per standard of care, and monitor until signs and symptoms resolve. Do not use in patients with a previous hypersensitivity to semaglutide or any of the excipients in semaglutide. Anaphylaxis and angioedema have been reported with other GLP-1 receptor agonists.

Use caution in a patient with a history of anaphylaxis or angioedema with another GLP-1 receptor agonist because it is unknown whether such patients will be predisposed to these reactions with semaglutide.

Diabetic Retinopathy Complications in Patients with Type 2 Diabetes

In a trial of patients with T2DM and BMI greater than or equal to 27 kg/m^2, diabetic retinopathy was reported by 4.0% of semaglutide-treated patients and 2.7% placebo-treated patients.

In a 2-year trial with semaglutide 0.5 mg and 1 mg once-weekly injection in patients with type 2 diabetes and high cardiovascular risk, diabetic retinopathy complications occurred in patients treated with semaglutide injection (3.0%) compared to placebo (1.8%). The absolute risk increase for diabetic retinopathy complications was larger among patients with a history of diabetic retinopathy at baseline (semaglutide injection 8.2%, placebo 5.2%) than among patients without a known history of diabetic retinopathy (semaglutide injection 0.7%, placebo 0.4%).

Rapid improvement in glucose control has been associated with a temporary worsening of diabetic retinopathy. The effect of long-term glycemic control with semaglutide on diabetic retinopathy complications has not been studied. Patients with a history of diabetic retinopathy should be monitored for progression of diabetic retinopathy.

Heart Rate Increase

Mean increases in resting heart rate of 1 to 4 beats per minute (bpm) were observed in semaglutide-treated patients compared to placebo in clinical trials. More patients treated with semaglutide compared with placebo had maximum changes from baseline at any visit of 10 to 19 bpm (41% versus 34%, respectively) and 20 bpm or more (26% versus 16%, respectively).

Instruct patients to inform their healthcare providers of palpitations or feelings of a racing heartbeat while at rest during semaglutide treatment. If patients experience a sustained increase in resting heart rate, discontinue semaglutide.

Suicidal Behavior and Ideation

Suicidal behavior and ideation have been reported in clinical trials with other weight management products. Monitor patients treated with semaglutide for the emergence or worsening of depression, suicidal thoughts or behavior, and/or any unusual changes in mood or behavior. Discontinue semaglutide in patients who experience suicidal thoughts or behaviors. Avoid semaglutide in patients with a history of suicidal attempts or active suicidal ideation.

Adverse reactions

Semaglutide was evaluated for safety in 3 randomized, double-blind, placebo-controlled trials that included 2116 patients with overweight or obesity treated with semaglutide for up to 68 weeks and a 7 week off drug follow-up period. In clinical trials, 6.8% of patients treated with semaglutide and 3.2% of patients treated with placebo permanently discontinued treatment as a result of adverse reactions. The most common adverse reactions leading to discontinuation were nausea (1.8% versus 0.2%), vomiting (1.2% versus 0%), and diarrhea (0.7% versus 0.1%) for semaglutide and placebo, respectively. The most frequent adverse events reported as AEs were nausea/vomiting reported in 44% / 24% respectively of patients on semaglutide versus 16 % / 6% of patients on patients treated with placebo. Diarrhea/constipation was reported in 24% /24% respectively of patients on semaglutide and 16% / 10% of patients on placebo. Abdominal pain was reported in 20% of patients on semaglutide and 10% on placebo.

2.3.1.3. Study Procedures

Infusion risks

Infusion-related reactions can occur with monoclonal antibodies. Hypersensitivity reactions can manifest as fever, chills, urticaria, dyspnea, headaches, myalgia and/or hypotension. No serious infusion reaction (anaphylaxis) has been seen in patients treated with bimagrumab. If a severe hypersensitivity reaction occurs, administration of bimagrumab should be discontinued and appropriate therapy initiated.

Dual-Energy X-ray Absorptiometry (DXA)

DXA scans in this study will be used to assess bone mineral density and body composition (i.e., fat and lean mass). The effective radiation dose of a DXA whole body scan on an adult is 2.1

μSv. Therefore, the total amount of radiation exposure per participant from 6 DXA scans will be about 12.6 μSv over a period of 96 weeks. This amount of radiation is less than 14 days of background exposure (approximately 0.03 μSv per hour at sea level). For effective radiation doses under 3 μSv (300 mrem), the risk is considered to be minimal [29]. Therefore, the radiation exposure in this study involves minimal risk.

2.3.2. Benefit Assessment

All participants enrolled in the study may benefit from lifestyle interventions including diet and physical activity, and regular monthly contact with the site staff. Potential benefits associated with bimagrumab and semaglutide are described below.

2.3.2.1. Bimagrumab

Based on recent clinical data in overweight and obese diabetic participants, treatment with bimagrumab in non-diabetic obesity is expected to result in decreased body weight and waist circumference, with improved body composition resulting from loss of fat mass and increase of lean mass. In the diabetic participant study, bimagrumab also resulted in reduction in visceral fat mass, reduction in hepatic fat fraction, and improved insulin sensitivity and glucose control. Bimagrumab in combination with semaglutide may mitigate the loss of lean mass.

2.3.2.2. Semaglutide

Semaglutide is approved in the USA and the EU as a chronic weight management treatment in obese or overweight adults. Once weekly s.c. semaglutide results in substantial percentage change and absolute change in body weight as reported in a recent meta-analysis [4]. Compared with placebo, once weekly semaglutide also led to superior reductions in body weight, waist circumference and BMI compared with placebo; however, losses in lean mass (approximately 10%) were also observed [30]. Furthermore, the effect on improving other cardiometabolic risk factors and health-related quality of life was more pronounced for once-weekly semaglutide relative to placebo.

2.3.3. Overall Benefit Risk Conclusion

Considering the measures taken to minimize risk to participants participating in this study, the potential risks identified in association with bimagrumab are justified by the anticipated benefits that may be afforded to participants who are obese or overweight.

3. OBJECTIVES, ENDPOINTS, AND ESTIMANDS

Table 1: Table of Objectives and Endpoints

OBJECTIVES	ENDPOINTS
**Primary Objective**	
1. To assess the treatment effect of bimagrumab, semaglutide, and bimagrumab in addition to semaglutide vs placebo on body weight	1. Change from baseline in total body weight at 48 weeks

OBJECTIVES	ENDPOINTS
**Secondary Objectives**	
1. To assess the treatment effect of bimagrumab, semaglutide, and bimagrumab in addition to semaglutide vs placebo on waist circumference (WC)	1. Change from baseline in WC (cm) at 48 and 72 weeks
2. To assess treatment effects on fat mass, visceral adipose tissue (VAT), trunk fat mass, subcutaneous adipose tissue (SAT), and lean mass (body composition)	2. a. Change from baseline at 48 and 72 weeks in total body fat mass (kg and % body fat) by DXA<br>b. Change from baseline at 48 and 72 weeks in VAT, SAT, and trunk fat mass (kg) by DXA<br>c. Proportion of participants who achieve a reduction from baseline at 48 weeks in the following measures of obesity:<br>i. WC ≥ 5 cm<br>ii. Body weight ≥ 5%, ≥ 10%, and ≥15%<br>iii. Fat mass ≥ 5%, ≥ 10%, and 15% by DXA<br>iv. Fat mass ≥ 10% with <5% decrease (or an increase) in lean mass by DXA<br>d. Percentage of weight loss due to fat mass or lean mass at 48 weeks by DXA<br>e. Change from baseline at 48 and 72 weeks in fat mass (kg and % body fat) by bioelectrical impedance analysis (BIA)<br>f. Change from baseline at 48 and 72 weeks in total body lean mass (kg and % body lean) by DXA and BIA and appendicular lean mass by DXA

3. To assess the safety and tolerability of bimagrumab, semaglutide and bimagrumab in addition to semaglutide	3. Safety and tolerability measurements throughout 48 weeks:<br>a. Incidence and severity of treatment emergent adverse events (TEAEs), treatment-related AEs, serious adverse events (SAEs) and evaluation of TEAEs of special interest (including significant muscle, skin, and gastrointestinal TEAEs, malignancy, and pancreatitis)<br>b. Clinical safety laboratory evaluation, including anti-drug antibodies (ADA)<br>c. Vital signs (temperature, blood pressure, pulse rate)
4. To assess the proportion of patients in each treatment group experiencing a change in the categorical classification of obesity based on BMI and waist-to-height ratio (WHtR) group	4. Proportion of patients with change from baseline in categorical classification of obesity at any time up to 48 weeks:<br>a. BMI categories:<br>i. Healthy weight: 18.5 kg/m^2 to 24.9 kg/m^2<br>ii. Overweight: 25 kg/m^2 to 29.9 kg/m^2<br>iii. Obesity class 1: 30 kg/m^2 to 34.9 kg/m^2<br>iv. Obesity class II: 35 kg/m^2 to 39.9 kg/m^2<br>v. Obesity class III: ≥ 40 kg/m^2<br>b. WHtR ratio categories: <0.5; 0.5-0.59; ≥0.6 [1]
5. To assess treatment effects on glucose metabolism	5. Change from baseline in HbA1c at 48 weeks
6. To assess treatment effects on self-reported health status and weight-related quality of life	6. Change from baseline at 24,48, and 72 weeks in:<br>a. Quality of Life Short Form 36 survey (SF-36) total score and physical functioning score<br>b. Impact of Weight on Quality of Life-Lite for Clinical Trials survey (IWQoL-Lite for CT) total score and physical function score
**Exploratory Objectives**	
1. To evaluate treatment effects on Pharmacodynamic (PD) biomarkers, including metabolism, lipids, inflammatory, hormonal, and TGF-β superfamily ligands	1. Change from baseline in PD biomarkers (see Section 8.8) at 24 and 48 weeks
2. To evaluate treatment effects on grip strength	2. Change from baseline in grip strength using handgrip dynamometer (kg) at 24 and 48 weeks

3. To evaluate treatment effects on physical activity level	3. Change from baseline in step count as assessed by Wrist Actigraphy at 24 and 48 weeks relative to baseline
4. To evaluate treatment effects on insulin resistance	4. Change from baseline in HOMA2 and QUICKI at 24 and 48 weeks
5. To evaluate treatment effects on total and regional BMD	5. Change from baseline in total, spine, and hip bone mineral density (BMD) at 24 and 48 weeks
6. To evaluate the pharmacokinetics of repeat doses of bimagrumab.	6. PK parameters based on pre- (Ctrough) and post-dose levels during treatment
7. To evaluate the pharmacokinetics of repeat doses of semaglutide	7. PK parameters based on pre-dose (Ctrough) levels during treatment
8. To assess the treatment withdrawal effect of bimagrumab, semaglutide, and bimagrumab in addition to semaglutide on body weight, waist circumference, and body composition	For participants who complete 72 weeks and start the withdrawal period,<br>a) Change from baseline and % change in body weight, waist circumference, and body composition at 104 weeks<br>b) Percentage of participants who maintain ≥70%, 80% and 90% of the body weight lost during the treatment period

3.1. Estimands

The primary estimand will quantify the average treatment effect of bimagrumab, semaglutide or a combination of bimagrumab with semaglutide relative to placebo after 48 weeks, in all randomized participants who received at least one dose of study treatment, regardless of adherence to treatment and regardless of intercurrent events. This follows the "treatment policy" strategy which closely adheres to full analysis set (FAS) with an intention-to-treat (ITT) population.

The following expansion of the primary estimand will quantify the average treatment effect of combination treatment of semaglutide with bimagrumab relative to each monotherapy of the same dosage strength after 48 weeks, in all randomized participants regardless of adherence to treatment and regardless of intercurrent events (ICE).

As a sensitivity analysis, the primary estimand will also be assessed using the "trial product or hypothetical estimand" to quantify the average treatment effect in all participants who adhered to treatment and did not experience any ICEs. Intercurrent events will be detailed in the SAP.

3.1.1. Estimands of Secondary Objectives

The estimands of the secondary objectives will follow the treatment policy strategies outlined for the primary estimand. Details of the secondary estimands and the ICEs that are relevant will be provided in the SAP.

Statistical analyses for the treatment extension and post-treatment follow-up periods will be detailed in the SAP.

4. STUDY DESIGN

4.1. Overall Design

This is a non-confirmatory, randomized, double-blind, placebo-controlled (for bimagrumab) multi-center study of i.v. bimagrumab, alone or in addition to open label s.c. semaglutide, to investigate the efficacy and safety in adult men and women between the ages of 18 and 80, inclusive, who are obese or who are overweight with at least one obesity related co-morbidity. Women must be post-menopausal, post-surgically sterilized or using an intrauterine device.

Approximately 495 participants are planned to be enrolled and randomized to 1 of 9 treatment arms (see Section 6.1).

The total study duration will be approximately 104 weeks and will consist of an up to 6-week screening period, a 72-week treatment period (48 weeks core followed by 24 weeks of extension), and a 32-week post-treatment follow-up period. Overview of the study design can be found in the study schema in Section 1.2.

Screening (Week -6 to day -1)

Potential participants will undergo the screening activities listed in the Schedule of Activities (SoA) Section 1.3 to determine their eligibility for the study (see enrollment criteria Section 5). Screening assessments that are outside of the Week -6 to Day -1 window may be repeated once.

The Investigator must recommend a lifestyle intervention [31] that includes dietary counseling for weight loss with a daily caloric deficit of approximately 500 kcal and with protein intake of at least 1.2 g/kg/day to support muscle anabolism. Participants will also be encouraged to exercise for at least 150 minutes per week.

These lifestyle interventions will be explained during the screening visit and initiated at the baseline visit.

Baseline (Day 1, up to 15 minutes prior to dosing)

Eligible participants will return to clinic to undergo baseline assessments as defined in the SoA Section 1.3. Baseline assessments must be completed prior to dosing. DXA scans may be performed up to 14 calendar days prior to dosing.

During this visit, participants will be provided with an actigraphy device in the form of a wrist band. Participants will be advised to wear the band at all times for the entire course of the study. The band should be removed during showering and may be removed during sleep if desired.

Site staff will assist participants with downloading study applications on their mobile phone that will send visit reminders, collect patient reported outcomes and provide general study information to the participant.

Randomization and Dosing (Day 1)

Upon completion of all baseline activities, eligible participants will be randomized to one of the 9 treatment arms listed in Section 6.1.

Bimagrumab or placebo will be administered i.v. over a period of ~30 minutes, followed by flushing for ~15 minutes and a 15-minute post-flush observation period that will include safety

and tolerability observations and PK sampling. Bimagrumab will be dosed based on the previous visit body weight at 10 mg/kg or 30 mg/kg depending on the assigned treatment arm.

Participants assigned to semaglutide treatment arms will receive their first dose of semaglutide in clinic, along with training on how to store semaglutide pens and self-administer injections at home.

Following all assessments, participants may be discharged from the Investigator site when the Investigator judges them to be medically stable, in good general health, and not needing further observation.

Participants assigned to semaglutide treatment arms will be sent home with a semaglutide pen (to be self-administered, once weekly), disposal containers and instructions to return any unused pens to clinic at their next scheduled visit.

Core Treatment Period (Weeks 1 to 48)

For the duration of the core treatment period, participants should:

- Follow the dietary and exercise recommendations prescribed by the Investigator.
- Wear the actigraphy band.
- Complete weekly eDiaries. Occasional missed diaries are permissible in the absence of persistent non-compliance.

For participants assigned to semaglutide arms, self-administer weekly semaglutide injections and return all used and unused pens at their next clinic visit.

Participants will return to clinic:

- at Weeks 4, 16, 28 and 40 for i.v. bimagrumab or placebo dosing. Additional assessments will be performed as detailed in the SoA Section 1.3.
- at Weeks 12, 24, and 48 for DXA scanning and other assessments detailed in the SoA Section 1.3.

Site staff will call participants at Weeks 2, 8, 20, 32, 36, and 44 to ensure there are no safety issues and to address any questions the participant may have.

Qualified dieticians will reach out to participants on a monthly basis to provide dietary and exercise counseling, and to calculate participant's dietary intake via 24-hour recall assessment (see study operations manual for detailed description). If desired, these touchpoints may be combined with the in-clinic visits or safety calls.

Should participants report any adverse events over the phone, these will be recorded in the eCRF.

Open Label Extension Treatment Period (Weeks 49 to 72)

The open label extension treatment period will be conducted similarly to the core treatment period described above, however, participants randomized to study treatment arms 1 (bimagrumab placebo) and 4 (bimagrumab 10 mg/kg) will switch treatment to bimagrumab 30 mg/kg at Week 52. Dispensing of drug product in a manner that maintains the blind is described

in Section 6.4.4. Note, the extension treatment period will be made open label after the 48-week database lock and study unblinding.

For the duration of the extension treatment period, participants should continue to follow dietary and exercise recommendations, wear the actigraphy band, complete the weekly eDiaries, and must self-administer weekly semaglutide (for participants assigned to semaglutide arms) as in the core treatment period.

Participants will return to clinic at Weeks 52 and 64 for i.v. bimagrumab or placebo dosing. Additional assessments will be performed as detailed in the SoA Section 1.3. Note: no placebo infusions will be administered to treatment arms 2 and 3 after the study is made open label, e.g., after the Week 48 database lock and study unblinding.

Site staff will call participants at Weeks 56, 60, and 68 as described above. Should participants report any adverse events over the phone, these will continue to be recorded in the eCRF.

Qualified dieticians will continue to counsel participants on a monthly basis as described above.

End of Treatment Visit (End of Week 72)

Participants will return for the End of Treatment (EOT) Visit at the end of Week 72 or at the time of early termination to perform DXA scan and other assessments as detailed in the SoA Section 1.3. If study intervention is permanently discontinued early, the EOT visit should be conducted as soon as possible, per Section 7.1.

Post-Treatment Follow-Up Period (Weeks 73 to 104)

Upon completion of the EOT visit, all study treatment will be withdrawn (bimagrumab/placebo and semaglutide) and participants will be followed for an additional 32 weeks.

For the duration of the post-treatment follow-up period, participants should follow dietary and exercise recommendations, wear the actigraphy band, and complete the weekly eDiaries.

Participants will return to clinic at Weeks 84 and 96 for efficacy and safety assessments to be performed as detailed in the SoA Section 1.3.

Site staff will call participants at Weeks 76, 80, 88, 92, and 100, as described above. Should participants report any adverse events over the phone, these will continue to be recorded in the eCRF.

Qualified dieticians will continue to counsel participants on a monthly basis as described above.

Participants will return for their end of treatment follow up visit at the end of Week 96 for DXA scan and other assessments detailed in the SoA Section 1.3.

End of Study Visit (End of Week 104)

Participants will return for their final end of study (EOS) visit at the end of Week 104 for vital sign and BIA measurements. During this visit, site staff should assist participants in removing study-specific applications from their personal devices.

If study intervention is permanently discontinued early during one of the treatment periods, the EOT visit should be conducted at that time, followed by an EOS visit at least 12 weeks after the last dose of bimagrumab or placebo.

If study participation is permanently discontinued early during the post-treatment follow-up period, the EOS visit should be conducted at least 12 weeks after the last dose or bimagrumab or placebo.

4.2. Scientific Rationale for Study Design

The design of this Phase 2 study addresses the primary and the key secondary objectives of assessing if the efficacy of bimagrumab i.v. administered in addition to semaglutide s.c. is superior to either drug administered alone or to placebo and if the efficacy of bimagrumab i.v. is superior to s.c. semaglutide.

The rationale for key elements of the study design include:

- **Randomization**: To provide equal access to any of the treatment arms regardless of gender, age, or baseline characteristics.
- **Stratification**: To decrease the chance of an imbalance in gender across treatment arms
- **Triple-blind with regards to bimagrumab**: The participant, Investigator and Sponsor will be blinded to bimagrumab dose and placebo-bimagrumab to avoid bias in adverse event and efficacy reporting, and to avoid any potential confounding effect of intentional and unintentional behavioral changes by participants.
- **Open label with regards to semaglutide:** Commercially available semaglutide will be used in the study. Because it is delivered in a pre-filled syringe, packaged and labeled by the manufacturer, it is not possible to blind semaglutide. The inclusion of two doses: a very low dose 1.0 mg and the registered dose of 2.4 mg, as well as the addition of the double-blinded bimagrumab treatment to all semaglutide doses, should mitigate the risk of bias.
- **Placebo arm**: Inclusion of a placebo group will allow the analysis of whether or not bimagrumab treatment is more effective than a standard treatment approach based on lifestyle intervention.
- **Primary endpoint**: The FDA guidance for phase 3 clinical trials in weight management recommends that in general, a product can be considered effective for weight management if after 1 year of treatment either of the following occurs:
  - The difference in mean weight loss between the active-product and placebo-treated groups is at least 5 percent and the difference is statistically significant

or

  - The proportion of participants who lose greater than or equal to 5 percent of baseline body weight in the active-product group is at least 35 percent, is approximately double the proportion in the placebo-treated group, and the difference between groups is statistically significant.

  For phase 2 clinical trials, the FDA guidance states that primary efficacy endpoints should include a comparison of the mean absolute or percent change in body weight between the active-product and placebo-treated groups.

- **Population:** Men and women, fulfilling the criteria with regards to overweight and obesity from the FDA Draft Guidance [32] of being at significant risk for weight-related morbidity and mortality, and who have already tried and failed lifestyle modifications. Bimagrumab is teratogenic in animal studies, therefore women who are not post-menopausal or post-surgically sterilized must meet the relevant eligibility criteria to minimize the risk that women of child-bearing potential experience a contraceptive failure or should wish to become pregnant.
- **Treatment extension:** Continued weight loss may not be observed during the 2nd year of weight loss treatment, as demonstrated in the STEP 5 study [33]. The extension treatment period in the current study will compare the results of bimagrumab and/or semaglutide treatment for continued weight loss and fat mass reduction in the 2nd year of treatment.
- **Post-treatment follow-up:** Prevention of weight regain following successful weight loss is a key challenge for obesity management. After withdrawal of treatment, weight regain is common for methods of weight loss dependent on reduced caloric intake. One year after withdrawal of semaglutide, for example, participants in the STEP 1 trial regained two-thirds of their prior weight loss (Wilding 2022). The post-treatment follow-up period in the current study will compare the results of bimagrumab and/or semaglutide in maintenance of weight loss and composition of weight regain.

4.3. Justification for Dose

4.3.1. Bimagrumab Doses

This study will evaluate two doses of bimagrumab 10 mg/kg and 30 mg/kg i.v., administered at baseline, Weeks 4, 16, 28, 40, 52, and 64.

The dose of bimagrumab, 10 mg/kg i.v., administered to healthy volunteers and to obese/ overweight participants provided exposure levels above 10 µg/mL, at which the anabolic effect is observed, with levels maintained over the dosing interval of 4 weeks. PK/PD modelling has predicted that with dosing of 10 mg/kg at baseline, 4 weeks and 16 weeks, exposures will drop below 10 ug/mL for approximately half the time through 24 weeks and result in fat mass losses intermediate between those expected in the placebo group and the 30 mg/kg group. The 10 mg/kg dose group has been included to help determine whether a lower dose of bimagrumab, when added to semaglutide, might result in near-maximal efficacy, superior tolerability, or both.

The 30 mg/kg i.v. dose was administered as 2 doses 8 weeks apart in healthy volunteers and in patients with chronic obstructive lung disease with cachexia. A 30 mg/kg dose of bimagrumab is expected to achieve circulating levels sufficient to block the ActRIIB receptor (greater than ~10 µg/ml) over nearly all dosing intervals. A serum level of bimagrumab above this threshold resulted in an increase in thigh muscle volume in healthy volunteers.

The loading doses of bimagrumab 4 weeks apart is supported from a safety perspective by the findings from both the 13-week and 26-week toxicity studies with maximum exposure of 14 weekly doses of 100 mg/kg. AUC and C_{max} values of the 100 mg/kg i.v. dose level in the cynomolgus monkey toxicity studies are approximately 3 and 6 times greater than that expected

in humans from a second i.v. dose of 30 mg/kg of bimagrumab 8 weeks apart (14,710 µg/day/mL vs. 5340 µg/day/mL and 4480 µg/mL vs. 726 µg/mL). While this is the first time bimagrumab will be administered as seven 30 mg/kg doses, it is considered safe based on the 13- and 26-week toxicity study results and the clinical data noted previously. The PK/PD modeling predicts the 30 mg/kg doses at baseline, and Weeks 4, 16, 28, 40, 52, and 64 will maintain exposures above 10 µg/mL over nearly the entire 12-week interval between doses and yield a near-maximal treatment effect on fat mass loss.

Participants in core treatment arms 1 (placebo; no semaglutide) and 4 (bimagrumab 10 mg/kg; no semaglutide) will be switched to the higher dose of bimagrumab (30 mg/kg) during the 24-week extension period in order to allow these participants to receive a dose of bimagrumab predicted to achieve maximal treatment effect during the 2nd year.

4.3.2. Semaglutide Doses

Semaglutide is a glucagon-like peptide-1 (GLP-1) receptor agonist indicated as an adjunct to a reduced calorie diet and increased physical activity for chronic weight management in adult patients with an initial BMI of 30 kg/m^2 or greater (obesity) or 27 kg/m^2 or greater (overweight) in the presence of at least one weight-related comorbid condition (e.g., hypertension, type 2 diabetes mellitus, or dyslipidemia).

Semaglutide is administered s.c. once weekly, on the same day each week, at any time of day, with or without meals. Treatment is initiated at 0.25 mg once weekly for 4 weeks. In 4-week intervals, the dose unit is increased until a dose of 2.4 mg is reached. The maintenance dose of Wegovy® is 2.4 mg once weekly. In this study the higher dose of semaglutide is the approved dose for obesity, while the lower dose of 1.0 mg s.c. per week is an approved dose for diabetes, and is predicted to yield body weight loss intermediate between no semaglutide and the 2.4 mg dose. The 1.0 mg dose group has been included to help determine whether a lower dose of semaglutide, when added to bimagrumab, might result in near-maximal efficacy, superior tolerability, or both.

4.4. End-of-Study Definition

The end of the study is defined as the date of the last scheduled procedure shown in the schedule of activities for the last participant in the study, globally.

A participant is considered to have completed the study if the participant has completed all periods of the study including the last scheduled procedure shown in the SoA Section 1.3.

5. STUDY POPULATION

Approximately 495 overweight/obese men and women aged 18 – 80 years (inclusive) will be enrolled in the study.

The Investigator must ensure that all participants being considered for the study satisfy the inclusion/exclusion criteria. No additional criteria should be applied by the Investigator, in order that the study population will be representative of all eligible participants.

Participant selection is to be established by checking through all eligibility criteria at screening and baseline. A relevant record (e.g., checklist) of the eligibility criteria must be stored with the source documentation at the study site.

Deviation from any entry criterion (inclusion and exclusion) excludes a participant from enrollment into the study.

Prospective approval of protocol deviations to recruitment and enrollment criteria, also known as protocol waivers or exemptions, is not permitted.

5.1. Inclusion Criteria

Participants are eligible to be included in the study only if all of the following criteria apply:

1. A written informed consent must be obtained before any study-related assessments are performed.
2. Men and women between 18 and 80 years, inclusive; women of child-bearing potential (defined as those who are not post-menopausal or post-surgical sterilization; see Section 10.4 Appendix 4) must meet both of the following criteria:
   - Two negative pregnancy tests (at screening and at randomization, prior to dosing)
   - Use of intrauterine device, from at least 3 months before the baseline visit through at least 4 months after the last dose of bimagrumab/placebo i.v., and an additional contraceptive (barrier) method from screening through at least 4 months after the last dose of bimagrumab/placebo i.v.
3. Body mass index (BMI) ≥ 30 kg/m^2 or ≥ 27 kg/m^2 with one or more obesity-associated comorbidities (e.g., hypertension, insulin resistance, sleep apnea, or dyslipidemia).
4. Stable body weight (±5 kg) within 90 days of screening, and body weight <150 kg.
5. Have a history of at least one self-reported unsuccessful behavioral effort to lose body weight.
6. Capable of using common software applications on a mobile device (smartphone).
7. Access to an internet-enabled smartphone, tablet, or computer for the duration of the study, meeting minimal operations systems (OS) requirements.
8. Able to communicate well with the Investigator, comply with the study requirements and adhere to the diet and activity programs for the study duration.

5.2. Exclusion Criteria

Participants are excluded from the study if any of the following criteria apply:

1. History of, or known hypersensitivity to, monoclonal antibody drugs or a contraindication to semaglutide (Ozempic® or Wegovy®).
2. Use of other investigational drugs at the time of enrollment or within 30 days or 5 half-lives of enrollment, whichever is longer, or longer if required by local regulations.
3. Lack of peripheral venous access.

4. Are not able or willing to comply with protocol requirements including lifestyle interventions, for example:
    a. History of clinically significant condition that precludes regular walking for exercise e.g., cardiovascular or pulmonary disease or osteoarthritis.
    b. Contraindication to following a 500-calorie daily deficit, high protein diet.
5. Women who are pregnant or intend to become pregnant or are nursing.
6. Diseases known to cause cachexia or muscle atrophy, or diseases known to cause GI malabsorption (e.g., inflammatory bowel disease, celiac disease, short bowel syndrome, pancreatic insufficiency).
7. Use of any prescription drugs known to adversely affect muscle mass or body weight, including anti-androgens (e.g., gonadotropin hormone releasing hormone agonists, androgen receptor antagonists, glucocorticoids administered systemically for >2 weeks if dose is ≥10 mg of prednisone or equivalent within 12 weeks prior to Day 1 to EoS). Low dose estrogen replacement therapy in post-menopausal women is acceptable and 5-alpha reductase inhibitors in men are acceptable. Spironolactone and related drugs are acceptable in men and women.
8. Treatment with any medication for the indication of obesity within the past 30 days before screening.
9. Previous or planned (during the trial period) obesity treatment with surgery or a weight-loss device. However, the following are allowed: (1) liposuction and/or abdominoplasty, if performed >1 year before screening; (2) lap banding, if the band has been removed >1 year before screening; (3) intragastric balloon, if the balloon has been removed >1 year before screening; (4) duodenal-jejunal bypass sleeve, if the sleeve has been removed >1 year before screening or Plenity®, if discontinued at least 1 week prior to screening.
10. Uncontrolled thyroid disease, defined as thyroid stimulating hormone above or below the normal range, at screening or within 6 months prior to screening. Hypothyroid patients treated with thyroid hormone replacement therapy must be on a stable dose for at least 6 weeks prior to screening.
11. Diagnosis of diabetes, requiring current use of any antidiabetic drug or HbA1c ≥ 6.5%.

    Note: Metabolic syndrome is not an exclusion, even if managed with an anti-diabetic drug such as metformin or an SGLT2 inhibitor. A diagnosis of prediabetes or impaired glucose tolerance managed exclusively with non-pharmacologic approaches (e.g., diet and exercise) is not an exclusion.
12. History of malignancy of any organ system, treated or untreated within the past five years, regardless of whether there was evidence of local recurrence or metastases, except non-melanoma skin cancer treated only with local therapy. Specifically excluded are participants with multiple endocrine neoplasia type 2 (MEN2) or a personal or family history of medullary thyroid cancer (MTC) or known elevation of blood calcitonin >50 ng/L.

13. Known heart failure classified as New York Heart Association Class III and IV or a history of chronic hypotension, defined as systolic blood pressure <100 mmHg or diastolic blood pressure <50 mmHg. Uncontrolled hypertension, defined as systolic blood pressure >180 or diastolic blood pressure >100 mmHg at screening/baseline.
14. ECG showing clinically significant abnormalities including any current supra-ventricular arrhythmia with an uncontrolled ventricular response (mean heart rate >100 beats per minute [bpm]) at rest despite medical or device therapy, or any history of spontaneous or induced sustained ventricular tachycardia (heart rate >100 bpm for 30 sec) despite medical or device therapy, or any history of resuscitated cardiac arrest or presence of an automated internal cardioverter-defibrillator. Prolonged QT syndrome or QTcF > 450 msec (Fridericia Correction) for males and >470 msec for females at screening.
15. History of unstable angina, myocardial infarction, coronary artery bypass graft surgery, or percutaneous coronary intervention (such as angioplasty or stent placement) within 180 days of screening.
16. History or presence of significant coagulopathy e.g., PT/INR >1.5.
17. History of familial hypertriglyceridemia or history of fasting triglyceride > 500 mg/dL (5.65 mmol/L).
18. Known history or presence of severe acute or chronic liver disease (compensated or decompensated), known cholelithiasis or cholecystitis or bile duct disease, acute or chronic pancreatitis (or medication associated with severe pancreatitis, such as valproate), or severe GI dysmotility syndrome including functional disorders such as severe irritable bowel syndrome. Serum lipase >2x upper limit of normal (ULN) or serum amylase > 2x ULN at screening.
19. Liver injury as indicated by abnormal liver function tests such as AST, ALT, GGT, alkaline phosphatase, or serum bilirubin;
    - Any single transaminase >3x ULN.
    - Total bilirubin concentration increased above 1.5x ULN (except for cases of known Gilbert syndrome).
20. History or presence of substantially impaired renal function as indicated by eGFR < 45 mL/min/1.73 m^2 or serum creatinine ≥ 1.5x ULN or proteinuria > 2+ by urine dipstick or equivalent.
21. Total WBC <3000/µL, neutrophils <1500/µL, hemoglobin <8.5 g/dL, or platelet count <100,000/µL at screening.
22. Any chronic infections likely to interfere with study conduct or interpretation such as hepatitis B (HBV), hepatitis C (HCV), or human immunodeficiency virus (HIV). History of hepatitis A or hepatitis C successfully treated is not exclusionary. Active COVID-19 infection.
23. Donation or loss of 400 mL or more of blood within eight weeks prior to initial dosing, or longer if required by local regulation, or plasma donation (> 250 mL) within 14 days prior to the first dose.

24. Acute illness within the 30 days prior to screening that, in the opinion of the Investigator, affects the patient's ability to participate in the study.
25. Known or suspected abuse of alcohol or other substances including, but not limited to:
    a. Smoking more than one pack of cigarettes daily.
    b. Drinking 5 or more alcoholic beverages on each of 5 or more days in the past 30 days.
    c. Using cannabis more than twice weekly.
    d. Any use of heroin, cocaine, etc.
26. Any disorder, unwillingness, or inability not covered by any of the other exclusion criteria, which in the Investigator's opinion, might jeopardize the participant's safety or compliance with the protocol.

5.3. Lifestyle Considerations

5.3.1. Meals and Dietary Restrictions

Study participants will be counseled to follow a modest calorie restricted diet with a daily deficit of approximately 500 kcal. The following is a recommended, but not required, breakdown of daily calories: approximately 45-50% from carbohydrate, 20-25% from protein and 30% from fat intake throughout the study. Participants will be advised to consume at least 1.2 g/kg/body weight of protein as per guidelines for daily recommended protein intake for anabolism (WHO guidelines 2015).

Counselling will be conducted by a dietician or a similar qualified healthcare professional every 4 weeks during the study via visits/phone contacts to encourage dietary intake that will promote weight loss and document any change in dietary habits. During these dietician contacts, participants will be asked to recall all food and drink they have consumed in the previous 24 hours.

If BMI ≤ 22 kg/m^2 is reached during the study, the recommended daily energy intake may be recalculated to promote weight maintenance. If further, potentially undesirable weight loss continues, or if BMI is ≤ 18.5 kg/m^2, the investigator should contact the Sponsor to discuss whether it is medically appropriate for the participant to continue in the study. See SOM for more information related to assessing dietary intake and calculating nutrient needs.

Participants must fast for at least 8 hours prior to all visits that include blood sampling i.e., without food or liquids, except for water. Study medication and any medication which should be taken with or after a meal should be withheld on the day of the visit until blood samples have been obtained.

For visits where PD samples are collected for analysis by the central lab, if the participant is not fasting as required, the participant should be called in for a new visit as soon as possible to have the fasting procedures done. Procedures requiring participants to fast include blood sampling of fasting plasma glucose (FPG), fasting serum insulin and lipid assessments.

Participants should restrict alcohol consumption for the 24 hours prior to study visits.

5.3.2. Activity

Participants will be encouraged to engage in at least 150 minutes of physical activity per week e.g., walking or climbing stairs. The actigraphy device worn by participants will be used to collect activity data.

Additional details of the physical activity program and the actigraphy device can be found in the SOM.

5.3.3. Other Restrictions

Participants should refrain from smoking for one hour prior to study visits and during the study visits.

Women of childbearing potential must use an intrauterine device (IUD), from at least 3 months before the baseline visit through at least 4 months after the last dose of bimagrumab/placebo i.v., and use an additional contraceptive (barrier) method after the screening visit through at least 4 months after the last dose of bimagrumab/placebo. Additional information on highly effective contraception methods is provided in Section 10.4 Appendix 4.

5.3.4. Support During Treatment Withdrawal Period

During the treatment withdrawal period, participants will continue to have access to personnel who have experience in providing diet and exercise counseling. Options for additional resources to help lifestyle management will also be available to participants during this time.

5.4. Screen Failures

A screen failure occurs when a participant who has consented to participate in the clinical study is not subsequently assigned to study intervention, either due to a failure to meet the eligibility criteria or by choosing not to proceed to enrollment.

A minimal set of screen failure information is required to ensure transparent reporting of screen failure participants to meet the CONSORT publishing requirements and to respond to queries from regulatory authorities. Minimal information includes demography, screen failure details, eligibility criteria, and any SAE.

Individuals who do not meet the criteria for participation in this study may be rescreened once at the discretion of the Investigator. Rescreened participants should be assigned a new screening number for every screening/rescreening event. Individuals with laboratory assessments that are outside of the Week -6 to Day -1 window may be reassessed once within the same screening event (without requiring a new screening number).

6. STUDY INTERVENTION(S) AND CONCOMITANT THERAPY

Study interventions are all pre-specified, investigational, and non-investigational medicinal products, medical devices and other interventions (e.g., behavioral) intended to be administered to the study participants during the study conduct.

A summary of study interventions administered in this study is found in Table 2. Treatment arms are listed in Table 3.

The semaglutide dosing regimen employed in this study follows the guidance provided in the package insert for Wegovy®. However, due to Wegovy® supply constraints, Ozempic® pens will be used in place of Wegovy® pens as needed. Ozempic® pens contain the same active ingredients as Wegovy® pens.

Site staff will provide participants with self-administration training specific to the type of semaglutide pen dispensed.

Refer to the Ozempic® and Wegovy® package inserts for additional information and instructions on administration.

6.1. Study Interventions Administered

Table 2: Study Interventions Administered

Intervention Label	Low dose bimagrumab	High dose bimagrumab	Placebo (for bimagrumab)	Low dose semaglutide [a]	High dose semaglutide [a,b]
**Intervention Name**	Bimagrumab		Placebo	Semaglutide	
**Intervention Description**	Human monoclonal antibody to the activin receptor type II		Placebo	Glucagon-like peptide-1 (GLP-1) receptor agonist	
**Type**	Biologic		n/a	Biologic	
**Dose Formulation**	Liquid in a vial			Autoinjector Pen	
**Unit Dose Strength(s)**	Vial: 150 mg/1 mL concentrate for solution for infusion contains 1500 mg of bimagrumab in 10 mL (excluding 10% overfill)		n/a	0.25 mg, 0.5 mg, 1.0 mg	0.25 mg, 0.5 mg, 1.0 mg, 1.7 mg, and 2.4 mg
**Core Treatment Dosage Level(s)**	10 mg/kg at Weeks 1, 4, 16, 28, and 40	30 mg/kg at Weeks 1, 4, 16, 28 and 40	1 infusion at Weeks 1, 4, 16, 28 and 40	Weeks 1 to 4: 0.25 mg<br>Weeks 5 to 8: 0.5 mg<br>Weeks 9 to 48: 1.0 mg	Weeks 1 to 4: 0.25 mg<br>Weeks 5 to 8: 0.5 mg<br>Weeks 9 to 12: 1.0 mg<br>Weeks 13 to 16: 1.7 mg<br>Weeks 17 to 48: 2.4 mg
**Extension Treatment Dosage Level(s)**	10 mg//kg at Weeks 52 and 64	30 mg/kg at Weeks 52 and 64	[c]1 infusion at Weeks 52 and 64	Weeks 49 to 71: 1.0 mg	Weeks 49 to 71: 2.4 mg
**Route of Administration**	i.v.			s.c.	
**Use**	Experimental		Placebo	Active comparator	
**IMP and NIMP/AxMP**	IMP				
**Sourcing**	Provided centrally by the Sponsor				
**Packaging and Labeling**	Study intervention will be provided as 3 vials per box. Each box and vial will be labeled as required per country requirement.			Comparator will be provided as either a single pen (Ozempic®) or 4 pens per box (Wegovy®), depending on the dose to be administered. Each box and pen will be labeled as required per country requirement.	
**Current / Former Name(s) / Alias**	Bimagrumab			Semaglutide (Ozempic®/Wegovy®)	

[a] Participants who are unable to tolerate a weekly dose of 0.5 mg or higher may decrease in dose to the preceding dose level.

[b] Semaglutide doses of 1.7 mg and 2.4 mg may be substituted with 1.5 mg and 2.5 mg, respectively, based on availability of semaglutide pens.

[c] Placebo infusions to be discontinued after the Week 48 database lock and study unblinding.

Table 3: Study Treatment Arms

Arm Title	Arm Type	Arm Description	Associated Intervention Labels
Core: Bimagrumab placebo<br>Extension: 30 mg/kg bimagrumab	Core: Placebo<br>Extension: Experimental	**Core Treatment Period:** Participants will receive i.v. bimagrumab placebo at baseline, and at Weeks 4, 16, 28 and 40<br>**Extension Treatment Period:** Participants will receive 30 mg/kg i.v. bimagrumab at Weeks 52 and 64	Treatment Arm 1[a]
Bimagrumab placebo + 1.0 mg semaglutide	Placebo + Comparator	**All Treatment Periods:** Participants will receive i.v. bimagrumab placebo at baseline, and at Weeks 4, 16, 28, 40, 52, and 64, and 1.0 mg s.c. semaglutide weekly per the dose escalation schedule	Treatment Arm 2[a]
Bimagrumab placebo + 2.4 mg semaglutide	Placebo + Comparator	**All Treatment Periods:** Participants will receive i.v. bimagrumab placebo at baseline, and at Weeks 4, 16, 28, 40, 52, and 64, and 2.4 mg s.c. semaglutide weekly per the dose escalation schedule	Treatment Arm 3[a]
Core: 10 mg/kg bimagrumab<br>Extension: 30 mg/kg bimagrumab	Experimental	**Core Treatment Period:** Participants will receive 10 mg/kg i.v. bimagrumab at baseline, and at Weeks 4, 16, 28 and 40<br>**Extension Treatment Period:** Participants will receive 30 mg/kg i.v. bimagrumab at Weeks 52 and 64	Treatment Arm 4
10 mg/kg bimagrumab + 1.0 mg semaglutide	Experimental + Comparator	**All Treatment Periods:** Participants will receive 10 mg/kg i.v. bimagrumab at baseline, and at Weeks 4, 16, 28, 40, 52, and 64, and 1.0 mg s.c. semaglutide weekly per the dose escalation schedule	Treatment Arm 5
10 mg/kg bimagrumab + 2.4 mg semaglutide	Experimental + Comparator	**All Treatment Periods:** Participants will receive 10 mg/kg i.v. bimagrumab at baseline, and at Weeks 4, 16. 28, 40, 52, and 64, and 2.4 mg s.c. semaglutide weekly per the dose escalation schedule	Treatment Arm 6
30 mg/kg bimagrumab	Experimental	**All Treatment Periods:** Participants will receive 30 mg/kg i.v. bimagrumab at baseline, and at Weeks 4, 16, 28, 40, 52, and 64	Treatment Arm 7

Arm Title	Arm Type	Arm Description	Associated Intervention Labels
30 mg/kg bimagrumab + 1.0 mg semaglutide	Experimental + Comparator	**All Treatment Periods:** Participants will receive 30 mg/kg i.v. bimagrumab at baseline, and at Weeks 4, 16. 28, 40, 52, and 64, and 1.0 mg s.c. semaglutide weekly per the dose escalation schedule	Treatment Arm 8
30 mg/kg bimagrumab + 2.4 mg semaglutide	Experimental + Comparator	**All Treatment Periods:** Participants will receive 30 mg/kg i.v. bimagrumab at baseline, and at Weeks 4, 16, 28, 40, 52, and 64, and 2.4 mg s.c. semaglutide per the dose escalation schedule	Treatment Arm 9

[a]Placebo infusions to be discontinued after the 48-week database lock and study unblinding.

6.2. Preparation, Handling, Storage, and Accountability

The Investigator or designee must confirm appropriate conditions (e.g., temperature) have been maintained during transit for all study intervention received, and any discrepancies reported and resolved before use of the study intervention.

Only participants enrolled in the study may receive study intervention, and only authorized site staff may supply, prepare, or administer study intervention.

All study intervention must be stored in a secure, environmentally controlled, and monitored (manual or automated) area in accordance with the labeled storage conditions with access limited to the Investigator and authorized site staff.

The Investigator and study pharmacist are responsible for study intervention accountability, reconciliation, and record maintenance (i.e., receipt, reconciliation, and final disposition records).

Further guidance and information for the final disposition of unused study interventions are provided in the pharmacy manual.

6.3. Assignment to Study Intervention

Eligible participants will be randomly assigned to one of nine treatment arms using a centralized interactive web randomization system (IWRS). Before the study is initiated, directions for the log-in information will be provided to each site.

Randomization will be balanced in a 1:1:1:1:1:1:1:1:1 ratio with approximately 55 participants per treatment arm. Stratification across the treatment arms will be based on gender.

The randomization schedule will be generated as a randomized block design using SAS version 9.4 or higher.

The randomization number becomes definitive once a participant has been dosed and it may not be reassigned.

6.4. Blinding

The study is triple blinded to the study participant, Investigator and Sponsor with respect to bimagrumab and bimagrumab placebo and open label with respect to semaglutide. Blinding will remain intact at least until database is locked for the Week 48 primary analysis. Additional details regarding blinding and unblinding will be described in the SAP and a separate blinding and unblinding plan.

6.4.1. Bimagrumab and Placebo

All treatment arms are triple blinded with regards to bimagrumab and bimagrumab placebo (participants/Investigators, Sponsor). The IWRS will be programmed with blind-breaking instructions. In case of an emergency, the Investigator has the sole responsibility for determining if unblinding of a participants' intervention assignment is warranted. Participant safety must always be the first consideration in making such a determination. If the Investigator decides that unblinding is warranted, the Investigator may, at the Investigator's discretion, contact the Sponsor to discuss the situation prior to unblinding a participant's intervention assignment unless this could delay emergency treatment for the participant. If a participant's intervention assignment is unblinded, the Sponsor must be notified within 24 hours of this occurrence. The date and reason for the unblinding must be recorded.

6.4.2. Semaglutide

Because a placebo of semaglutide is not available for this study, the study cannot be double blinded to all semaglutide treatments. Therefore, participants assigned to a combination of semaglutide with bimagrumab or its placebo (approximately N=330) will be aware of their open label semaglutide dosage but blinded to the bimagrumab/placebo assignment and dosage.

Potential bias will be reduced through central randomization.

6.4.3. SAE and Unblinding

Sponsor safety staff may unblind the intervention assignment for any participant with an SAE. If the SAE requires that an expedited regulatory report be sent to one or more regulatory agencies, a copy of the report, identifying the participant's intervention assignment, may be sent to Investigators in accordance with local regulations and/or Sponsor policy.

6.4.4. Unblinded Personnel

With the exception of any unblinded site staff identified below, all site staff (including study Investigator and study nurse) will be blinded to study treatment throughout the study.

Unblinded study personal will include:

- Unblinded site pharmacist or pharmacy staff
- Unblinded study monitor

Bimagrumab and its placebo will be supplied as bulk in boxes containing 3 vials per box. An unblinded pharmacist or other qualified trained personnel, who is independent of the study team, will be required to prepare study drug bags for infusion. They are the only site level personnel who will have access to treatment assignments.

Appropriate measures must be taken by the unblinded pharmacist to ensure that the treatment assignments are concealed from the rest of the site staff. Any potential visible difference in treatments will be concealed by the use of an opaque sleeve.

6.5. Study Intervention Compliance

When the individual dose for a participant is prepared, the preparation of the dose will be confirmed by another unblinded study staff member.

When participants are dosed at the site, they will receive study intervention directly from the Investigator or designee, under medical supervision. The date and time of each dose administered in the clinic will be recorded in the source documents. The dose of study intervention and study participant identification will be confirmed at the time of dosing by a member of the study site staff other than the person administering the study intervention.

In treatment arms receiving semaglutide, when participants self-administer semaglutide at home, compliance with study intervention will be assessed at each visit. Compliance will be assessed by direct questioning, checking of participant diary, and return of all used and unused medication during the site visits and documented in the source documents and relevant form. Deviation(s) from the prescribed dosage regimen should be recorded.

A record of the quantity of study interventions dispensed to and administered to/by each participant must be maintained and reconciled with study intervention and compliance records. Intervention start and stop dates, including dates for intervention delays and/or dose reductions will also be recorded.

6.6. Dose Modification

6.6.1. Bimagrumab

No dose modification (up or down titration) of bimagrumab is allowed. Interruption of dosing may be allowed for safety reasons (see Section 7.1.1).

6.6.2. Semaglutide

Semaglutide is initiated at 0.25 mg once weekly for 4 weeks. Dose is increased in 4-week intervals until a dose of 2.4 mg is reached for the 2.4 mg dose arms and until 1.0 mg is reached for the 1.0 mg dose arms.

If participants do not tolerate the maintenance 2.4 mg once-weekly dose, the dose can be temporarily decreased to 1.7 mg once-weekly, for a maximum of 4 weeks. After 4 weeks, semaglutide should be increased to the maintenance dose of 2.4 mg once weekly.

If participants do not tolerate the planned dose-escalation schedule, they may drop back to the highest previously tolerated dose and extend that dose for an additional one week, and then re-attempt dose escalation at least once. If the higher dose is not tolerated, the participant can step down and remain on the lower, highest-tolerated dose.

Management of Participants with BMI ≤22 kg/m²

If …	And..	Then …
a participant has a BMI ≤22 kg/m² at any time during the study	1) the participant has completed dose escalation with semaglutide<br>2) dietary modifications to slow weight loss have been attempted	the investigator should contact the sponsor to consider a dose reduction of semaglutide to the next lower maintenance dose level.
body weight reduction continues to decline after 30 days despite a dose reduction to the next lower maintenance dose level	the investigator wants to consider discontinuing study intervention	the investigator should contact the sponsor for further discussion.
a participant has a BMI ≤18.5		study intervention must be discontinued.

Management of Participants with Perceived Excessive Body Weight Loss

If BMI is not ≤22 kg/m² but there are concerns that the degree of body weight reduction may lead to participant decision to discontinue from study intervention, the investigator should contact the sponsor to consider dose reduction to the next lower maintenance dose level.

6.7. Continued Access to Study Intervention after the End of Treatment

No access to study medication will be provided after completion of the study treatment period or post-treatment follow-up period.

6.8. Treatment of Overdose

6.8.1. Bimagrumab

The Sponsor does not recommend specific treatment for an overdose of bimagrumab.

6.8.2. Semaglutide

Overdose of semaglutide should follow the standard directions as outlined in the package insert.

6.9. Prior and Concomitant Therapy

Any medication or vaccine (including over the counter or prescription medicines, recreational drugs, vitamins, and/or herbal supplements) that the participant is receiving at the time of enrolment or receives during the study must be recorded along with:

- Reason for use
- Dates of administration including start and end dates
- Dosage information including dose and frequency

The medical monitor should be contacted if there are any questions regarding concomitant or prior therapy.

6.9.1. Prohibited Medicine

The table below lists medications which are prohibited from use for the study period.

Table 4: Prohibited Medications

Prohibited Medication	Prohibition Period	Action
Anti-androgens	12 weeks prior to Day 1 to EoS	Report protocol deviation, if used >7 days discontinue the study
Gonadotropin releasing hormone (GnRH) analogues	12 weeks prior to Day 1 to EoS	Report protocol deviation, if used >7 days discontinue the study
Anabolic steroids (testosterone, danazol, oxandrolone, stanozolol etc.) except replacement doses in hypogonadal men.<br>Hormone replacement therapy in women with the exception of low dose estrogen.	12 weeks prior to Day 1 to EoS	Report protocol deviation, if used >7 days discontinue the study
Recombinant human growth hormone (rhGH), hGH receptor antagonist (e.g., pegvisomant), hGH releasing hormone agonist	12 weeks prior to Day 1 to EoS	Report protocol deviation, if used >7 days discontinue the study
Oral beta-adrenergic agonists	4 weeks prior to Day 1 to EoS	Report protocol deviation, if used >14 days discontinue the study
Systemic glucocorticoid (dexamethasone, hydrocortisone, methyl prednisolone, prednisolone, prednisone, triamcinolone) at doses > 10 mg/d prednisone-equivalent	12 weeks prior to Day 1 to EoS	Short term courses of systemic glucocorticoid treatment up to 14 days is allowed (maximum, 2 courses per year). Report protocol deviation, if used >14 days; discontinue the study if >2 courses of short-term steroid.
Anti-obesity medications, dietary supplements, dietary interventions other than described in the protocol, or over the counter products for weight loss.	30 days prior to Day 1 to EoS	Report protocol deviation, if used >30 days discontinue the study
Any antidiabetic medication used to treat diabetes. Note: Metformin or SGLT2 inhibitors are permitted if used for the treatment of metabolic syndrome. Insulin is allowed for up to one week at a time to correct for acute hyperglycemia.	12 weeks prior to Day 1 to EoS	Report protocol deviation, if used >7 days discontinue the study
Any product or medication known or suspected to cause significant weight change (e.g., mirtazapine), in consultation with the medical monitor), unless on stable dose for at least 12 weeks prior to Day 1.	12 weeks prior to Day 1 to EoS	Report protocol deviation (unless specific medication allowed by medical monitor); if used >30 days, discontinue the study

Prohibited Medication	Prohibition Period	Action
Valproate	12 weeks prior to Day 1 to EoS	Report protocol deviation, if used >7 days discontinue the study

6.9.2. Prohibited Non-Pharmacological Concomitant Treatments

Bariatric surgery and use of Plenity® are prohibited for the study period.

6.9.3. Recommended Treatment of Adverse Events

Muscle symptoms can be addressed with light self-massage of the involved area concentrating on improving circulation and relaxing muscle tissue. If excessive or prolonged soreness presents, then acetaminophen can be used as needed.

Acne may be treated with a face wash containing 4-10% benzoyl peroxide and over the counter topical treatments. If needed, a prescription topical treatment (e.g., antibiotic) or oral antibiotic (e.g., minocycline 100 mg bid) may be recommended at the Investigator's discretion. Early intervention is recommended when acne presents.

Diarrhea, nausea, vomiting, constipation may be treated with over-the-counter remedies. In cases of prolonged occurrence, other treatments may be used at the Investigator's discretion.

7. DISCONTINUATION OF STUDY INTERVENTION, PARTICIPANT DISCONTINUATION / WITHDRAWAL AND STUDY STOPPING / PAUSING

7.1. Participant Discontinuation of Study Intervention

In rare instances, it may be necessary for a participant to permanently discontinue study intervention. If study intervention is permanently discontinued during one of the treatment periods, the End of Treatment (EOT) visit should be conducted as soon as possible, followed by the End of Study (EOS) visit at least 12 weeks after last treatment with bimagrumab/placebo.

Study treatment *must* be discontinued for an individual participant under the following circumstances:

- Withdrawal of consent.
- An infusion reaction that is considered severe.
- One or more symptomatic hypoglycemic events necessitating third party rescue and suspected to be related to study drug.
- A serious adverse event (SAE) thought to be related to study drug.
- Use of prohibited treatment fulfilling the criteria in Table 4.

- Pregnancy: a positive pregnancy test after start of study treatment requires immediate discontinuation of study treatment, even if confirmation testing is pending. In the event of pregnancy, report as described above.
- Any protocol deviation that results in a significant risk to the participant's safety.
- Emergence of one or more adverse events that in the judgment of the Investigator, considering the participant's overall status, prevent the participant from safely continuing in thestudy.

Investigational treatment *may* be discontinued under the following circumstances:

- Breaking of the blind (inadvertently or for emergency reasons)
- Liver, pancreas, or muscle related adverse event as described in Sections 10.6, 10.7, and 10.8.

Participants who discontinue study treatment should **NOT** be considered withdrawn from the study UNLESS they withdraw their consent. They should return approximately 12 weeks after their last dose of bimagrumab/placebo for the End of Study visit as described above. If they fail to return for these assessments for unknown reasons, every effort should be made to contact them as specified in Section 7.3.

7.1.1. Liver, Pancreas, or Muscle Related Events Stopping Criteria

Study treatment may be interrupted or discontinued if a participant meets one of the conditions for liver, pancreas or muscle related adverse events, as outlined in the algorithms in Sections 10.6, 10.7, and 10.8, Appendices 6, 7, and 8, or if the Investigator believes it is in the best interest of the participant, even if the criteria are not met. At the Week 4 and Week 64 visits, participants' safety lab results must be available and reviewed against the guidance in Appendices 6, 7, and 8 in advance of dose administration with bimagrumab/placebo.

7.1.2. Temporary Discontinuation

No temporary discontinuation is allowed except for management of adverse events, as described in Section 7.1.1. Refer to Section 6.6.2 if the current semaglutide dose cannot be tolerated, and for management of participants with BMI ≤22 kg/m^2 or perceived excessive body weight loss.

7.2. Participant Discontinuation/Withdrawal from the Study

A participant may withdraw from the study at any time at the participant's own request for any reason (or without providing any reason).

A participant may be withdrawn at any time at the discretion of the Investigator for safety or compliance reasons.

At the time of discontinuing from the study, if during one of the treatment periods, an "end of treatment" visit should be conducted as soon as possible, as described in Section 7.1. Please see SoA Section 1.3 for data to be collected at the time of study discontinuation and follow-up for

any further evaluations needed for follow up of adverse events. Participants should return at least 12 weeks after their last dose of bimagrumab/placebo for the End of Study visit.

If the participant withdraws consent for disclosure of future information, the Sponsor may retain and continue to use any data collected before such a withdrawal of consent.

If a participant withdraws from the study, the participant may request destruction of any samples taken and not tested, and the Investigator must document this in the site study records.

7.3. Lost to Follow-up

A participant will be considered lost to follow-up if the participant repeatedly fails to return for scheduled visits and is unable to be contacted by the study site.

The following actions must be taken if a participant fails to return to the clinic for a required study visit:

- The site must attempt to contact the participant and reschedule the missed visit as soon as possible, counsel the participant on the importance of maintaining the assigned visit schedule and ascertain whether the participant wishes to and/or should continue in the study.
- Before a participant is deemed lost to follow-up, the Investigator or designee must make every effort to regain contact with the participant (where possible, 3 telephone calls, and if necessary, a certified letter to the participant's last known mailing address or local equivalent methods). These contact attempts should be documented in the participant's medical record.
- Should the participant continue to be unreachable, the participant will be considered "lost to follow-up".

7.4. Treatment Arm and Study Stopping/Pausing Rules

The study will be paused and no further dosing and/or new recruitment will occur pending full safety review by the DMC, if any of the below criteria are met. After a safety review by the DMC, the DMC will recommend to the sponsor either to continue the study, to stop an arm or to stop the study. The study may resume following the safety review, if the DMC and Sponsor agree it is safe to proceed and necessary approvals have been obtained from authorities according to local regulations.

- Any death considered to be related to study treatment.
- Two or more participants experience a TESAEs of a similar type (other than death, corresponding to NCI-CTCAE grade 3 or higher) considered to be related to blinded study treatment, unless expected per Reference Safety Information.
- One or more participants develop a life-threatening (NCI-CTCAE grade 4) or fatal acute allergic reaction within 24 hours following study treatment administration (i.e., active drug/placebo), unless clearly caused by exposure to a known allergen (e.g., peanut allergy).

- Two or more participants are discontinued due to a liver event as defined in Section 10.6 Appendix 6.
- Number and/or severity of AEs, abnormal safety monitoring tests, or abnormal laboratory findings justify putting the study on hold.
- The Sponsor unilaterally requests a stoppage.

8. STUDY ASSESSMENTS AND PROCEDURES

Study procedures and their timing are summarized in the SoA Section 1.3. Protocol waivers or exemptions are not allowed. Visits should be performed as close to the planned timepoint as possible, with a visit window of +/- 7 days, with the following exceptions:

- All required Screening assessments must be completed within Week -6 and Day -1. Any screening assessments that are outside of this range may be repeated once without screen failing.
- For the baseline visit, DXA scans may be performed up to 14 days prior to the baseline visit. For all other visits, the DXA assessment window is -2 weeks to +1 week of the scheduled time.
- Visit 4 (Week 4) and Visit 19 (Week 64) dosing should be performed within a visit window of -2 to +14 days.

Adherence to the study design requirements, including those specified in the SoA Section 1.3, is essential and required for study conduct.

All screening evaluations must be completed and reviewed to confirm that potential participants meet all eligibility criteria. The Investigator will maintain a screening log to record details of all participants screened and to confirm eligibility or record reasons for screening failure, as applicable.

Procedures conducted as part of the participant's routine clinical management (e.g., complete blood count) and obtained before signing of the ICF may be utilized for screening or baseline purposes provided the procedures met the protocol-specified criteria and were performed within the timeframe defined in the SoA Section 1.3.

In the event of a significant study-continuity issue (e.g., caused by a pandemic), alternate strategies for participant visits, assessments, medication distribution and monitoring may be implemented by the Sponsor or the Investigator, as per local health authority/ethics requirements.

Repeat or unscheduled samples may be taken for safety reasons or for technical issues with the samples.

8.1. Administrative Procedures

Demographic and baseline characteristic data will include year of birth, sex, race, predominant ethnicity.

Relevant medical history and current medical conditions will be recorded. Where possible, diagnoses but not symptoms will be recorded. Investigators have the discretion to record

abnormal test findings as medical history, if in their judgment, the test abnormality occurred prior to the informed consent signature.

Disease history will include duration of obesity/overweight (number of years). Obesity-related comorbidities will be recorded on the Medical History form, along with assessment of status of these at end of study.

All current medications and significant non-drug therapies must be listed on the Concomitant medications eCRF.

Urine drug screening will be performed, and the results kept as source data.

Body height will be measured as described in the SOM.

8.2. Efficacy / Pharmacodynamics Assessments

Planned timepoints for all efficacy and pharmacodynamic assessments are provided in the SoA Section 1.3. Additional details of the methods to perform assessments are provided in the SOM.

8.2.1. Anthropometric Measurements

8.2.1.1. Body Weight

Body weight will be measured in kilograms (kg) to the nearest 0.1 kg, in light indoor clothing, without shoes and on an empty bladder, using the scale provided by the Sponsor throughout the trial.

8.2.1.2. Waist Circumference

Waist circumference will be measured in standing position with a non-stretchable measuring tape and to the nearest 0.1 centimeter (cm). The measurement should be taken with the participant standing and with the waist area unclothed. The tape should be positioned around the participant, parallel to the floor, with the lower edge of the tape at the level of the posterolateral superior iliac crest bilaterally and the circumference measured at the end of a normal expiration.

The same type of measuring tape should be used throughout the trial. The measuring tape will be provided by the Sponsor to ensure standardization.

8.2.1.3. Body Mass Index

BMI will be calculated in the electronic case report form (eCRF) using the following formula:

$$\text{BMI} = \text{Body weight (kg)} / [\text{Height (m)}]^2$$

8.2.1.4. Bioelectrical Impedance Analysis

Bioelectrical impedance analysis (BIA) is a widely used method for estimating body composition. The technology is relatively simple, quick, and noninvasive. BIA is currently used in diverse settings, including private clinicians' offices, health clubs, and hospitals, and across a spectrum of ages, body weights, and disease states. An estimate of total body water (TBW) is derived by measuring electrical impedance of body tissues, from which fat-free mass (FFM), lean (muscle) mass, and body fat (adiposity) are estimated.

8.2.2. DXA Scan

Dual energy X-ray absorptiometry (DXA) will be used to assess changes in body composition, including total fat and lean body mass (FBM and LBM), trunk fat mass, visceral adipose tissue (VAT), subcutaneous adipose tissue (SAT), and bone mineral density (total body, lumbar spine, hip).

Quality assurance is an important issue in the use of DXA scans to determine body composition. DXA instrument manufacturer and model should remain consistent, and their calibration should be monitored throughout the study. Use of a standardized scan acquisition protocol and appropriate and unchanging scan acquisition and analysis software is essential to achieve consistent results. Likewise, because of variability in interpretation of the scans, it is important to utilize centralized scan analysis by experienced staff.

Data collection and processing is explained in the Imaging Manual written and provided by the imaging CRO. DXA scans will be sent to the imaging CRO for central reading. Results must remain blinded to Investigator and participant until database lock, however medically significant incidental findings (e.g., tumor) not related to the study analyses can be disclosed to the Investigator as appropriate for the medical care of the participant.

8.2.3. Grip Strength

Handgrip strength, as measured by dynamometry (kg), is used as a marker of overall muscle strength. Grip strength will be assessed using the dominant hand and the same hand should be consistently tested in all follow-up measurements.

8.2.4. Impact of Weight on Quality of Life-Lite Clinical Trials Version

The IWQOL-Lite CT is a 20-item modified survey instrument that is used to quantitatively assess an individual’s perception of how their weight affects their day-to-day life [34]. The LITE CT version has been specifically developed for clinical trials. The physical function score will also be evaluated. This instrument is especially valuable to validate the effectiveness of the treatment for obesity using metrics that go beyond the physical measurements of weight loss.

8.2.5. Short Form (SF-36) Health Survey

SF-36 measures a participant’s overall health related quality of life. It is a 36-item generic measure of health status that yields 2 summary scores for physical health and mental health, and 8 domain scores [35]. The physical functioning score will also be evaluated.

8.2.6. Physical Activity Monitoring Via Actigraphy

The Investigator must encourage American Diabetes Association walking guidelines (at least 150 minutes of physical activity per week). Daily steps and general activity levels will be monitored by a wrist worn actigraphy device provided to participants by the Sponsor.

8.2.6.1. Lipid Profile

Blood samples for total cholesterol, HDL cholesterol, LDL cholesterol, and triglycerides will be collected under fasting conditions, unless specified otherwise. These parameters will be

measured by the local lab at screening and by the central laboratory at Sponsor determined timepoints.

8.2.6.2. Glucose Metabolism Parameters: HbA1c, Fasting Insulin and Glucose

With the exception of the screening visit, samples for insulin and glucose will be collected under fasting conditions. Glucose will be measured by the local lab for safety. HbA1c and fasting insulin will be measured by the central laboratory, per the study's Laboratory Manual, at timepoints indicated in the SoA Section 1.3.

The Homeostatic model assessment 2 – insulin resistance index (HOMA-IR) and the quantitative insulin-sensitivity check index (QUICKI) are derived insulin resistance indexes that will be calculated by data management.

8.3. Safety Assessments

Planned timepoints for all safety assessments are provided in the SoA Section 1.3. Details of the methods to perform assessments are provided in the SOM.

8.3.1. Physical Examinations

A complete physical examination will be performed at all listed visits and will include the examination of general appearance, skin, neck (including thyroid), eyes, ears, nose, throat, lungs, heart, abdomen, back, lymph nodes, extremities, vascular, and neurological systems. If indicated based on medical history and/or symptoms, additional exams may be performed.

Skin assessment should include findings of new, or worsening, and significant acne or skin lesions.

For males, a bilateral assessment and measurement of gynecomastia (breast tissue diameter) will be made, if present.

Investigators should pay special attention to clinical signs related to previous serious illnesses and to those listed under "Warnings and precautions" for Wegovy® or Ozempic®.

8.3.2. Vital Signs

Temperature, pulse rate, and blood pressure will be recorded (before blood collection for laboratory tests).

Blood pressure and pulse rate measurements will be assessed in a sitting position with a completely automated device. Manual techniques will be used only if an automated device is not available or not suitable for the participant.

Blood pressure and pulse rate measurements should be preceded by at least 5 minutes of rest for the participant in a quiet setting without distractions (e.g., television, cell phones).

If a blood pressure measurement is out of range, it may be repeated once, after the participant has rested for at least 5 minutes.

8.3.3. Electrocardiograms

A standard 12-lead ECG will be performed with the participant in a supine position, using an ECG machine that automatically calculates the heart rate and measures PR, QRS, QT, and QTcF intervals. Interpretation of the tracing must be made by a qualified physician and documented on the ECG and in the ECG section of the eCRF.

Each ECG tracing should be labeled with the study number, participant initials, participant number and date, and kept in the source documents at the study site. Clinically significant abnormalities should be recorded on the relevant medical history eCRF page prior to informed consent signature and on the Adverse Events page thereafter. Clinically significant findings must be discussed with the Sponsor.

The eCRF will contain the date and time of ECG, PR and RR intervals, QT interval, QTcF, and QRS duration.

The Fridericia QT correction formula (QTcF) should be used for clinical decisions.

Unless auto calculated by the ECG machine, the Investigator must calculate QTcF. See the Site Operations Manual for additional details.

Original ECG tracings, appropriately signed, will be archived at study site.

8.3.4. Clinical Safety Laboratory Tests

All protocol-required safety lab tests, as defined in Section 10.2 Appendix 2, must be conducted in accordance with the SoA Section 1.3.

The Investigator must review the laboratory results, document this review, and record any clinically significant changes occurring during the study as an AE.

Abnormal laboratory findings associated with the underlying disease are not considered clinically significant unless judged by the Investigator to be more severe than expected for the participant's condition.

All laboratory tests with values considered clinically significantly abnormal during participation in the study or until 30 days from the EOS visit should be repeated until the values return to normal or baseline or are no longer considered clinically significant by the Investigator or medical monitor. Additional guidance for the follow up of abnormal liver and pancreas tests, as well as elevated CK, is provided in the safety monitoring section of this protocol and Sections 10.6, 10.7, and 10.8, Appendices 6, 7, and 8.

If clinically significant values do not return to normal/baseline within a period judged reasonable by the Investigator, the etiology should be identified, and the Sponsor notified.

If laboratory values from non-protocol-specified laboratory tests performed at the institution's local laboratory require a change in participant management or are considered clinically significant by the Investigator (e.g., SAE or AE or dose modification), then the results must be recorded.

8.3.5. Pregnancy Testing

Serum pregnancy test is required for all female participants at screening and for confirmation of any positive urine pregnancy test.

For all other visits, urine dipstick pregnancy testing will be required for pre-menopausal female participants, unless serum is required based on local requirements. Women surgically sterilized (but pre-menopausal) are still required to complete pregnancy tests. In case urine dipstick pregnancy test is positive, it must be confirmed with a serum pregnancy test prior to dosing. All positive urine tests will be followed up by a serum test.

A negative test result is required prior to receiving any study medication.

Additional serum or urine pregnancy tests may be performed, as determined necessary by the Investigator, or required by local regulation, to establish the absence of pregnancy at any time during the participant's participation in the study.

8.3.6. Hematology/Coagulation

Hemoglobin, hematocrit, red blood cell count, RBC indices, white blood cell count with differential (e.g., neutrophils, basophils, eosinophils, monocytes, lymphocytes), erythrocyte sedimentation rate, platelet count, aPTT, and PT/INR will be measured.

8.3.7. Blood Chemistry

Blood chemistry should be assessed after a fast of approximately 8 hours. Blood chemistry will include sodium, potassium, calcium, magnesium, bicarbonate/CO_2, chloride, phosphate or phosphorus, creatinine, BUN or urea, glucose, uric acid, albumin, total protein, alkaline phosphatase, total and direct bilirubin, LDH, GGT, AST, ALT, amylase, lipase, and CK.

If the total bilirubin concentration is increased above 1.5x ULN, direct and indirect bilirubin should be obtained.

Additional safety labs may be performed at the Investigator's discretion for any clinically/medically significant abnormalities.

8.3.8. Urinalysis and Urine Chemistry

A midstream urine sample (approx. 30 mL) will be obtained in order to avoid contamination with epithelial cells and sediments and to allow proper assessment. Required assessments include occult blood, pH, specific gravity, ketones, glucose, protein, bilirubin, nitrite and leukocytes. A microscopic examination will be performed if blood or protein are abnormal. The sample will also be used for measurement of urine microalbumin and urine creatinine, for calculation of urine albumin:creatinine ratio (ACR).

8.4. Adverse Events, Treatment Emergent Adverse Events, Serious Adverse Events, and Other Safety Reporting

The definitions of AEs, TEAEs, and SAEs unsolicited and solicited adverse events can be found in Section 10.3 Appendix 3.

The Investigator and any qualified designees are responsible for detecting, documenting, and recording events that meet the definition of an AE or SAE and remain responsible for following up all AEs. This includes events reported by the participant (or, when appropriate, by a caregiver, surrogate, or the participant's legally authorized representative).

The method of recording, evaluating, and assessing causality of AEs and SAEs and the procedures for completing and transmitting SAE reports are provided in Section 10.3 Appendix 3.

8.4.1. Time Period and Frequency for Collecting AE, TEAE and SAE Information

All SAEs will be collected from the time of the signature of the informed consent until the EoS visit and will be recorded in the database for all randomized participants. Non-serious adverse events will be collected from the time of first dose of study medication(s) until the EoS visit (treatment-emergent adverse events [TEAEs]). Non-serious medical conditions identified during the screening period should be recorded as medical history.

The Investigator will record and report all SAEs to the Sponsor or designee immediately and under no circumstance should this exceed 24 hours, as indicated in Section 10.3 Appendix 3. The Investigator will submit any updated SAE data to the Sponsor within 24 hours of it being available.

Investigators are not obligated to actively seek information on AEs or SAEs after conclusion of the study participation. However, if the Investigator learns of any SAE, including a death, at any time after a participant has been discharged from the study, and the Investigator considers the event to be reasonably related to the study intervention or study participation, the Investigator must promptly notify the Sponsor.

8.4.2. Follow-up of AEs and SAEs

After the initial AE/SAE report, the Investigator is required to proactively follow each participant at subsequent visits/contacts. All SAEs and AEs of special interest (as defined in Section 8.4.5) will be followed until resolution, stabilization, the event is otherwise explained, or the participant is lost to follow-up (as defined in Section 7.3). Further information on follow-up procedures is provided in Section 10.3 Appendix 3.

8.4.3. Regulatory Reporting Requirements for SAEs

Prompt notification by the Investigator to the Sponsor of an SAE is essential so that legal obligations and ethical responsibilities towards the safety of participants and the safety of a study intervention under clinical investigation are met.

The Sponsor has a legal responsibility to notify both the local regulatory authority and other regulatory agencies about the safety of a study intervention under clinical investigation. The Sponsor will comply with country-specific regulatory requirements relating to safety reporting to the regulatory authority, institutional review boards (IRBs)/independent ethics committees (IECs), and Investigators.

An Investigator who receives an Investigator Notification/Safety Report describing an SAE or other specific safety information (e.g., summary or listing of SAEs) from the Sponsor will

review and then file it along with the IB/package insert and will notify the IRB/IEC, if appropriate according to local requirements.

Investigator Notification/Safety Reports must be prepared for suspected unexpected serious adverse reactions (SUSARs) according to local regulatory requirements and Sponsor policy and forwarded to all participating Investigators.

8.4.4. Pregnancy

Details of all pregnancies in female participants, or in female partners of male participants, occurring in the study and until 4 months after the last dose of study drug (if the participant was on active drug) will be collected to determine the outcome of the pregnancy. The Investigator will collect the follow-up information on the participant and the neonate and forward the information to the Sponsor.

If a pregnancy is reported or detected, the Investigator will record pregnancy information on the appropriate form and submit it to the Sponsor within 24 hours of learning of the pregnancy.

While pregnancy itself is not considered to be an AE or SAE, any pregnancy complication or elective termination of a pregnancy for medical reasons will be reported as an AE or SAE.

Abnormal pregnancy outcomes (e.g., spontaneous abortion, fetal death, stillbirth, congenital anomalies, ectopic pregnancy) are considered SAEs and will be reported as such.

Any post-study pregnancy-related SAE considered reasonably related to the study intervention by the Investigator will be reported to the Sponsor as described in Section 8.4.4. While the Investigator is not obligated to actively seek this information in former study participants, he or she may learn of an SAE through spontaneous reporting.

Any female participant who becomes pregnant or has a positive pregnancy test while participating in the study must discontinue study intervention immediately.

8.4.5. Treatment Emergent Adverse Events of Special Interest

Adverse events of special interest (AESI) for bimagrumab are defined based on an ongoing review of all safety data and include (using CTCAE intensity grading):

- Muscle related events (e.g., rhabdomyolysis, muscle spasm, cramps, twitching, tightness) [at least grade 3]
- Skin lesions (e.g., acne, growths) [at least grade 3]
- Pancreatitis
- Gastrointestinal events (e.g., diarrhea, nausea) [at least grade 3]
- Malignancy

8.5. Pharmacokinetics

Serum bimagrumab and plasma semaglutide concentrations (as applicable) will be evaluated in samples collected from all participants according to the SoA Section 1.3.

Each serum/plasma sample will be divided into 2 aliquots (1 for analysis and another for backup). Samples collected for analyses of bimagrumab concentration and semaglutide concentration may also be used to evaluate safety or efficacy aspects related to concerns arising during or after the study.

Instructions for the collection and handling of biological samples will be provided in the laboratory manual. Post-bimagrumab samples should be targeted for collection 15 minutes after the end of the post-infusion flush, but no later than 45 minutes after the completion of the flush.

Bimagrumab and serum concentration information will not be reported to investigative sites or blinded personnel until the study has been unblinded.

Information on storage and future use of samples is detailed in Section 10.1.5.1.

8.6. Pharmacodynamics

8.6.1. PD Biomarkers

PD Biomarkers may include, but are not limited to, those listed below. Samples will be collected according to the schedule described in the SoA Section 1.3 and measured by the central lab.

- Adipokines: leptin, adiponectin, adipsin
- Fasting lipid profile: TC, HDL, LDL and TG
- Inflammation biomarkers: IL-6, IL- 18, hsCRP, NGAL
- Endocrine biomarkers: FSH, estradiol, free testosterone, insulin, HbA1c, serum CTX, BSAP, P1NP
- TGF-β superfamily biomarkers: activins, GDFs (specific tests to be determined)
- Safety biomarkers: ALP/amylase/ lipase isoenzymes, 5'NT, miR-122 and CCK18

Analysis of ALP/amylase/lipase isozymes and miR-122/CCK18 will only be performed for participants with abnormal local lab results.

Instructions for the collection and handling of biological samples will be provided in the laboratory manual.

Results of the above assessments are solely for research purposes and may not be reported to investigative sites or blinded personnel until after the study has been unblinded.

Samples collected may be used to evaluate other PD, safety, or efficacy aspects of concern arising during or after the study. Information on storage and future use of samples is detailed in Section 10.1.5.1.

8.7. Genetics

A 10 mL blood sample for DNA isolation will be collected at the baseline visit from participants who have consented to participate in the genetic analysis component of the study. Participation is optional. Participants who do not wish to participate in genetic research may still participate in the study.

In the event of DNA extraction failure, a replacement genetic blood sample may be requested from the participant. Signed informed consent will be required to obtain a replacement sample unless it was included in the original consent.

Instructions for the collection and handling of biological samples will be provided in the laboratory manual.

See Section 10.5 Appendix 5 Genetics for information regarding genetic research.

Information on storage and future use of samples is detailed in Section 10.1.5.1.

8.8. Immunogenicity Assessments

Serum samples from all participants collected according to the SoA will be screened for antibodies binding to bimagrumab (anti-drug-antibodies, ADA) and the titer of confirmed positive samples will be reported. Other analyses may be performed to evaluate the neutralizing activity or verify the stability of antibodies to bimagrumab and/or further characterize the immunogenicity of bimagrumab.

Instructions for the collection and handling of biological samples will be provided in the laboratory manual.

Results will not be reported to investigative sites or blinded personnel until the study has been unblinded.

Information on storage and future use of samples is detailed in Section 10.1.5.1.

9. STATISTICAL CONSIDERATIONS

The statistical analysis plan (SAP) will be finalized prior to the primary efficacy analysis (e.g., when participants complete Week 48) and it will include a more technical and detailed description of the endpoints to be summarized and the statistical analyses described in this section. This section is a summary of the planned statistical analyses of the key endpoints, subject to possible modifications in the final SAP.

9.1. Statistical Analyses

The primary objective is to assess the effect of varying doses of bimagrumab, semaglutide and bimagrumab in addition to semaglutide on body weight after 48 weeks of treatment. Change in body weight is the primary outcome to be analyzed. The proportion of participants in each treatment group who achieve a weight loss of ≥5% over the course of treatment will also be summarized and compared to control. A viable treatment outcome will be one that shows either:

- The difference in mean weight loss between the active-product and placebo-treated groups is at least 5% and the difference is statistically significant,

or

- The proportion of participants who lose greater than or equal to 5% of baseline body weight in the active-product group is at least 35%, is approximately double the proportion in the placebo-treated group, and the difference between groups is statistically significant.

The FDA guidance for phase 3 clinical trials in weight management recommends that in general, a product can be considered effective for weight management if after 1 year of treatment either of the following occurs:

- The difference in mean weight loss between the active-product and placebo-treated groups is at least 5 percent and the difference is statistically significant

or

- The proportion of participants who lose greater than or equal to 5 percent of baseline body weight in the active-product group is at least 35 percent, is approximately double the proportion in the placebo-treated group, and the difference between groups is statistically significant.

For phase 2 clinical trials, the FDA guidance states that primary efficacy endpoints should include a comparison of the mean absolute or percent change in body weight between the active-product and placebo-treated groups.

All randomized participants who received at least one dose of study medication will be included in an intention-to-treat population (treatment policy strategy). Baseline and efficacy results will be summarized by randomized treatment arm, with active treatment arms compared to placebo, and combination treatment arms to each monotherapy of the same dose.

The analyses to be performed will be similar to the primary analysis described above. Details of these analyses will be provided in the SAP.

9.1.1. Multiplicity Adjustment

No multiplicity adjustment will be performed for this study.

9.2. Analysis Sets

For the purposes of statistical analysis and data summaries, the following analysis sets are defined:

Analysis Set	Description
Full analysis set (FAS)	1. All participants who meet eligibility criteria, sign the informed consent and are randomized to a treatment arm, regardless of exposure to investigational products, are included in the FAS.
Safety analysis set (SAF)	2. All participants who meet the definition of FAS and who are exposed to at least one dose of investigational products (including placebo) are included in the SAF.

The FAS will be used to summarize demographics and baseline characteristics and analyze endpoints related to the efficacy objectives. Participants will be grouped in summaries and included in the analyses according to the planned investigational intervention.

The SAF will be used to summarize and analyze the endpoints relate to safety and tolerability. Participants will be grouped in summaries and included in the analyses according to the highest dose level of investigational products they actually received.

9.3. Interim Analysis

An interim analysis (IA) will be conducted when approximately 80% of the participants complete the Week 24 visit. The interim analysis will evaluate efficacy as well as the safety profile of both the combination and monotherapy treatment arms, and may be used to help determine the doses of bimagrumab to be used in future studies.

9.4. Treatment Extension and Post-Treatment Follow-Up Period Analyses

There will be no contemporaneous placebo/no treatment group with data through the extension phase since all participants originally enrolled into the placebo/no treatment arm will be switched to 30 mg/kg bimagrumab. Likewise, all participants originally enrolled into the 10 mg/kg bimagrumab arm will switch to 30 mg/kg bimagrumab. All other arms will remain unchanged. As a result, the maximum placebo response will be considered the response at the end of the core phase (48 weeks). Analyses and summaries of treatment effect will continue to be based on the original randomization assignments. Analyses and summaries of treatment effect at 72 weeks will be provided.

After 72 weeks, all participants remaining in the study will be withdrawn from treatment and followed for an additional 24 weeks. Analyses and summaries of withdrawal effect will be provided.

Details of analysis methods will be presented in an SAP for the extension phase.

9.5. Sample Size Determination

Approximately 495 participants will be randomized to 1 of 9 treatment groups. The sample size calculation is based on the primary efficacy estimand for weight. Based on the FDA guidance on treatment for weight management, the minimum meaningful treatment effect for weight loss is 5% of baseline body weight over 1 year of treatment. Assuming the minimum treatment effect of 5% weight loss (approximately 5 kg) with a standard deviation (SD) of 8, for a minimum effect size of 0.62 [2, 3], a sample size of 45 participants per treatment arm will have approximately 83.6% statistical power to detect a treatment difference between any active group and placebo/no treatment using a 2-sided t-test at alpha=0.05. Assuming a 20% dropout rate, enrollment will target 55 participants per treatment arm.

Sample size was estimated using a t-test for 2 means in NQuery Advisor V9.1 (www.statsols.com). [4, 5].

10. SUPPORTING DOCUMENTATION AND OPERATIONAL CONSIDERATIONS

10.1. APPENDIX 1: Regulatory, Ethical, and Study Oversight Considerations

10.1.1. Regulatory and Ethical Considerations

This study will be conducted in accordance with the protocol and with the following:

- Consensus ethical principles derived from international guidelines including the Declaration of Helsinki and Council for International Organizations of Medical Sciences (CIOMS) international ethical guidelines
- Applicable ICH Good Clinical Practice (GCP) guidelines
- Applicable laws and regulations

The protocol, protocol amendments, ICF, Investigator's brochure, and other relevant documents (e.g., advertisements) must be submitted to an IRB/EC by the Investigator and reviewed and approved by the IRB/IEC before the study is initiated.

Any amendments to the protocol will require IRB/EC approval before implementation of changes made to the study design, except for changes necessary to eliminate an immediate hazard to study participants.

Protocols and any substantial amendments to the protocol will require health authority approval prior to initiation except for changes necessary to eliminate an immediate hazard to study participants.

The Investigator will be responsible for the following, as applicable:

- Providing written summaries of the status of the study to the IRB/EC annually or more frequently in accordance with the requirements, policies, and procedures established by the IRB/EC.
- Notifying the IRB/EC of SAEs or other significant safety findings as required by IRB/EC procedures.
- Providing oversight of the conduct of the study at the site and adherence to requirements of 21 CFR, ICH guidelines, the IRB/EC, and all other applicable local regulations.

10.1.2. Financial Disclosure

Investigators and sub-Investigators will provide the Sponsor with sufficient, accurate financial information as requested to allow the Sponsor to submit complete and accurate financial certification or disclosure statements to the appropriate regulatory authorities. Investigators are responsible for providing information on financial interests during the study and for 1 year after completion of the study.

10.1.3. Informed Consent Process

The Investigator or the Investigator's representative will explain the nature of the study, including the risks and benefits, to the potential participant and answer all questions regarding the study.

Potential participants must be informed that their participation is voluntary. They will be required to sign a statement of informed consent that meets the requirements of 21 CFR 50, local regulations, ICH guidelines, privacy, and data protection requirements, where applicable, and the IRB/IEC or study center.

The medical record must include a statement that written informed consent was obtained before the participant was enrolled in the study and the date the written consent was obtained. The authorized person obtaining the informed consent must also sign the ICF.

Participants must be reconsented to the most current version of the ICF(s) during their participation in the study.

A copy of the ICF(s) must be provided to the participant. Participants who are rescreened are required to sign a new ICF.

10.1.4. Recruitment Strategy

Recruitment into the study will be via the clinical Investigator sites' patient database, new referrals. Advertisements for the study may also be used, following EC/IRB approval.

10.1.5. Data Protection

Participants will be assigned a unique identifier by the Sponsor. Any participant records or datasets that are transferred to the Sponsor will contain the identifier only; participant names or any information which would make the participant identifiable will not be transferred.

The participant must be informed that their personal study-related data will be used by the Sponsor in accordance with local data protection law. The level of disclosure must also be explained to the participant who will be required to give consent for their data to be used as described in the informed consent.

The participant must be informed that their medical records may be examined by Clinical Quality Assurance auditors or other authorized personnel appointed by the Sponsor, and by inspectors from regulatory authorities.

The contract between Sponsor/designee and study sites specifies responsibilities of the parties related data protection, including handling of data security breaches and respective communication and cooperation of the parties.

Information technology systems used to collect, process, and store study-related data are secured by technical and organizational security measures designed to protect such data against accidental or unlawful loss, alteration, or unauthorized disclosure or access.

10.1.5.1. Future Use of Stored Specimens and Data

Any residual samples remaining after the protocol-defined analysis has been performed may be used for additional exploratory analysis of similar kind. For example, samples analyzed for

enzyme or hormone levels may indicate a need for further isozyme or related hormone analyses to better understand the results. These results may be included in the clinical study report.

Future use may also include, but is not limited to, using residual samples for analytical purposes (e.g., to cross check between different sites and/or for stability assessment). Given the exploratory nature of the work, the analytical method used for those assessments may not be validated. As such, the results from this exploratory analysis will not be included in the clinical study report.

The Sponsor may store samples for up to 15 years after the end of the study to achieve study objectives. Additionally, with participants' consent, samples may be used for further research by the Sponsor or others such as universities or other companies to contribute to the development of related or new treatments or research methods.

During the conduct of the study, an individual participant can choose to withdraw consent to have biological specimens stored for future research. However, withdrawal of consent regarding biosample storage may not be possible after the study is completed.

10.1.6. Data Safety Committee

Participant safety will be continuously monitored by the site Investigator and medical monitor, which includes safety signal detection at any time during the study.

In addition, an early aggregated safety data review will be performed by the DMC, the goal of which is to ensure no emerging safety trends. An initial safety review for this study is planned for the first 25% of participants who are dosed and have provided safety data for at least 45 days after administration of the first dose. Enrollment will not be paused during the review. Thereafter, the DMC will meet periodically, with ad hoc meetings as necessary.

The committee will consist of at least 2 independent clinicians, knowledgeable in bariatric treatment, and 1 biostatistician. Prior to initiating enrollment, a DMC charter will be drafted and approved by the committee members. The primary role of the DMC is to advise on study conduct that will ensure participant safety. This may include recommendations to discontinue a specific treatment arm that appears to be showing characteristics of intolerance, or the study as a whole. Unblinded data may be provided to the DMC in accordance with the DMC charter.

Although the DMC may make recommendations to the Sponsor about changes in the conduct of the study, final decisions will be made by the Sponsor. In the case of early termination, consultation with Health Authorities may be required.

Members of the DMC will not share any unblinded or semi-blinded information with anyone outside of the DMC. Particularly, the Sponsor blinded team members will remain fully blinded to any results throughout the study unless the DMC recommends changes in the conduct of the study (for example, early termination due to negative safety findings).

An independent statistical reporting team not involved in the conduct of the studies will prepare the information for the DMC according to the specifications from the DMC statistician. The main tasks may include:

- Generation of blinded and/or unblinded outputs for the DMC, including tables, figures, and listings, as required.

- Preparation of any other reports requested by the DMC during the closed session.
- Review of the data reports before sending to the DMC.

10.1.7. Site Monitoring

Before an investigational site can enroll participants into the study, the Sponsor or designee will visit the investigational study site to:

- Determine the adequacy of the facilities.
- Discuss with the Investigator(s) and other personnel their responsibilities regarding protocol adherence and applicable regulations. This will be documented in a Clinical Study Agreement between the Sponsor/designee and the Investigator.
- Sites which have been qualified for a previous Sponsor study within the past 2 years or sites participating in low-risk studies, do not need an onsite visit and may be qualified remotely.

During the study, the Sponsor or designee will have regular contacts with the investigational site, for the following:

- Provide information and support to the Investigator(s).
- Confirm that facilities remain acceptable.
- Confirm that the investigational team is adhering to the protocol and that data are being accurately recorded in the case report forms.
- Perform source data verification. This includes a comparison of the data in the case report forms with the participant’s medical records at the hospital or practice, and other records relevant to the study. This will require direct access to all original records for each participant (e.g., clinic charts).
- Record and report any protocol deviations not previously sent to the Sponsor/designee.
- Confirm AEs and SAEs have been properly documented on eCRFs and confirm any SAEs have been forwarded to the Sponsor/designee and those SAEs that met criteria for reporting have been forwarded to the IRB.

The monitor will be available between visits if the Investigator(s) or other staff needs information or advice.

10.1.8. Clinical Monitoring

Clinical site monitoring is conducted to ensure that the rights and well-being of trial participants are protected, that the reported trial data are accurate, complete, and verifiable, and that the conduct of the trial is in compliance with the currently approved protocol/amendment(s), with International Conference on Harmonization Good Clinical Practice (ICH GCP), and with applicable regulatory requirement(s).

Monitoring for this study will be performed by the CRO.

Details of clinical site monitoring are documented in the Clinical Monitoring Plan (CMP). The CMP describes in detail who will conduct the monitoring, at what frequency monitoring will be done, at what level of detail monitoring will be performed, and the distribution of monitoring reports.

Independent audits may be conducted by Sponsor/designee to ensure monitoring practices are performed consistently across all participating sites and that monitors are following the CMP.

10.1.9. Audits and Inspections

Authorized representatives of the Sponsor, a regulatory authority, an Independent Ethics Committee or an Institutional Review Board may visit the site to perform audits or inspections, including source data verification.

The purpose of a sponsor audit or inspection is to systematically and independently examine all study-related activities and documents to determine whether these activities were conducted, and data were recorded, analyzed, and accurately reported according to the protocol, Good Clinical Practice guidelines of the International Conference on Harmonization, and any applicable regulatory requirements. The Investigator should contact the Sponsor immediately if contacted by a regulatory agency about an inspection.

10.1.10. Data Quality Assurance

- All participant data relating to the study will be recorded on printed or electronic eCRFs unless transmitted to the Sponsor or designee electronically (e.g., laboratory data). The Investigator is responsible for verifying that data entries are accurate and correct by physically or electronically signing the eCRF.
- Guidance on completion of eCRFs will be provided.
- The Investigator must permit study-related monitoring, audits, IRB/IEC review, and regulatory agency inspections and provide direct access to source documents.
- Monitoring details describing strategy, including definition of study critical data items and processes (e.g., risk-based initiatives in operations and quality such as risk management and mitigation strategies and analytical risk-based monitoring), methods, responsibilities, and requirements, including handling of noncompliance issues and monitoring techniques (central, remote, or on-site monitoring) are provided in the monitoring plan.
- The Sponsor or designee is responsible for the data management of this study, including quality checking of the data.
- The Sponsor assumes accountability for actions delegated to other individuals (e.g., contract research organizations).
- Records and documents, including signed ICFs, pertaining to the conduct of this study must be retained by the Investigator for 20 years after study completion unless local regulations or institutional policies require a longer retention period. No records may be destroyed during the retention period without the written approval of the

Sponsor. No records may be transferred to another location or party without written notification to the Sponsor.

10.1.11. Data Handling and Recordkeeping

Source Documents

Source documents provide evidence for the existence of the participant and substantiate the integrity of the data collected. Source documents are filed at the Investigator's site.

Data reported on the eCRF or entered in the eCRF that are transcribed from source documents must be consistent with the source documents or the discrepancies must be explained. The Investigator may need to request previous medical records or transfer records, depending on the study. Also, current medical records must be available.

Definition of what constitutes source data, and its origin can be found in monitoring guidelines.

The Investigator must maintain accurate documentation (source data) that supports the information entered into the eCRF.

The Sponsor or designee will perform monitoring to confirm that data entered into the eCRF by authorized site personnel are accurate, complete, and verifiable from source documents; that the safety and rights of participants are being protected; and that the study is being conducted in accordance with the currently approved protocol and any other study agreements, ICH GCP, and all applicable regulatory requirements.

The Investigator/study personnel will allow the Sponsor (or designee) and appropriate regulatory authorities access to these records.

Data Collection and Electronic Case Report Form (eCRF) Completion

The Investigator, or designee, will enter data from the clinic's medical records and study source worksheets into an online electronic data capture (EDC) system. All study personnel will be trained and receive a password protected login. Sponsor designated monitors will perform clinical monitoring, including verification of eCRFs and the source documentation.

Data entry should occur in a timely manner for accuracy, but especially for complying with regulations if an unanticipated or serious adverse event occurs (within 24 hours). If delays in entry occur frequently, a formal corrective plan will be established.

Inspection of Records

Sponsor/designee will be allowed to conduct site visits to the investigation facilities for the purpose of monitoring any aspect of the study. The Investigator agrees to allow the monitor to inspect participant charts and study source documents, and other records relative to study conduct.

Retention of Records

The Principal Investigator must maintain all documentation relating to the study for a period of up to 20 years after the end of the study. If it becomes necessary for the Sponsor or the Regulatory Authority to review any documentation relating to the study, the Investigator must permit access to such records.

10.1.12. Study and Site Start and Closure

Study Start

The study start date is defined as the date when the first participant signs the informed consent.

Study/Site Termination

The Sponsor or designee reserves the right to close the study site or terminate the study at any time for any reason at the sole discretion of the Sponsor. Study sites will be closed upon study completion. A study site is considered closed when all required documents and study supplies have been collected and a study-site closure visit has been performed.

The Investigator may initiate study-site closure at any time, provided there is reasonable cause and sufficient notice is given in advance of the intended termination.

Reasons for the early closure of a study site by the Sponsor or Investigator may include but are not limited to:

For study termination:

- Discontinuation of further study intervention development.

For site termination:

- Failure of the Investigator to comply with the protocol, the requirements of the IRB/IEC or local health authorities, the Sponsor's procedures, or GCP guidelines.
- Inadequate or no recruitment (evaluated after a reasonable amount of time) of participants by the Investigator.
- Total number of participants included earlier than expected.

If the study is prematurely terminated or suspended, the Sponsor shall promptly inform the Investigators, the IECs/IRBs, the regulatory authorities, and any CRO(s) used in the study of the reason for termination or suspension, as specified by the applicable regulatory requirements. The Investigator shall promptly inform the participant and should ensure appropriate participant therapy and/or follow-up.

10.1.13. Publication Policy

The results of this study are expected to be published or presented at scientific meetings and in clinical study registries.

The Sponsor will comply with the requirements for publication of study results. In accordance with standard editorial and ethical practice, the Sponsor will generally support publication of multicenter studies only in their entirety and not as individual site data. In this case, a coordinating Investigator will be designated by mutual agreement.

Authorship will be determined by mutual agreement and in line with the International Committee of Medical Journal Editors authorship requirements.

10.2. APPENDIX 2: Clinical Laboratory Tests

The tests detailed in Table 5 will be performed by the safety lab (local or central, depending on region).

Protocol-specific requirements for inclusion or exclusion of participants are detailed in Section 5 of the protocol.

Additional tests may be performed at any time during the study as determined necessary by the Investigator or required by local regulations.

Table 5: Safety Laboratory Tests

Laboratory Tests	Parameters
**Hematology**	• Hemoglobin<br>• Hematocrit<br>• Red blood cell (RBC) count<br>• RBC indices<br>– Mean corpuscular volume (MCV)<br>– Mean corpuscular hemoglobin (MCH)<br>– Mean corpuscular hemoglobin concentration (MCHC)<br>– Reticulocytes (% and absolute)<br>• White blood cell (WBC) count with differential (% and absolute)<br>• Neutrophils<br>– Lymphocytes<br>– Monocytes<br>– Eosinophils<br>– Basophils<br>• Erythrocyte sedimentation rate<br>• Platelet count

Laboratory Tests	Parameters
**Clinical chemistry**[a]	• Sodium<br>• Potassium<br>• Chloride<br>• Bicarbonate (CO2)<br>• Phosphate<br>• Magnesium<br>• Calcium<br>• Albumin<br>• Total protein<br>• Uric acid<br>• Urea/Blood urea nitrogen (BUN)<br>• Creatinine<br>• Glucose<br>• Creatine kinase (CK)<br>• Lipase<br>• Amylase<br>• Liver function tests<br>– AST / SGOT<br>– ALT/ SGPT<br>– LDH<br>– GGT<br>– Alkaline phosphatase<br>• Total and direct bilirubin
**Urinalysis and urine chemistry**	• pH, specific gravity, glucose, protein, bilirubin, nitrite, and leukocytes, blood, ketones by dipstick<br>• Microscopic examination (if blood or protein is abnormal)<br>• Microalbumin, urine creatinine, albumin creatine ratio (calculated)
**Pregnancy testing**	• Serum (for all women at screening and to confirm a positive urine test)<br>• Rapid urine human chorionic gonadotropin (hCG) pregnancy test (for all other visits)
**Coagulation parameters**	• aPTT, PT/INR

Laboratory Tests	Parameters
**Screening only tests**	• Total cholesterol<br>• Triglycerides<br>• HDL cholesterol<br>• LDL cholesterol<br>• HbA1C<br>• Follicle-stimulating hormone (FSH) for post-menopausal women<br>• Urine drug screen (to include at minimum: amphetamines, barbiturates, cocaine, opiates, cannabinoids and benzodiazepines)<br>• Thyroid-stimulating hormone (TSH), conducted unless a normal value is documented within 6 months prior to screening<br>• HIV antibody, HBsAg, HCVAb

[a] Details of liver, pancreas, and CK chemistry stopping criteria and required actions and follow-up are given in Sections 10.6, 10.7, and 10.8 (Appendices 6, 7, and 8), respectively.

Investigators must document their review of each laboratory safety report.

The tests detailed in Table 6 will be performed by the central lab. A list of potential biomarkers to be analyzed by the central lab may include, but is not limited to, those in Table 6. These results may not be reported to the Investigator until the end of the study.

Table 6: Other Laboratory Tests (Central Lab)

Laboratory Tests	Parameters
**Adipokines**	• Leptin<br>• Adiponectin<br>• Adipsin
**Fasting lipid profile**	• Total cholesterol<br>• HDL cholesterol<br>• LDL cholesterol<br>• Triglycerides
**Inflammation biomarkers**	• IL-6<br>• IL-18<br>• hsCRP<br>• NGAL
**Endocrine biomarkers**	• FSH<br>• Estradiol<br>• Free testosterone<br>• Insulin<br>• HbA1c<br>• Serum CTX<br>• BSAP<br>• P1NP
**TGF-β superfamily biomarkers**	• Activins (TBD)<br>• GDFs (TBD)

**Immunogenicity**	• Anti-drug antibodies (ADA) to bimagrumab
**Safety biomarkers**	• ALP isoenzymes<br>• Amylase isoenzymes<br>• Lipase isoenzymes<br>• 5'NT<br>• miR-122<br>• CCK18

10.3. APPENDIX 3: AEs and SAEs: Definitions and Procedures for Recording, Evaluating, Follow-up, and Reporting

10.3.1. Definition of AE

AE Definition

- An AE is any untoward medical occurrence in a clinical study participant, temporally associated with the use of study intervention, whether or not considered related to the study intervention.
- NOTE: An AE can therefore be any unfavorable and unintended sign (including an abnormal laboratory finding), symptom, or disease (new or exacerbated) temporally associated with the use of study intervention.

Definition of Unsolicited and Solicited AE

- An unsolicited AE is an AE that was not solicited using a participant diary and that is communicated by a participant who has signed the informed consent. Unsolicited AEs include serious and nonserious AEs.
- Potential unsolicited AEs may be medically attended (i.e., symptoms or illnesses requiring a hospitalization, emergency room visit, or visit to/by a healthcare provider). The participant will be instructed to contact the site as soon as possible to report medically attended event(s), as well as any events that, though not medically attended, are of participant concern. Detailed information about reported unsolicited AEs will be collected by qualified site personnel and documented in the participant's records.
- Unsolicited AEs that are not medically attended nor perceived as a concern by the participant will be collected during an interview with the participant and by review of available medical records at the next visit.
- Solicited AEs are predefined local (at the injection site) and systemic events for which the participant is specifically questioned, and which are noted by the participant in their diary.

Events Meeting the AE Definition

- Any abnormal laboratory test results (hematology, clinical chemistry, or urinalysis) or other safety assessments (e.g., ECG, radiological scans, vital signs measurements), including those that worsen from baseline, considered clinically significant in the medical and scientific judgment of the Investigator (i.e., not related to progression of underlying disease, or more severe than expected for the participant's condition)
- Exacerbation of a chronic or intermittent pre-existing condition including either an increase in frequency and/or intensity of the condition
- New condition detected or diagnosed after study intervention administration even though it may have been present before the start of the study
- Signs, symptoms, or the clinical sequelae of a suspected drug-drug interaction

- Signs, symptoms, or the clinical sequelae of a suspected overdose of either study intervention or a concomitant medication. Overdose per se will not be reported as an AE/SAE unless it is an intentional overdose taken with possible suicidal/self-harming intent. Such overdoses should be reported regardless of sequelae.

Events not Meeting the AE Definition

- Any abnormal laboratory findings or other abnormal safety assessments that are associated with the underlying disease, unless judged by the Investigator to be more severe than expected for the participant's condition
- The disease/disorder being studied or expected progression, signs, or symptoms of the disease/disorder being studied, unless more severe than expected for the participant's condition
- Medical or surgical procedure (e.g., endoscopy, appendectomy): the condition that leads to the procedure is the AE
- Situations in which an untoward medical occurrence did not occur (social and/or convenience admission to a hospital)
- Anticipated day-to-day fluctuations of pre-existing disease(s) or condition(s) present or detected at the start of the study that do not worsen.

10.3.2. Definition of SAE

An SAE is defined as any untoward medical occurrence that, at any dose, meets one or more of the criteria listed:

a. Results in death
b. Is life threatening

The term *life threatening* in the definition of *serious* refers to an event in which the participant was at risk of death at the time of the event. It does not refer to an event, which hypothetically might have caused death, if it were more severe.

c. Requires inpatient hospitalization or prolongation of existing hospitalization

   - In general, hospitalization signifies that the participant has been admitted (usually involving at least an overnight stay) at the hospital or emergency ward for observation and/or treatment that would not have been appropriate in the physician's office or outpatient setting. Complications that occur during hospitalization are AEs. If a complication prolongs hospitalization or fulfills any other serious criteria, the event is serious. When in doubt as to whether hospitalization occurred or was necessary, the AE should be considered serious.
   - Hospitalization for elective treatment of a pre-existing condition that did not worsen from baseline is not considered an AE.

d. Results in persistent or significant disability/incapacity

   - The term disability means a substantial disruption of a person's ability to conduct normal life functions.

- This definition is not intended to include experiences of relatively minor medical significance such as uncomplicated headache, nausea, vomiting, diarrhea, influenza, and accidental trauma (e.g., sprained ankle) that may interfere with or prevent everyday life functions but do not constitute a substantial disruption.

e. Is a congenital anomaly/birth defect
f. Is a suspected transmission of any infectious agent via an authorized medicinal product
g. Other situations:

- Medical or scientific judgment should be exercised by the Investigator in deciding whether SAE reporting is appropriate in other situations such as important medical events that that may not be immediately life-threatening or result in death or hospitalization but may jeopardize the participant or may require medical or surgical intervention to prevent one of the other outcomes listed in the above definition. These events should usually be considered serious.
- Examples of such events include invasive or malignant cancers, intensive treatment in an emergency room or at home for allergic bronchospasm, blood dyscrasias, convulsions not resulting in hospitalization, or development of intervention dependency or intervention abuse. All malignancies must be reported as SAEs.

10.3.3. Recording and Follow-Up of AE and/or SAE

AE and SAE Recording

- When an AE/SAE occurs, it is the responsibility of the Investigator to review all documentation (e.g., hospital progress notes, laboratory reports, and diagnostics reports) related to the event.
- The Investigator will then record all relevant AE/SAE information.
- It is not acceptable for the Investigator to send photocopies of the participant's medical records in lieu of completion of the required form.
- There may be instances when copies of medical records for certain cases are requested by the Sponsor/designee. In this case, all participant identifiers, except for the participant number, will be redacted on the copies of the medical records before submission.
- The Investigator will attempt to establish a diagnosis of the event based on signs, symptoms, and/or other clinical information. Whenever possible, the diagnosis (not the individual signs/symptoms) will be documented as the AE/SAE.

Assessment of Intensity

The Investigator will make an assessment of intensity based on the CTCAE grades [36] for each AE and SAE reported during the study.

Assessment of Causality

- The Investigator is obligated to assess the relationship between study intervention and each occurrence of each AE/SAE. The Investigator will use clinical judgment to determine the relationship.
- A *reasonable possibility* of a relationship conveys that there are facts, evidence, and/or arguments to suggest a causal relationship, rather than a relationship cannot be ruled out.
- Alternative causes, such as underlying disease(s), concomitant therapy, and other risk factors, as well as the temporal relationship of the event to study intervention administration, will be considered and investigated.
- For causality assessment, the Investigator will also consult the IB and/or product information, for marketed products.
- The Investigator must review and provide an assessment of causality for each AE/SAE and document this in the medical notes. There may be situations in which an SAE has occurred, and the Investigator has minimal information to include in the initial report. However, it is very important that the Investigator always assess causality for every event before the initial transmission of the SAE data.
- The Investigator may change their opinion of causality considering follow-up information and send an SAE follow-up report with the updated causality assessment.
- The causality assessment is one of the criteria used when determining regulatory reporting requirements.

Follow-up of AEs and SAEs

- The Investigator is obligated to perform or arrange for the conduct of supplemental measurements and/or evaluations as medically indicated or as requested by the Sponsor to elucidate the nature and/or causality of the AE or SAE as fully as possible. This may include additional laboratory tests or investigations, histopathological examinations, or consultation with other health care professionals.
- If a participant dies during participation in the study or during a recognized follow-up period, the Investigator will provide Sponsor/designee with a copy of any postmortem findings including histopathology.
- New or updated information will be recorded in the originally submitted documents.
- The Investigator will submit any updated SAE data to Sponsor/designee within 24 hours of receipt of the information.

10.3.4. Reporting of SAEs

SAE Reporting to Sponsor/designee via an Electronic Data Collection Tool

- The primary mechanism for reporting an SAE to Sponsor/designee will be the electronic data collection tool.

- If the electronic system is unavailable, then the site will use the paper SAE data collection tool (see next section) to report the event within 24 hours.
- The site will enter the SAE data into the electronic system as soon as it becomes available.
- After the study is completed at a given site, the electronic data collection tool will be taken offline to prevent the entry of new data or changes to existing data.
- If a site receives a report of a new SAE from a study participant or receives updated data on a previously reported SAE after the electronic data collection tool has been taken offline, then the site can report this information on a paper SAE form (see next section) or to the Sponsor/designee by telephone.
- Contacts for SAE reporting can be found in the SOM.

SAE Reporting to Sponsor/designee via Paper Data Collection Tool

- Facsimile transmission of the SAE paper data collection tool is the preferred method to transmit this information to the Sponsor/designee.
- In rare circumstances and in the absence of facsimile equipment, notification by telephone either verbally (less preferred) or by cell phone photo (more preferred) is acceptable with a copy of the SAE data collection tool sent by overnight mail or courier service.
- Initial notification via telephone does not replace the need for the Investigator to complete and sign the SAE data collection tool within the designated reporting timeframes.
- Contacts for SAE reporting can be found in SOM.

10.4. APPENDIX 4: Contraceptive Guidance

In order to prevent pregnancy, female participants must satisfy one of the following criteria:

- Be post-menopausal, defined as having had ≥12 months of natural (spontaneous) amenorrhea with an appropriate clinical/hormonal profile (e.g., FSH > 40 IU/L)
- Have undergone surgical sterilization (surgical bilateral oophorectomy with or without hysterectomy), total hysterectomy or tubal ligation at least six weeks before taking study treatment. In case of oophorectomy alone, only when the reproductive status of the woman has been confirmed by follow up hormone level assessment.
- All other female participants must meet both of the following criteria:
  - Two negative pregnancy tests (1 at screening and 1 at randomization)
  - Use of an intrauterine device, from at least 3 months prior to the baseline visit through at least 4 months after the last dose of bimagrumab/placebo i.v.
  - Use of an additional contraceptive (barrier) method from screening through at least 4 months after the last dose of bimagrumab/placebo i.v. to further reduce the risk of unintended pregnancy.

10.5. APPENDIX 5: Genetics

10.5.1. Optional Consent

The study includes an optional DNA component which requires a separate informed consent signature if the participant agrees to participate.

Genetic variation may impact a participant's response to study intervention, susceptibility to, and severity and progression of disease. Variable response to study intervention may be due to genetic determinants that impact drug absorption, distribution, metabolism, and excretion; mechanism of action of the drug; disease etiology; and/or molecular subtype of the disease being treated. Therefore, where local regulations and IRB/IEC allow, a blood sample will be collected for DNA analysis from consenting participants.

10.5.2. Use/Analysis of DNA

As technology changes over time, the most appropriate technology will be used at the time the exploratory DNA research is performed. This may include the study of the entire genome.

In addition, recent advances in genotyping technologies have made genome-wide approaches possible. Genome-wide approaches may also be undertaken within the restricted scope described below.

Exploratory DNA research studies are planned as a part of this study with the objectives of identifying genetic factors which may

- be related to obesity
- predict response to treatment with bimagrumab
- predict relative susceptibility to drug-drug interactions
- predict genetic predisposition to side effects

Additional analyses may be conducted if it is hypothesized that this may help further understand the clinical data.

The samples may be analyzed as part of a multi-study assessment of genetic factors involved in the response to bimagrumab or study interventions of this class to understand the study disease or related conditions.

The results of genetic analyses may be reported in the clinical study report (CSR) or in a separate study summary.

10.5.3. Storage of Genetic Samples

The Sponsor will store the DNA samples in a secure storage space with adequate measures to protect confidentiality.

The samples will be retained while research on study interventions of this class or obesity/overweight continues but no longer than 10 years or other period as per local requirements.

10.6. APPENDIX 6: Liver Safety: Suggested Actions and Follow-up Assessments

Table 7: Liver Event and Laboratory Trigger Definitions

Definition	Thresholds
Potential Hy's law case	• ALT or AST >3x ULN and total bilirubin >2x ULN without notable increase in ALP to >2x ULN)
If ALT, AST, and total bilirubin normal at baseline	• ALT or AST >8x ULN<br>• ALT or AST >5x ULN and ≤8x ULN<br>• ALT or AST>3x ULN and ≤5x ULN<br>• ALP >2x ULN (in the absence of known bone pathology)<br>• Total bilirubin >3x ULN (in the absence of known Gilbert syndrome)
ALT or AST elevation with coagulopathy	• ALT or AST >3x ULN and PT/INR >1.5 (in the absence of anti-coagulation)
ALT or AST elevation accompanied by symptoms	• ALT or AST >3x ULN accompanied by (general) malaise, fatigue, abdominal pain, nausea, or vomiting, or rash, or eosinophilia
Isolated ALP elevation	• ALP >2x ULN (unless attributable to bone-specific ALP)
Others	• Any clinical event of jaundice<br>• Any AE potentially indicative of liver toxicity

Table 8: Actions Required for Liver Events and Laboratory Triggers

Criteria	Actions required
Potential Hy's law case<br>ALT or AST elevation with coagulopathy<br>ALT or AST elevation accompanied by symptoms<br>Isolated ALT or AST elevation >8x ULN<br>Jaundice	• If possible Hy's law, report to sponsor within 24 h<br>• Discontinue study treatment<br>• Hospitalize if clinically appropriate<br>• Establish causality
Isolated ALT or AST elevation >5x ULN and ≤8 x ULN	• If confirmed, consider interruption or discontinuation of study drugs<br>• If elevation persists for more than 2 weeks, discontinue study drugs<br>• Follow-up for symptoms<br>• Establish causality
Isolated ALT or AST elevation >3x ULN and ≤5x ULN	• Monitor liver chemistry at least 2 times weekly<br>• If confirmed, consider interruption or discontinuation of study drugs

Criteria	Actions required
	• Follow-up for symptoms
Isolated ALP elevation	• Repeat liver chemistry test within 48–72 hours<br>• If elevation is confirmed, measure fractional ALP; if >50% of it is liver origin, establish hepatic causality
Any AE potentially indicative of liver toxicity	• Consider study treatment interruption or discontinuation<br>• Hospitalize if clinically appropriate

Table 9: Follow Up Requirements for Liver Laboratory Triggers - Isolated Hyperbilirubinemia

Criteria	Actions required
**Total bilirubin (isolated)**	
>1.5–3x ULN	• Maintain treatment<br>• Repeat LFTs within 48-72 hours<br>• Monitor LFTs weekly until resolution to ≤ Grade 1 or to baseline
>3-10x ULN (in the absence of known Gilbert syndrome)	• Interrupt treatment<br>• Repeat LFT within 48-72 hours<br>• Monitor LFTs weekly until resolution to ≤ Grade 1 or to baseline (ALT, AST, total bilirubin, Alb, PT/INR, ALP and GGT)<br>• Test for hemolysis (e.g., reticulocytes, haptoglobin, unconjugated [indirect] bilirubin)<br>• Hospitalize if clinically appropriate<br>• Establish causality
>10x ULN	• Discontinue the study treatment immediately<br>• Hospitalize the participant<br>• Establish causality
Any AE potentially indicative of a liver toxicity	• Consider study treatment interruption or discontinuation<br>• Hospitalization if clinically appropriate<br>• Establish causality

In all instances, record the AE and contributing factors (e.g., concomitant medications, medical history, laboratory results) in the appropriate eCRF.

Based on investigator's discretion, investigation(s) for contributing factors for the liver event can include serology tests, imaging, and pathology assessments, hepatologist's consultancy, obtaining more detailed history of symptoms and prior or concurrent diseases, history of concomitant drug use, exclusion of underlying liver disease.

10.7. APPENDIX 7: Lipase and Amylase Safety

To ensure participant safety and enhance reliability in determining the potential for pancreatic events with bimagrumab, a standardized process for identification, monitoring and evaluation of pancreatic events should be followed (Table 10).

Table 10: Safety Monitoring Guidance for Amylase and Lipase Elevations

Event	Follow up monitoring
Lipase and/or amylase >2x and ≤3x ULN	• If asymptomatic, re-check enzymes (amylase, lipase) and additional follow up at discretion of Investigator.<br>• If clinical symptoms are present (e.g., abdominal pain, nausea, diarrhea) and suggestive of pancreatic involvement, re-check enzymes (amylase, lipase) and inflammatory markers (e.g., C-reactive protein) and consider pancreatic imaging (e.g., contrast-enhanced CT or MRI/MRCP) to evaluate for presence of pancreatitis.<br>• If serum lipase is >2x ULN *and* >1.5x baseline at or prior to Visit 4, interrupt study treatments and consult with medical monitor.
Lipase and/or amylase >3x and ≤5X ULN	• If at or prior to Visit 4, interrupt study treatments and consult with medical monitor.<br>• Re-check enzymes (amylase, lipase) and inflammatory markers (C-reactive protein) independent of presence of clinical symptoms, until enzyme levels return to <2x ULN.<br>• Assess participant for clinical symptoms (e.g., abdominal pain, nausea, diarrhea).<br>• Conduct pancreatic imaging (e.g., CECT or MRI/MRCP) to evaluate for presence of pancreatitis.<br>• If clinical symptoms and/or pancreatic imaging indicative of pancreatitis, discontinue study treatments.
>5x ULN or confirmed diagnosis of pancreatitis	• Discontinue study treatments.<br>• Hospitalize if clinically appropriate.<br>• Establish causality.

Medically significant pancreatic events which are considered as serious adverse events (SAEs) should follow the **standard procedures for SAE reporting** as described in Section 8.4. Every pancreatic event reported as an SAE should include a causality assessment of the event, considering alternative causes (e.g., gallstones, co-medication).

An investigation of the pancreas needs to be followed up until resolution. A gastroenterology consult can be included at the Investigator’s discretion. All follow-up information, and the procedures performed, should be recorded in the appropriate eCRFs.

An independent adjudication committee may be formed to adjudicate suspected cases of acute pancreatitis and other major adverse events in a blinded manner. Any completed adjudications will be utilized in safety analyses.

10.8. APPENDIX 8: Creatine Kinase Safety

To ensure participant safety and enhance reliability in determining the potential for rhabdomyolysis, a standardized process for identification, monitoring and evaluation of serum CK should be followed (Table 11).

Table 11: Safety Monitoring Guidance for Creatine Kinase Elevations

Event	Follow up monitoring
Creatine kinase (CK) >5x ULN and ≤10x ULN	• Inquire about recent intensive muscular exertion or injury, infection, body temperature changes, medications such as statins<br>• Assess participant for clinical symptoms (e.g., myalgia, weakness, dark urine).<br>• Re-check CK and check serum troponin I/T, creatinine, sodium, potassium, calcium, phosphate, and urine myoglobin<br>• If symptoms and/or lab tests indicative of rhabdomyolysis, discontinue study treatments.
Creatine kinase (CK) >10x ULN or rhabdomyolysis is confirmed	• In addition to above actions:<br>o Interrupt study drugs and consult with the medical monitor<br>o Discontinue study if rhabdomyolysis is confirmed<br>o Hospitalize if clinically appropriate.<br>o Establish causality

11. REFERENCES

1. NICE. *Obesity: identification and classification of overweight and obesity (update)*. 2022; Available from: https: //www .nice.org.uk/guidance/indevelopment/gid-ng10284.
2. Jastreboff, A.M., et al., *Tirzepatide Once Weekly for the Treatment of Obesity.* N Engl J Med, 2022. **387**(3): p. 205-216.
3. Wildman, R.P., *Healthy obesity.* Curr Opin Clin Nutr Metab Care, 2009. **12**(4): p. 438-43.
4. Zhong, P., et al., *Efficacy and safety of once-weekly semaglutide in adults with overweight or obesity: a meta-analysis.* Endocrine, 2022. **75**(3): p. 718-724.
5. Heymsfield, S.B., et al., *Effect of Bimagrumab vs Placebo on Body Fat Mass Among Adults With Type 2 Diabetes and Obesity: A Phase 2 Randomized Clinical Trial.* JAMA Netw Open, 2021. **4**(1): p. e2033457.
6. WHO, *World Health Organization: Obesity and overweight.* 2021.
7. Ritchie, H.a.R., M. *Obesity*. 2017; Available from: https: //ourworldindata.org/obesity.
8. Kushner, R.F., *Weight Loss Strategies for Treatment of Obesity: Lifestyle Management and Pharmacotherapy.* Prog Cardiovasc Dis, 2018. **61**(2): p. 246-252.
9. Ahima, R.S., *Adipose tissue as an endocrine organ.* Obesity (Silver Spring), 2006. **14 Suppl 5**: p. 242S-249S.
10. Janssen, I., P.T. Katzmarzyk, and R. Ross, *Waist circumference and not body mass index explains obesity-related health risk.* Am J Clin Nutr, 2004. **79**(3): p. 379-84.
11. Pischon, T., et al., *General and abdominal adiposity and risk of death in Europe.* N Engl J Med, 2008. **359**(20): p. 2105-20.
12. De Lorenzo, A., et al., *Adiposity rather than BMI determines metabolic risk.* Int J Cardiol, 2013. **166**(1): p. 111-7.
13. Romero-Corral, A., et al., *Accuracy of Body Mass Index to Diagnose Obesity in the US Adult Population.* Int J Obes (Lond), 2008. **32**(6): p. 959-66.
14. Shah, N.R. and E.R. Braverman, *Measuring adiposity in patients: the utility of body mass index (BMI), percent body fat, and leptin.* PLoS One, 2012. **7**(4): p. e33308.
15. Heymsfield, S.B., et al., *Weight loss composition is one-fourth fat-free mass: a critical review and critique of this widely cited rule.* Obesity Reviews, 2014. **15**(4): p. 310-321.
16. Phillips, S.M. and M.B. Zemel, *Effect of protein, dairy components and energy balance in optimizing body composition.* Nestle Nutr Inst Workshop Ser, 2011. **69**: p. 97-108; discussion 108-13.
17. Ravussin, E., et al., *Reduced rate of energy expenditure as a risk factor for body-weight gain.* N Engl J Med, 1988. **318**(8): p. 467-72.
18. Tsai, A.G. and T.A. Wadden, *Systematic review: an evaluation of major commercial weight loss programs in the United States.* Ann Intern Med, 2005. **142**(1): p. 56-66.
19. Heymsfield, S.B., B. Bourgeois, and D.M. Thomas, *Why is it Difficult to Lose and Maintain Large Amounts of Weight with Lifestyle and Pharmacologic Treatments?* Obesity (Silver Spring), 2017. **25**(12): p. 2017.
20. Jo, E., et al., *Resistance training during a 12-week protein supplemented VLCD treatment enhances weight-loss outcomes in obese patients.* Clin Nutr, 2019. **38**(1): p. 372-382.
21. Chmelo, E.A., et al., *Legacy effects of short-term intentional weight loss on total body and thigh composition in overweight and obese older adults.* Nutr Diabetes, 2016. **6**(4): p. e203.

22. Koncarevic, A., et al., *A novel therapeutic approach to treating obesity through modulation of TGFbeta signaling.* Endocrinology, 2012. **153**(7): p. 3133-46.
23. Lee, S.J., et al., *Regulation of muscle mass by follistatin and activins.* Mol Endocrinol, 2010. **24**(10): p. 1998-2008.
24. Lee, S.J., et al., *Regulation of muscle growth by multiple ligands signaling through activin type II receptors.* Proc Natl Acad Sci U S A, 2005. **102**(50): p. 18117-22.
25. Trendelenburg, A.U., et al., *TAK-1/p38/nNFkappaB signaling inhibits myoblast differentiation by increasing levels of Activin A.* Skelet Muscle, 2012. **2**(1): p. 3.
26. Emdin, C.A., et al., *DNA Sequence Variation in ACVR1C Encoding the Activin Receptor-Like Kinase 7 Influences Body Fat Distribution and Protects Against Type 2 Diabetes.* Diabetes, 2019. **68**(1): p. 226-234.
27. Kelly, A., *Trauma-Informed Mindfulness-Based Stress Reduction: A Promising New Model for Working with Survivors of Interpersonal Violence.* Smith College Studies in Social Work, 2015. **85**(2): p. 194-219.
28. Tomlinson, B., et al., *Selective beta-adrenoceptor partial agonist effects of pindolol and xamoterol on skeletal muscle assessed by plasma creatine kinase changes in healthy subjects.* Br J Clin Pharmacol, 1990. **30**(5): p. 665-72.
29. Albanese, C.V., E. Diessel, and H.K. Genant, *Clinical applications of body composition measurements using DXA.* J Clin Densitom, 2003. **6**(2): p. 75-85.
30. Wilding, J.P.H., et al., *Once-Weekly Semaglutide in Adults with Overweight or Obesity.* New England Journal of Medicine, 2021. **384**(11): p. 989-1002.
31. USDHHS, *2008 Physical Activity Guidelines for Americans.* 2008.
32. FDA, *FDA Draft Guidance: Developing Products for Weight Management - Revision 1.* 2007.
33. Garvey, W.T., et al., *Two-year effects of semaglutide in adults with overweight or obesity: the STEP 5 trial.* Nat Med, 2022. **28**(10): p. 2083-2091.
34. Kolotkin, R.L., et al., *Development of a clinical trials version of the Impact of Weight on Quality of Life-Lite questionnaire (IWQOL-Lite Clinical Trials Version): results from two qualitative studies.* Clin Obes, 2017. **7**(5): p. 290-299.
35. Ware, J.E., Jr. and C.D. Sherbourne, *The MOS 36-item short-form health survey (SF-36). I. Conceptual framework and item selection.* Med Care, 1992. **30**(6): p. 473-83.
36. USDHHS, *Common Terminology Criteria for Adverse Events (CTCAE).* 2017.

Signature Page for VV-CLIN-150100 v1.0

Approval	PPD<br>08-Apr-2024 18:00:01 GMT+0000

Approval	PPD<br>09-Apr-2024 09:01:23 GMT+0000

Signature Page for VV-CLIN-150100 v1.0

STATISTICAL ANALYSIS PLAN

(Part 1)

**PROTOCOL NUMBER:**	VER201-PH2-031 (Lilly Alias: J4Z-MC-GIDA)
**TITLE:**	A randomized, double-blind, placebo-controlled multi-center study of intravenous bimagrumab, alone or in addition to open label subcutaneous semaglutide, to investigate the efficacy and safety in overweight or obese men and women
**STUDY DRUG:**	Bimagrumab (LY3985863)
**SPONSOR:**	Versanis Bio, Inc.<br>A wholly owned subsidiary of Eli Lilly and Co.<br>1111 Broadway, Suite 1300<br>Oakland, CA – 94607<br>United States

Approved on 10 Jul 2024 GMT

AUTHORS:

PPD MCG
PPD MCG

REVIEWERS:

PPD MCG
PPD to Versanis, Applied Statistics and Consulting
PPD Versanis Bio, Inc.
PPD Eli Lilly and Company
PPD Eli Lilly and Company
PPD Eli Lilly and Company

LIST OF ABBREVIATIONS

ADA	Anti-Drug-Antibodies
AE	Adverse Event
AESI	Adverse Events of Special Interest
ALT	Alanine Aminotransferase
ALP	Alkaline Phosphate
AST	Aspartate Aminotransferase
aPTT	Activated Partial Thromboplastin Time
BIA	Biomolecular Interaction Analysis
BMD	Bone Mineral Density
BMI	Body Mass Index
CBC	Complete Blood Count
CCK18	Caspase-Cleaved K18
CK	Creatine Kinase
CP	Concomitant Procedures
CRF	Case Report Form
DMC	Data Monitoring Committee
DXA	Dual-Energy X-Ray Absorptiometry
ECG	Electrocardiogram
FAS	Full Analysis Set
FLI	Fat Loss Index
FSH	Follicle Stimulating Hormone
GGT	Gamma-Glutamyl Transferase
HbA1c	Hemoglobin A1C
HBV	Hepatitis B Virus
HCV	Hepatitis C Virus
HDL	High Density Lipoprotein
HIV	Human Immunodeficiency Virus
HOMA2	Homeostasis Model Assessment, version 2
hsCRP	High-sensitivity C-reactive protein
IBW	Ideal Body Weight
ICE	Inter-current Event
IEC	Independent Ethics Committees
IL-6	Interleukin 6
IL-18	Interleukin 18
IgG	Immunoglobulin G
INR	International Normalized Ratio
IRB	Institutional Review Boards
i.v.	Intravenous

IWQOL-Lite-CT	Impact of Weight on Quality of Life-Lite for Clinical Trials
LDL	Low Density Lipoprotein
LLN	Lower Limit of Normal
miR-122	MircoRNA 122
MMRM	Mixed Model for Repeated Measurements
NGAL	Neutrophil gelatinase-associated lipocalin
OLE	Open-label extension
PD	Pharmacodynamic(s)
PDMP	Protocol Deviation Management Plan
PK	Pharmacokinetic(s)
PT	Prothrombin Time
QOL	Quality of Life
QTc	Corrected QT Interval
QUICKI	Quantitative Insulin-Sensitivity Check Index
SAE	Serious Adverse Event
SAF	Safety Analysis Set
SF-36	Short Form 36 Health Survey Questionnaire
s.c.	Subcutaneous
SD	Standard Deviation
SGOT	Serum Glutamic-Oxaloacetic Transaminase
SGPT	Serum Glutamate Pyruvate Transaminase
TEAE	Treatment Emergent Adverse Event
TGF-β	Transforming Growth Factor Beta
ULN	Upper Limit of Normal
VAT	Visceral Adipose Tissue
WD	Withdrawal
WHtR	Waist-to-Height Ratio

CONTENTS

List of Abbreviations 3

Contents 5

1. Introduction 9

2. Study Objectives 9

2.1 Primary Objectives 9

2.2 Secondary Objectives 9

2.3 Exploratory Objectives 10

3. Study Design 10

3.1 General Description 10

3.2 Length of Study 12

3.3 Schedule of Events 12

3.4 Determination of Sample Size 12

4. Planned Analyses 13

4.1 Internal safety review committee 13

4.2 Independent review committee 13

4.3 Interim Analysis 14

4.4 Final Analysis – Core Study Period 14

4.5 Final Analysis – Open-Label Extension Phase 15

5. Analysis Populations 15

5.1 Full Analysis Set/Intention-to-Treat Population 15

5.2 Safety Analysis Set 15

6. Data Handling Conventions 16

6.1 Treatment Groups 16

6.2 Data Presentation 17

6.3 Premature Withdrawal and Missing Data 17

6.4 Common Derivations....18
6.4.1 First Day of Study Drug Administration(TR01SDT)....18
6.4.2 Last Day of Study Drug Administrationduring Core Study Period (TR01EDT)....19
6.4.3 First Day of Study Drug Administration during Extension Treatment Period (TR02SDT)....19
6.4.4 Last Day of Study Drug Administrationduring Extension Treatment Period (TR02EDT)....19
6.4.5 Baseline....19
6.4.6 Core Study Completion....20
6.5 Visit Window Conventions....20
6.5.1 Visit Windows for Body Weight including BIA, Wait Circumference, and Vital Signs....21
6.5.2 Visit Windows for DXA Scans....21
6.5.3 Visit Windows for Grip Strength, QoL Questionnaires, PD Biomarkers, and Dietary Intake....22
6.5.4 Visit Windows for ECG....22
6.5.5 Visit Windows for Hematology (CBC), Clinical Chemistry, Coagulation, and Safety Biomarkers....23
6.5.6 Visit Windows for Urine Chemistry and Urinalysis....23
6.5.7 Visit Windows for Anti-drug Antibodies....24
6.5.8 Visit Windows for Pharmacokinetics....24
6.5.9 Visit Windows for eDiary Assessments....24
6.5.10 Visit Windows for Actigraphy....25
6.6 Software....26
7. Statistical Considerations....26
7.1 Statistical Tests....26
7.2 Multiplicity Adjustments....27
7.3 Multi-center Studies....27
7.4 Other Strata and Covariates....27

7.5 Subgroup Analyses 27

8. Output Shells 27

9. Study Population 27

9.1 Subject Disposition 27

9.2 Protocol Deviations 28

9.3 Demographics and Baseline Characteristics 28

9.4 Medical History and Pre-existing Conditions 29

9.5 Prior and Concomitant Medications, and Procedures 29

10. Efficacy 31

10.1 Primary Efficacy 31

10.1.1 Primary Estimand – Treatment Policy Strategy 31

10.1.2 Primary Endpoint 31

10.1.3 Analysis of Primary Endpoint using Treatment Policy Strategy 31

10.1.4 Secondary Estimand – Trial Product (Hypothetical) Strategy 32

10.1.5 Analysis of the Primary Endpoint Using the Trial Product/Hypothetical Strategy 33

10.1.6 Multiple Imputation of Missing Data for the Treatment Policy Estimand 34

10.2 Sensitivity Analyses 35

10.3 Secondary Efficacy 36

10.3.1 Secondary Efficacy Endpoints 36

10.3.2 Analysis of Continuous Secondary Efficacy Endpoints 39

10.3.3 Analysis of Percentage of Weight Loss due to Fat Mass or Lean Mass at 48 weeks by DXA 40

10.3.4 Analysis of Ratio Secondary Efficacy Endpoints 41

10.3.5 Analysis of Response Rate Secondary Efficacy Endpoints 42

10.3.6 Analysis of Categorical Secondary Efficacy Endpoints 42

10.4 Exploratory Analyses 43

10.4.1 Exploratory Endpoints 43

10.4.2 Analysis of Exploratory Variables 44

11. Safety 45

11.1 Exposure 45

11.1.1 Bimagrumab/Placebo Exposure 45

11.1.2 Semaglutide Exposure 47

11.2 Adverse Events 48

11.2.1 Adverse Events of Special Interest (AESI) 50

11.2.2 Adjudicated Adverse Events 50

11.3 Laboratory Evaluations 51

11.4 Vital Signs 53

11.5 12-lead ECG 54

11.6 Physical Examination 54

11.7 Dietary Intake 54

12. Pharmacokinetics 55

13. Pharmacodynamics 56

14. Immunogenicity 57

15. Changes from Protocol 57

16. APPENDICES 57

16.1 Appendix 1: Details of Laboratory scatterplots 57

16.2 Appendix 2: Details Of MedDRA Terms Used to Define AESI 61

16.3 Appendix 3: Consolidation of Urinalysis Results Categories 64

17. References 66

1. INTRODUCTION

This document describes the planned statistical analyses for the core treatment period (first 48 weeks) of Protocol VER201-PH2-301 (Lilly Alias: J4Z-MC-GIDA), a study conducted by Versanis Bio, Inc, a wholly owned subsidiary of Eli Lilly and Co. The purpose of the study is to evaluate the safety and efficacy of intravenous bimagrumab and subcutaneous semaglutide for weight loss in overweight or obese adults. Analyses for the extension treatment period of the study will be covered by a separate SAP addendum for the Extension Treatment period.

This analysis plan supplements the study protocol (VER201-PH2-301 Protocol Version 5.0 dated 9th April 2024). If the protocol is amended, this statistical analysis plan will be revised as required. The SAP v 1.0 is approved before any sponsor team member were unblinded. The plan will be finalized before the study database is locked for the core treatment period of the study.

2. STUDY OBJECTIVES

The planned study is designed to evaluate the effect of bimagrumab alone or in addition to the approved obesity therapeutic semaglutide in obese or overweight subjects with at least one obesity related co-morbidity, without T2DM.

2.1 Primary Objectives

- To assess the treatment effect of bimagrumab, semaglutide, and bimagrumab in addition to semaglutide vs placebo on body weight.

2.2 Secondary Objectives

- To assess the treatment effect of bimagrumab, semaglutide, and bimagrumab in addition to semaglutide vs placebo on waist circumference (WC, cm).

- To assess treatment effects on fat mass, visceral adipose tissue (VAT), trunk fat mass, subcutaneous adipose tissue (SAT), and lean mass.

- To assess the safety and tolerability of bimagrumab, semaglutide and bimagrumab in addition to semaglutide.
- To assess the proportion of subjects in each treatment group experiencing a change in the categorical classification of obesity based on body mass index (BMI) and waist-to-height ratio (WHtR) group.
- To assess treatment effects in each treatment group on glucose metabolism.
- To assess treatment effects in each treatment group on self-reported health status and weight-related quality of life.

2.3 Exploratory Objectives

- To evaluate treatment effects on pharmacodynamic (PD) biomarkers, including metabolism, lipids, inflammatory, hormonal, and TGF-β superfamily ligands.
- To evaluate treatment effects on grip strength.
- To evaluate treatment effects on physical activity level.
- To evaluate treatment effects on insulin resistance.
- To evaluate treatment effects on total and regional BMD.
- To evaluate the pharmacokinetics of repeat doses of bimagrumab.
- To evaluate the pharmacokinetics of repeat doses of semaglutide.

3. STUDY DESIGN

3.1 General Description

This is a non-confirmatory, multi-center, randomized, double-blind, placebo-controlled (for bimagrumab) study of intravenous bimagrumab, alone or in addition to open label

subcutaneous semaglutide, to investigate efficacy and safety in adult men and women between the ages of 18 and 80, inclusive, who are obese or who are overweight with at least one obesity related co-morbidity. Women must be post-menopausal, post-surgically sterilized or using an intrauterine device.

For the 48-week core treatment period, subjects will be randomized to one of 9 treatment arms formed by the combinations of dosages of bimagrumab and semaglutide in a 3 x 3 factorial design consisting of 3 dose levels of bimagrumab – placebo, 10 mg/kg, and 30 mg/kg, and 3 dose levels of semaglutide – none, semaglutide 1.0mg, and semaglutide 2.4mg. Stratification across the treatment arms will be based on gender.

The 48-week core treatment period will be followed by a 24-week treatment extension period, during which two of the treatment arms will follow a modified treatment assignment: participants randomized to study treatment arms 1 (bimagrumab placebo) and 4 (bimagrumab 10 mg/kg) will switch treatment to bimagrumab 30 mg/kg at Week 48.

Doses of bimagrumab or placebo will be administered intravenously in Week 1 (Day 1), followed by a loading dose at Weeks 4, then every 12 weeks at Weeks 16, 28, 40. Doses of semaglutide will be administered weekly by self-injection.

The study will consist of a screening period to assess eligibility, a baseline visit, followed by clinic visits/phone contacts every 4 weeks during the core treatment period. Clinic visits are scheduled at:

- Weeks 1 (Day 1), 4, 16, 28, and 40 for blinded study treatment (intravenous bimagrumab or placebo) dosing and additional assessments.
- Weeks 12, 24, and 48 for DXA scanning and other assessments.

Site staff will call participants at Weeks 2, 8, 20, 32, 36, and 44 to ensure there are no safety issues and to address any questions the participant may have.

Qualified dieticians will reach out to participants on a monthly basis to provide dietary and exercise counseling, and to calculate participant's dietary intake via 24-hour recall assessment (see study operations manual for detailed description). If desired, these touchpoints may be combined with the in-clinic visits or safety calls.

Should participants report any adverse events over the phone, these will be recorded in the eCRF.

3.2 Length of Study

The total study duration will be approximately 110 weeks and will consist of a screening period of up to 6 weeks, a 72-week treatment period (48 weeks core plus 24 weeks extension), and a 32-week post-treatment follow-up period.

The primary endpoint analysis will take place after 48 weeks of core treatment. The remainder of this SAP will cover the analyses on data collected up to this time point only. A separate SAP addendum will cover the post-Week 48 analyses.

3.3 Schedule of Events

The schedule of assessments can be found in Section 1.3 of the Protocol.

3.4 Determination of Sample Size

Approximately 495 participants will be randomized to 1 of 9 treatment groups. The sample size calculation is based on the primary efficacy estimand for weight. Based on the FDA guidance on treatment for weight management, the minimum meaningful treatment effect for weight loss is 5% of baseline body weight over 1 year of treatment. Assuming the minimum treatment effect of 5% weight loss with a standard deviation (SD) of 8%, for a minimum effect size of 0.62 (Jastreboff, et al., 2022) (Wildman, 2009), a sample size of 45 participants per treatment arm will have approximately 83.6% statistical power to detect a treatment difference between any active group and placebo/no treatment using a 2-sided t-test at alpha=0.05. Assuming a 20% dropout rate, enrollment will target 55 subjects per treatment arm. Sample size was estimated using a t-test for 2 means in NQuery Advisor V9.1 (www.statsols.com).

4. PLANNED ANALYSES

4.1 Internal safety review committee

There is no internal safety review committee planned for this study. Subject safety will be continuously monitored by the site Investigator and medical monitor, which includes safety signal detection at any time during the study.

4.2 Independent review committee

This study will have a contracted independent Data Monitoring Committee (DMC), consisting of at least 2 independent clinicians, knowledgeable in obesity treatment, and 1 biostatistician. In addition, one non-voting members from Eli Lilly (safety) may be included for their expertise. Prior to initiating enrolment, a DMC charter will be drafted and approved by the committee members. The primary role of the DMC will be to advise on study conduct that will ensure subject safety. This may include recommendations to discontinue a specific treatment arm that appears to be showing characteristics of intolerance, or the entire study. Although the DMC may make recommendations to the Sponsor about changes in the conduct of the study, final decisions will be made by the Sponsor.

Unblinded data may be provided to the DMC in accordance with the DMC charter. MCG may provide descriptive and/or analytical statistical data as requested by the DMC to this end. Examples of such data include:

- Summary of demographics and baseline characteristics
- Summary of TEAEs, by System Organ Class (SOC) and by Preferred Term (PT)
- Summary of AEs, by SOC and severity
- Summary of AEs and SAEs deemed to be related to study drug, by SOC and PT
- Summary of serious adverse events (SAEs), by SOC and PT

- Summary of adverse events of special interest, by SOC and PT
- Summary and listing of deaths and accompanying Narratives
- Summary and listing of discontinuations from study
- Summary of key laboratory measurements
- Summaries of PK or PD/efficacy data if requested and/or pertinent

4.3 Interim Analysis

An unblinded interim analysis (IA) will be conducted when approximately 80% of the participants have completed the Week 24 visit or discontinued study treatment. The interim analysis will evaluate efficacy as well as the safety profile of both the combination and monotherapy treatment arms. The team conducting the unblinded interim analysis will be independent from the main study team and will use the results for internal decision-making purposes only. The Data Management Committee will have access to the interim analysis results. The detailed plan of unblinding is included in a separate blinding and unblinding plan.

4.4 Final Analysis – Core Study Period

The core study period is the randomized, double-blind, placebo-controlled portion of the study. This phase is the focus of this SAP. The final analysis for the core study period will be performed once all enrolled subjects have completed the end of study/Week 48 visit and a 48-week primary analysis database lock has occurred. The data will be unblinded and the analyses described in this SAP will be conducted.

The 48-week primary analysis database lock will include locking the data for all visits up to week 48.

4.5 Final Analysis – Open-Label Extension Phase

Following the analysis of the core study period, the study will then become open-label. Details of analyses of the open-label extension (OLE) phase, which includes data collected from 48 weeks through 72 weeks, and the withdrawal (WD) phase, which will end after the last participant completed 104 weeks, will be documented in a separate SAP addendum. The final analysis will be conducted once all enrolled subjects have completed the end of study/Week 104 visit and database lock has occurred.

At the time of the open-label analysis, the external data transfers will be programmatically compared to those received at the time of the 48-week primary analysis database lock and any differences will be documented.

5. ANALYSIS POPULATIONS

5.1 Full Analysis Set/Intention-to-Treat Population

The Full Analysis Set (FAS)/Intention-to-Treat (ITT) Population will include all subjects who provided informed consent and were randomised to a treatment group. This population will be used for subject disposition outputs, demographics, baseline characteristics and efficacy outputs unless otherwise specified.

5.2 Safety Analysis Set

The Safety Analysis Set (SAF) will include all subjects who received at least one dose of study medication. Subjects will be analyzed as randomized, regardless of the actual treatment received. This population will be used for all safety, pharmacokinetics, and pharmacodynamics outputs unless otherwise specified.

6. DATA HANDLING CONVENTIONS

6.1 Treatment Groups

Data will be grouped by treatment group, using the following treatment names and order.

a) Placebo

b) Bima 10mg/kg

c) Bima 30mg/kg

d) Sema 1.0mg

e) Sema 2.4mg

f) Bima 10 mg/kg + Sema 1.0 mg

g) Bima 10 mg/kg + Sema 2.4 mg

h) Bima 30 mg/kg + Sema 1.0 mg

i) Bima 30 mg/kg + Sema 2.4 mg

These groupings will apply for all analyses up to Week 48. For listings the extension group arm will also be included as below:

- Placebo / Bima 30mg/kg
- Bima 10mg/kg / Bima 30mg/kg
- Bima 30mg/kg / Bima 30mg/kg
- Placebo + Sema 1.0mg / Placebo + Sema 1.0mg
- Placebo + Sema 2.4mg / Placebo + Sema 2.4mg

- Bima 10 mg/kg + Sema 1.0 mg / Bima 10 mg/kg + Sema 1.0 mg
- Bima 10 mg/kg + Sema 2.4 mg / Bima 10 mg/kg + Sema 2.4 mg
- Bima 30 mg/kg + Sema 1.0 mg / Bima 30 mg/kg + Sema 1.0 mg
- Bima 30 mg/kg + Sema 2.4 mg / Bima 30 mg/kg + Sema 2.4 mg

For summaries that include a total arm, this will use the name 'All Subjects'.

6.2 Data Presentation

Descriptive analyses will be completed for this study. For continuous parameters, these will include the number of observations, mean, median, standard deviation, minimum and maximum. The minimum and maximum values will be presented to the same number of decimal places as recorded in the database. Mean, median and standard deviation will be presented to 1 more decimal place than the raw data. Categorical parameters will be summarized as frequency counts and percentages. Percentages will be rounded to 1 decimal place, with the denominator being the number of subjects in the relevant population, unless otherwise stated. All confidence intervals reported will be two-sided 95% confidence intervals.

6.3 Premature Withdrawal and Missing Data

For the primary and key secondary efficacy analyses, missing data will be imputed according to the estimand strategy utilized. For analyses using the treatment policy estimand, intercurrent events (ICEs) will not be utilized to exclude data from analyses. Missing data will be assumed to be missing at random (MAR) and will be imputed using multiple imputation (MI) within group defined by randomised treatment group and treatment status at week 48. Details of the multiple imputation approach for the primary estimand are provided in Section 10.1.6. Sensitivity analyses will be conducted to test the degree to which departures from the MAR assumption influence the conclusions for the primary endpoint using the treatment policy estimand only. The sensitivity analyses are described in Section 10.2. For analyses using the trial product/hypothetical estimand,

which will take into consideration impacts of ICEs on interpretability of data, ICEs will be utilized. Missing data or data impacted by ICEs will be assumed to be missing at random (MAR) and will be handled by the Mixed Model with Repeated Measures (MMRM).

All other efficacy analyses, as well as disposition, demographic, and safety analyses will be completed based on available data and no imputation algorithms will be applied for missing data unless otherwise specified. All data for subjects who withdraw early from the study will be used for these analyses up to the point of their discontinuation including data after subjects withdraw from treatment (retrieved drop out data).

6.4 Common Derivations

6.4.1 First Day of Study Drug Administration(TR01SDT)

The start date of semaglutide will be defined as the earliest 'Start date of dose' from the semaglutide dispensing page of the eCRF. This is because:

- Although the actual treatment start date of semaglutide is recorded in the participant eDiary, this date is missing for some participants.

- As participants take their first dose in the clinic, the planned start dose date of semaglutide from the dispensing page should be correct and complete for all participants.

The start date of bimagrumab/placebo will be defined as the earliest date of study drug administration from the study drug administration page of the eCRF.

The start date of first study drug will be defined as the start date of bimagrumab/placebo. It is expected that the start date of semaglutide and start date of bimagrumab/placebo will be the same since they are given on the same day in the clinic. The start date of first study drug is saved in variable TR01SDT in the ADAM datasets.

6.4.2 Last Day of Study Drug Administrationduring Core Study Period (TR01EDT)

The date of last study drug during the core study period will be equal to the first dose of study drug during the extension period i.e. the first administration or dispensing record on or after the date of Visit 16 (Week 52). If the participant has not taken a dose during the extension study period, the date of last study drug is defined as the latest of Visit 2 (Week 1) - Visit 15 (Week 48), the end of treatment visit, and the date of discontinuation of treatment from the completion page.

The end date of study drug during the core study period is saved in variable TR01EDT in the ADAM datasets.

6.4.3 First Day of Study Drug Administration during Extension Treatment Period (TR02SDT)

For participants who took extension study treatment, the start date of study drug during the extension study period (TR02SDT) is the same as the end date of study drug during the core study period (defined in Section 6.4.2).

6.4.4 Last Day of Study Drug Administrationduring Extension Treatment Period (TR02EDT)

For participants who took extension study treatment and completed Visit 21 (Week 72), the end date of study drug during the extension study period (TR02EDT) is the date of the Visit 21 (Week 72) visit.

For participants who took extension study treatment but did not complete Visit 21 (Week 72), the end date of study drug during the extension study period is the latest of Visit 16 (Week 52) - Visit 21 (Week 72), the end of treatment visit, and the date of discontinuation of treatment from the completion page.

6.4.5 Baseline

For all parameters presented by time point in the summary tables, baseline will be defined as the last, valid, non-missing assessment prior to study drug administration. This includes assessments that were recorded as unscheduled visits.

For DXA scans, baseline will be defined as last, evaluable assessment prior to the first study drug administration. This includes assessments that were recorded as unscheduled visits. Missing baseline will be imputed using the first post-baseline assessment if evaluable assessment available within 5 days after the first study drug administration. The number of patients with missing baseline due to unevaluable DXA scan who repeat scan after first dosing is expected to be low.

Study Days will be defined relative to the date of first study drug administration and will be used to calculate visit windows. Study Day 1 will be defined as the day of first study drug administration. The day before Study Day 1 is Study Day -1. Study Day 0 is not defined.

For assessments occurring on or after the date of first study drug administration:

Study Day = date of assessment - date of first study drug administration +1

For assessments occurring prior to the date of first study drug administration:

Study Day = date of assessment – date of first study drug administration

6.4.6 Core Study Completion

A subject is considered to have completed the core study period if the subject completes all 5 doses of bimagrumab/placebo per protocol.

6.5 Visit Window Conventions

Visit windows will be applied to all efficacy and safety assessments which will be summarised over time using the rules defined below. Unless otherwise specified, if there is more than one assessment within a visit window then the observation closest to the target day will be selected, or, if equidistant then the latter will be selected. All observations, including those excluded from the summary table as a result of this algorithm, will be included in the listings. Different windows are applied to the measures depending on the scheduled assessments for the measures.

6.5.1 Visit Windows for Body Weight including BIA, Wait Circumference, and Vital Signs

The visit windows applied to body weight including BIA, waist circumference, and vital signs are defined in Table 6.1.

Table 6.1. Visit Windows for Body Weight including BIA, Waist Circumference, and Vital Signs

Analysis Visit	Target Study Day	Visit Window (Study Days)
Baseline	Day 1 (Treatment Start Date)	≤1
Week 4	Day 29	>15 to ≤57
Week 12	Day 85	>57 to ≤99
Week 16	Day 113	>99 to ≤141
Week 24	Day 169	>141 to ≤183
Week 28	Day 197	>183 to ≤225
Week 40	Day 281	>253 to ≤309
Week 48	Day 337	>309 to ≤351

6.5.2 Visit Windows for DXA Scans

The visit windows applied to DXA scans are defined in Table 6.2.

As described in Section 6.4.1 as a few baseline DXA scans were unevaluable and had to be repeated, the visit window for Baseline DXA scans is up to 5 days after the date of first study drug administration. Conceptually, this is the same as choosing the closest DXA scan within 5 days of treatment start date if there is an unevaluable/missing baseline assessment.

Table 6.2 Visit Windows for DXA Scans

Analysis Visit	Target Study Day	Visit Window (Study Days)
Baseline	Day 1 (Treatment Start Date)	≤ 5
Week 12	Day 85	>57 to ≤113
Week 24	Day 169	>141 to ≤197
Week 48	Day 337	>309 to ≤365

6.5.3 Visit Windows for Grip Strength, QoL Questionnaires, PD Biomarkers, and Dietary Intake

The visit windows applied to grip strength, QoL questionnaire, PD biomarker, and Dietary Intake assessments are defined in Table 6.3.

Table 6.3. Visit Windows for Grip Strength, QoL Questionnaires, PD Biomarkers, and Dietary Intake

Analysis Visit	Target Study Day	Visit Window (Study Days)
Baseline	Day 1 (Treatment Start Date)	≤ 1*
Week 12	Day 85	>57 to ≤113
Week 24	Day 169	>141 to ≤197
Week 48	Day 337	>309 to ≤365

*Up to Study Day 5 allowed for grip strength and up to Study Day 14 allowed for dietary intake.

6.5.4 Visit Windows for ECG

The visit windows applied to ECG assessments are defined in Table 6.4.

Table 6.4. Visit Windows for ECG

Analysis Visit	Target Study Day	Visit Window (Study Days)
Baseline	Day 1 (Treatment Start Date)	≤ 1
Week 12	Day 85	>57 to ≤113
Week 48	Day 337	>309 to ≤365

6.5.5 Visit Windows for Hematology (CBC), Clinical Chemistry, Coagulation, and Safety Biomarkers

The visit windows applied to hematology, clinical chemistry, coagulation, and safety biomarker assessments are defined in Table 6.5.

Table 6.5. Visit Windows for Hematology (CBC), Clinical Chemistry, Coagulation, and Safety Biomarkers

Analysis Visit	Target Study Day	Visit Window (Study Days)
Baseline	Day 1 (Treatment Start Date)	≤1
Week 4	Day 29	>15 to ≤57
Week 12	Day 85	>57 to ≤113
Week 24	Day 169	>141 to ≤183
Week 28	Day 197	>183 to ≤225
Week 40	Day 281	>253 to ≤309
Week 48	Day 337	>309 to ≤365

6.5.6 Visit Windows for Urine Chemistry and Urinalysis

The visit windows applied to urine chemistry and urinalysis assessments are defined in Table 6.6.

Table 6.6. Visit Windows for Urine Chemistry and Urinalysis

Analysis Visit	Target Study Day	Visit Window (Study Days)
Baseline	Day 1 (Treatment Start Date)	≤1
Week 4	Day 29	>15 to ≤57
Week 12	Day 85	>57 to ≤113
Week 24	Day 169	>141 to ≤183
Week 28	Day 197	>183 to ≤225
Week 40	Day 281	>253 to ≤309
Week 48	Day 337	>309 to ≤365

6.5.7 Visit Windows for Anti-drug Antibodies

The visit windows applied to anti-drug antibodies assessments are defined in Table 6.7.

Table 6.7. Visit Windows for Anti-drug Antibodies

Analysis Visit	Target Study Day	Visit Window (Study Days)
Baseline	Day 1 (Treatment Start Date)	≤1
Week 4	Day 29	>15 to ≤57
Week 12	Day 85	>57 to ≤113
Week 24	Day 169	>141 to ≤183
Week 28	Day 197	>183 to ≤225
Week 48	Day 337	>309 to ≤365

6.5.8 Visit Windows for Pharmacokinetics

The visit windows applied to pharmacokinetic assessments are defined in Table 6.8.

Table 6.8. Visit Windows for Pharmacokinetics

Analysis Visit	Target Study Day	Visit Window (Study Days)
Baseline	Day 1 (Treatment Start Date)	≤1
Week 4	Day 29	>15 to ≤57
Week 12	Day 85	>57 to ≤99
Week 16	Day 113	>99 to ≤141
Week 24	Day 169	>141 to ≤183
Week 28	Day 197	>183 to ≤225
Week 48	Day 337	>309 to ≤365

6.5.9 Visit Windows for eDiary Assessments

The visit windows applied to eDiary assessments are defined in Table 6.9.

Table 6.9. Visit Windows for eDiary

Analysis Visit	Target Study Day	Visit Window (Study Days)
Baseline	Day 1 (Treatment Start Date)	≤1
Week 1	Day 8	>4 to ≤11
Week 2	Day 15	>11 to ≤18
Week 3	Day 22	>18 to ≤25
etc.		
Week *i*	Day (*i**7)+1	>(*i**7)- 3 to ≤(*i**7)+4
…		
Week 47	Day 330	>326 to ≤333
Week 48	Day 337	>333 to ≤340

6.5.10 Visit Windows for Actigraphy

The date of the weight assessments at study visits will be used to assign daily actigraphy summaries for each subject to visit windows as defined in Table 6.8. For step-count, the average of the daily summaries within the visit window will be used as the result for that analysis visit. For daily minutes of physical activity, the sum of the daily summaries within the visit window will be used as the result for that visit. For Baseline the assessments on study day 1 will not be included in the visit window as participants will receive the actigraphy device during their first clinic visit on day 1. This means they will not have a full 24 hours of activity recorded on day 1.

Table 6.10. Visit Windows for Actigraphy

Analysis Visit	Target Study Day	Visit Window
Baseline	Day 1 (Treatment Start Date)	7 days following the date of the Baseline weight assessment
Week 4	Day 29	7 days prior to the date of the Week 4 weight assessment
Week 12	Day 85	7 days prior to the date of the Week 12 weight assessment
Week 16	Day 113	7 days prior to the date of the Week 16 weight assessment
Week 24	Day 169	7 days prior to the date of the Week 24 weight assessment
Week 28	Day 197	7 days prior to the date of the Week 28 weight assessment
Week 40	Day 281	7 days prior to the date of the Week 40 weight assessment
Week 48	Day 337	7 days prior to the date of the Week 48 weight assessment

6.6 Software

All analyses will be performed using SAS version 9.4 or later.

7. STATISTICAL CONSIDERATIONS

7.1 Statistical Tests

Unless otherwise specified, all statistical tests will be conducted at the two-sided 5% significance level.

7.2 Multiplicity Adjustments

No multiplicity adjustment will be performed for this study. All statistical tests will be performed at the two-sided 5% significance level.

7.3 Multi-center Studies

Since this is a multi-centre study, all statistical models will include country as a fixed effect.

7.4 Other Strata and Covariates

To decrease the chance of an imbalance in gender across treatment arms, randomization was stratified by gender. All statistical models will include gender as a stratification factor.

For all change from baseline analyses, the baseline values will be used as a covariate.

As mentioned in Section 7.3 country will be included in all statistical models as a fixed effect.

7.5 Subgroup Analyses

There are no planned subgroup analyses.

8. OUTPUT SHELLS

Template shells for the tables, listings, and figures are presented in a separate document. All outputs will be presented in landscape format.

9. STUDY POPULATION

9.1 Subject Disposition

The number and percentage of subjects who were included in each analysis population, who completed the core treatment period, who completed the core study period, and who completed each visit in the core period will be presented by treatment group. The reason

for early withdrawal from treatment and early withdrawal from study will be summarized by treatment group.

Data listings will be provided for screen failures, subject disposition, subjects prematurely discontinued from the study, and subjects excluded from analysis (with reasons for exclusion) by treatment group. Protocol deviations will be listed.

9.2 Protocol Deviations

The Protocol Deviation Management Plan (PDMP) details the process for capturing and reporting protocol deviations in this study.

A Protocol Deviation is defined as any nonconformance, divergence, or departure (whether planned or unplanned), irrespective of the degree of seriousness or potentially resulting consequences from the study design or procedures defined in the protocol.

Important Protocol Deviations are a subset of PDs that may significantly impact the completeness, accuracy and/or reliability of study data or that may significantly affect a participant’s rights, safety or well-being. Further details are provided in the PDMP.

A final list of important protocol deviations will be approved by the Sponsor, the Medical Monitor, and others as appropriate, and provided to McCloud Consulting Group after database lock. The final list will include date of deviation, date of identification, date resolved, category, deviation description, and mitigation/ comments. Important protocol deviations will be presented in the study listings for inclusion in the CSR. Important subject-level protocol deviations will also be summarized by treatment group and category using the ITT population.

9.3 Demographics and Baseline Characteristics

All demographic and baseline characteristic data recorded prior to study treatment will be listed by subject and treatment group.

The following demographics will be summarized by treatment group.

- Sex, age at informed consent, race, ethnicity
- Height, weight, BMI, waist circumference, fat mass (kg) from BIA, lean mass (kg) from BIA, percent body fat (%) from BIA, percent lean body mass (%) from BIA, fat mass (kg) from DXA, lean mass (kg) from DXA, percent body fat (%) from DXA, percent lean body mass (%) from DXA at baseline
- Duration of obesity/overweight in years

Age will be derived as Year of initial informed consent – Year of birth.

9.4 Medical History and Pre-existing Conditions

Medical history will be coded using the latest version of the MedDRA dictionary (refer to the Data Management Plan for the version number).

The number and percentage of subjects with past and current medical conditions will be summarized by treatment group. The summary will be presented separately for obesity related conditions and other conditions. Medical history will also be listed by subject and treatment group.

9.5 Prior and Concomitant Medications, and Procedures

Prior and concomitant medications will by coded using the latest WHO dictionary (refer to the Data Management Plan for the version number). Prior and concomitant surgeries and medical procedures will be coded using the latest version of the MedDRA dictionary (refer to the Data Management Plan for the version number).

The start and stop dates will be used to identify when a concomitant medication was taken during the study. The date of procedure will be used to identify whether a surgery/procedure was conducted during the study.

Prior and concomitant medications will be grouped by classification and preferred term (PT), and summarised by treatment group. Prior and concomitant medications will be

summarised separately. Prior and concomitant medications will be listed separately by treatment group and subject.

The same approach will be taken for procedures, including surgeries. Prior and concomitant procedures will be grouped by classification and preferred term (PT), and summarised by treatment group. Prior and concomitant surgeries/procedures will be summarised separately. Prior and concomitant procedures will be listed separately by treatment group and subject.

Prior medications are those medications that were stopped prior to first investigational product administration. Concomitant medications are medications that are taken at least once after investigational product administration. Medications stopping on the same day as investigational product administration will be considered as concomitant medications.

For partially missing dates, the following conventions will be used in order to classify the medications as prior or concomitant:

- If start date is completely missing, start date will not be imputed.
- If (year is present and month and day are missing) or (year and day are present and month is missing), set month and day to January 1.
- If year and month are present and day is missing, set day to first day of the specified month.
- If end date is completely missing, end date will not be imputed.
- If (year is present and month and day are missing) or (year and day are present and month is missing), set month and day to December 31.
- If year and month are present and day is missing, set day to last day of the specified month.

10. EFFICACY

All efficacy data will be listed by treatment group and participant using the ITT population.

10.1 Primary Efficacy

10.1.1 Primary Estimand – Treatment Policy Strategy

The treatment policy strategy will be used to quantify the average treatment effect of bimagrumab, semaglutide or a combination of bimagrumab with semaglutide relative to placebo after 48 weeks, in all randomized participants, regardless of adherence to treatment and regardless of intercurrent events. This strategy closely adheres to full analysis set (FAS) with an intention-to-treat (ITT) population.

The following expansion of this estimand strategy will quantify the average treatment effect of combination treatment of semaglutide with bimagrumab relative to each monotherapy of the same dosage strength after 48 weeks, in all randomized participants regardless of adherence to treatment and regardless of intercurrent events (ICE).

10.1.2 Primary Endpoint

The primary endpoint is mean absolute change from baseline in total body weight (kg) at Week 48.

10.1.3 Analysis of Primary Endpoint using Treatment Policy Strategy

The primary analysis will utilize an Analysis of Covariance (ANCOVA) model with total body weight change from baseline to week 48 as the dependent variable, country and gender as stratification factors, baseline total body weight as the baseline covariate, and treatment group as a predictor variable.

In accordance with the “treatment policy” approach for the primary endpoint, missing data in the primary analysis will be assumed to be missing at random (MAR) and will be imputed with multiple imputation using 100 imputed datasets separately within groups defined by randomised treatment and treatment status at Week 48. Details of the multiple imputation approach for the treatment policy estimand are provided in Section 10.1.6.

The primary comparisons of interest will be the difference in Least Squares means (LSMeans, means adjusted for the covariates in the model) at Week 48. The results will be presented as LSMeans and standard errors (SEs) for each of the treatment groups, as well as the LSMeans of treatment differences for all 36 possible pairwise treatment comparisons and their associated 95% confidence intervals.

In addition to the analysis results, the summary statistics for the actual total body weight values at Week 48 and change from baseline will be presented for each of the treatment groups.

10.1.4 Secondary Estimand – Trial Product (Hypothetical) Strategy

The “trial product policy (hypothetical)” strategy will be utilized to quantify the average treatment effect of bimagrumab, semaglutide or a combination of bimagrumab with semaglutide relative to placebo after 48 weeks, in all randomized participants who received at least one dose of study treatment, adhered to treatment and did not experience the ICEs defined below.

The following expansion of the trial product/hypothetical estimand will quantify the average treatment effect of combination treatment of semaglutide with bimagrumab relative to each monotherapy of the same dosage strength after 48 weeks, in all randomized participants who received at least one dose of study treatment, adhered to treatment and did not experience the ICE defined below.

The ICE for the trial product/hypothetical estimand is:

1. Permanent discontinuation of study drug

Primary efficacy endpoint data occurring after the date of an ICE will be excluded from the analysis using the trial product/hypothetical estimand.

Any time a participant permanently discontinues treatment, this will be considered an ICE and the date of the onset of the ICE will be the treatment discontinuation date. This is the

end date of study treatment during core treatment period (TR01EDT) defined in Section 6.4.2

10.1.5 Analysis of the Primary Endpoint Using the Trial Product/Hypothetical Strategy

The analysis of this estimand will use a Mixed Model with Repeated Measures (MMRM), with change from baseline to weeks 4, 12, 16, 24, 28, 40 and 48 in total body weight as the dependent variable, country and gender as stratification factors, baseline weight as a baseline covariate, week and treatment group as factors, and week by treatment group as an interaction variable. An unstructured covariance structure will be used to model the within-participant errors. Significance tests will be based on LS means and robust "Huber White" standard errors. If this analysis fails to converge, the following covariance structures will be tested in order: autoregressive, and compound symmetry. The first covariance structure that converges will be used.

In accordance with the "trial product (hypothetical)" approach for the primary endpoint, missing data in this estimand analysis will be assumed to be missing at random (MAR) and will be handled implicitly by the MMRM.

The primary comparisons of interest will be the difference in Least Squares means (LSMeans, means adjusted for the covariates in the model) at Week 48. The results will be presented as LSMeans and SEs) for each of the treatment groups, as well as the LSMeans of treatment differences for all 36 possible pairwise treatment comparisons and their associated 95% confidence intervals.

In addition to the analysis results, the summary statistics for the actual total body weight values at each of the visits up to Week 48 and change from baseline will be presented for each of the treatment groups. Plots of the modelled (MMRM) treatment group means and 95% Confidence Intervals over time will be produced.

10.1.6 Multiple Imputation of Missing Data for the Treatment Policy Estimand

For the treatment policy estimand, missing values will be assumed to be missing at random (MAR). Sensitivity analyses will be conducted to test the degree to which missing data and departure from the MAR assumption influence the conclusions. The sensitivity analyses are described in Section 10.2.

Missing values for the treatment policy estimand will be imputed using multiple imputation with 100 samples drawn. The MI will be performed separately within groups defined by randomised treatment and treatment status at Week 48, in total up to 18 groups (9 treatment groups for patients on-treatment at week 48, and 9 treatment groups for patients off-treatment at week 48). If there is insufficient retrieved drop out data to conduct the planned multiple imputation, then missing data will be imputed from the control group, as planned for the sensitivity analysis described in Section 10.2.

The analysis will be conducted using all subjects in the ITT population.

There are 3 stages to the multiple imputation process, as described below.

1. Imputation Phase

During this phase, 100 datasets will be created where missing values in any of the variables in the planned ANCOVA model (described in Section 10.1.3 will be imputed by randomly selecting data from an appropriate distribution.

All variables in the imputation model will be assumed to have a multivariate joint normal distribution and will be modelled as continuous variables. This includes categorical variables, which will be recoded from categorical variables with k levels to k-1 indicator variables.

A Markov Chain Monte Carlo (MCMC) procedure will be used, where missing data will be selected from a multivariate joint normal distribution conditional on the observed data. The imputation model will include the stratification factors of country and gender, baseline weight and week 48 total body weight.

The multiple imputation procedures will be implemented using PROC MI in SAS with a random seed of 4895420.

Imputed data will be used in the analysis as imputed, even if it seems to fall outside the plausible range. This is because post-imputation truncation of data can lead to biased estimates (Rodwell, Lee, Romaniuk, & Carlin, 2014).

2. Analysis Phase

The 100 imputed datasets will be analysed 100 times using the planned ANCOVA model described in Section 10.1.3.

3. Pooling Phase

The parameter estimates from the 100 analyses conducted in step 2 will be combined into a single set of statistics that reflect the uncertainty associated with the imputed values using PROC MIANALYZE in SAS. Using the parameter estimates and standard errors from the 100 imputed datasets, this procedure applies Rubin's (1987) rules to make valid univariate statistical inferences.

10.2 Sensitivity Analyses

Sensitivity analyses will be conducted to test the degree to which missing data and departure from the MAR assumption influence the conclusions for the analysis of the primary endpoint using treatment policy estimand.

One violation of the MAR assumption would be if the statistical behaviour of participants with missing data transitions to that of participants in the Placebo group. This scenario will be modelled using control based imputation approach. A similar multiple imputation approach for missing data will be applied as described in Section 10.1.6. However, under the control based approach, missing data for the treatment groups will be imputed based on the imputation model derived from the Placebo arm only. This approach will be a possible worst case of departure from the MAR assumption, and the study conclusions will

be examined under this scenario. The SAS code for the sensitivity analysis will be as follows:

```
proc mi data= <DATASET> nimpute=100 seed=4895420;
    class country sex trt01p;
    var sex country baseline week_48;
    monotone method=reg;
    mnar model (week_48 / modelobs=(trt01p='Placebo+No
Sema'));
run;
```

10.3 Secondary Efficacy

10.3.1 Secondary Efficacy Endpoints

The following sections set out the definitions and analyses for the secondary efficacy endpoints. The secondary efficacy endpoints are described in Table 10.1. These follow the order of secondary endpoints set out in the Protocol.

All secondary efficacy analyses will be conducted using the treatment policy estimand strategy. In addition the continuous secondary variables will also be analysed using the trial product/hypothetical estimand strategy.

Table 10.1. Secondary Efficacy Endpoints

**Objective**	**Endpoints**	**Type**
To assess treatment effect of bimagrumab, semaglutide, and bimagrumab in addition to semaglutide vs placebo on body weight	1. Percent change from baseline in body weight at 48 weeks	Continuous

To assess treatment effects on waist circumference	2. Mean absolute change from baseline in waist circumference (cm) at 48 weeks	Continuous
To assess treatment effects on fat mass, visceral adipose tissue (VAT), trunk fat mass, subcutaneous adipose tissue (SAT), and lean mass	3. Mean absolute change from baseline and percent change from baseline at 48 weeks in total body fat mass (kg and % body fat) by dual-energy x-ray absorptiometry (DXA). The percent change from baseline for fat mass at each nominal visit is defined as: (post baseline fat mass [kg] – baseline fat mass [kg]) / baseline fat mass [kg] * 100%.	Continuous
	4. Mean absolute change from baseline and percent change from baseline at 48 weeks in VAT (kg), SAT (kg) and trunk fat mass (kg) by DXA	Continuous
	5. Proportion of subjects at 48 weeks with the following reductions from baseline:<br>i. Waist circumference ≥ 5 cm, ≥ 10 cm, and ≥ 15 cm<br>ii. Body weight ≥ 5%, ≥ 10%, and ≥ 15%<br>iii. Fat mass ≥ 5%, ≥ 10%, ≥ 15%, ≥ 20%, ≥ 25% by DXA<br>iv. Fat mass ≥ 10% with < 5% decrease (or increase) in lean mass by DXA	Response rate

	6. Ratio of VAT/SAT ratio, and ratio of android/gynoid fat mass ratio at 48 weeks versus baseline by DXA	Ratio
	7. Percentage of weight loss due to fat mass or lean mass at 48 weeks by DXA	Continuous
	8. Mean absolute change from baseline and percent change from baseline at 48 weeks in fat mass (kg and % body fat) by bioelectrical impedance analysis (BIA)	Continuous
	9. Mean absolute change from baseline and percent change from baseline at 48 weeks in total body lean mass (kg and % body lean) by DXA and BIA, as well as appendicular lean mass, android fat mass, and gynoid fat mass by DXA	Continuous
To assess treatment effects on categorical classification of obesity	10. Proportion of subjects in each BMI or WHtR category at any visit up to 48 weeks.<br><br>The BMI categories are:<br>• Healthy weight: 18.5 kg/m^2 to 24.9 kg/m^2<br>• Overweight: 25 kg/m^2 to 29.9 kg/m^2<br>• Obesity class I: 30 kg/m^2 to 34.9 kg/m^2<br>• Obesity class II: 35 kg/m^2 to 39.9 kg/m^2<br>• Obesity class III: ≥ 40 kg/m^2<br>The WHtR categories are:<br>• < 0.5<br>• 0.5 – 0.59<br>• ≥ 0.6	Categorical

To assess treatment effects on glucose metabolishm	11. Mean change and percent change from baseline in HbA1c at 48 weeks	Continuous
To assess treatment effects on self-reported health status and weight-related quality of life	Change from baseline at 24 weeks and 48 weeks in:<br><br>12. Quality of Life Short Form 36 survey (SF-36) total score and physical functioning score<br><br>13. Impact of Weight on Quality of Life-Lite for Clinical Trials survey (IWQoL-Lite for CT) total score and physical function	Continuous

10.3.2 Analysis of Continuous Secondary Efficacy Endpoints

Most of the secondary endpoints are continuous.

Each continuous secondary efficacy endpoint will be analysed using a similar ANCOVA model and MI approach as was used for the analysis of the primary endpoint using the treatment policy estimand. The exception is the analysis of percentage of weight loss due to fat mass or lean mass at 48 weeks by DXA, which will be described in Section 10.3.3.

The ANCOVA will include change from baseline (or percent change from baseline) to week 48 as the dependent variable, country and gender as stratification factors, baseline value as the baseline covariate, and treatment group as a predictor variable.

The results will be presented as LSMeans and standard errors (SEs) for each of thetreatment groups, as well as the LSMeans of treatment differences for all 36 possible pairwise treatment comparisons, their associated 95% confidence intervals and p-values.

In addition to the analysis results, the summary statistics for absolute values at Week 48 and change from baseline will be presented for each of the treatment groups.

Each continuous secondary efficacy endpoint will also be analysed using a similar MMRM model as was used for the analysis of the primary endpoint using the trial product/hypothetical estimand.

The MMRM will include with change from baseline (or percent change from baseline) to weeks 4, 12, 16, 24, 28, 40 and 48 as the dependent variable, country and gender as stratification factors, baseline value as a baseline covariate, week and treatment group as factors, and week by treatment group as an interaction variable. An unstructured covariance structure will be used to model the within-participant errors. Significance tests will be based on LS means and robust "Huber White" standard errors. If this analysis fails to converge, the following covariance structures will be tested in order: autoregressive, and compound symmetry. The first covariance structure that converges will be used.

The primary comparisons of interest will be the difference in Least Squares means (LSMeans, means adjusted for the covariates in the model) at Week 48. The results will be presented as LSMeans and SEs) for each of the treatment groups, as well as the LSMeans of treatment differences for all 36 possible pairwise treatment comparisons and their associated 95% confidence intervals.

In addition to the analysis results, the summary statistics for the actual values at each of the visits up to Week 48 and change from baseline will be presented for each of the treatment groups. Plots of the modelled (MMRM) treatment group means and 95% Confidence Intervals over time will be produced.

10.3.3 Analysis of Percentage of Weight Loss due to Fat Mass or Lean Mass at 48 weeks by DXA

Percentage of weight loss due to fat mass or lean mass will be analysed using the Fat Loss Index (FLI). The FLI at Week *x* will be calculated as:

FLI at Week *x* =Fat Loss at Week *x*/(Fat Loss + Lean Loss at Week *x*) * 100%

Composite endpoints of >5kg Weight loss and FLI>70%, >80%, and >90% will be analysed.

No hypothesis testing or multiple imputation will be performed for these composite endpoints. The endpoints will be summarised using as observed data and the treatment policy estimand at each timepoint by treatment group. The number and frequency of participants meeting the composite endpoint at each timepoint will be presented. Missing data will be considered as not meeting the endpoint.

10.3.4 Analysis of Ratio Secondary Efficacy Endpoints

There are two ratio secondary endpoints - the ratio of VAT/SAT ratio at Week 48 versus Baseline, and the ratio of android/gynoid fat mass ratio at Week 48 versus Baseline.

The first endpoint can described by formula as below.

Let VSR_{48} denote the VAT/SAT ratio at Week 48

Let VSR_{BL} denote the VAT/SAT ratio at Baseline.

The endpoint is $VSR_{48}/VSR_{BL.}$

VAT/SAT ratio will be summarised descriptively by treatment group over time. The number of observations, arithmetic mean, standard deviation (SD), minimum, median, and maximum will be summarised, as well as the geometric mean, geometric SD and geometric coefficient of variation. The ratio of VAT/SAT ratio (Week 48 versus Baseline) will also be summarised descriptively in the same manner.

To assess the treatment effect, the log-transformed ratio of VAT/SAT ratio (Week 48 versus Baseline) will be analysed using an ANCOVA model. The ANCOVA will include log-transformed ratio of VAT/SAT ratio (Week 48 versus Baseline) as the dependent variable, centre and gender as stratification factors, log-transformed Baseline VAT/SAT ratio as a baseline covariate, and treatment group as a predictor variable.

The geometric means of the ratio of VAT/SAT (Week 48 versus Baseline) and 95% CIs will be estimated for each of the nine treatment groups based on the ANCOVA model described above, by exponentiating the estimated LSMeans and their CIs. The treatment effects and their 95% CIs will also be estimated from the same model as the ratio of geometric means, for all 36 possible pairwise treatment comparisons, by exponentiating the difference of estimated LSMeans and their associated 95% CIs and p-values.

The second ratio endpoint, the ratio of android/gynoid fat mass ratio at Week 48 versus Baseline, will be analysed in the same manner.

10.3.5 Analysis of Response Rate Secondary Efficacy Endpoints

The secondary efficacy response rate endpoints will be analysed using a logistic regression model with response as the dependent variable, country and gender as a stratification factors, and treatment group as a predictor variable. Odds ratios and 95% confidence intervals for the treatment effect will be presented.

The treatment effect will be tested for statistical significance using the logistic regression model. For each response rate endpoint, the null hypothesis ‘that there no difference between treatment groups in the odds of response’ will be tested against the alternative hypothesis ‘that the odds of response is different between treatment groups’. A two-sided Wald Type 3 Chi-squared test at the 5% significance level will be used.

For each response rate secondary efficacy variable, the number and percentage of responders at Week 48, will be presented by treatment group.

10.3.6 Analysis of Categorical Secondary Efficacy Endpoints

The categorical endpoints will be summarized at each time point by treatment group. The summary will show the number and percentage of subjects in each category, including missing category. Shift tables from baseline to Week 24, and to Week 48 will also be presented.

The treatment effect on distribution of category at week 48 will be analysed using the Chi-square test. The Chi-square test will be applied separately for each pairwise treatment comparison. In addition, a stacked bar chart will be presented, displaying the proportion of participants in each category at baseline, week 24 and week 48 for each treatment group.

10.4 Exploratory Analyses

10.4.1 Exploratory Endpoints

The exploratory endpoints of the study are:

- Change from baseline in PD biomarkers at 24 and 48 weeks. The PD biomarkers may include: TC, HDL, LDL and TG, insulin, leptin, adiponectin, hsCRP, FSH, estradiol, free testosterone, IL-6, IL-18, NGAL, CTX, BSAP, P1NP, activin A, GDF8, GDF11. FSH, estradiol, and free testosterone will be analyzed separately for males, pre-menopausal women and post-menopausal women.

- Change from baseline in grip strength using handgrip dynamometer (kg) at 24 and 48 weeks.

- Change from baseline in step count as assessed by Wrist Actigraphy at 24 and 48 weeks.

- Change from baseline in total minutes of physical activity in the last 7 days as assessed by Wrist Actigraphy at 24 and 48 weeks.

- Change from baseline in HOMA-IR and QUICKI at 24 and 48 weeks. The Homeostatic Model Assessment for Insulin Resistance (HOMA-IR) and the Quantitative Insulin-Sensitivity Check Index (QUICKI) are insulin resistance indexes based on fasting insulin and fasting glucose measurements (Matthews, et al., 1985) (Katz, et al., 2000), calculated as follows:

HOMA-IR = (fasting insulin) * (fasting glucose)/C where C is dependent upon units:

- If Units (uIU/L) * (mg/dL)/405

- If Units (uIU/L) * (mmol/L)/22.5 because the conversion from mg/dL to mmol/L is 1/18.
- If Units (pmol/L) * (mmol/L)/135 because the conversion from uIU/L to pmol/L is 6

QUICKI = 1 / [LOG10(fasting insulin, uIU/L) + LOG10(fasting glucose, mg/dL)]

If either lab parameter is in different units, they must be converted to these:

- To convert pmol/L to uIU/L divide by 6.
- To convert mmol/L to mg/dL multiple by 18.
- Absolute and % change from baseline in bone mineral density (BMD) at 24 and 48 weeks, in the following areas – total, spine, and hip (total and femoral neck).
- Bimagrumab PK parameters based on pre-dose (Ctrough) and post-dose levels during treatment
- Semaglutide PK parameters based on pre-dose (Ctrough) levels during treatment
- Association of change from baseline in lean mass and fat mass using DXA measurement versus BIA measurement.

10.4.2 Analysis of Exploratory Variables

For all continuous exploratory variables, summary statistics for the actual values at each of the visits up to Week 48, change from baseline, and percent change from baseline, will be presented for each of the treatment groups. Selected biomarker variables will be summarized by gender. Selected biomarker variables will be analyzed using MMRM and the hypothetical estimand, using the same approach as the continuous secondary endpoints. For step-count and physical activity, the visits to be included in the summary statistics table are: Baseline, Weeks 4, 8, 12, 16, 24, 28, 40, and 48 only. The ITT population will be used

for all exploratory variables except for PK parameters, for which the Safety population will be used. Further detail on PK parameters can be found in Section 12.

A scatterplot of lean mass by DXA versus BIA will be presented with a linear regression line and Pearson Correlation Coefficient. Similarly, a scatterplot of fat mass by DXA versus BIA will be presented with a linear regression line and Pearson Correlation Coefficient.

11. SAFETY

11.1 Exposure

11.1.1 Bimagrumab/Placebo Exposure

The total duration of exposure during the Core Study Period (weeks) and number of infusions during the Core Study Period will be summarized with descriptive statistics (n, mean, SD, median, minimum and maximum) by treatment group. A list of study drug treatment assignment will be provided.

Total duration of exposure during the core study period in weeks is defined as (TR01EDT-TR01SDT+1)/7, where TR01SDT and TR01EDT are defined in Sections 6.4.1 and 6.4.2.

The overall treatment compliance rate for bimagrumab/placebo infusions will be summarized by treatment group. A patient is considered compliant if at least 80% of the time prior to the permanent treatment discontinuation, the patient received planned dose per administration (i.e., a partial infusion does not count). The algorithm is described in further detail below.

Compliant during core study period = Yes if (number of times ‘Did participant receive the correct dose’ = ‘Y’ at Visits 2 to 15)/(planned number of doses during Visits 2 to 15) >= 80%.

Planned number of doses during the period TR01SDT to TR01EDT = 5 for core treatment period completers

Planned number of doses during the period TR01SDT to TR01EDT is based on last visit attended for core period discontinuers as described in Table 11.1:

Table 11.1 Planned Number of Bimagrumab/Placebo Doses for Participants who Discontinued Core Study Treatment

Last Visit Attended	Planned Bimagrumab/Placebo doses
VISIT 2 (Week 1)	1
VISIT 3 (Week 2)	1
VISIT 4 (Week 4)	2
VISIT 5 (Week 8)	2
VISIT 6 (Week 12)	2
VISIT 7 (Week 16)	3
VISIT 8 (Week 20)	3
VISIT 9 (Week 24)	3
VISIT 10 (Week 28)	4
VISIT 11 (Week 32)	4
VISIT 12 (Week 36)	4
VISIT 13 (Week 40)	5
VISIT 14 (Week 44)	5
VISIT 15 (Week 48)	5

11.1.2 Semaglutide Exposure

Semaglutide will be administered weekly by self-injection. Semaglutide dispensing is recorded in the eCRF whilst administration is self-reported in the participant eDiary.

The total duration of exposure during the Core Study Period (weeks) and number of injections during the Core Study Period will be summarized with descriptive statistics (n, mean, SD, median, minimum and maximum) by treatment group. A list of study drug treatment assignment will be provided.

Total duration of exposure is defined in Table 11.2 below. TR01SDT, TR01EDT, TR02SDT and TR02EDT are defined in Sections 6.4.1, 6.4.2, 6.4.3, and 6.4.4 respectively.

Table 11.2 Total Duration of Semaglutide Exposure (weeks)

Treatment Arm	Exposure during Core Study Period (weeks)	Exposure by time of Core Study Database Lock (weeks)
Placebo + No Sema/Bima 30mg/kg + No Sema	0	0
Bima 10mg/kg + No Sema/Bima 30mg/kg + No Sema	0	0
Bima 30mg/kg + No Sema/Bima 30mg/kg + No Sema	0	0

All other treatment arms	(TR01EDT-TR01SDT+1)/7	(TR01EDT-TR01SDT+1)/7 or (TR02EDT-TR01SDT+1)/7 if participant has entered the Extension Treatment Period

The overall treatment compliance rate for semaglutide injection will be summarized by treatment group. A patient is considered compliant if at least 80% of the time prior to the permanent treatment discontinuation, the patient reported receiving planned number of injections. The algorithm is described in further detail below.

Compliant during core study period for core period completers = Yes if (number of injections recorded in diary during the period TR01SDT to Visit 15 (Week 48 date)/48 >= 80%.

Compliant during core study period for core period discontinuers = Yes if (number of injections recorded in diary during the period TR01SDT to TR01EDT)/(planned number of doses during the period TR01SDT to TR01EDT) >= 80%.

Planned number of injections during the period TR01SDT to TR01EDT for core period discontinuers = [(TR01EDT - TR01SDT+1)/7]+1 rounded down to the nearest integer

11.2 Adverse Events

Adverse events will be coded using the most recent version of the Medical Dictionary for Regulatory Activities (MedDRA) coding system to give a System Organ Class (SOC) and Preferred Term (PT) for each adverse event (AE).

Treatment emergent AEs will be defined as AEs with an onset date on or after the date of administration of study drug. If the onset date is missing, the AE is regarded as treatment emergent. Only treatment-emergent AEs that onset during the core study period will be summarized. All reported AEs will be listed.

The number of AEs, and the number and percentage of subjects reporting at least one AE will be summarized by PT nested within SOC for each of the following AE types:

1. Any AE
2. Any adverse events of special interest (AESI) (defined in Section 11.2.1)
3. Any AE related to bimagrumab only
4. Any AE related to semaglutide only
5. Any AE related to both bimagrumab and semaglutide
6. Any severe AE
7. Any serious AE (SAE)
8. Any serious AE related to bimagrumab only
9. Any serious AE related to semaglutide only
10. Any serious AE related to both bimagrumab and semaglutide

In addition, TEAEs and AESIs will be summarized by severity (mild, moderate, severe). Summary tables by severity will be split into two tables. The first displaying number and percentage of subjects, and the second displaying the number of events. For the first table of subjects, each subject will only count once in the maximum severity of the same PT. Similarly, each subject will only count once in the maximum severity of the same SOC,

and for the count of subjects with ‘at least one AE’, each subject will only count once in the maximum severity of any AE they experienced.

For the second table displaying number of events, all events will be included. This includes subjects with multiple occurrences of the same adverse event at different severities.

The following listings will be presented by treatment group and subject:

1. Any AEs
2. Any SAEs
3. Any related AEs

11.2.1 Adverse Events of Special Interest (AESI)

Adverse events of special interest (AESI) for bimagrumab are defined on the basis of an ongoing review of all safety data and include (using CTCAE intensity grading).

AESI will be identified programmatically using MedDRA terms rather than using the AESI flag from the eCRF. TEAEs which meet the criteria in Appendix 2 (Table 16.2) will be selected as AESI. The skin and subcutaneous tissue disorders identified in Appendix 2 (Table 16.3) will be excluded and will not be classified as AESI.

11.2.2 Adjudicated Adverse Events

An adjudication committee called the Clinical Event Committee (CEC) was formed to adjudicate potential events that occur during the course of a trial. Members of the CEC will adjudicate each potential event per study specific protocol, based on the pre-specified definitions, and render an assessment as to whether the case represents a confirmed event (meeting the event definition with all necessary documentation), a non-event (does not meet the event definition and likely represents an alternative or non-event diagnosis), or lacks sufficient documentation for confirmation of an event. Events are reviewed on an on-going process as cases become available. Events reported in the AE CRF will not be changed. However, adjudication may conclude that an event is not consistent with the AE

CRF. Adjudicated AEs will be listed only. For purposes of summarizing AEs, only AE CRF data will be included in the summary tables. AE CRF data will also be listed.

11.3 Laboratory Evaluations

The following clinical laboratory tests will be analysed at the local laboratory at times stipulated in the Protocol Schedule of Assessments:

- Biochemistry (blood sample): Sodium, potassium, chloride, bicarbonate (CO2), phosphate, magnesium, calcium, albumin, total protein, uric acid, urea/blood urea nitrogen (BUN), creatinine, glucose, creatine kinase (CK), lipase, amylase, liver function tests (AST / SGOT, ALT/ SGPT, LDH, GGT, alkaline phosphatase), total and direct bilirubin.

- Hematology (blood sample): Hemoglobin, hematocrit, red blood cell (RBC) count, RBC indices (mean corpuscular volume (MCV), mean corpuscular hemoglobin (MCH), mean corpuscular hemoglobin concentration (MCHC), reticulocytes (% and absolute)), white blood cell (WBC) count with differential (% and absolute) (neutrophils, lymphocytes, monocytes, eosinophils, basophils), erythrocyte sedimentation rate, platelet count.

- Coagulation (blood sample): aPTT, PT, INR.

- Urinalysis: pH, specific gravity, glucose, protein, bilirubin, nitrite, leukocytes, blood, ketones. Microscopy will be carried out if blood or protein is abnormal.

- Urine Chemistry: Microalbumin, urine creatinine, ACR.

- Lipid profile (blood sample): Total cholesterol, triglycerides, HDL cholesterol, LDL cholesterol. Samples will be collected under fasting conditions unless specified otherwise.

- Glucose metabolism parameters (blood sample): HbA1C, insulin, glucose. Samples will be collected under fasting parameters except for the screening visit.

- Pregnancy Screen: All women will be screened for pregnancy. The screening pregnancy test will be by blood sample to detect the presence of β-human chorionic gonadotropin (β-HCG). Subsequent visits will utilize rapid urine hCG tests for pregnancy.

- Urine drug screen: to include at minimum amphetamines, barbiturates, cocaine, opiates, cannabinoids and benzodiazepines.

- Hepatitis B and HIV screening: HIV antibody, HBsAg, HBsAb, HBcAb, HCVAb.

Clinical safety laboratory data (hematology, biochemistry, and urine chemistry) will be standardised to SI units. The normal range for the standardised data will be data dependent as follows:

Standardized Lower Limit of Normal (LLN) for a parameter = minimum LLN for that parameter in the standardized dataset

Standardized Upper Limit of Normal (ULN) for a parameter = maximum ULN for that parameter in the standardized dataset

Clinical safety laboratory data (hematology, biochemistry, and urine chemistry) will be summarised at each protocol scheduled time point, by treatment group. Actual values and actual changes from baseline will be presented. Continuous urinalysis parameters will be similarly summarized. Categorical urinalysis parameters will be summarized using frequencies and percentages at each protocol scheduled time point by treatment group. For the summary of categorical urinalysis results, the results will be consolidated to the categories displayed in Appendix 16.3 Table 16.4.

The following plots will also be presented for all biochemistry and urine chemistry parameters:

- For selected laboratory parameters, scatterplots of shift from baseline to "worst post baseline" value with reference lines for normal ranges will be presented. The

"worst" value will be defined as either the lowest or highest post-baseline value. For some parameters, both the highest and lowest values will be considered the "worst" and the scatterplot will be presented twice. The selected parameters and definition of worst are detailed in Section 16 Appendix 1 (Table 16.1). For these scatterplots, the most frequent normal range will be used as the standard normal range, and all results will be linearly transformed from their original units and original normal range so they fall in a similar relative position within the standard normal range, compared to their original position within their original normal range. Further details of the transformation algorithm are described in Section 16 Appendix 1.

- Graphs of mean change from baseline values by treatment group over time.

Urinalysis results evaluation (normal, abnormal not clinically significant or abnormal clinically significant) will be summarised at each protocol scheduled time point, by treatment group, using frequency tabulations.

Abnormal laboratory values will be flagged and will be identified in the listings. Microscopy data, if available, will be listed.

All laboratory results except drug screening will be listed. Drug screening results are kept as source data only.

11.4 Vital Signs

Vital signs measurements will include body temperature, pulse rate and systolic and diastolic blood pressure. Vital signs will be collected at the times stipulated in the Protocol Schedule of Assessments.

Vital sign results will be summarised at each protocol scheduled time point, by treatment group. Actual values and actual changes from baseline will be presented. Vital sign results will be listed by treatment group and subject.

Height will be presented as part of the demographic and baseline assessments.

Weight and body mass index (BMI) will be presented as part of efficacy assessments.

11.5 12-lead ECG

Standard 12-lead ECG (PR, RR, QRS, QT and QTcF) will be performed at times stipulated in the Protocol Schedule of Assessments.

Electrocardiogram evaluation (normal, abnormal not clinically significant or abnormal clinically significant) will be summarised at each protocol scheduled time point, by treatment group at each protocol scheduled time point, using frequency tabulations. Actual values and actual changes from baseline will be presented.

ECG results will also be listed by treatment group and subject.

11.6 Physical Examination

A physical examination will be performed at times stipulated in the Protocol Schedule of Assessments.

Physical examination findings will be listed by subject and visit.

11.7 Dietary Intake

Participants' dietary intake will be recorded via 24-hour recall assessment at Baseline, Week 12, 24, and 48. Dietary intake will include Total Calories (kcal/day), Carbohydrate (g/day), Carbohydrate Percentage by Total Calories (% total calories), Fat (g/day), Fat Percentage by Total Calories (% total calories), Protein (g/kg body weight/day), and Protein Percentage by Total Calories (% total calories).

The dietary intake parameters will be summarised at time point, by treatment group. Actual values and actual changes from baseline will be presented. Dietary intake will be listed by treatment group and participant.

Protein intake is collected in units of g/day and will be converted to g/kg body weight/day using the following algorithm (MacCarron & Devine, 1974):

1. Calculate ideal body weight (IBW) using the following formula:
    - Male: IBW = 50 kg + 2.3*(Actual height in cm/2.54 – 60 inches)
    - Female: IBW = 45.5 kg + 2.3 kg*(Actual height in cm/2.54 – 60 inches)
2. Compare actual weight to IBW
    - Actual weight = X% of IBW
3. If actual weight is >30% above IBW, then calculate adjusted body weight (1-3) for use in converting g/day to g/kg body weight/day
    - Adjusted body weight = IBW + 0.4* (Actual weight – IBW)
4. Convert protein intake (g/day) to protein intake (g/kg body weight/day)
    - If actual weight > 1.3*IBW, protein intake (g/kg body weight/day) = protein intake (g/day) / adjusted body weight
    - If actual weight ≤1.3*IBW, protein intake (g/kg body weight/day) = protein intake (g/day) / actual body weight

12. PHARMACOKINETICS

Pharmacokinetics (PK) biomarkers include blood serum concentration of bimagrumab and blood plasma concentration of semaglutide, which will be evaluated for all subjects at times specified by the Protocol Schedule of Assessments. For bimagrumab, samples will be collected pre-dose and 15 min post dose completion on specified dosing days, and as convenient at the Week 48 visit. For semaglutide, samples will be collected pre-dose on

specified visits and as convenient at the Week 48 visit. Results will not be reported to blinded study personnel until after the study has been unblinded.

Data will be summarized for each subject and treatment group using graphs and tables. Results, change from baseline, and percent change from baseline will be analyzed by treatment group using descriptive statistics including mean, SD, median, minimum, and maximum. PK biomarkers will be listed by treatment group and subject ID.

13. PHARMACODYNAMICS

Pharmacodynamic (PD) biomarkers may include, but are not limited to, those listed below. Samples will be collected at times stipulated by the Protocol Schedule of Assessments and measured by the central laboratory.

- Adipokines: leptin, adiponectin, adipsin
- Inflammation biomarkers: IL-6, IL- 18, hsCRP, NGAL
- Endocrine biomarkers: FSH, estradiol, free testosterone, insulin, HbA1c
- Bone turnover biomarkers: BSAP, CTX-1, P1NP
- TGF-β superfamily biomarkers: activins, GDFs (specific tests to be determined)
- Safety biomarkers: ALP/amylase/ lipase isoenzymes, 5’NT, miR-122 and CCK18

Analysis of ALP/amylase/lipase isozymes and miR-122/CCK18 will only be performed for subjects with abnormal local lab results.

Data will be summarized for each subject and treatment group using graphs and tables. Results, change from baseline, and percent change from baseline will be analyzed by treatment group using descriptive statistics including mean, SD, median, minimum, and maximum. All PD biomarker data will be listed by treatment group and subject ID.

14. IMMUNOGENICITY

Serum samples from all subjects collected according to the SoA will be screened for antibodies binding to bimagrumab (anti-drug antibodies, ADA) and the titer of confirmed positive samples will be reported. Other analyses may be performed to evaluate the neutralizing activity or verify the stability of antibodies to bimagrumab and/or further characterize the immunogenicity of bimagrumab.

ADA results will be summarised at each protocol scheduled time point, by treatment group. Actual values and actual changes from baseline will be presented. ADA results will be listed by treatment group and subject.

15. CHANGES FROM PROTOCOL

Not applicable.

16. APPENDICES

16.1 Appendix 1: Details of Laboratory scatterplots

As described in Section 11.3, for selected laboratory parameters, scatterplots of shift from baseline to “worst post baseline” value with reference lines for normal ranges will be presented. Table 16.1 lists the parameters to be plotted along with the definition of the “worst” value as either the lowest or highest post-baseline value, or both. The scatterplot will be presented twice if both lowest and highest are considered “worst”.

Table 16.1 Details of Laboratory Scatterplots for Selected Parameters

Type of Laboratory Test	Parameter	Definition of "Worst"	
		Lowest Post-Baseline Value	Highest Post-Baseline Value
Chem	Calcium	x	X
Chem	Glucose	x	X

Chem	BUN/urea		x
Chem	Uric Acid	x	x
Chem	Protein	x	
Chem	Albumin	x	
Chem	Potassium	x	x
Chem	Sodium	x	x
Chem	Total Bilirubin		x
Chem	Direct Bilirubin		x
Chem	Alk.phos.	x	x
Chem	LDH		x
Chem	AST		x
Chem	ALT		x
Chem	GGT		x
Chem	Creatinine Kinase	x	x
Chem	Creatinine	x	x
Chem	Magnesium	x	x
Chem	Amylase	x	x
Chem	Lipase	x	x
Chem	WBC	x	x
Chem	RBC	x	x
Hem	Hemoglobin	x	x
Hem	Abs.reticulocyte count		x
Hem	Abs. Neutrophils	x	x
Hem	Abs. Lymphocytes.	x	x
Hem	Abs. Eosinoph.		x
Hem	Platelets	x	x
Hem	Sedimentation rate		x
Hem	aPTT	x	x
Hem	Prothrombin time	x	x
Hem	INR		x
**Total Figures**		**21**	**30**

For parameters with multiple normal ranges, the different units and normal ranges were transformed to the same standard units and standard normal range using the linear transformation described below. This transformation was applied to the values in the

laboratory scatterplots only. It preserves the relationship between the original laboratory values and their original normal ranges and the relationship between the standardized values and the standardized reference range. If the original value lies within the original normal range then the standardized value will also lie within the standardized normal range. For original values that fall outside the original normal range the standardized value will also fall outside the standardized normal range, and will approximately maintain the relationship between values and xULN.

Let L_v denote the original value, and (L_{LLN}, L_{ULN}) denote the original normal range.

Let S_v denote the standardized value, and (S_{LLN}, S_{ULN}) denote the standardized normal range. The linear transformation is

$$S_v = S_{LLN} + \frac{(L_v - L_{LLN})}{(L_{ULN} - L_{LLN})} \times (S_{ULN} - S_{LLN})$$

Examples:

Suppose the original ranges for a parameter are (32, 182), (41, 331), and (49, 439). Suppose the standardized normal range is (45, 300).

Suppose the first original result is L_v=32 which is the LLN for the first original normal range, then

$$S_v = 45 + \frac{(32 - 32)}{(182 - 32)} \times (300 - 45)$$

$$= 45 = S_{LLN}$$

which maintains the result's relative position at the lower limit of the normal range.

Suppose the second original result is L_v=107 which is the mid-point of the first original normal range, then

$$S_v = 45 + \frac{(107 - 32)}{(182 - 32)} \times (300 - 45)$$

$$= 45 + \frac{75}{150} \times (300 - 45)$$

$$= 45 + 127.5 = 172.5$$

which is the mid-point of the standardised normal range.

Suppose the third original result is L_v=182 which is the ULN for the first original normal range, then

$$S_v = 45 + \frac{(182 - 32)}{(182 - 32)} \times (300 - 45)$$

$$= 45 + (300 - 45) = 300 = S_{ULN}$$

which maintains the result's relative position at the upper limit of the normal range.

Suppose as fourth original result is L_v=2×182 which is 2xULN for the first original normal range, then

$$S_v = 45 + \frac{(364 - 32)}{(182 - 32)} \times (300 - 45)$$

$$= 45 + 2.213 \times (300 - 45) = 609.4 \sim 2 \times S_{ULN}$$

which maintains the result's relative position at approximately twice the upper limit of the normal range.

16.2 Appendix 2: Details Of MedDRA Terms Used to Define AESI

Table 16.2 MedDRA Terms Used to Define AESI

AESI Category	MedDRA Level	Term	Term Code	Minimum Grade
Muscle related events	HLGT	Muscle disorders	10028302	3
	HLT	Musculoskeletal and connective tissue pain and discomfort	10068757	3
	PT	Musculoskeletal stiffness	10052904	3
Skin-related lesions excluding rashes defined in Table 6	BODSYS	Skin and subcutaneous tissue disorders	10040785	3
Gastrointestinal events	HLGT	Gastrointestinal conditions NEC	10017943	3
	HLT	Gastrointestinal inflammatory disorders NEC	10017921	3
	HLGT	Gastrointestinal motility and defaecation conditions	10017977	3
	HLGT	Gastrointestinal signs and symptoms	10018012	3
Pancreatitis	HLGT	Exocrine pancreas conditions	10015674	Any grade
Malignancy	HLGT	Breast neoplasms malignant and unspecified (incl nipple)	10006291	Any grade
	HLGT	Endocrine neoplasms malignant and unspecified	10014713	Any grade
	HLGT	Gastrointestinal neoplasms malignant and unspecified	10017991	Any grade
	HLGT	Haematopoietic neoplasms (excl leukaemias and lymphomas)	10018865	Any grade
	HLGT	Hepatobiliary neoplasms malignant and unspecified	10019815	Any grade
	HLGT	Leukaemias	10024324	Any grade
	HLGT	Lymphomas Hodgkin's disease	10025319	Any grade
	HLGT	Lymphomas NEC	10025323	Any grade
	HLGT	Lymphomas non-Hodgkin's B-cell	10025320	Any grade
	HLGT	Lymphomas non-Hodgkin's T-cell	10025321	Any grade
	HLGT	Lymphomas non-Hodgkin's unspecified histology	10025322	Any grade

	HLGT	Mesotheliomas	10027412	Any grade
	HLGT	Metastases	10027476	Any grade
	HLGT	Miscellaneous and site unspecified neoplasms malignant and unspecified	10027655	Any grade
	HLGT	Nervous system neoplasms malignant and unspecified NEC	10029211	Any grade
	HLGT	Plasma cell neoplasms	10035227	Any grade
	HLGT	Renal and urinary tract neoplasms malignant and unspecified	10038364	Any grade
	HLGT	Reproductive and genitourinary neoplasms gender unspecified NEC	10038588	Any grade
	HLGT	Reproductive neoplasms female malignant and unspecified	10038594	Any grade
	HLGT	Reproductive neoplasms male malignant and unspecified	10038597	Any grade
	HLGT	Respiratory and Mediastinal neoplasms malignant and unspecified	10038666	Any grade
	HLGT	Skeletal neoplasms malignant and unspecified	10040778	Any grade
	HLGT	Skin neoplasms malignant and unspecified	10040900	Any grade
	HLGT	Soft tissue neoplasms malignant and unspecified	10072990	Any grade
	HLT	Ocular melanomas	10030052	Any grade
	HLT	Ocular neoplasms malignancy unspecified	10030053	Any grade
	HLT	Ocular neoplasms malignant (excl melanomas)	10030057	Any grade

Table 16.3 MedDRA Terms within the SOC, 'Skin and Subcutaneous Tissue Disorders' Which Will Not Be Identified as AESI

Code	Preferred Term	HLT	HLGT
10037844	Rash	Rashes, eruptions and exanthems NEC	Epidermal and dermal conditions
10067982	Butterfly rash	Rashes, eruptions and exanthems NEC	Epidermal and dermal conditions

10064579	Exfoliative rash	Exfoliative conditions	Epidermal and dermal conditions
10081454	Heliotrope rash	Rashes, eruptions and exanthems NEC	Epidermal and dermal conditions
10056671	Mucocutaneous rash	Rashes, eruptions and exanthems NEC	Epidermal and dermal conditions
10075807	Nodular rash	Rashes, eruptions and exanthems NEC	Epidermal and dermal conditions
10074687	Paraneoplastic rash	Rashes, eruptions and exanthems NEC	Epidermal and dermal conditions
10037855	Rash erythematous	Rashes, eruptions and exanthems NEC	Epidermal and dermal conditions
10037857	Rash follicular	Pustular conditions	Epidermal and dermal conditions
10037867	Rash macular	Rashes, eruptions and exanthems NEC	Epidermal and dermal conditions
10037868	Rash maculo-papular	Rashes, eruptions and exanthems NEC	Epidermal and dermal conditions
10050004	Rash maculovesicular	Rashes, eruptions and exanthems NEC	Epidermal and dermal conditions
10037870	Rash morbilliform	Rashes, eruptions and exanthems NEC	Epidermal and dermal conditions
10037871	Rash neonatal	Rashes, eruptions and exanthems NEC	Epidermal and dermal conditions
10037876	Rash papular	Rashes, eruptions and exanthems NEC	Epidermal and dermal conditions
10037879	Rash papulosquamous	Papulosquamous conditions	Epidermal and dermal conditions
10037884	Rash pruritic	Rashes, eruptions and exanthems NEC	Epidermal and dermal conditions
10057984	Rash rubelliform	Rashes, eruptions and exanthems NEC	Epidermal and dermal conditions
10037890	Rash scarlatiniform	Rashes, eruptions and exanthems NEC	Epidermal and dermal conditions
10037898	Rash vesicular	Rashes, eruptions and exanthems NEC	Epidermal and dermal conditions
10042946	Systemic lupus erythematosus rash	Rashes, eruptions and exanthems NEC	Epidermal and dermal conditions
10047111	Vasculitic rash	Skin vasculitides	Skin vascular abnormalities

16.3 Appendix 3: Consolidation of Urinalysis Results Categories

For the summary of categorical urinalysis results, the results will be consolidated in the categories displayed in Table 16.4.

Table 16.4 Consolidation of Urinalysis Results Categories

**Parameter Short Code (ADLB.PARAMCD)**	**Parameter Result (ADLB.AVALC)**	**Consolidate to**	**Order**
BILU	1+	1+	1
BILU	SMALL (1+)		
BILU	MODERATE (2+)	2+	2
BLD	NEGATIVE	Negative	-1
BLD	HAEMOLYSED-TRACE	Trace - hemolyzed	0.5
BLD	NON-HAEMOLYSED-TRACE	Trace - non-hemolyzed	
BLD	TRACE	Trace	
BLD	1+	1+	1
BLD	SMALL (1+)		
BLD	2+	2+	2
BLD	MODERATE (2+)		
BLD	NON-HAEMOLYSED-MODERATE	Moderate - non-hemolyzed	
BLD	3+	3+	3
BLD	LARGE (3+)		

KETONES	1+	1+	1
KETONES	SMALL		
KETONES	2+	2+	2
KETONES	MODERATE		
KETONES	LARGE	3+	3

17. REFERENCES

Benjamini, Y., & Hochberg, Y. (1995). Controlling the False Discovery Rate: A Practical and Powerful Approach to Multiple Testing. *Journal of the Royal Statistical Society Series B (Methodological), 57*(1), 289-300.

Jastreboff, A. M., Aronne, L. J., Ahmad, N. N., Wharton, S., Connery, L., Alves, B., . . . Stefanski, A. (2022). Tirzepatide Once Weekly for the Treatment of Obesity. *New England Journal of Medicine, 387*, 205-216.

Katz, A., Nambi, S. S., Mather, K., Baron, A. D., Follman, D. A., Sullivan, G., & Quon, M. J. (2000). Quantitative insulin sensitivity check index: a simple, accurate method for assessing insulin sensitivity in humans. *The Journal of Clinical Endocrinology & Metabolism, 85*(7), 2402-10.

MacCarron, M., & Devine, B. (1974). Clinical Pharmacy: Case Studies: Case Number 25 Gentamicin Therapy. *Drug Intelligence & Clinical Pharmacy, 8*(11), 650-655. doi:10.1177/106002807400801104

Matthews, D. R., Hosker, J. P., Rudenski, A. S., Naylor, B. A., Treacher, D. F., & Turner, R. C. (1985, July). Homeostasis model assessment: insulin resistance and beta-cell function from fasting plasma glucose and insulin concentrations in man. *Diabetologia, 28*(7), 412-9.

Osborne, J. A. (2006). Estimating the False Discovery Rate using SAS (R). *SAS Users Group International.* San Francisco. Retrieved from https://support.sas.com/resources/papers/proceedings/proceedings/sugi31/190-31.pdf

Rodwell, L., Lee, K., Romaniuk, H., & Carlin, J. (2014). Comparison of methods for imputing limited-range variables: a simulation study. *BMC Medical Research*, 14-57.

Sean Wharton, A. A. (2021). Estimating and reporting treatment effects in clinical trials for weight management: using estimands to interpret effects of intercurrent events and missing data. *International Journal of Obesity*(45), 923-933. doi:https://doi.org/10.1038/s41366-020-00733-x

Wildman, R. P. (2009). Healthy obesity. *Current Opinion in Clinical Nutrition and Metabolic Care, 12*(4), 438-443.

Signature Page for VV-CLIN-136795 v2.0

Approval	PPD<br>10-Jul-2024 00:40:55 GMT+0000

Signature Page for VV-CLIN-136795 v2.0
