# Supplementary material for: Bimagrumab plus semaglutide alone or in combination for the treatment of obesity: a randomized phase 2 trial
Source: Nat Med. 2026 Mar 2;32(3):869–82. doi: 10.1038/s41591-026-04204-0 (PMC13004672; doi:10.1038/s41591-026-04204-0)
Supplement: Supplementary file 2 — Reporting Summary [file 41591_2026_4204_MOESM2_ESM.pdf]

nature portfolio

Corresponding author(s): Steven Heymsfield

Last updated by author(s): 12 December 2025

Reporting Summary

Nature Portfolio wishes to improve the reproducibility of the work that we publish. This form provides structure for consistency and transparency in reporting. For further information on Nature Portfolio policies, see our Editorial Policies and the Editorial Policy Checklist.

Statistics

For all statistical analyses, confirm that the following items are present in the figure legend, table legend, main text, or Methods section.

n/a	Confirmed	
☐	☒	The exact sample size (*n*) for each experimental group/condition, given as a discrete number and unit of measurement
☒	☐	A statement on whether measurements were taken from distinct samples or whether the same sample was measured repeatedly
☐	☒	The statistical test(s) used AND whether they are one- or two-sided<br>*Only common tests should be described solely by name; describe more complex techniques in the Methods section.*
☐	☒	A description of all covariates tested
☐	☒	A description of any assumptions or corrections, such as tests of normality and adjustment for multiple comparisons
☐	☒	A full description of the statistical parameters including central tendency (e.g. means) or other basic estimates (e.g. regression coefficient) AND variation (e.g. standard deviation) or associated estimates of uncertainty (e.g. confidence intervals)
☐	☒	For null hypothesis testing, the test statistic (e.g. *F*, *t*, *r*) with confidence intervals, effect sizes, degrees of freedom and *P* value noted<br>*Give P values as exact values whenever suitable.*
☒	☐	For Bayesian analysis, information on the choice of priors and Markov chain Monte Carlo settings
☒	☐	For hierarchical and complex designs, identification of the appropriate level for tests and full reporting of outcomes
☒	☐	Estimates of effect sizes (e.g. Cohen's *d*, Pearson's *r*), indicating how they were calculated

Our web collection on statistics for biologists contains articles on many of the points above.

Software and code

Policy information about availability of computer code

Data collection	No specific software were used for data collection
Data analysis	All analyses will be performed using SAS version 9.4 or later

For manuscripts utilizing custom algorithms or software that are central to the research but not yet described in published literature, software must be made available to editors and reviewers. We strongly encourage code deposition in a community repository (e.g. GitHub). See the Nature Portfolio guidelines for submitting code & software for further information.

Data

Policy information about availability of data

All manuscripts must include a data availability statement. This statement should provide the following information, where applicable:

- Accession codes, unique identifiers, or web links for publicly available datasets
- A description of any restrictions on data availability
- For clinical datasets or third party data, please ensure that the statement adheres to our policy

Provide your data availability statement here.

Research involving human participants, their data, or biological material

Policy information about studies with human participants or human data. See also policy information about sex, gender (identity/presentation), and sexual orientation and race, ethnicity and racism.

Reporting on sex and gender	Randomization was stratified by gender across the treatment groups, and recruitment was controlled to ensure not more than 60% of participants of one gender. The participants self-reported their sex. Demographics provided in Table 1.
Reporting on race, ethnicity, or other socially relevant groupings	Demographics provided in Table 1.
Population characteristics	Population characteristics provided in Table 1.
Recruitment	Participants were recruited from United States, Australia, and New Zealand.
Ethics oversight	The trial adhered to Declaration of Helsinki, Council for International Organizations of Medical Sciences international ethical guidelines, and Good Clinical Practice guidelines. An independent ethics committee (New Zealand Southern Health and Disability Ethics Committee, New Zealand; Bellberry Limited Human Research Ethics Committee, Australia; and Austin Health Human Research Ethics Committee, Australia) or institutional review board (WCG Institutional Review Board, United States; and Pennington Biomedical Research Center Institutional Review Board, United States) for each site approved the protocol. All participants provided written informed consent before participation.

Note that full information on the approval of the study protocol must also be provided in the manuscript.

Field-specific reporting

Please select the one below that is the best fit for your research. If you are not sure, read the appropriate sections before making your selection.

☒ Life sciences ☐ Behavioural & social sciences ☐ Ecological, evolutionary & environmental sciences

For a reference copy of the document with all sections, see nature.com/documents/nr-reporting-summary-flat.pdf

Life sciences study design

All studies must disclose on these points even when the disclosure is negative.

Sample size	The planned sample size of 495 participants was estimated to provide over 80% statistical power to detect a difference between any active treatment group and placebo with respect to the primary endpoint using a two-sided t-test with significance level of 0.05. This assumed a minimum treatment effect of 5% weight reduction at Week 48 with a standard deviation of 8% and dropout rate of 20%.
Data exclusions	Described in the manuscript
Replication	This is a clinical study and measurements in participants of all treatment groups were not reassessed to check for replication. Details of randomization, blinding, procedures are provided in manuscript to facilitate replication of outcome measures.
Randomization	Participants were randomly assigned (1:1:1:1:1:1:1:1:1 ratio) to nine treatment groups (placebo; bimagrumab 10 or 30mg/kg IV Q12W; semaglutide 1.0 or 2.4mg SC QW or combinations thereof) using a centralized web-based interactive response system. Randomization was stratified by sex across the treatment groups.
Blinding	The participant, Investigator, and Sponsor were blinded to bimagrumab dose or placebo-bimagrumab until database lock to avoid bias in reporting adverse events and efficacy. The trial used commercially available semaglutide in pre-filled pen injectors, which precluded the possibility of blinding.

Reporting for specific materials, systems and methods

We require information from authors about some types of materials, experimental systems and methods used in many studies. Here, indicate whether each material, system or method listed is relevant to your study. If you are not sure if a list item applies to your research, read the appropriate section before selecting a response.

Materials & experimental systems

n/a	Involved in the study	
☒	☐	Antibodies
☒	☐	Eukaryotic cell lines
☒	☐	Palaeontology and archaeology
☒	☐	Animals and other organisms
☐	☒	Clinical data
☒	☐	Dual use research of concern
☒	☐	Plants

Methods

n/a	Involved in the study	
☒	☐	ChIP-seq
☒	☐	Flow cytometry
☒	☐	MRI-based neuroimaging

Clinical data

Policy information about clinical studies

All manuscripts should comply with the ICMJE guidelines for publication of clinical research and a completed CONSORT checklist must be included with all submissions.

Clinical trial registration	ClinicalTrials.gov number NCT05616013
Study protocol	Provided in the supplementary material
Data collection	The trial conducted at 26 sites in United States, Australia, and New Zealand. Data for the results presented in the manuscript was collected from November 2022 - November 2024.
Outcomes	The primary endpoint was absolute change from baseline in body weight at Week 48. The secondary and exploratory endpoints are provided in the manuscript

Plants

Seed stocks	*Report on the source of all seed stocks or other plant material used. If applicable, state the seed stock centre and catalogue number. If plant specimens were collected from the field, describe the collection location, date and sampling procedures.*
Novel plant genotypes	*Describe the methods by which all novel plant genotypes were produced. This includes those generated by transgenic approaches, gene editing, chemical/radiation-based mutagenesis and hybridization. For transgenic lines, describe the transformation method, the number of independent lines analyzed and the generation upon which experiments were performed. For gene-edited lines, describe the editor used, the endogenous sequence targeted for editing, the targeting guide RNA sequence (if applicable) and how the editor was applied.*
Authentication	*Describe any authentication procedures for each seed stock used or novel genotype generated. Describe any experiments used to assess the effect of a mutation and, where applicable, how potential secondary effects (e.g. second site T-DNA insertions, mosiacism, off-target gene editing) were examined.*
